# Selection and geographic isolation influence hummingbird speciation: genetic, acoustic and morphological divergence in the wedge-tailed sabrewing (*Campylopterus curvipennis*)

**DOI:** 10.1186/1471-2148-11-38

**Published:** 2011-02-08

**Authors:** Clementina González, Juan Francisco Ornelas, Carla Gutiérrez-Rodríguez

**Affiliations:** 1 Departamento de Biología Evolutiva, Instituto de Ecología AC, carretera antigua a Coatepec No. 351, El Haya, Xalapa, Veracruz 91070 México; 2 Posgrado en Ciencias Biomédicas, Universidad Nacional Autónoma de México (UNAM), México, D. F., 04510 México

## Abstract

**Background:**

Mesoamerica is one of the most threatened biodiversity hotspots in the world, yet we are far from understanding the geologic history and the processes driving population divergence and speciation for most endemic taxa. In species with highly differentiated populations selective and/or neutral factors can induce rapid changes to traits involved in mate choice, promoting reproductive isolation between allopatric populations that can eventually lead to speciation. We present the results of genetic differentiation, and explore drift and selection effects in promoting acoustic and morphological divergence among populations of *Campylopterus curvipennis*, a lekking hummingbird with an extraordinary vocal variability across Mesoamerica.

**Results:**

Analyses of two mitochondrial genes and ten microsatellite loci genotyped for 160 individuals revealed the presence of three lineages with no contemporary gene flow: *C. c. curvipennis, C. c. excellens*, and *C. c. pampa *disjunctly distributed in the Sierra Madre Oriental, the Tuxtlas region and the Yucatan Peninsula, respectively. Sequence mtDNA and microsatellite data were congruent with two diversification events: an old vicariance event at the Isthmus of Tehuantepec (*c*. 1.4 Ma), and a more recent Pleistocene split, isolating populations in the Tuxtlas region. Hummingbirds of the *excellens *group were larger, and those of the *pampa *group had shorter bills, and lineages that have been isolated the longest shared fewer syllables and differed in spectral and temporal traits of a shared syllable. Coalescent simulations showed that fixation of song types has occurred faster than expected under neutrality but the null hypothesis that morphological divergence resulted from drift was not rejected.

**Conclusions:**

Our phylogeographic analyses uncovered the presence of three Mesoamerican wedge-tailed sabrewing lineages, which diverged at different time scales. These results highlight the importance of the Isthmus of Tehuantepec and more recent Pleistocene climatic events in driving isolation and population divergence. Coalescent analyses of the evolution of phenotypic traits suggest that selection is driving song evolution in wedge-tailed sabrewings but drift could not be rejected as a possibility for morphological divergence.

## Background

Mesoamerica is considered one of the largest and most important biodiversity hotspots in the world, based mainly on the global proportion of vertebrate endemism and the loss of the original primary vegetation cover [1]. Nonetheless, the processes influencing the evolutionary history and diversification within the region are not well understood in most taxa. The Mesoamerican region has been a key land bridge for biotic migrations between North and South America (see for example [2]), and a significant pulse of avian interchange and further radiation occurred in concert with the Isthmus of Panama uplift [3,4]. Geographic barriers, by promoting and maintaining population divergence, are expected to have influenced historical patterns of diversification and the evaluation of such patterns can provide guidance for the preservation and management of genetic diversity and endemism in the region [5]. The Mesoamerican highlands have featured recently in vertebrate mitochondrial DNA phylogeographic studies [6-11]. These studies observed a point of divergence at the Isthmus of Tehuantepec, most noticeably in the rodents and birds that inhabit the mesic highlands. The Isthmus of Tehuantepec, a geologically complex zone that has undergone continental uplift and sea level oscillation since the late Miocene [12,13], has been considered a major biogeographic barrier [14]. The temporary isolation of populations from either side of the isthmus owing to oceanic incursions, and the emergence of the Sierra de los Tuxtlas in the late Pliocene -a volcanic massif 200-250 km away from the Sierra Madre Oriental- along with changes in local environmental conditions that accompanied late Pleistocene glacial cycles, may also explain patterns of biotic diversification in this region.

The wedge-tailed sabrewing (*Campylopterus curvipennis*), a sexually monochromatic, size dimorphic hummingbird species complex commonly found in montane cloud forests and humid tropical forests [15], offers an excellent system for addressing questions about historical biogeography and speciation of Mesoamerican biota. It is one of the few hummingbird species known for both the lowlands and montane region with a geographical disjunction at the Isthmus of Tehuantepec [15]: populations in the foothills of the Atlantic slope of the Sierra Madre Oriental (from south Tamaulipas to north Oaxaca) (SMO), and the Tuxtlas region (Sierra de los Tuxtlas and Sierra de Santa Marta) and a small area on the Isthmus of Tehuantepec (Jesús Carranza and Uxpanapa) (TUX), are separated from those found from northeastern Chiapas to central-south of the Yucatan Peninsula (YUC) (Table 1) [15]. Across their geographic range, they display subtle variation in plumage coloration and bill and body size, which has lead taxonomists to name three subspecies: *Campylopterus curvipennis curvipennis *(SMO), *C. c. excellens *(TUX) and *C. c. pampa *(YUC) [16]. Wedge-tailed sabrewings are remarkable among hummingbirds both because they are one of *c*. 30 out of 320 extant hummingbird species known as lek breeders [17], and because of their elaborate singing displays [18]. In addition, there are marked regional differences in their vocalizations, most noticeably in the introductory syllable and syllable repertoire [18]. Their complex syllable structure exhibits geographic variation that ranges from differences between neighboring males within a lek to differences between lek members separated by several kilometers [18,19].

**Table 1:** Geographic region, coordinates, and altitude of *Campylopterus curvipennis *sampled populations

**Locality**	**Region**	**Latitude N**	**Longitude W**	**Altitude** **(masl)**
	
	*curvipennis *group			
	
1. El Cielo (Ciel)*	nSMO	25° 3'33.66"	99° 12'21.40"	943
2. Gomez Farías (GF)*	nSMO	23° 3'58.26"	99° 10'6.52"	564
3. El Naranjo (Nar)*	nSMO	22° 34'33.33"	99° 21'11.80"	270
4. Aquismón (Aqm)*	cSMO	21° 37'30.87"	99° 1'12.52"	378
5. Xilitla (Xil)*	cSMO	21° 22'39.50"	98° 59'35.77"	637
6. San Bartolo Tutotepec (SBT)	cSMO	20° 21'11.10"	98° 13'10.40"	1155
7. Cuetzalan (Cuet)*	sSMO	20° 0'49.14"	97° 30'21.27"	906
8. Macuiltépetl (Mac)*	sSMO	19° 32'50.51"	96° 55'12.45"	1500
9. Coapexpan (Coap)	sSMO	19° 31'22.90"	96° 58'2.20"	1392
10. Parque Clavijero (Clav)	sSMO	19° 30'47.01"	96° 56'28.64"	1225
11. La Orduña (Ord)*	sSMO	19° 27'50.94"	96° 56'13.05"	1190
12. El Riscal (Risc)	sSMO	19° 28'47.40"	96° 59'51.00"	1586
13. Ursulo Galván (UG)*	sSMO	19° 25'31.48"	96° 58'35.20"	1200
14. Xico (Xico)	sSMO	19° 24'37.99"	96° 59'31.81"	1350
15. Amatlán (Ama)	sSMO	18° 49'51.84"	96° 54'7.78"	720
	
	*excellens *group			
	
16. Los Tuxtlas (Tux)*	TUX	18° 33'29.05"	95° 11'46.00"	998
17. El Nopal (Nop)	TUX	17°14'23.5"	90°45'40.5"	664
18. Chalchijapa (Chal)	TUX	17°2'4.07"	94°41'57.85"	260
	
	*pampa *group			
	
19. Escárcega (Esc)	YUC	18° 38'12.5"	90° 47'16.89"	60
20. Río Bec (RB)	YUC	18° 24'31.4"	89° 26'37.2"	251
21. Ejido 20 de Noviembre (Nov)*	YUC	18° 25'29.0"	89° 18'37.3"	179
22. Tres Garantías (Gar)	YUC	18° 12'51.2"	89° 02'34.8"	137

Geographic regions correspond to three disjunct areas along the Sierra Madre Oriental, the Tuxtlas region and Yucatan Peninsula (see also Figure 2). Asterisk indicates populations on for acoustic recordings were made.

Species with highly differentiated populations may represent the early stages of the speciation process. In these populations, drift, ecological selection or both can induce changes in traits involved in mate choice, promoting reproductive isolation between allopatric populations that can eventually lead to speciation [20-23]. The sole action of drift and mutation can promote phenotypic differentiation or reproductive isolation [24]. In contrast, ecological speciation can occur when divergent selection pressures act generally on the morphological or behavioral characters of species distributed on heterogeneous habitats, where gene flow among locally adapted populations is reduced [25]. For acoustic traits, microclimate and vegetation structure can be important selective pressures on the transmission of acoustic traits of birds living in different habitats [26,27]. Another possibility is that within populations, stochastic changes in traits driven by processes such as sexual selection promote reproductive isolation between geographically isolated populations where divergent natural selection is not acting [28,29].

To better understand the patterns of diversification and endemism in Mesoamerica, we use mitochondrial (mtDNA) and nuclear (microsatellites) DNA data to infer the processes behind the evolutionary history of *C. curvipennis*. The use of both bi-parentally inherited molecular markers with different mutation rates is important because it allows us to make inferences at different temporal scales and provides more reliable insight into the historical processes involved in the evolution of taxa than studies based on the analyses of a single marker do. In addition to genetic data (mtDNA and microsatellites as a proxy for neutral processes; [30]), we used morphological and acoustic data along with information about the habitat (climate and topography) to examine the relative roles of drift and selection in driving population divergence. The specific goals of this study were to: (i) examine the patterns of genetic variation and demographic history of *C. curvipennis *populations, (ii) infer the processes behind its evolutionary history, specifically assessing the role of the Isthmus of Tehuantepec as a barrier, and (iii) evaluate the role of drift and selection in driving phenotypic (morphology and song) divergence in wedge-tailed sabrewings. Patterns resulting from intraspecific geographical variation in phenotypic traits and genetic markers should provide insights into the factors driving population differentiation and ultimately speciation in Mesoamerica.

## Results

### Sequence variation and phylogenetic analyses

From 160 wedge-tailed sabrewings we obtained mtDNA sequences that contained 81 polymorphic sites (38 in the ATPase and 43 in the control region) of the 1,407 bp of the genes analyzed. No insertions or deletions were present; therefore, variants were identified based solely on nucleotide substitutions. Sixty-three haplotypes were identified for the 22 wedge-tailed sabrewing populations. Haplotype and nucleotide diversity are summarized in Table 2. Haplotype proportion relative to the number of samples per population was more than 50% in most cases, indicating high haplotype diversity. No differences between populations were evident. Nucleotide diversity was low, indicating little variation between sequences from the same population.

**Table 2:** Population genetic variability of *Campylopterus curvipennis*

		Microsatellite DNA				Mitochondrial DNA			
			
Locality	*n*	Mean alleles /locus	Allelic Richness	*H*_O_	*H*_E_	H	S	*Hd*	π
*curvipennis *group									

El Cielo	18	5.6	1.60	0.47*	0.59	6	9	0.83	0.0021
Gomez Farías	4	3.6	1.61	0.65	0.61	2	1	0.5	0.0035
El Naranjo	6	3.6	1.58	0.55	0.57	6	8	1	0.0020
Aquismón	4	3.5	1.63	0.55	0.63	2	1	0.5	0.0003
Xilitla	8	5	1.64	0.49*	0.64	6	7	0.92	0.0013
S. B. Tutotepec	1	1.4	1.40	-	-	1	0	-	-
Cuetzalan	27	6.2	1.62	0.57	0.62	17	20	0.92	0.0020
Macuiltépetl	3	2.66	1.55	0.55	0.57	3	5	1	0.0023
Coapexpan	6	4.5	1.65	0.18	0.30	6	11	1	0.0027
Parque Clavijero	7	4.1	1.60	0.46	0.60	7	8	1	0.0023
La Orduña	22	6.2	1.63	0.49*	0.63	11	14	0.819	0.0020
El Riscal	2	2.3	1.60	0.45	0.60	2	1	1	0.0007
Ursulo Galván	13	5.3	1.60	0.48†	0.60	6	8	0.85	0.0014
Xico	4	2.8	1.61	0.50	0.61	2	8	0.83	0.0033
Amatlán	3	3.1	1.62	0.47	0.62	3	6	1	0.0028

*excellens *group									

Los Tuxtlas	10	4.7	1.61	0.51	0.61	5	5	0.66	0.0007
El Nopal	1	1.3	1.30	-	-	1	0	-	-
Chalchijapa	1	1.7	1.70	-	-	1	0	-	-

*pampa *group									

Escárcega	1	1.5	1.50	-	-	1	0	-	-
Río Bec	2	1.9	1.45	0.45	0.45	2	2	1	0.0014
20 de Noviembre	15	5.5	1.57	0.46*	0.57	11	12	0.96	0.0027
Tres Garantías	3	2.7	1.56	0.4	0.56	2	5	0.66	0.0020

Estimates based on ten microsatellite loci, and 1407 bp of the control region and ATPase 6-8 mtDNA genes. *n *= sample size, observed (*H*_O_) and expected heterozygosity (*H*_E_), H = number of haplotypes observed, S = number of polymorphic sites, *Hd *= haplotype diversity, and π = nucleotide diversity. Asterisks indicate significant departure (*P *< 0.05, after a sequential Bonferroni correction) from Hardy-Weinberg equilibrium for locus CACU13-2 and † for locus CACU13-7.

**Figure 1:**
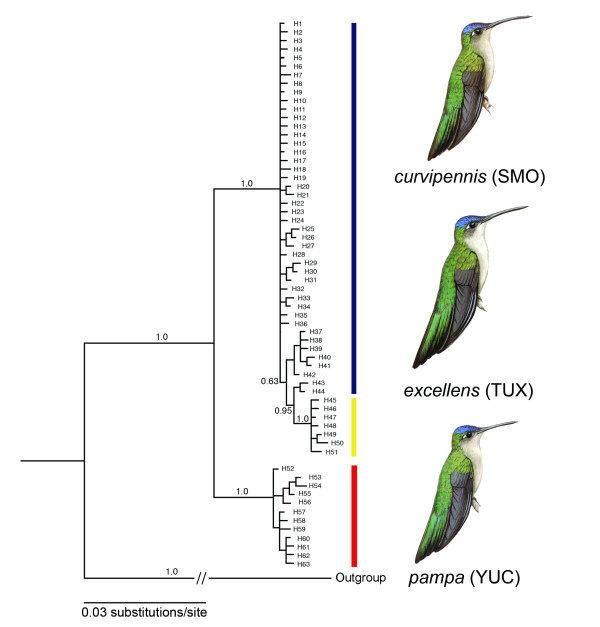
**Phylogenetic relationships among mtDNA haplotypes based on Bayesian inference**. Values above branches denote posterior probabilities, and numbers at the tip of the branches indicate distinct haplotypes. Outgroups were *Campylopterus rufus, C. hemileucurus*, and *C. largipennis*, and are shown collapsed into a single branch. Hummingbird illustrations were taken from color plates in the *Handbook of the Birds of the World *[32].

The consensus tree obtained from Bayesian inference clustered the haplotypes into two main well-supported clades (posterior probabilities of 1.0), corresponding to disjunct western (*curvipennis*, SMO) and eastern (*pampa*, YUC) groups on either side of the Isthmus of Tehuantepec (Figure 1). Haplotypes of the *excellens *group from the Tuxtlas region were clustered in a well-supported clade nested within the poorly resolved SMO clade.

Assuming a constant molecular clock and rates of 2 and 5% divergence per My, for the ATPase coding region BEAST estimated that the SMO and TUX clades diverged from the YUC clade 1.47 (0.35-3.42) and 0.52 (0.13-1.21) Mya, respectively. These results suggest that the split between the Sierra Madre Oriental and Yucatan Peninsula clades may have occurred during the mid-Pleistocene. With respect to SMO, for the TUX clade the constant clock TMRCA estimated divergence times of 614,000 (0.12-1.55 Mya) and 202,000 (0.05-0.47 Mya) years ago for 2 and 5% substitution/My, respectively. This implies that the split between the TUX and SMO clades may have occurred more recently in the late Pleistocene.

### Phylogeographic and genetic structure

**Figure 2:**
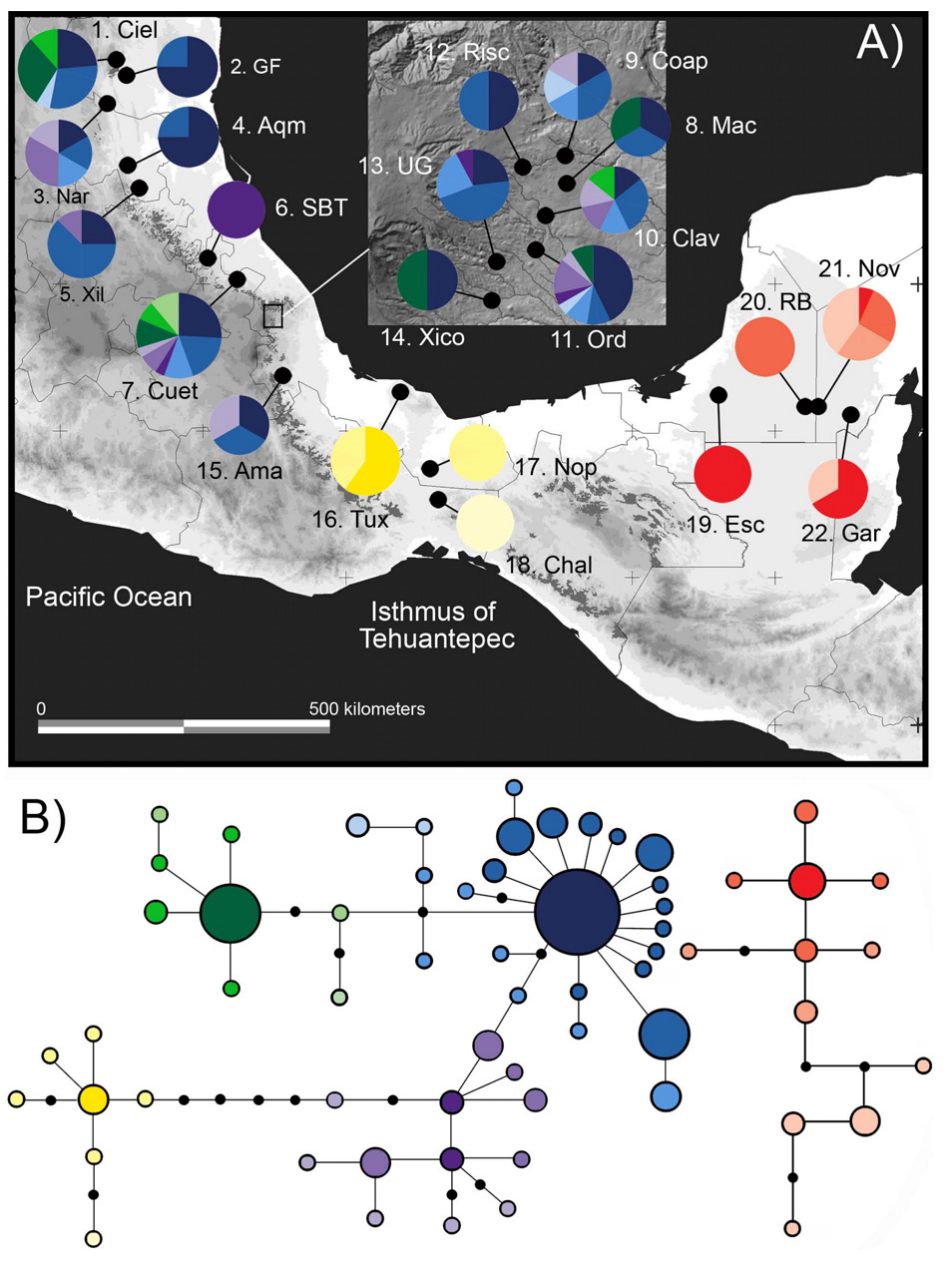
**Sampling localities, geographic distribution, and genealogical relationships of mtDNA haplotypes**. **A**. Pie charts indicate the frequency of occurrence of haplotypes in each population, and colors correspond to those shown in the haplotype network below. The current natural range of cloud forest (indicated by dark gray shading) is overlaid on a relief map of eastern Mexico. **B**. Statistical parsimony haplotype network derived from a combined matrix of mitochondrial genes (ATPase 6-8 and control region). Each circle represents a haplotype and its size is proportional to its frequency. Lines represent a single mutational change and black circles correspond to unsampled haplotypes.

The haplotype network showed a strong phylogeographic structure among three groups (SMO, TUX, YUC), but not among populations or areas within the SMO (nSMO, cSMO, sSMO) (Figure 2). Of the 63 haplotypes obtained, 44 were private to SMO populations. The most frequent haplotype was shared by samples from all of the SMO populations, and the rest were low frequency haplotypes (Figure 2). Seven haplotypes were private to the Tuxtlas region, and are separated from the SMO haplotypes by five mutational steps. The remaining haplotypes (12) were exclusively found in populations from the Yucatan Peninsula, and formed a separate network (Figure 2).

The AMOVA results revealed significant genetic differentiation at every hierarchical level. When grouped by geographic areas (nSMO, cSMO, sSMO, TUX, and YUC; see Table 1 and field procedures for the definitions) most of the variation (85%) was explained by differences among areas, whereas variation among populations within areas (0.92%) and variation within populations (13.94%) only explained a small percentage of the total variation (Table 3). The same pattern was observed when grouped by subspecies (*curvipennis*, SMO; *excellens*, TUX; and *pampa*, YUC) with the highest percentage of the variation being explained by differences among groups (90.41%) and the lowest by differences among populations within groups (0.55%; Table 3). Φ_CT _values for both analyses were very high (Table 3) indicating high levels of genetic differentiation among areas and groups. Pairwise comparisons of *F_ST _*among sampling localities ranged from 0.003 for Aqm/Ama to 0.95 for UG/Gar and were significant in most cases between sampling localities from the TUX region and the other localities, and for the YUC region and the other localities [Additional file 1]. Comparisons between sites located in the same geographic area (SMO, TUX, YUC) were not significant [Additional file 1].

**Table 3:** Analysis of molecular variance (AMOVA) for the control region and ATPase 6-8 mtDNA genes of *Campylopterus curvipennis*

Structure	Source of variation	Variation (%)	Fixation indices
Five areas	Among areas	85.13	Φ_CT _0.85**
	Among populations within areas	0.92	Φ_SC _0.06**
	Within populations	13.94	Φ_ST _0.86**
Three groups	Among groups	90.41	Φ_CT _0.90**
	Among populations within groups	0.55	Φ_SC _0.05**
	Within populations	9.05	Φ_ST _0.91**

Data were grouped by geographic area and subspecies. The five areas are nSMO, cSMO, sSMO, TUX and YUC. Groups are *curvipennis *(SMO), *excellens *(TUX), and *pampa *(YUC). ** *P *< 0.001.

### Microsatellite markers

Across all sampling localities, the number of alleles per locus varied from 4 to 20, and within localities across all loci this number varied from 13 to 62 (mean alleles per locus, 1.3-6.2) (Table 2). Observed heterozygosity values did not consistently deviate from H-W equilibrium. Only four localities were not in H-W equilibrium after Bonferroni corrections at locus CACU13-2 and one locality at locus CACU13-7 (Table 2) probably due to the presence of null alleles. No significant linkage disequilibrium was detected in any of the population-loci comparisons after Bonferroni corrections.

Significant genetic subdivision was detected among sampling localities (global *R_ST _*estimate ± SE, 0.085 ± 0.0021, *P *< 0.0001), but not among sampling localities within the fragmented areas of the SMO (global *R_ST _*estimate ± SE, -0.020 ± 0.0021, *P *> 0.05). Pairwise *F_ST _*and *R_ST _*were quantitatively similar but we only report *R_ST _*values because the stepwise mutation model implemented in this estimate is more appropriate for microsatellites [Additional file 1]. Pairwise *R_ST _*values among sampling localities ranged from 0.001 for Xil/Ord to 0.42 for Risc/Nov. As with the pairwise *F_ST _*values for the mitochondrial genes, estimates of *R_ST _*were significant for almost all comparisons between localities from TUX *versus *the rest of the localities and comparisons between localities from YUC *versus *the rest after Bonferroni corrections. Values from comparisons between sampling localities within SMO were not statistically significant. Comparisons between samples from Bec and Gar (from the YUC region) *versus *the rest were not significant in most cases after Bonferroni corrections probably due to sample size, affecting statistical power.

The phenogram constructed with pairwise *R_ST_*, recovered sampling localities from the YUC group clustering together in a basal position followed by the TUX and the SMO groups [Additional file 2]. Within the SMO group, samples from the northern limit of the distribution (Tamaulipas) are clustered together, as are samples from San Luis Potosi (central distribution). However, localities from the southern distribution (Veracruz and Puebla) are scattered throughout the tree [Additional file 2].

**Figure 3:**
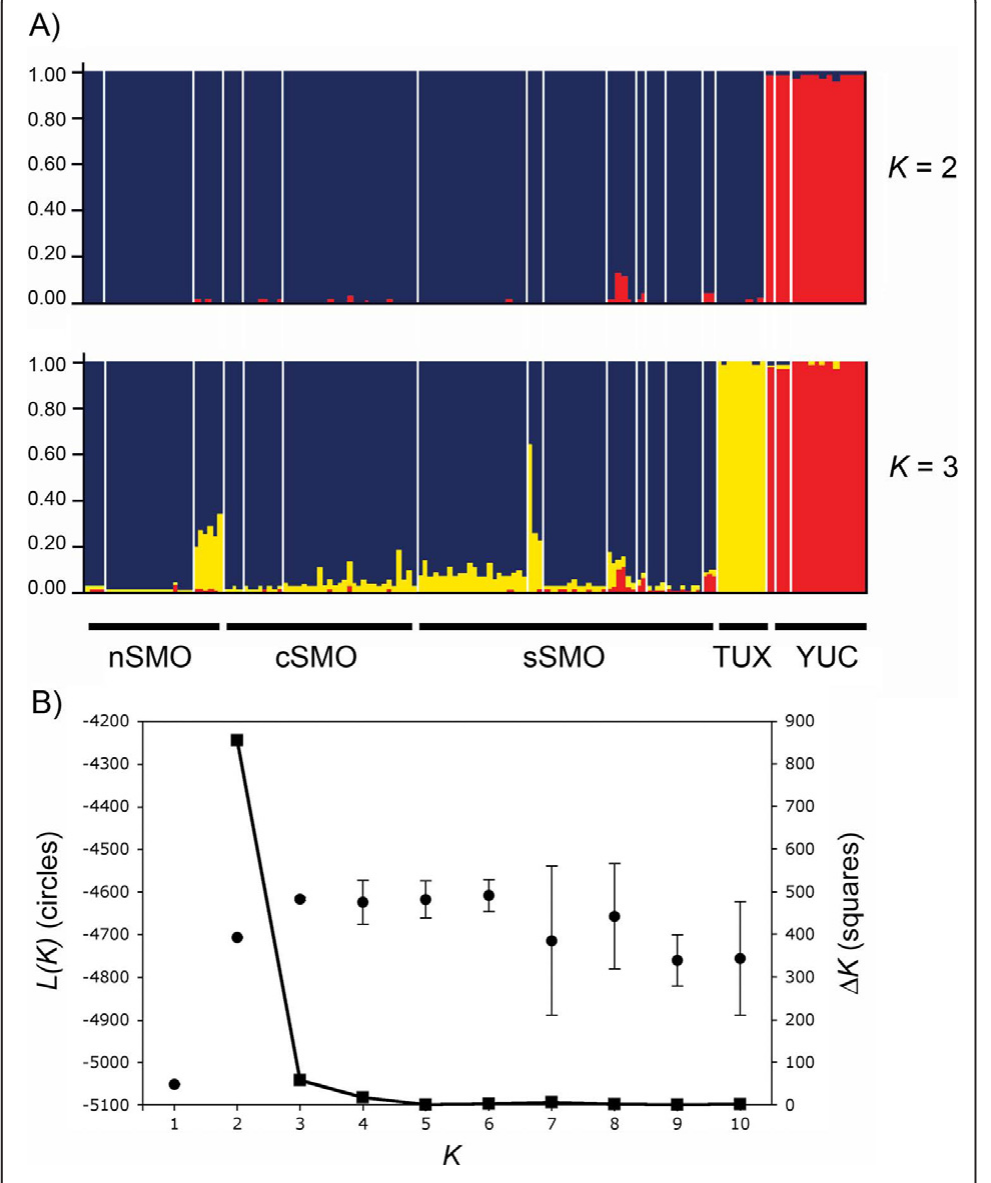
**Spatial genetic structure determined by a Bayesian assignment analysis using STRUCTURE**. **A**. Posterior assignment probabilities of 160 individuals of *Campylopterus curvipennis *to an optimal number of *K *= 2 and *K *= 3. Each individual is represented by a vertical line that is partitioned into *K *colored sections, with the length of each section proportional to the estimated membership coefficient. **B**. Mean log probability of the data (*L(K*) ± SD) over 10 runs for each *K *value (left Y axis) and values of Δ*K *calculated according to Evanno *et al. *[90] (right Y axis).

Results of the STRUCTURE analysis corroborated population substructure. Using all genotypes together the log likelihood was highest for *K *= 6; however, when Δ*K *is calculated the break in the slope of the distribution of *L(K) *was at *K *= 2 (Figure 3). One cluster includes populations from the Yucatan Peninsula (YUC), and the other includes samples from populations from the TUX and SMO groups (Figure 3). However, in the clustering pattern at *K *= 3, samples were assigned with high probabilities to three clusters, corresponding to the YUC, TUX and SMO groups, which is congruent with the sequence mtDNA data results. In further analyses of these data from which the YUC samples were first excluded, STRUCTURE assigned samples to two groups (TUX and SMO); in the analysis where the TUX samples were excluded two clusters were detected for the SMO samples with no evidence of genetic clustering at this level. These results suggest a hierarchical clustering pattern where YUC samples are highly divergent and samples from TUX are sub-structured in a cluster that contains samples from SMO.

Among-group comparisons of gene flow (M) from the MIGRATE analysis ranged from 0.49 (TUX to YUC) to 1.98 (TUX to SMO), however, all comparisons were less than or not significantly greater than 1.0 (Table 4). None of the comparisons among genetic groups indicate contemporary gene flow, suggesting that the Isthmus of Tehuantepec is preventing gene flow between populations on either side of the isthmus, and that the more recently divergent populations from the Tuxtlas region and SMO also have interrupted gene flow. Estimates of M among populations were only significantly greater than 1.0 between those within the SMO (M values ranged between 1.4 and 2.3) and the direction of gene flow was asymmetric in most cases. The direction was mainly northwards from sSMO to cSMO, and only in few cases did gene flow reached populations on the northern limit of the distribution (Ciel, GF). Estimates of gene flow between populations of different genetic groups were not significantly greater than 1.0.

**Table 4:** Estimates of M (mutational corrected migration) from the MIGRATE analysis of microsatellites among genetic groups

	**SMO**	**TUX**	**YUC**
	
**SMO**	-	1.48 (0.58 - 4.22)	0.58 (-0.1 - 1.56)
**TUX**	1.98 (0.54 - 3.81)	-	0.49 (-0.68 - 3.39)
**YUC**	0.91 (0.03 - 2.29)	0.63 (-0.25 - 1.58)	-

Donor populations are in the first column. Estimates given are followed by 95% confidence intervals and none of the comparisons was significantly greater than 1.

Our simulations to test whether genetic groups might have originated in the face of gene flow were consistent across replicates, and produced confident posterior probability peaks for the parameters estimated. When testing for migration following the split between the SMO and TUX groups, migration rates were higher than 1 in the SMO-TUX direction (*m *= 1.416, 90% highest posterior density (HPD), 0.004-4.21) and close to 1 in the opposite direction TUX-SMO (*m *= 0.91, 90% HPD, 0.007-4.47), suggesting that divergence took place in the presence of gene flow from the SMO to the Tuxtlas region. In contrast, low migration rates following the split were estimated for populations east and west of the Isthmus of Tehuantepec (west: *m *= 0.52, 90% HPD, 0.003-1.27; east: *m *= 0.36, 90% HPD, 0.002-1.23), indicating that the population split occurred in the absence of gene flow, i.e. this geographic barrier was not permeable to migrants. Assuming a mutation rate for microsatellites of 2.96 × 10^-3^, estimates of effective population size indicate that the ancestral population was significantly larger (3,924 individuals, 90% HPD, 2,598-19,005) than the populations after divergence. The TUX group had the lowest population size (103.88 individuals 90% HPD, 38.01-259.3) followed by YUC (175.67 individuals 90% HPD, 96.28-282.93) and SMO (326.01 individuals 90% HPD, 203.54-474.66).

### Morphological and acoustical variation

**Figure 4:**
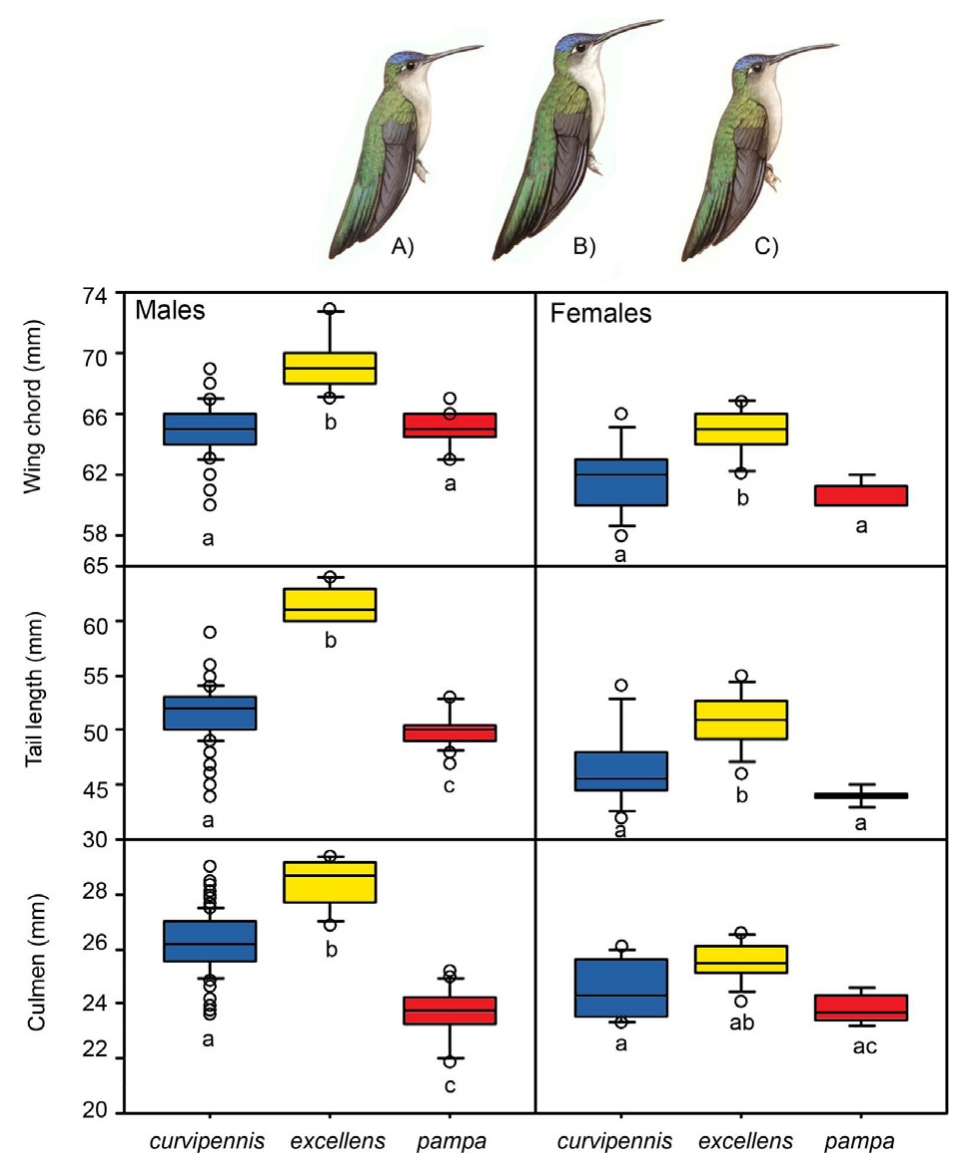
**Morphological traits taken for males and females of *C. curvipennis *lineages**. Boxplots show the 10^th^, 25^th^, 50^th ^(median), 75^th^, and 90^th ^percentiles. Values above the 90^th ^and below the 10^th ^percentile are plotted as open circles. Illustrations correspond to *C. c. curvipennis *(**A**), *C. c. excellens *(**B**) and *C. c. pampa *(**C**) taken from the *Handbook of the Birds of the World *[32].

The MANOVA showed significant lineage differences in male morphology (Wilks' Lambda, *F*_6,222 _= 33.77, *P *= 0.0001), and these differences were significant for each morphological variable (one-way ANOVAs: wing chord *F*_2,113 _= 16.71, *P *= 0.0001; tail length *F*_2,113 _= 48.45, *P *= 0.0001; culmen *F*_2,113 _= 57.58, *P *= 0.0001, SMO *n *= 106, TUX *n *= 6, YUC *n *= 16; Figure 4). The relationship between culmen length and wing chord (as a measure of body size; *r *= 0.38, *P *< 0.0001) was further examined for possible allometric effects using the residuals of this relationship, and the differences between lineages were maintained (*F*_2,113 _= 48.33, *P *< 0.0001). For females the MANOVA also showed significant lineage differences (Wilks' Lambda, *F*_6,42 _= 3.89, *P *< 0.005), and the results of the univariate ANOVAs were all significant (wing chord *F*_2,23 _= 10.17, *P *< 0.001; tail length *F*_2,23 _= 11.94, *P *< 0.0005; culmen *F*_2,23 _= 4.65, *P *< 0.05, SMO *n *= 21, TUX = 11, YUC *n *= 5; Figure 4). Overall these results are congruent with previous subspecific designation based on bill and body size [31,32], indicating that individuals from the *excellens *group (TUX) are significantly larger than those of the *curvipennis *(SMO) and *pampa *(YUC) groups, and that *pampa *individuals have shorter bills.

**Figure 5:**
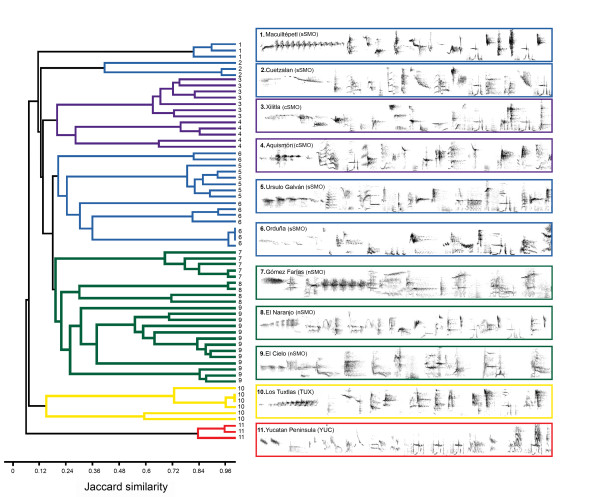
**Dendrogram generated by cluster analysis of a presence/absence matrix of syllable types from recordings of individuals**. Colored lines correspond to individuals recorded at different geographic locations: green, purple and blue correspond to sites located in the northern, central and southern parts of the Sierra Madre Oriental respectively (nSMO, cSMO, and sSMO) for the *C. c. curvipennis *lineage, yellow corresponds to the Tuxtlas region for the *C. c. excellens *lineage and red corresponds to the Yucatan Peninsula for the *C. c. pampa *lineage. Attached to the dendrogram fragments of vocalizations (4 sec) representing each sampled site are shown.

A total of 344 syllable types were detected across populations. Songs were very versatile and no successive syllable repetitions were detected except for vocalizations recorded in the YUC region from the *C. c. pampa *lineage, where individuals commonly repeated one of the syllables three times in succession (Figure 5). Based on vocal similarity measures (syllable type sharing), individuals from each sampling locality were clustered accordingly (Figure 5). In addition, the analysis showed individuals from the YUC lineage group together in a basal position followed by individuals from the TUX and SMO lineages (Figure 5). On average populations from SMO shared a lower proportion of syllable types with the TUX and YUC lineages (0.062 ± 0.018), than within any other locality from the SMO (0.145 ± 0.037). Despite the great syllable diversity observed across populations and regions, there were some syllables shared by all recorded individuals, whereas other were shared only by members from populations from the SMO. Regarding acoustic measurements of the common syllable, the first three PCs accounted for 53% of variation (PC1 = 24.4%, PC2 = 18.2%, PC3 = 10.4%). One-way ANOVAs yielded significant group differences in the first PC (PC1, *F*_2,74 _= 107.05, *P *< 0.0001), but not in the second and third (PC2, *F*_2,74 _= 2.13, *P *> 0.05; PC3, *F*_2,74 _= 1.01, *P *> 0.05 SMO *n *= 66, TUX = 6, YUC *n *= 5). PC1 was mainly explained by minimum and peak frequency of note 1, duration of note 3, and frequency range, peak frequency and duration of note 4.

### Comparisons between morphological, acoustic, habitat-related, and genetic distances

Mantel tests showed a strong positive correlation between genetic (pairwise *F_ST_*) and morphological distances for males (*r *= 0.42, *P *< 0.05), even when controlled for geographic distance (*r *= 0.38, *P *< 0.05). However, the relationships between pairwise *R_ST _*and morphology were not significant. In the case of females none of the relationships were significant. Regarding acoustic distances, Mantel test showed a strong positive correlation between genetic and song sharing distance (*F_ST_*: *r *= 0.72, *P *< 0.05; *R_ST_*: *r *= 0.71, *P *< 0.05), and also for the genetic and common syllable distance (*F_ST_*: *r *= 0.72, *P *< 0.01; *R_ST_*: *r *= 0.72, *P *< 0.01). These relationships were maintained when geographic distance was accounted for (song sharing: *F_ST_, r *= 0.50, *P *= 0.05; *R_ST_, r *= 0.51, *P *< 0.05; common syllable: *F_ST_, r *= 0.44, *P *< 0.05, *R_ST_, r *= 0.44, *P *= 0.01). These analyses indicate that more genetically divergent lineages shared fewer syllable types, and differed acoustically in the common syllable, independently of distance. Lastly, we did not find a significant relationship between morphological and acoustic distances *versus *habitat-related (climate and topography) distances for either males or females.

### The role of drift and selection: a coalescent test

Coalescent simulations [30] of morphology and mtDNA sequence data from three populations with large sample sizes of sequences corresponding to the three genetic groups in which morphological characters are fixed (Tux, Ciel, Nov) were used to differentiate the roles of selection and genetic drift in morphology evolution. If morphological characters, assumed to be encoded by nuclear genes, have sorted significantly faster than mtDNA haplotypes, then the hypothesis of divergent selection rather than that of drift is supported. The observed *s *value for reconstructed trees was equal to 2 when considering three populations for morphology characters. The upper 95% CL for time since population divergence was 3*N_e _*generations assuming a dichotomous branching model of divergence and 3.3*N_e _*generations assuming a simultaneous model. When branch lengths were reduced four times to mimic nuclear genes, an *s *value of 2 occurred in one third of the simulated trees for dichotomous branching, and in one fifth of the simulated trees for simultaneous branching. There is a high probability (*P *> 0.05) that nuclear genes would be fixed in these populations under neutrality, suggesting that drift cannot be rejected as a possibility for morphological divergence [Additional file 3].

The coalescent simulations of the song and mtDNA sequence data from four populations with large sample sizes of sequences in which song characters are fixed (Ciel, Ord, Tux and Nov) were used to differentiate the roles of selection and genetic drift in song evolution. The observed *s *value for reconstructed trees was equal to 8 when considering four populations for vocal characters. The upper 95% CL for time since population divergence was 0.48*N_e _*generations assuming a dichotomous branching model of divergence and 0.68*N_e _*generations assuming a simultaneous model. When branch lengths were reduced four times, an *s *value of 3 occurred in none of simulated trees, suggesting that there is a very low probability (*P *< 0.0001) that nuclear genes would be fixed in different populations under neutrality [Additional file 3]. In this case the hypothesis of divergent selection rather than that of drift is supported.

## Discussion

### Phylogeography and evolutionary history

Phylogeographic analyses on mtDNA sequence data and microsatellites were highly congruent identifying three wedge-tailed sabrewing lineages: the *pampa *group in the Yucatan Peninsula, the *curvipennis *group along the Sierra Madre Oriental, and the *excellens *group in the Tuxtlas region. The patterns of genetic differentiation inferred here for *C. curvipennis *are consistent with the results for other codistributed avian species in montane cloud forests of the Sierra Madre Oriental and the Tuxtlas region such as *Chlorospingus ophthalmicus *[9] and *Buarremon *finches [11]. Even though the TUX mtDNA haplotypes formed a clade nested within the more diverse haplotype clade of the Sierra Madre Oriental, these haplotypes are not shared with individuals from any other population, and are separated by several mutational steps from the SMO haplotypes. This suggests that not enough time has elapsed for lineages to sort out the formation of two reciprocally monophyletic clades [33]. Similarly, the cluster analysis with microsatellites identified the *pampa *group when *K *= 2 and *excellens *when *K *= 3, indicating a lesser degree of divergence between the TUX and SMO genotypes in comparison to those of YUC. Consistent with the lack of haplotype sharing and high pairwise *R_ST _*values, the results of gene flow measures based on microsatellites indicate that the SMO, TUX and YUC populations exchange no migrants. Both mtDNA and biparentally inherited microsatellite loci are spatially structured between allopatric populations of wedge-tailed sabrewings suggesting that gene flow is restricted and that *C. c. curvipennis, C. c. excellens*, and *C. c. pampa *constitute genetically unique populations.

It is possible that allopatric fragmentation has affected the genetic structure of wedge-tailed sabrewings. Although the existence of several mutational steps in a haplotype network is indeed indicative of allopatric fragmentation, it could also result from non-sampled haplotypes from intermediate populations. The latter is not the case here, as we sampled populations covering the entire geographical range of the species complex [Additional file 4]. In addition, the possibility that allopatric fragmentation has been a historical process causing the pronounced mtDNA divergence between the *pampa *and the *curvipennis-excellens *groups is consistent with the hypothesis that the Isthmus of Tehuantepec was a major vicariant event and has been a significant habitat barrier to dispersal, as observed in previous mtDNA phylogeographical studies with montane bird species [7,9-11]; however, our study is the first to document the impact of this barrier in a species complex with lowland populations on either side of the isthmus. The isthmus, formed by the superposition of three distinct tectonic episodes, has been exposed to continental uplift and sea level oscillation since the late Miocene (*c*. 6 Ma; [12]), periodically encroaching upon or retreating from the coastal plains, with the peak in sea level occurring at the end of the Pleistocene [34]. The phylogeographic breaks in our genetic data suggest a split between the SMO-TUX and YUC clades in the mid-Pleistocene (1.4 - 0.56 Ma) toward the end of the Isthmus of Tehuantepec's process of formation. Despite the variability in date estimation using the coalescence approach, our divergence time estimates point to the Pleistocene epoch, and these estimates are consistent with the timing of one of the two pulses of diversification proposed by Barber and Klicka [14] across the isthmus for a community of montane bird species. Our results from gene flow and isolation with migration (IMa) support the role of the isthmus as an effective barrier in which no migration occurred during genetic divergence.

Regarding the most recent divergence between the SMO and TUX populations, the oscillations in climate during the Pleistocene caused a considerable expansion in the range of highland habitats and their descent to lower elevations [35,36]. These repeated altitudinal up- and downslope migrations of montane forest during glacial periods probably connected the cloud forests of the SMO with the TUX montane region, and as temperatures increased, the redistributed forested habitat caused the isolation of the TUX populations. This idea is supported by our results from IMa, which suggests that this vicariant event occurred in the face of gene flow, from populations of the SMO to those of the Tuxtlas region, but not in the opposite direction, indicating that the connection of the forests allow individuals to disperse towards the Tuxtlas region. However, estimates of contemporary migration rates indicate that contemporary gene flow is restricted between these two groups. The current distribution of coastal plain along the Gulf of Mexico, which surrounds and isolates the Tuxtlas region from the Sierra Madre Oriental, could be a dispersal barrier between wedge-tailed sabrewing populations. Because microsatellites have a faster mutation rate than mitochondrial genes and therefore these markers have very different temporal scales of inference, our measures of gene flow based on microsatellite loci indicate that contemporary gene flow is prevented by both barriers (the isthmus and the coastal plain). The absence of suitable habitat on the coastal plains along with the presence of pastures and savanna-like habitats in the isthmus region likely prevents population expansion that could result in secondary contact between the SMO, TUX and YUC populations. Hence, gene flow would require long distance dispersal for wedge-tailed sabrewings to overcome these barriers.

### Morphological and acoustical variation: the role of drift and selection

Morphological data for both males and females indicated that individuals from TUX were significantly larger than those from SMO and YUC, and individuals from SMO and TUX had longer bills than those from YUC. The lack of correspondence between climate and topographic conditions and morphological traits suggests that weak habitat selection pressures have been shaping morphological divergence. This inference should be made with caution, considering that habitat structure was characterized using only ground-based interpolated climate and topography data. Information about vegetation structure obtained using satellite and airborne remotely sensed images would offer a more powerful way for testing adaptive and nonadaptive hypotheses concerning the evolutionary processes that operate across the environmentally heterogeneous space occupied by wedge-tailed sabrewings. However, these data require substantial processing for applications in spatial analyses and are sensitive to cloud contamination [37]; these data may not be useful for regions with cloud forest and rainforest.

When conducted a coalescent-based test using mtDNA sequence data to evaluate whether the observed phenotypic differentiation can be attributed to drift or some form of selection, we could not reject the possibility of drift in driving morphological differentiation of the SMO (*C. c. curvipennis*), TUX (*C. c. excellens*), and YUC (*C. c. pampa*) populations. Besides, independently of geographic distance, a significant positive relationship between genetic and morphological distances was found highlighting the idea that drift might facilitate phenotypic divergence when acting in concert with selection [38,39]. The increase in body size in small peripheral populations can be driven by random genetic drift [38-40]. In addition, the effective population size of wedge-tailed sabrewings in the TUX region is one-third and one-half smaller compared to the SMO and YUC groups, respectively. Another possible explanation for the increased body size of the TUX individuals is the relaxed competition for resources that is expected on islands [40] or in isolated populations such as those in the TUX region, which are competing for resources with fewer hummingbird species. If so, this would suggest that individuals from the TUX region have evolved independently and unconstrained by the evolutionary events that occurred in the other regions [38].

The increase in culmen length in the SMO and TUX regions may indicate selection-driven divergence, and the effect of adaptation to local resources (e.g. flower size and shape) through natural selection. Differences in culmen length are congruent with the differences in habitat characteristics of the Yucatan Peninsula (drier deciduous tropical forests at a much lower elevation) compared to the higher altitude and wetter habitats of the Sierra Madre Oriental and the Tuxtlas region, suggesting that populations adapted to different ecological conditions, including perhaps the presence of flowers with longer corollas. Differences in environmental variables provide the potential for ecological differentiation where different selection pressures might act to shape the relationship between pollinator and plant species [41]. However, a comparative study involving traits of flowers with hummingbird pollination syndrome occurring in each region and the culmen characteristics of hummingbirds using those resources need to be done.

The divergence of mating-related acoustic signals has been described as being shaped by habitat-dependent selection in many bird species [42-47], because sounds with certain features are better transmitted in some environments than others. In wedge-tailed sabrewings, some populations live in different habitats with divergent environmental conditions, most markedly in the *pampa *lineage from the Yucatan Peninsula whose individuals live in deciduous tropical forest at a lower altitude and much drier conditions than populations from the other lineages, which inhabit cloud or tropical forests at higher altitudes that have wetter conditions. However, we found no significant correlations between acoustic and habitat-related distances (climate and topographic variables), which suggest that these conditions play a minor role in shaping song divergence.

In contrast, we found a strong acoustic divergence pattern in syllable sharing between SMO populations and also between geographic areas, the latter corresponding to the genetic differentiation among the three lineages of wedge-tailed sabrewings. Each lineage had an exclusive assemblage of syllable types, and song sharing was lower between than within lineages. Also, the acoustic traits of a common syllable were more divergent between than within lineages. The coalescent analysis to evaluate whether vocal differentiation can be attributed to drift or selection, suggested that the fixation of song types assumed to be encoded by nuclear genes, has occurred faster than expected by genetic drift, providing evidence that selection is driving song evolution in wedge-tailed sabrewings. In lek-breeding systems where male birds are exposed to strong sexual selection pressures, the use of certain song types over others could increase their adaptation to social conditions and/or reproductive success. Although song could potentially play a role in increasing genetic polymorphisms and generating reproductive isolation and speciation through sexual selection [21,48,49], further evidence of female preference for local male signals over foreign ones is needed to fully demonstrate that sexual selection is causing speciation in wedge-tailed sabrewings (see [50]). An alternative cause of divergence of songs in wedge-tailed sabrewings is reinforcement selection against maladaptive hybridization, being song a sexually selected mechanism to maintain reproductive isolation in case of secondary contact [51]. However, it is unlikely that the wedge-tailed sabrewings from these populations come into contact owing to the lack of contemporary gene flow shown in this study, which indicates a historically low potential for hybridization.

The positive relationship between genetic and acoustic distances suggests that independently of geographic distance, the most genetically distant lineages shared fewer vocal elements and differed in the acoustic traits of the common syllable. This relationship is expected under a drift model of song evolution, where acoustic divergence is higher between populations that have been genetically isolated for the longest time [45]. As in songbirds, song is learned in hummingbirds [52]. Likely song traits are culturally transmitted through imitation, and learning errors or innovations (new syllables) across generations can provide an important source of variation [21,53] affected by processes similar to those driving genetic variation, such as drift or selection [54]. In the case of drift, dialects or song variation at spatial scales could arise from a process of cultural diversification where random song mutations are produced by copy errors [55]. It has also been suggested that song learning may increase the rate at which genetic predisposition to learn or prefer certain songs evolves in allopatry [56]. Our data suggest that besides the role of selection in song divergence, these long time isolated lineages may have limited acoustic contact, probably resulting in a low level of repertoire syllable sharing. Despite the long isolation, individuals of the three lineages share some syllable types suggesting that their complex songs have conservative vocal elements retained through generations, and that non-shared syllables were gained or lost during the isolation and selection process.

### Conservation recommendations

The information generated by our study allows us to make the following conservation recommendation. The genetic differentiation among the three genetic groups revealed from the mtDNA analyses suggests that these groups have been genetically isolated for a long period of time. This genetic isolation and the lack of contemporary gene flow among these groups were also supported by the microsatellite data. In addition, the ancestral effective population size was larger than the size of either genetic group after splitting as revealed by IMa analysis. Because SMO, YUC and TUX are distinct genetic groups with no genetic connection among them, conservation plans should consider the sites within each of them as independent, and future management plans should focus on conserving the genetic diversity of the three genetic groups.

Conservation of the Tuxtlas region is particularly important due to the restricted distribution of the *excellens *group, the relatively small effective population size (one-third of that from SMO), and because the accelerated deforestation rates in the Tuxtlas region [57] is a threat to the unique genetic diversity of this group and possibly that of other endemic forms. Although populations within each of the genetic groups showed similar levels of genetic diversity, erosion of genetic variation is more likely to occur in the TUX group and thus conservation plans are of particular relevance for this geographic area. The mountains of this region, isolated from other mountain systems, arose from volcanic activity in the Oligocene, where oceanward orographic uplift produced one of the wettest climates in Mesoamerica [58]. This region contains one of the most diverse avifaunas in the northern Neotropics and, due to physical isolation it has been considered an important region of endemism [59]. Considering the alarming rate of deforestation in the Neotropics, the preservation of the montane and tropical forest in this region is urgently needed. To this end, the study of other co-distributed taxa to cover the phylogeography of Mesoamerica is warranted. This would shed light on any shared patterns of colonization and isolation in Mesoamerica and contribute to our understanding of the processes and responses of organisms to the environmental changes that drive population differentiation in this region.

## Conclusions

The genetic and phylogeographic analyses of this study, based on mtDNA and microsatellites, uncover the presence of three lineages of Mesoamerican wedge-tailed sabrewings that exhibit no contemporary gene flow. These correspond to the disjunct distribution of populations at the Sierra Madre Oriental, the Tuxtlas region, and the Yucatan Peninsula. Our results highlight the importance of the Isthmus of Tehuantepec in generating population divergence *c*. 1.4 million years ago without gene flow during the process of divergence, as well as more recent climate events during the Pleistocene in driving isolation and population divergence of wedge-tailed sabrewings with gene flow from the SMO to the Tuxtlas populations. Coalescent analyses of the evolution of phenotypic traits suggest that the fixation of song types has occurred faster than would be expected by genetic drift, suggesting that the action of selection is driving song evolution in wedge-tailed sabrewings. However, the role of drift in driving morphological divergence could not be rejected. Finally, considering the accelerated deforestation rates in the Neotropics the conservation of the montane and tropical forest in this region is needed, and special attention must be paid to small isolated areas with genetic uniqueness such as the Tuxtlas region.

## Methods

### Field procedures and feather sampling

For genetic analysis, based on the disjunct geographical distribution of the species complex [15,32], we sampled 160 individuals from 22 populations during the 2006, 2007 and 2008 *Campylopterus curvipennis *breeding seasons, covering the entire geographical range of the species complex: (*i*) 15 populations of *C. curvipennis curvipennis *(SMO), (*ii*) three populations of *C. curvipennis excellens *(TUX), and (*iii*) four populations of *C. curvipennis pampa *(YUC) (Table 1 Figure 2). The populations of the SMO group were sampled from three disjunct areas along the Sierra Madre Oriental: (*i*) three populations from southern Tamaulipas and northern San Luis Potosí (nSMO herein); (*ii*) three populations from southern San Luis Potosí and Hidalgo (cSMO herein); and (*iii*) nine populations from Puebla and central Veracruz (sSMO herein) (Table 1 Figure 2). Most of the *curvipennis *SMO populations are located in the understory of cloud forest or second-growth vegetation at a higher elevation (270-1,500 m above sea level) where the temperature is lower. The *excellens *TUX populations are located in wetter semideciduous tropical forests at a lower elevation (*c*. 260-1,000 m above sea level), and those of the *pampa *YUC populations are in drier deciduous tropical forests at a much lower elevation (*c*. 60-250 m above sea level) and higher temperatures (Table 1). Birds were captured in mist nets and the two outer tail rectrices were collected for subsequent genetic analysis before released. Research reported here was performed with the approval of the Mexican government (INE, SEMARNAT, SGPA/DGVS/02038/07), the UNAM Graduated Studies Committee (Doctorado en Ciencias Biomédicas), and followed the Guidelines for the Use of Wild Birds in Research proposed by the Ornithological Council. We also obtained tissue samples from three congeners to be used as outgroups in the phylogenetic analysis (*Campylopterus rufus*, *C. hemileucurus *and *C. largipennis*) (see Acknowledgments for tissue loans).

Three body measurements were obtained from mist-netted hummingbirds using a dial caliper to an accuracy of 0.1 mm and a wing ruler: exposed culmen (from the base of the bill to the tip of the upper mandible), wing chord (the distance from the carpal joint to the tip of the longest primary), and tail length (from the base of the uropygial gland to the tip of the longest rectrix). All measurements were taken by CG. Sex determination was carried out in the laboratory by means of a polymerase chain reaction (PCR) using primers 2550F [60] and MSZ1R [61]. González and Ornelas [19] have further detailed amplification conditions. Because males were not captured in the Tuxtlas region, we used data measurements taken by Lowery and Dalquest [62] from six males at the same collecting sites. To confirm the feasibility of these data and the possibility of combining them with ours, we compared the measurements in males and females from San Luis Potosí taken by these authors with data taken by us at the same locality. Also we compared the published measurements taken of females from those in the Tuxtlas region with our own data. As we did not detect significant differences between the two sources of measurements (data not shown) we used the published measurements of males from the Tuxtlas region in subsequent analyses.

Acoustic recordings were made for 64 males (557 recordings, ca. 9 recordings per bird) at 11 of the 22 sampled populations (Table 1). Song recordings were made with a Marantz PMD660 portable solid-state recorder and a Sennheiser MKH-70 directional microphone.

### Mitochondrial DNA sequencing and microsatellite genotyping

We extracted genomic DNA from one of the feather samples using chelex (5%, [63]), and that from tissue samples using the DNA easy blood and tissue kit (Qiagen, Inc.), following the recommended protocol. We used PCR to amplify two mitochondrial genes. The ATPase 6 and 8 coding region (875 bp), which includes two partially overlapping mitochondrial genes, was amplified using primers L8929 and H9947 [64]. The first domain of the control region (532 bp) was amplified using primers ARCO1F (5' AATTTTATGGTGTTTGTGTGTGAA 3') and ARCO1R (5' ACCCTAGCACAACTCGCACT 3') designed for this study from an *Archilochus colubris *D-loop and the complete tRNA-Phe gene sequence, and a partial 12 S ribosomal RNA gene sequence (GenBank accession EF520732.1). Amplification reactions (25 μl total volume) contained 0.72× buffer, 3.5 mM MgCl_2_, 0.14 mM of each dNTP, 0.2 μg/μl of BSA, 0.3 μM of each primer, 0.05 U *Taq *(Promega), and 2-5 μl of genomic DNA. PCR reactions were performed in a 2720 thermal cycler (Applied Biosystems) or in an Eppendorf Mastercycler thermocycler with the following temperature profile: initial denaturation at 94°C for 5 min; 35 cycles consisting of denaturation at 94°C for 1.5 min, annealing at 53-60°C for 1 min and an extension of 1.5 min at 72°C; and a final extension of 72°C for 7 min. PCR products were visualized on a 1% agarose gel stained with ethidium bromide, purified with the QIAquick PCR purification kit (Qiagen, Inc.) and sequenced using the Big Dye cycle terminator kit. Sequences were visualized in a 310 automated sequencer (Applied Biosystems) and by Macrogen Inc. ATPase genes were sequenced in both directions and the control region only in the reverse direction using primer ARCO1R except when ambiguities were present in the sequences. Sequences were edited using SEQUENCHER 4.8 demo (Gene Codes) and then manually aligned with SE-AL 2.0a11 [65]. All unique sequences used in this study have been deposited in the GenBank under accession nos. HQ380686-HQ380755.

Samples were genotyped at 10 polymorphic microsatellite loci designed specifically for *Campylopterus curvipennis *([66], GeneBank accession nos. GQ294539-GQ294550). PCR conditions and fragment sizing are fully described in Abdoullaye et al. [66].

### Analysis of mtDNA sequence data

We reconstructed intraspecific phylogenetic relationships among haplotypes using Bayesian inference in MRBAYES 3.12 [67]. Data were partitioned into two matrices, one corresponding to 875 bp of ATPase 6 and 8 and the other to 532 bp of control region. MODELTEST 3.7 [68] was run for each partition to choose the model of molecular evolution that best fit our sequence data. The best model for ATPase under the Bayesian information criterion (BIC) was HKY + G (base frequencies: A, 0.3588; C, 0.1664; G, 0.2373; T, 0.2376; gamma distribution shape parameter = 0.1825; transition/tranversion ratio = 6.22), and for the control region was TrN + I (base frequencies: A, 0.3688; C, 0.1564; G, 0.2470; T, 0.2278; proportion of invariable sites = 0.6869). We ran the analysis for 10 million generations using four chains (twice), sampling every 100th generation. We plotted the number of generations versus likelihood scores to check stationarity. Trees prior to stationarity were discarded as burn-in (25%), and the remaining were used to generate a consensus tree, later visualized in FIGTREE 1.2.3. We used three congeners as outgroups (*C. rufus, C. hemileucurus*, and *C. largipennis*) to root the tree. Alignment for the phylogenetic analyses is included as an additional file [Additional file 5].

In the absence of appropriate internal calibration points for many groups of birds, the 2% divergence-per-My clock calibration has been widely used. However, the degree of heterogeneity of molecular evolution rates across lineages and genetic loci could confound the accuracy of divergence time estimates, making the use of the 2% rule controversial [69-71]. In a more recent study, however, Weir and Schluter [72] cross-validated 90 avian clock calibrations (including one for hummingbirds) for cytochrome *b *obtained from fossil records and biogeographical events, demonstrating support for the 2% rule across taxonomic orders. Similar substitution rates have been described for other protein-coding mitochondrial DNA markers such as ATPase and ND2 [5,73-75]. Nevertheless, in order to minimize potential biases of this calibration, we calculated divergence times using two rates for the ATPase coding region data (0.02 and 0.05 substitutions/site/My) as suggested by Tarr and Fleischer [76], Milá et al. [77], and Barber and Klicka [14]. Uncertain homology arising from hypervariability or heteroplasmy in non-protein coding mitochondrial control region could confound measures of molecular clock estimates [77,78], so we did not estimate divergence time for that locus. Time to the most recent common ancestor (TMRCA) was estimated for the resulting clades using a Bayesian MCMC sampling approach with BEAST 1.4.2 [79]. TMRCA estimates were obtained using a relaxed clock model with log-normally distributed and uncorrelated rates of substitution between branches. No topological constraints were used allowing topological uncertainty to be taken into account. The model of evolution determined by MODELTEST under BIC (HKY+G for ATPase), was used for the analyses, and all other priors were set as default values in the program BEAUTI 1.4.7 that was used to create the XML files for input into BEAST. We ran BEAST for 10 million generations sampled every 1,000 generations, with the first 10 percent of the sampled points removed as burn-in. We obtained TMRCA estimates employing a range of 2 - 5% My to convert TMRCA to millions of years ago.

A statistical parsimony haplotype network was obtained using the program TCS 1.2.1 [80] using the 95% connection probability limit. Some ambiguities were detected in the networks (loops), and were broken according to three criteria (frequency, topology and geography), as proposed by Pfenninger and Posada [81].

We assessed the genetic variation within populations by calculating the haplotype diversity (*Hd*) and nucleotide diversity (*π*). To estimate population genetic structure we conducted an analysis of molecular variance (AMOVA). We used the model of molecular evolution of Tamura and Nei with a gamma shape parameter = 1.2757 for the combined data set, to estimate genetic distances. A total of 16,000 permutations were estimated to determine the AMOVA's significance. Two AMOVAs were run, grouping sampling sites based on the fragmented geographical range of wedge-tailed sabrewings (Table 1 Figure 2), and grouping sampling sites into subspecific groups (SMO, TUX, and YUC). Genetic differentiation among populations was determined with Φ statistics and pairwise *F_ST_*. Differences between populations were tested using 10,000 permutations among populations with Fisher's exact test. Genetic diversity estimates, AMOVAs and pairwise *F_ST _*were performed in ARLEQUIN 2.0 [82].

### Analysis of microsatellite data

Expected and observed heterozygosity and mean number of alleles per locus (allelic diversity) were calculated in GENEPOP 3.4 [83]. Allelic richness, a measure of the number of alleles per locus among populations independent of sample size, was calculated with the program FSTAT 2.9.3. Microsatellite genotypes were tested for linkage disequilibrium between pairs of loci and for departures from Hardy-Weinberg equilibrium (HWE) within populations and loci in GENEPOP. Bonferroni corrections were applied to correct for multiple simultaneous comparisons [84].

To investigate population genetic structure we calculated global and pairwise *R_ST _*[85] and pairwise *F_ST _*[86] in RSTCALC 2.2 [87] and ARLEQUIN, respectively. Differences between populations were tested using 10,000 permutations with a Fisher's exact test. To represent the relationships between localities we constructed a neighbor-joining tree using *R_ST _*pairwise values in PAUP 4.0 [88].

To examine geographic patterns of population genetic substructure, we performed Bayesian genetic clustering using STRUCTURE 2.2.3 [89], which is used to infer the most likely number of genetic clusters (*K*) through the multilocus genotypic data. We ran STRUCTURE without population information under the admixture model with correlated allele frequencies. Ten independent chains were run for each *K*, from *K *= 1 to *K *= 10. The length of the burn-in was 500,000 and the number of Markov chain Monte Carlo (MCMC) replications after the burn-in was 1,000,000. To determine an accurate number of clusters we calculated the statistic Δ*K *based on the rate of change in the log probability of data between successive *K *values following Evanno et al. [90].

Contemporary gene flow (M) among populations, and groups of populations, defined by mtDNA and microsatellite analyses, was estimated with a maximum likelihood coalescent approach using microsatellite data and MIGRATE v. 3 [91]. The first genealogy was started with a random tree, and initial theta and migration rate (M) parameters were obtained from *F_ST _*calculations. We ran 10 short (30,000 genealogies sampled) and three long chains (800,000 genealogies sampled), after discarding the first 20,000 genealogies as a burn-in.

Both mtDNA and microsatellite genetic analyses indicated three genetically diverged groups. We investigated whether these groups of populations had diverged in the face of gene flow by using IMa [92]. We performed two different tests, one involving populations from more recently diverged SMO and TUX populations, and the other involving populations east and west of the Isthmus of Tehuantepec. IMa is based on a Bayesian coalescent implementation of a model in which an ancestral population splits into two populations that may exchange genes in both directions at unequal rates during divergence. We performed preliminary simulations to specify the appropriate priors for each analysis, and ran three replicates with different random seed numbers with burn-in periods of 3,000,000 steps and 50,000,000 steps in chain following burn-in. We obtained estimates of migration rates between populations (*m_1_, m_2_*), and effective population sizes (*q*) from the ancestral population and after the split occurred between the two groups in both analyses. Estimates of migration rates were converted to the effective number of immigrants per generation by using estimates of theta. For estimates of effective population sizes of each genetic group we assumed a mutation rate per generation of 2.96 × 10^-3 ^[93].

### Morphological and acoustical variation

To examine differences in morphological variation between groups of the *C. curvipennis *complex, we performed a multivariate analysis of variance (MANOVA) with the three measurements taken as dependent variables, and lineages (see below) as fixed factors. One-way ANOVAs followed the MANOVA to determine lineage differences for each variable. To meet parametric analysis assumptions, variables were log transformed, but untransformed data are reported in figures. These analyses were performed for each sex separately due to size dimorphism using SUPERANOVA (Abacus Concepts, Inc).

**Figure 6:**
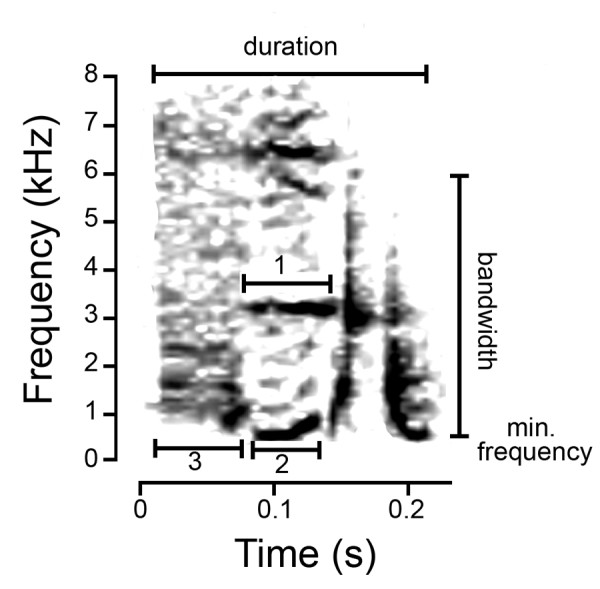
**Measurements taken from a common syllable emitted by every individual recorded**.

We generated spectrograms of recordings using RAVENPRO V. 1.4 http://www.birds.cornell.edu/raven with the parameters fully described in González and Ornelas [19]. We used syllabic units throughout the wedge-tailed sabrewing repertoire for population comparisons, and syllables were visually classified by structure on printed spectrograms by CG (see [19] for details). To assess acoustic structure among groups, we performed hierarchical cluster analyses based on a presence/absence matrix of syllable types for each of 64 individuals, where each entry consists of 0 or 1. We used the unweighted pair group method of arithmetic averages (UPGMA) and the Jaccard similarity measure to construct a dendrogram. As syllabic composition between individuals and localities is extremely variable [18,19], we took measurements from a common syllable emitted by all individuals from all localities to compare the temporal and spectral traits of song. The common syllable was randomly chosen from each individual and the duration (s), minimum frequency (kHz), bandwidth (kHz) and peak frequency (kHz) were measured. As this syllable has several elements (notes), we took the same measures for two of their elements (numbers 1 and 2, Figure 6), and the duration of another element (number 3, Figure 6). Principal component analysis (PCA) reduced the 13 variables taken from this syllable, and further one-way ANOVAs with uncorrelated factor scores were used to determine group differences for the first three PC components. PCAs and cluster analysis were performed in SPSS v.11.0.2 (SPSS, Inc.), and ANOVAs in STATVIEW (Abacus Concepts, Inc).

### Comparisons between morphological, acoustic, habitat-related, and genetic distances

We used climate and topographic variables across all 22 sampling localities to test for habitat selection acting on morphology and acoustic traits. We used these variables as a broad level approximation of habitat and ecological variation potentially related to availability of floral nectar resources (see also [41,45]). We extracted 19 climate variables from WorldClim, a global climate database with a spatial resolution of *c*. 1 km^2 ^[94], in combination with elevation, slope, and the compound topographic index (CTI; a function of upstream contributing area and slope that reflects tendency to pool water), all from the Hydro-1K dataset [95] for each geographic point where samples were obtained for genetic analysis. The WorldClim database provides high-resolution climate matrices that capture climate variability and also extreme or limiting climate factors.

We followed Ruegg et al. [45] to examine the relationships between morphology, acoustic, habitat-related (climate) and genetic distances. Namely, we performed a series of simple and partial Mantel tests using IBD [96], assessing the significance levels of association between matrices with 1,000 randomizations. Morphological and habitat-related (climate) distances between populations were calculated as a dissimilarity matrix with Euclidean distance scaling to values between 0 and 1. Acoustic distance matrices were built as follows. For song sharing data (presence/absence syllable types), we estimated the pairwise Jaccard similarity coefficient between individuals among sampling localities. This coefficient was calculated in ESTIMATES v. 8.0.0 [97] and values were subtracted from 1 to change it to a dissimilarity matrix. For the distance matrix of the common syllable measurements, we first performed a discriminant function analysis (DFA) using locality as the grouping variable, and the distance between localities was estimated as the Euclidean distance between group centroids for the first discriminant functions, and variables scaled to values between 0 and 1. Dissimilarity matrices and DFA were performed in SPSS 11.0.2. To test for the vicariance-drift model, we compared the matrices of morphology (for males and females separately) and acoustic distances (syllable sharing and acoustic measurements distance matrices) *versus *genetic distances (*F_ST _*for sequence data and *R_ST _*for microsatellites), controlling for geographic distance with partial Mantel tests. To test for the habitat-selection model, we compared matrices of morphology and acoustic distances (acoustic measurements) with habitat-related (climate and topography) distances.

### The role of drift and selection: a coalescent test

In order to test whether the degree of male phenotypic differentiation (vocal and morphological characters) was caused by drift or divergent selection, we performed an indirect coalescent-based simulation method developed by Masta and Maddison [30]. Because sexual selection by female choice reduces the amount of time needed for fixation of male phenotypic characters, without reducing the effective population size of neutral mitochondrial genes, Masta and Maddison's method tests statistically whether the rate of fixation of mitochondrial sequences differ from those of male phenotypic characters. This method assumes that nuclear genes code for the phenotypic character of interest (in our case, body size measurements and predisposition to learn certain song type), and asks whether the degree of fixation of phenotypes observed is likely to occur under the assumption of neutrality (see also [50,98]). We performed the HKA neutrality test for each set of sequences, to determine whether the genes were appropriate neutral markers.

In order to implement this method with our data, we performed two different analyses: in the first analysis for morphological characters we considered three representative populations (a higher number of populations increases the complexity of simulations; [30]) corresponding to the three genetic groups (Tux, Ciel and Nov). Because the use of continuous characters in phylogenetic studies can be problematic, we converted the morphological measurements into discrete characters states for this analysis. We constructed the character states of each of the continuous variables (wing chord, tail length and culmen) using the gap-weighting method in which the observed variation is divided into a number of segments of equal size (five character states), giving large weights to large differences in individual measures and small weights to small differences [99]. Continuous characters were coded as follows: wing chord, 0: 62-63 mm, 1: 64-66 mm, 2: 67-68 mm, 3: 69-71 mm, 4: 72-74 mm; tail length, 0: 47-49 mm, 1: 50-53 mm, 2: 54-55 mm, 3: 60-61 mm, 4: 63-64 mm; culmen, 0: 20-22 mm, 1: 23-24 mm, 2: 25-27 mm, 3: 27.5-28 mm, 4: 28.5-30 mm. The character states were fixed for the three populations considered: Nov, intermediate in size with short bill (wing chord: 0, 1; tail lengh: 0, 1; culmen: 0,1); Ciel, intermediate in size (wing chord: 0, 1; tail lengh: 1, 2; culmen: 2, 3); Tux, large in size (wing chord: 2, 3, 4; tail lengh: 3, 4; culmen: 3, 4). In the second analysis for vocal variation, we considered four populations in which vocal characters are fixed (Ciel, Ord, Tux and Nov); vocal traits were coded as presence/absence of introductory syllable type. In both analyses tests were performed alternatively by means of two assumptions of population divergence, dichotomous branching and simultaneous divergence.

We first calculated the *s *statistic of Slatkin and Maddison [100], a measure of incomplete lineage sorting among populations, for maximum parsimony trees inferred from the analysis of mtDNA sequences (results not shown); larger *s *values indicate greater levels of incomplete lineage sorting, suggesting a more recent population divergence, and smaller *s *values indicate lower levels of incomplete lineage sorting suggesting older population divergence. Then, we compared the observed *s *value against *s *values obtained from simulated trees to estimate time since population divergence. This comparison was performed with computer simulations generating 10,000 gene trees within a population tree by gene coalescence. We set *N_e _*= 500 in each population (as suggested by Masta and Maddison, [30]), and through simulations the upper 95% confidence limit was estimated for the number of generations since population divergence that would be expected to give the observed *s *value. Finally, simulations of nuclear gene trees were run to estimate the probability of complete fixation of morphological (*s *= 2) and vocal (*s *= 3) characters. Estimates of generations since population divergence were divided by four considering that nuclear genes have four times the population size of mitochondrial genes. Estimates of *s*, branch lengths and coalescent simulations were performed in MESQUITE v. 2.73 [101].

## Authors' contributions

CG and JFO conceived of and designed the study. CG carried out the fieldwork, generated the molecular data, and conducted all genetic and phenotypic analyses with input from CGR and JFO. CG and JFO wrote the manuscript with contributions from all authors, who read and approved the final manuscript.

## Supplementary Material

Additional file 1 **Pairwise comparisons between populations of *Campylopterus curvipennis***. Pairwise *F_ST _*values (above diagonal) of mtDNA and pairwise *R_ST _*values (below diagonal) of microsatellites between populations. Values statistically significant at *P *< 0.001 are indicated in bold. Click here for file

Additional file 2 **Neighbour-joining tree of sampled locations based on pairwise *R_ST_***. Analysis obtained from microsatellite data showing YUC group clustering together in a basal position, and sampling localities of SMO group clustering in a geographically unresolved clade. Sampled locations with only one individual were excluded from the analysis. Click here for file

Additional file 3 **Results of the coalescent-based simulation on phenotypic traits**. Estimates of *s *of Slatkin and Maddison from 10,000 coalescent simulated gene trees to test the probability of complete sorting of vocal characters (A and B) and morphological characters (C and D) in wedge-tailed sabrewing populations. Regarding vocal characters, there is a low probability of fixation in nuclear genes under neutrality regardless of the assumption of dichotomous branching (A) or the simultaneous model of divergence (B). In contrast, the probabilities that nuclear genes would be fixed under neutrality are high for morphological characters, regardless of the assumption of dichotomous branching (C) or the simultaneous model of divergence (D). This suggests that divergent selection has caused the pattern of vocal variation among populations, but the null hypothesis that morphological divergence resulted from drift was not rejected. Arrows indicate the expected value of *s *in a completely sorted tree. Click here for file

Additional file 4 **Geographic distribution of *Campylopterus curvipennis *species complex**. Distribution of *Campylopterus curvipennis *species complex based on museum (MZFC) and bibliographic records [32,59], showing the disjunct distribution of three subspecies. Blue = *C. c. curvipennis*, yellow = *C. c. excellens*, red = *C. c. pampa*. Click here for file

Additional file 5 **Alignment of ATPase 6-8 and control region mtDNA sequences**. Alignment of the concatenated ATPase 6-8 (1-875 bp) and control region (876-1407 bp) mtDNA sequences for 160 individuals of *Campylopterus curvipennis *and three outgroups used in the phylogenetic analyses. Click here for file
