# Supplementary material for: Selection and geographic isolation influence hummingbird speciation: genetic, acoustic and morphological divergence in the wedge-tailed sabrewing (Campylopterus curvipennis)
Source: BMC Evol Biol. 2011 Feb 8;11:38. doi: 10.1186/1471-2148-11-38 (PMC3045325; doi:10.1186/1471-2148-11-38)
Supplement: Additional file 1 — Pairwise comparisons between populations of Campylopterus curvipennis. Pairwise FST values (above diagonal) of mtDNA and pairwise RST values (below diagonal) of microsatellites between populations. Values statistically significant at P < 0.001 are indicated in bold. [file 1471-2148-11-38-S1.DOC]

**Additional file 1 Supplemental Table S1. Pairwise comparisons between populations of *Campylopterus curvipennis*.**

|  | SMO | | | | | | |  | |  |  |  |  |  | |  |  | |  |  |  |  | |  | TUX |  | YUC | | |  |
| --- | --- | --- | --- | --- | --- | --- | --- | --- | --- | --- | --- | --- | --- | --- | --- | --- | --- | --- | --- | --- | --- | --- | --- | --- | --- | --- | --- | --- | --- | --- |
|  | GF | Ciel | Nar | Aqm | Xil | Ord | Mac | | UG | | | Coap | | | Risc | | | Cuet | | | Xico | Ama | Clav | | Tux | Bec | | Gar | Nov | |
| GF | – | 0.141 | 0.199 | 0 | -0.045 | 0.029 | 0.079 | | 0.007 | | | -0.043 | | | 0.113 | | | 0.019 | | | 0.308 | 0.079 | 0.175 | | **0.899** | 0.982 | | 0.966 | **0.934** | |
| Ciel | -0.025 | – | 0.249 | 0.1159 | 0.169 | 0.111 | -0.138 | | 0.159 | | | 0.097 | | | 0.077 | | | 0.003 | | | 0.185 | 0.036 | 0.155 | | **0.746** | **0.940** | | **0.938** | **0.932** | |
| Nar | 0.031 | 0.083 | – | 0.2010 | 0.169 | 0.030 | 0.105 | | 0.234 | | | 0.052 | | | 0.025 | | | 0.105 | | | 0.048 | -0.065 | -0.035 | | **0.795** | 0.929 | | 0.926 | **0.921** | |
| Aqm | 0.016 | 0.053 | 0.043 | – | -0.092 | 0.030 | 0.079 | | 0.065 | | | -0.040 | | | 0.110 | | | -0.002 | | | 0.308 | 0.003 | 0.155 | | **0.899** | 0.982 | | **0.966** | **0.934** | |
| Xil | 0.003 | -0.017 | 0.024 | -.0310 | – | 0.029 | 0.051 | | 0.026 | | | -0.041 | | | -0.097 | | | 0.043 | | | 0.282 | 0.011 | 0.155 | | **0.845** | 0.960 | | 0.954 | **0.935** | |
| Ord | 0.022 | 0.021 | 0.079 | .0393 | 0.001 | – | -0.037 | | 0.095 | | | -0.027 | | | -0.063 | | | 0.009 | | | 0.137 | -0.087 | 0.042 | | **0.749** | 0.941 | | **0.939** | **0.933** | |
| Mac | 0.016 | 0.027 | 0.069 | .0778 | 0.004 | 0.068 | – | | 0.057 | | | -0.094 | | | -0.114 | | | -0.135 | | | 0.026 | -0.139 | 0.034 | | 0.827 | 0.941 | | **0.933** | **0.924** | |
| UG | -0.053 | 0.010 | 0.072 | .0072 | 0.008 | 0.008 | 0.078 | | – | | | 0.047 | | | -0.042 | | | 0.074 | | | 0.332 | -0.019 | 0.187 | | **0.823** | 0.958 | | **0.954** | **0.939** | |
| Coap | 0.013 | 0.012 | -0.012 | -.0204 | -0.015 | 0.035 | 0.005 | | 0.032 | | | – | | | -0.176 | | | 0.003 | | | 0.111 | -0.137 | 0.058 | | **0.757** | 0.927 | | 0.924 | **0.923** | |
| Risc | 0.047 | 0.089 | 0.169 | .0350 | 0.046 | 0.165 | 0.060 | | 0.117 | | | 0.056 | | | – | | | -0.063 | | | 0.133 | -0.132 | 0.065 | | 0.887 | 0.969 | | 0.947 | 0.925 | |
| Cuet | -0.023 | 0.002 | 0.045 | .0340 | -0.006 | 0.009 | 0.033 | | 0.005 | | | -0.002 | | | 0.125 | | | – | | | 0.119 | -0.065 | 0.057 | | **0.702** | **0.931** | | **0.930** | **0.928** | |
| Xico | -0.002 | 0.058 | 0.068 | .1129 | 0.061 | 0.031 | 0.139 | | 0.038 | | | 0.043 | | | 0.252 | | | 0.007 | | | – | 0.025 | 0.015 | | **0.793** | 0.916 | | 0.914 | **0.918** | |
| Ama | 0.069 | 0.151 | -0.022 | .0342 | 0.091 | 0.135 | 0.155 | | 0.102 | | | -0.055 | | | 0.279 | | | 0.087 | | | 0.094 | – | -0.067 | | 0.798 | 0.930 | | **0.924** | **0.922** | |
| Clav | -0.037 | **0.**072 | -0.005 | .0102 | 0.040 | 0.059 | 0.099 | | 0.006 | | | -0.001 | | | 0.207 | | | 0.025 | | | 0.010 | -0.048 | – | | **0.789** | 0.935 | | 0.931 | **0.924** | |
| Tux | **0.212** | **0.238** | **0.239** | **.2367** | **0.194** | **0.223** | 0.094 | | **0.203** | | | 0.139 | | | **0.301** | | | **0.222** | | | **0.312** | **0.295** | **0.248** | | – | 0.979 | | 0.974 | **0.950** | |
| Bec | 0.209 | **0.351** | 0.215 | **.**3000 | 0.327 | **0.322** | 0.302 | | 0.246 | | | 0.153 | | | 0.525 | | | **0.302** | | | 0.304 | 0.113 | 0.128 | | **0.318** | – | | -0.202 | 0.015 | |
| Gar | 0.140 | **0.226** | 0.134 | .2099 | 0.217 | **0.208** | 0.215 | | 0.171 | | | 0.089 | | | 0.421 | | | **0.196** | | | 0.180 | 0.046 | 0.070 | | **0.316** | -0.158 | | – | -0.128 | |
| Nov | **0**.**201** | **0.296** | **0.172** | **.2611** | **0.277** | **0.289** | **0.237** | | **0.253** | | | 0.124 | | | **0.428** | | | **0.266** | | | **0.269** | 0.114 | **0.150** | | **0.360** | -0.020 | | -0.068 | – | |

Pairwise *F_ST_* values (above diagonal) of mtDNA and pairwise *R_ST_* values (below diagonal) of microsatellites between populations.

Values statistically significant at *P* < 0.001 are indicated in bold.
