# Supplementary figures and images for: Selection and geographic isolation influence hummingbird speciation: genetic, acoustic and morphological divergence in the wedge-tailed sabrewing (Campylopterus curvipennis)
Source: BMC Evol Biol. 2011 Feb 8;11:38. doi: 10.1186/1471-2148-11-38 (PMC3045325; doi:10.1186/1471-2148-11-38)

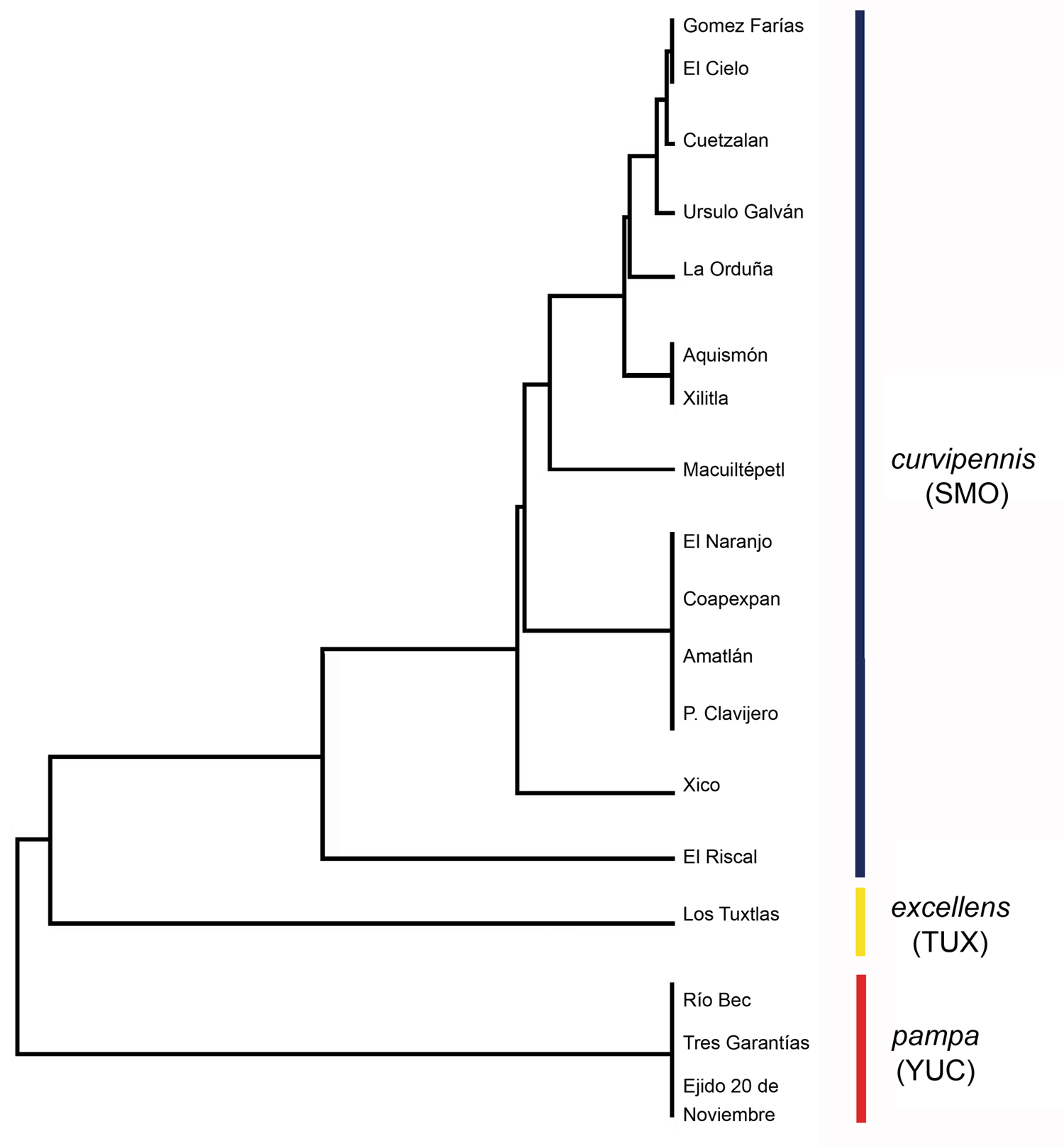

Supplement: Additional file 2 — Neighbour-joining tree of sampled locations based on pairwise RST. Analysis obtained from microsatellite data showing YUC group clustering together in a basal position, and sampling localities of SMO group clustering in a geographically unresolved clade. Sampled locations with only one individual were excluded from the analysis. [file 1471-2148-11-38-S2.TIFF]

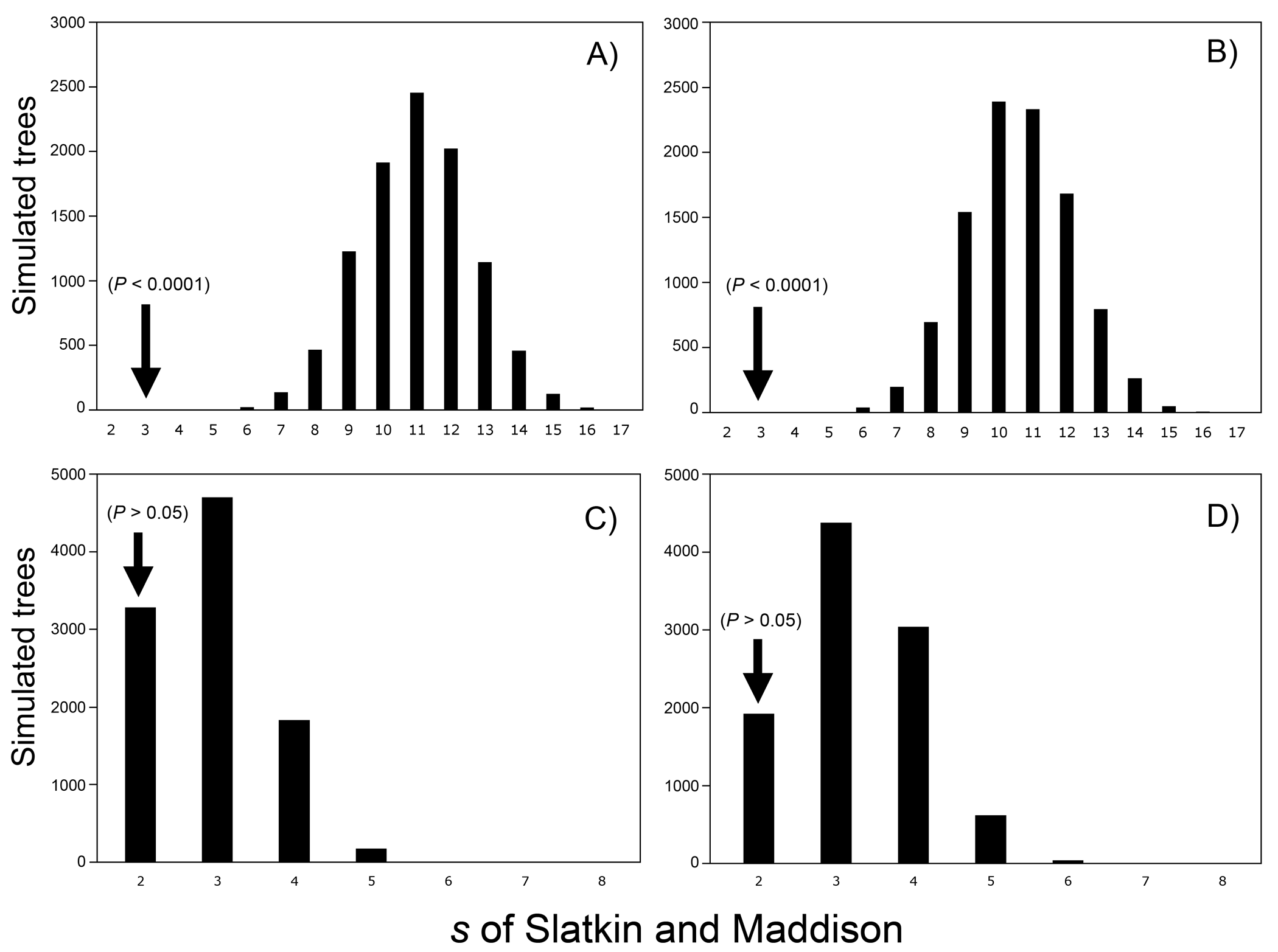

Supplement: Additional file 3 — Results of the coalescent-based simulation on phenotypic traits. Estimates of s of Slatkin and Maddison from 10,000 coalescent simulated gene trees to test the probability of complete sorting of vocal characters (A and B) and morphological characters (C and D) in wedge-tailed sabrewing populations. Regarding vocal characters, there is a low probability of fixation in nuclear genes under neutrality regardless of the assumption of dichotomous branching (A) or the simultaneous model of divergence (B). In contrast, the probabilities that nuclear genes would be fixed under neutrality are high for morphological characters, regardless of the assumption of dichotomous branching (C) or the simultaneous model of divergence (D). This suggests that divergent selection has caused the pattern of vocal variation among populations, but the null hypothesis that morphological divergence resulted from drift was not rejected. Arrows indicate the expected value of s in a completely sorted tree. [file 1471-2148-11-38-S3.TIFF]

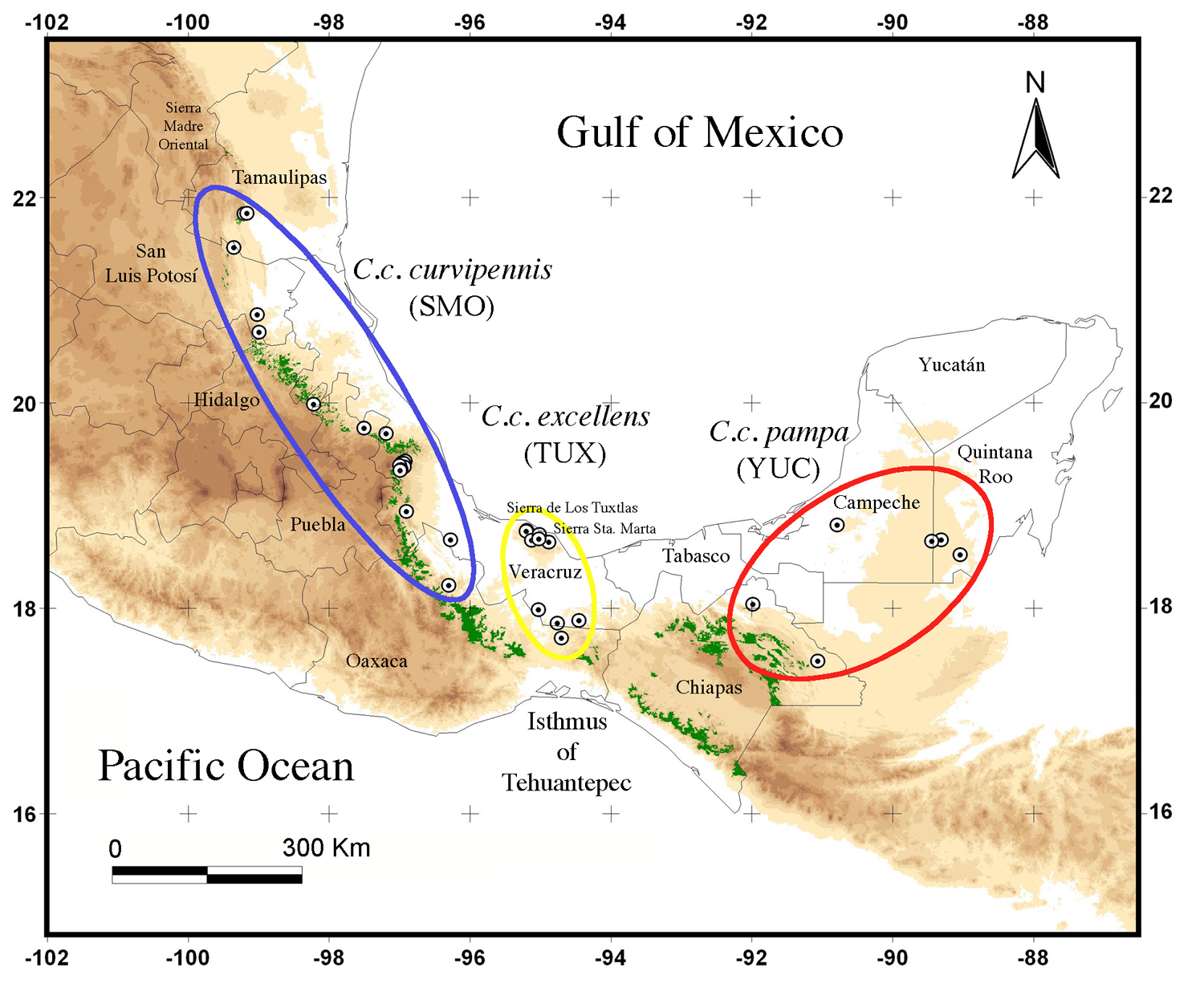

Supplement: Additional file 4 — Geographic distribution of Campylopterus curvipennis species complex. Distribution of Campylopterus curvipennis species complex based on museum (MZFC) and bibliographic records [32,59], showing the disjunct distribution of three subspecies. Blue = C. c. curvipennis, yellow = C. c. excellens, red = C. c. pampa. [file 1471-2148-11-38-S4.TIFF]
