# Supplementary material for: Selection and geographic isolation influence hummingbird speciation: genetic, acoustic and morphological divergence in the wedge-tailed sabrewing (Campylopterus curvipennis)
Source: BMC Evol Biol. 2011 Feb 8;11:38. doi: 10.1186/1471-2148-11-38 (PMC3045325; doi:10.1186/1471-2148-11-38)
Supplement: Additional file 5 — Alignment of ATPase 6-8 and control region mtDNA sequences. Alignment of the concatenated ATPase 6-8 (1-875 bp) and control region (876-1407 bp) mtDNA sequences for 160 individuals of Campylopterus curvipennis and three outgroups used in the phylogenetic analyses. [file 1471-2148-11-38-S5.DOC]

1_Campylopterus_curvipennis_ord.03-05

GGATTATGGCTACTGCTACTTCTAGGATGGTTAGTAGGAGTAGTACGCAGGCGGTTAGAGCAGAGATTGCGGGTATGATG

GAGAATAGGACTGCGGTGGCTGTTGAGATGAGTTGGATTAGTAGGTGGCCTGCTGTTAGGTTAGCTGTGAGGCGGACTCC

TAGGGCTAGAGGGCGGATCAGGAGGCTGGTAGTTTCAATTATGATTAAGGCTGGGATTAGTGGAGTTGGGGTTCCTTCGG

GTAGGAGGTGGCCAAGTGAAGCGGATGGTTGGTTTCGGAGGCCTGTGAGTAGAGTGGCTAGTCATAGTGGGAAGGCTAGT

GCTAGGTTTATAGATAATTGTGTGGTAGGTGTGAATGTGTAGGGGAGTAGGCCTAGTAGGTTGATTGAGAGAAGGAAAAT

TATTAGTGATGACAGGATAAGGGCTCATTTGTGGCCTTTTTTGTTCAATGGGATTATCAGTTGTTTGGTGATTATATAGA

TGAATCACGATTGTAGGGTGGATGTTCGGTTGGTGATTCATCGGTTGTGGGGGGAGGGGAGTAGTAGGGCTGGGAATAAT

AGTGAGATGAGAATTAGGGGGATTCCTAGTAGGTGGGGGCTTATAAATTGGTCGAAGAAGCTTAGGTTCATGGTCAGGGT

CAGGATGAGGGTTTTGTGTTTGTTAGGGTTTTTTCTAGGGGGGAGTTAGTGGGCATGAATGAAAGGAGTTTGGGTTGGAT

GATTAGTGAAAAGGTCAGTCATGAAATAGTTATAATGAATAGTCATGGGTTTGGATTTAGTTGGGGCATATCATTAGGGA

GGGTTGGTAATCCTCTTTCTCTAGCTTAAAAGGCTAGTGCTGTTGCATAGCTTCCTAGTGGTTAAGATGATAGGAAAATT

AACATGATAAACATGATGATGGATGATGATTATTTTTTAATGTAATCTCGGTGTTGAATCTGGGTTAAAGTTAGATGATA

AAATGTGTGATGAAAATATCATGGATAGTATGGAAATTATAACTTAGAACTCCAGTGGTGTTGGTTAGAAAGTTAGAGGA

AGTGAAAAACTTGTAAAATATGTCCGGCAACCATAAATCAAAAAGCAACATAGCTTAGTAAGTATGTGAAAATACCATAA

CCAAATGCAAGACAAAGTGCATCAGTGTCAAAATGAGACTGAATTACACATGAAACCGTCTCATTCAGCAACTCTTGAAG

GAGAGAAAAGGCTCGGGAGCCACTGATGCCCAGGTCAAGAGATTGTGTGCTCCGCGTTAGCCACTGGAAGGGCCTATTGA

GATTACCCAAAAAAAAAGA-GAACCAACC-GCCTAAAAGAAGGGAACCATGCCGCGATTAAGAAAGGTATGAACTATAAA

GTACCAACCAAATGCCTTTGAAAGGAAATCCACAGGGGAGGAGTCTA

2_Campylopterus_curvipennis_ord.23-05

GGATTATGGCTACTGCTACTTCTAGGATGGTTAGTAGGAGTAGTACGCAGGCGGTTAGAGCAGAGATTGCGGGTATGATG

GAGAATAGGACTGCGGTGGCTGTTGAGATGAGTTGGATTAGTAGGTGGCCTGCTGTTAGGTTAGCTGTGAGGCGGACTCC

TAGGGCTAGAGGGCGGATCAGGAGGCTGGTAGTTTCAATTATGATTAAGGCTGGGATTAGTGGAGTTGGGGTTCCTTCGG

GTAGGAGGTGGCCAAGTGAAGCGGATGGTTGGTTTCGGAGGCCTGTGAGTAGAGTGGCTAGTCATAGTGGGAAGGCTAGT

GCTAGGTTTATAGATAATTGTGTGGTAGGTGTGAATGTGTAGGGGAGTAGGCCTAGTAGGTTGATTGAGAGAAGGAAAAT

TATTAGTGATGACAGGATAAGGGCTCATTTGTGGCCTTTTTTGTTCAATGGGATTATCAGTTGTTTGGTGATTATATAGA

TGAATCACGATTGTAGGGTGGATGTTCGGTTGGTGATTCATCGGTTGTGGGGGGAGGGGAGTAGTAGGGCTGGGAATAAT

AGTGAGATGAGAATTAGGGGGATTCCTAGTAGGTGGGGGCTTATAAATTGGTCGAAGAAGCTTAGGTTCATGGTCAGGGT

CAGGATGAGGGTTTTGTGTTTGTTAGGGTTTTTTCTAGGGGGGAGTTAGTGGGCATGAATGAAAGGAGTTTGGGTTGGAT

GATTAGTGAAAAGGTCAGTCATGAAATAGTTATAATGAATAGTCATGGGTTTGGATTTAGTTGGGGCATATCATTAGGGA

GGGTTGGTAATCCTCTTTCTCTAGCTTAAAAGGCTAGTGCTGTTGCATAGCTTCCTAGTGGTTAAGATGATAGGAAAATT

AACATGATAAACATGATGATGGATGATGATTATTTTTTAATGTAATCTCGGTGTTGAATCTGGGTTAAAGTTAGATGATA

AAATGTGTGATGAAAATATCATGGATAGTATGGAAATTATAACTTAGAACTCCAGTGGTGTTGGTTAGAAAGTTAGAGGA

AGTGAAAAACTTGTAAAATATGTCCGGCAACCATAAATCAAAAAGCAACATAGCTTAGTAAGTATGTGAAAATACCATAA

CCAAATGCAAGACAAAGTGCATCAGTGTCAAAATGAGACTGAATTACACATGAAACCGTCTCATTCAGCAACTCTTGAAG

GAGAGAAAAGGCTCGGGAGCCACTGATGCCCAGGTCAAGAGATTGTGTGCTCCGCGTTAGCCACTGGAAGGGCCTATTGA

GATTACCCAAAAAAAAAGA-GAACCAACC-GCCTAAAAGAAGGGAACCATGCCGCGATTAAGAAAGGTATGAACTATAAA

GTACCAACCAAATGCCTTTGAAAGGAAATCCACAGGGGAGGAGTCTA

3_Campylopterus_curvipennis_ord.38-05

GGATTATGGCTACTGCTACTTCTAGGATGGTTAGTAGGAGTAGTACGCAGGCGGTTAGAGCAGAGATTGCGGGTATGATG

GAGAATAGGACTGCGGTGGCTGTTGAGATGAGTTGGATTAGTAGGTGGCCTGCTGTTAGGTTAGCTGTGAGGCGGACTCC

TAGGGCTAGAGGGCGGATCAGGAGGCTGGTAGTTTCAATTATGATTAAGGCTGGGATTAGTGGAGTTGGGGTTCCTTCGG

GTAGGAGGTGGCCAAGTGAAGCGGATGGTTGGTTTCGGAGGCCTGTGAGTAGAGTGGCTAGTCATAGTGGGAAGGCTAGT

GCTAGGTTTATAGATAATTGTGTGGTAGGTGTGAATGTGTAGGGGAGTAGGCCTAGTAGGTTGATTGAGAGAAGGAAAAT

TATTAGTGATGACAGGATAAGGGCTCATTTGTGGCCTTTTTTGTTCAATGGGATTATCAGTTGTTTGGTGATTATATAGA

TGAATCACGATTGTAGGGTGGATGTTCGGTTGGTGATTCATCGGTTGTGGGGGGAGGGGAGTAGTAGGGCTGGGAATAAT

AGTGAGATGAGAATTAGGGGGATTCCTAGTAGGTGGGGGCTTATAAATTGGTCGAAGAAGCTTAGGTTCATGGTCAGGGT

CAGGATGAGGGTTTTGTGTTTGTTAGGGTTTTTTCTAGGGGGGAGTTAGTGGGCATGAATGAAAGGAGTTTGGGTTGGAT

GATTAGTGAAAAGGTCAGTCATGAAATAGTTATAATGAATAGTCATGGGTTTGGATTTAGTTGGGGCATATCATTAGGGA

GGGTTGGTAATCCTCTTTCTCTAGCTTAAAAGGCTAGTGCTGTTGCATAGCTTCCTAGTGGTTAAGATGATAGGAAAATT

AACATGATAAACATGATGATGGATGATGATTATTTTTTAATGTAATCTCGGTGTTGAATCTGAGTTAAAGTTAGATGATA

AAATGTGTGATGAAAATATCATGGATAGTATGGAAATTATAACTTAGAACTCCAGTGGTGTTGGTTAGAAAGTTAGAGGA

AGTGAAAAACTTGTAAAATATGTCCGGCAACCATAAATCAAAAAGCAACATAGCTTAGTAAGTATGTGAAAATACCATAA

CCAAATGCAAGACAAAGTGCATCAGTGTCAAAATGAGACTGAATTACACATGAAACCGTCTCATTCAGCAACTCTTGAAG

GAGAGAAAAGGCTCGGGAGCCACTGATGCTCAGGTCAAGAGATTGTGTGCTCCGCGTTAGCCACTGGAAGGGCCTATTGA

GATTACCCAAAAAAAAAGA-GAACCAACC-GCCTAAAAGAAGGGAACCATGCCGCGATTAAGAAAGGTATGAACTATAAA

GTACCAACCAAATGCCTTTGAAAGGAAATCCACAGGGGAGGAGTCTA

4_Campylopterus_curvipennis_ord.42-05

GGATTATGGCTACTGCTACTTCTAGGATGGTTAGTAGGAGTAGTACGCAGGCGGTTAGAGCAGAGATTGCGGGTATGATG

GAGAATAGGACTGCGGTGGCTGTTGAGATGAGTTGGATTAGTAGGTGGCCTGCTGTTAGGTTAGCTGTGAGGCGGACTCC

TAGGGCTAGAGGGCGGATCAGGAGGCTGGTAGTTTCAATTATGATTAAGGCTGGGATTAGTGGAGTTGGGGTTCCTTCGG

GTAGGAGGTGGCCAAGTGAAGCGGATGGTTGGTTTCGGAGGCCTGTGAGTAGAGTGGCTAGTCATAGTGGGAAGGCTAGT

GCTAGGTTTATAGATAATTGTGTGGTAGGTGTGAATGTGTAGGGGAGTAGGCCTAGTAGGTTGATTGAGAGAAGGAAAAT

TATTAGTGATGACAGGATAAGGGCTCATTTGTGGCCTTTTTTGTTCAATGGGATTATCAGTTGTTTGGTGATTATATAGA

TGAATCACGATTGTAGGGTGGATGTTCGGTTGGTGATTCATCGGTTGTGGGGGGAGGGGAGTAGTAGGGCTGGGAATAAT

AGTGAGATGAGAATTAGGGGGATTCCTAGTAGGTGGGGGCTTATAAATTGGTCGAAGAAGCTTAGGTTCATGGTCAGGGT

CAGGATGAGGGTTTTGTGTTTGTTAGGGTTTTTTCTAGGGGGGAGTTAGTGGGCATGAATGAAAGGAGTTTGGGTTGGAT

GATTAGTGAAAAGGTCAGTCATGAAATAGTTATAATGAATAGTCATGGGTTTGGATTTAGTTGGGGCATATCATTAGGGA

GGGTTGGTAATCCTCTTTCTCTAGCTTAAAAGGCTAGTGCTGTTGCATAGCTTCCTAGTGGTTAAGATGATAGGAAAATT

AACATGATAAACATGATGATGGATGATGATTATTTTTTAATGTAATCTCGGTGTTGAATCTGGGTTAAAGTTAGATGATA

AAATGTGTGATGAAAATATCATGGATAGTATGGAAATTATAACTTAGAACTCCAGTGGTGTTGGTTAGAAAGTTAGAGGA

AGTGAAAAACTTGTAAAATATGTCCGGCAACCATAAATCAAAAAGCAACATAGCTTAGTAAGTATGTGAAAATACCATAA

CCAAATGCAAGACAAAGTGCATCAGTGTCAAAATGAGACTGAATTACACATGAAACCGTCTCATTCAGCAACTCTTGAAG

GAGAGAAAAGGCTCGGGAGCCACTGATGCCCAGGTCAAGAGATTGTGTGCTCCGCGTTAGCCACTGGAAGGGCCTATTGA

GATTACCCAAAAAAAAAGA-GAACCAACC-GCCTAAAAGAAGGGAACCATGCCGCGATTAAGAAAGGTATGAACTATAAA

GTACCAACCAAATGCCTTTGAAAGGAAATCCACAGGGGAGGAGTCTA

5_Campylopterus_curvipennis_ord.44-05

GGATTATGGCTACTGCTACTTCTAGGATGGTTAGTAGGAGTAGTACGCAGGCGGTTAGAGCAGAGATTGCGGGTATGATG

GAGAATAGGACTGCGGTGGCTGTTGAGATGAGTTGGATTAGTAGGTGGCCTGCTGTTAGGTTAGCTGTGAGGCGGACTCC

TAGGGCTAGAGGGCGGATCAGGAGGCTGGTAGTTTCAATTATGATTAAGGCTGGGATTAGTGGAGTTGGGGTTCCTTCGG

GTAGGAGGTGGCCAAGTGAAGCGGATGGTTGGTTTCGGAGGCCTGTGAGTAGAGTGGCTAGTCATAGTGGGAAGGCTAGT

GCTAGGTTTATAGATAATTGTGTGGTAGGTGTGAATGTGTAGGGGAGTAGGCCTAGTAGGTTGATTGAGAGAAGGAAAAT

TATTAGTGATGACAGGATAAGGGCTCATTTGTGGCCTTTTTTGTTCAATGGGATTATCAGTTGTTTGGTGATTATATAGA

TGAATCACGATTGTAGGGTGGATGTTCGGTTGGTGATTCATCGGTTGTGGGGGGAGGGGAGTAGTAGGGCTGGGAATAAT

AGTGAGATGAGAATTAGGGGGATTCCTAGTAGGTGGGGGCTTATAAATTGGTCGAAGAAGCTTAGGTTCATGGTCAGGGT

CAGGATGAGGGTTTTGTGTTTGTTAGGGTTTTTTCTAGGGGGGAGTTAGTGGGCATGAATGAAAGGAGTTTGGGTTGGAT

GATTAGTGAAAAGGTCAGTCATGAAATAGTTATAATGAATAGTCATGGGTTTGGATTTAGTTGGGGCATATCATTAGGGA

GGGTTGGTAATCCTCTTTCTCTAGCTTAAAAGGCTAGTGCTGTTGCATAGCTTCCTAGTGGTTAAGATGATAGGAAAATT

AACATGATAAACATGATGATGGATGATGATTATTTTTTAATGTAATCTCGGTGTTGAATCTGGGTTAAAGTTAGATGATA

AAATGTGTGATGAAAATATCATGGATAGTATGGAAATTATAACTTAGAACTCCAGTGGTGTTGGTTAGAAAGTTAGAGGA

AGTGAAAAACTTGTAAAATATGTCCGGCAACCATAAATCAAAAAGCAACATAGCTTAGTAAGTATGTGAAAATACCATAA

CCAAATGCAAGACAAAGTGCATCAGTGTCAAAATGAGACTGAATTACACATGAAACCGTCTCATTCAGCAACTCTTGAAG

GAGAGAAAAGGCTCGGGAGCCACTGATGCCCAGGTCAAGAGATTGTGTGCTCCGCGTTAGCCACTGGAAGGGCCTATTGA

GATTACCCAAAAAAAAAGA-GAACCAACC-GCCTAAAAGAAGGGAACCATGCCGCGATTAAGAAAGGTATGAACTATAAA

GTACCAACCAAATGCCTTTGAAAGGAAATCCACAGGGGAGGAGTCTA

6_Campylopterus_curvipennis_ord.50-05

GGATTATGGCTACTGCTACTTCTAGGATGGTTAGTAGGAGTAGTACGCAGGCGGTTAGAGCAGAGATTGCGGGTATGATG

GAGAATAGGACTGCGGTGGCTGTTGAGATGAGTTGGATTAGTAGGTGGCCTGCTGTTAGGTTAGCTGTGAGGCGGACTCC

TAGGGCTAGAGGGCGGATCAGGAGGCTGGTAGTTTCAATTATGATTAAGGCTGGGATTAGTGGAGTTGGGGTTCCTTCGG

GTAGGAGGTGGCCAAGTGAAGCGGATGGTTGGTTTCGGAGGCCTGTGAGTAGAGTGGCTAGTCATAGTGGGAAGGCTAGT

GCTAGGTTTATAGATAATTGTGTGGTAGGTGTGAATGTGTAGGGGAGTAGGCCTAGTAGGTTGATTGAGAGAAGGAAAAT

TATTAGTGATGACAGGATAAGGGCTCATTTGTGGCCTTTTTTGTTCAATGGGATTATCAGTTGTTTGGTGATTATATAGA

TGAATCACGATTGTAGGGTGGATGTTCGGTTGGTGATTCATCGGTTGTGGGGGGAGGGGAGTAGTAGGGCTGGGAATAAT

AGTGAGATGAGAATTAGGGGGATTCCTAGTAGGTGGGGGCTTATAAATTGGTCGAAGAAGCTTAGGTTCATGGTCAGGGT

CAGGATGAGGGTTTTGTGTTTGTTAGGGTTTTTTCTAGGGGGGAGTTAGTGGGCATGAATGAAAGGAGTTTGGGTTGGAT

GATTAGTGAAAAGGTCAGTCATGAAATAGTTATAATGAATAGTCATGGGTTTGGATTTAGTTGGGGCATATCATTAGGGA

GGGTTGGTAATCCTCTTTCTCTAGCTTAAAAGGCTAGTGCTGTTGCATAGCTTCCTAGTGGTTAAGATGATAGGAAAATT

AACATGATAAACATGATGATGGATGATGATTATTTTTTAATGTAATCTCGGTGTTGAATCTGGGTTAAAGTTAGATGATA

AAATGTGTGATGAAAATATCATGGATAGTATGGAAATTATAACTTAGAACTCCAGTGGTGTTGGTTAGAAAGTTAGAGGA

AGTGAAAAACTTGTAAAATATGTCCGGCAACCATAAATCAAAAAGCAACATAGCTTAGTAAGTATGTGAAAATACCATAA

CCAAATGCAAGACAAAGTGCATCAGTGTCAAAATGAGACTGAATTACACATGAAACCGTCTCATTCAGCAACTCTTGAAG

GAGAGAAAAGGCTCGGGAGCCACTGATGCCCAGGTCAAGAGATTGTGTGCTCCGCGTTAGCCACTGGAAGGGCCTATTGA

GATTACCCAAAAAAAAAGA-GAACCAACC-GCCTAAAAGAAGGGAACCATGCCGCGATTAAGAAAGGTATGAACTATAAA

GTACCAACCAAATGCCTTTGAAAGGAAATCCACAGGGGAGGAGTCTA

7_Campylopterus_curvipennis_ord.01-06

GGATTATGGCTACTGCTACTTCTAGGATGGTTAGTAGGAGTAGTACGCAGGCGGTTAGAGCAGAGATTGCGGGTATGATG

GAGAATAGGACTGCGGTGGCTGTTGAGATGAGTTGGATTAGTAGGTGGCCTGCTGTTAGGTTAGCTGTGAGGCGGACTCC

TAGGGCTAGAGGGCGGATCAGGAGGCTGGTAGTTTCAATTATGATTAAGGCTGGGATTAGTGGAGTTGGGGTTCCTTCGG

GTAGGAGGTGGCCAAGTGAAGCGGATGGTTGGTTTCGGAGGCCTGTGAGTAGAGTGGCTAGTCATAGTGGGAAGGCTAGT

GCTAGGTTTATAGATAATTGTGTGGTAGGTGTGAATGTGTAGGGGAGTAGGCCTAGTAGGTTGATTGAGAGAAGGAAAAT

TATTAGTGATGACAGGATAAGGGCTCATTTGTGGCCTTTTTTGTTCAATGGGATTATCAGTTGTTTGGTGATTATATAGA

TGAATCACGATTGTAGGGTGGATGTTCGGTTGGTGATTCATCGGTTGTGGGGGGAGGGGAGTAGTAGGGCTGGGAATAAT

AGTGAGATGAGAATTAGGGGGATTCCTAGTAGGTGGGGGCTTATAAATTGGTCGAAGAAGCTTAGGTTCATGGTCAGGGT

CAGGATGAGGGTTTTGTGTTTGTTAGGGGGTTTTCTAGGGGGGAGTTAGTGGGCATGAATGAAAGGAGTTTGGGTTGGAT

GATTAGTGAAAAGGTCAGTCATGAAATAGTTATAATGAATAGTCATGGGTTTGGATTTAGTTGGGGCATATCATTAGGGA

GGGTTGGTAATCCTCTTTCTCTAGCTTAAAAGGCTAGTGCTGTTGCATAGCTTCCTAGTGGTTAAGATGATAGGAAAATT

AACATGATAAACATGATGATGGATGATGATTATTTTTTAATGTAATCTCGGTGTTGAATCTGGGTTAAAGTTAGATGATA

AAATGTGTGATGAAAATATCATGGATAGTATGGAAATTATAACTTAGAACTCCAGTGGTGTTGGTTAGAAAGTTAGAGGA

AGTGAAAAACTTGTAAAATATGTCCGGCAACCATAAATCAAAAAGCAACATAGCTTAGTAAGTATGTGAAAATACCATAA

CCAAATGCAAGACAAAGTGCATCAGTGTCAAAATGAGACTGAATTACACGTGAAACCGTCTCATTCAGCAACTCTTGAAG

GAGAGAAAAGGCTCGGGAGCCACTGATGCCCAGGTCAAGAGATTGTGTGCTCCGCGTTAGCCACTGGAAGGGCCTATTGA

GATTACCCAAAAAAAAAGA-GAACCAACC-GCCTAAAAGAAGGGAACCATGCCGCGATTAAGAAAGGTATGAACTATAAA

GTACCAACCAAATGCCTTTGAAAGGAAATTCACAGGGGAGGAGTCTA

8_Campylopterus_curvipennis_ord.2-06

GGATTATGGCTACTGCTACTTCTAGGATGGTTAGTAGGAGTAGTACGCAGGCGGTTAGAGCAGAGATTGCGGGTATGATG

GAGAATAGGACTGCGGTGGCTGTTGAGATGAGTTGGATTAGTAGGTGGCCTGCTGTTAGGTTAGCTGTGAGGCGGACTCC

TAGGGCTAGAGGGCGGATCAGGAGGCTGGTAGTTTCAATTATGATTAAGGCTGGGATTAGTGGAGTTGGGGTTCCTTCGG

GTAGGAGGTGGCCAAGTGAAGCGGATGGTTGGTTTCGGAGGCCTGTGAGTAGAGTGGCTAGTCATAGTGGGAAGGCTAGT

GCTAGGTTTATAGATAATTGTGTGGTAGGTGTGAATGTGTAGGGGAGTAGGCCTAGTAGGTTGATTGAGAGAAGGAAAAT

TATTAGTGATGACAGGATAAGGGCTCATTTGTGGCCTTTTTTGTTCAATGGGATTATCAGTTGTTTGGTGATTATATAGA

TGAATCACGATTGTAGGGTGGATGTTCGGTTGGTGATTCATCGGTTGTGGGGGGAGGGGAGTAGTAGGGCTGGGAATAAT

AGTGAGATGAGAATTAGGGGGATTCCTAGTAGGTGGGGGCTTATAAATTGGTCGAAGAAGCTTAGGTTCATGGTCAGGGT

CAGGATGAGGGTTTTGTGTTTGTTAGGGTTTTTTCTAGGGGGGAGTTAGTGGGCATGAATGAAAGGAGTTTGGGTTGGAT

GATTAGTGAAAAGGTCAGTCATGAAATAGTTATAATGAATAGTCATGGGTTTGGATTTAGTTGGGGCATATCATTAGGGA

GGGTTGGTAATCCTCTTTCTCTAGCTTAAAAGGCTAGTGCTGTTGCATAGCTTCCTAGTGGTTAAGATGATAGGAAAATT

AACATGATAAACATGATGATGGATGATGATTATTTTTTAATGTAATCTCGGTGTTGAATCTGGGTTAAAGTTAGATGATA

AAATGTGTGATGAAAATATCATGGATAGTATGGAAATTATAACTTAGAACTCCAGTGGTGTTGGTTAGAAAGTTAGAGGA

AGTGAAAAACTTGTAAAATATGTCCGGCAACCATAAATCAAAAAGCAACATAGCTTAGTAAGTATGTGAAAATACCATAA

CCAAATGCAAGACAAAGTGCATCAGTGTCAAAATGAGACTGAATTACACATGAAACCGTCTCATTCAGCAACTCTTGAAG

GAGAGAAAAGGCTCGGGAGCCACTGATGCCCAGGTCAAGAGATTGTGTGCTCCGCGTTAGCCACTGGAAGGGCCTATTGA

GATTACCCAAAAAAAAAGA-GAACCAACC-GCCTAAAAGAAGGGAACCATGCCGCGATTAAGAAAGGTATGAACTATAAA

GTACCAACCAAATGCCTTTGAAAGGAAATCCACAGGGGAGGAGTCTA

9_Campylopterus_curvipennis_ord.7-06

GGATTATGGCTACTGCTACTTCTAGGATGGTTAGTAGGAGTAGTACGCAGGCGGTTAGAGCAGAGATTGCGGGTATGATG

GAGAATAGGACTGCGGTGGCTGTTGAGATGAGTTGGATTAGTAGGTGGCCTGCTGTTAGGTTAGCTGTGAGGCGGACTCC

TAGGGCTAGAGGGCGGATCAGGAGGCTGGTAGTTTCAATTATGATTAAGGCTGGGATTAGTGGAGTTGGGGTTCCTTCGG

GTAGGAGGTGGCCAAGTGAAGCGGATGGTTGGTTTCGGAGGCCTGTGAGTAGAGTGGCTAGTCATAGTGGGAAGGCTAGT

GCTAGGTTTATAGATAATTGTGTGGTAGGTGTGAATGTGTAGGGGAGTAGGCCTAGTAGGTTGATTGAGAGAAGGAAAAT

TATTAGTGATGACAGGATAAGGGCTCATTTGTGGCCTTTTTTGTTCAATGGGATTATCAGTTGTTTGGTGATTATATAGA

TGAATCACGATTGTAGGGTGGATGTTCGGTTGGTGATTCATCGGTTGTGGGGGGAGGGGAGTAGTAGGGCTGGGAATAAT

AGTGAGATGAGAATTAGGGGGATTCCTAGTAGGTGGGGGCTTATAAATTGGTCGAAGAAGCTTAGGTTCATGGTCAGGGT

CAGGATGAGGGTTTTGTGTTTGTTAGGGGTTTTTCTAGGGGGGAGTTAGTGGGCATGAATGAAAGGAGTTTGGGTTGGAT

GATTAGTGAAAAGGTCAGTCATGAAATAGTTATAATGAATAGTCATGGGTTTGGATTTAGTTGGGGCATATCATTAGGGA

GGGTTGGTAATCCTCTTTCTCTAGCTTAAAAGGCTAGTGCTGTTGCATAGCTTCCTAGTGGTTAAGATGATAGGAAAATT

AACATGATAAACATGATGATGGATGATGATTATTTTTTAATGTAATCTCGGTGTTGAATCTGGGTTAAAGTTAGATGATA

AAATGTGTGATGAAAATATCATGGATAGTATGGAAATTATAACTTAGAACTCCAGTGGTGTTGGTTAGAAAGTTAGAGGA

AGTGAAAAACTTGTAAAATATGTCCGGCAACCATAAATCAAAAAGCAACATAGCTTAGTAAGTATGTGAAAATACCATAA

CCAAATGCAAGACAAAGTGCATCAGTGTCAAAATGAGACTGAATTACACGTGAAACCGTCTCATTCAGCAACTCTTGAAG

GAGAGAAAAGGCTCGGGAGCCACTGATGCTCAGGTCAAGAGATTGTGTGCTCCGCGTTAGCCACTGGAAGGGCCTATTGA

GATTACCCAAAAAAAAAGA-GAACCAACC-GCCTAAAAGAAGGGAACCATGCCGCGATTAAGAAAGGTATGAACTATAAA

GTACCAACCAAATGCCTTTGAAAGGAAATCCACAGGGGAGGAGTCTA

10_Campylopterus_curvipennis_ord.28-06

GGATTATGGCTACTGCTACTTCTAGGATGGTTAGTAGGAGTAGTACGCAGGCGGTTAGAGCAGAGATTGCGGGTATGATG

GAGAATAGGACTGCGGTGGCTGTTGAGATGAGTTGGATTAGTAGGTGGCCTGCTGTTAGGTTAGCTGTGAGGCGGACTCC

TAGGGCTAGAGGGCGGATCAGGAGGCTGGTAGTTTCAATTATGATTAAGGCTGGGATTAGTGGAGTTGGGGTTCCTTCGG

GTAGGAGGTGGCCAAGTGAAGCGGATGGTTGGTTTCGGAGGCCTGTGAGTAGAGTGGCTAGTCATAGTGGGAAGGCTAGT

GCTAGGTTTATAGATAATTGTGTGGTAGGTGTGAATGTGTAGGGGAGTAGGCCTAGTAGGTTGATTGAGAGAAGGAAAAT

TATTAGTGATGACAGGATAAGGGCTCATTTGTGGCCTTTTTTGTTCAATGGGATTATCAGTTGTTTGGTGATTATATAGA

TGAATCACGATTGTAGGGTGGATGTTCGGTTGGTGATTCATCGGTTGTGGGGGGAGGGGAGTAGTAGGGCTGGGAATAAT

AGTGAGATGAGAATTAGGGGGATTCCTAGTAGGTGGGGGCTTATAAATTGGTCGAAGAAGCTTAGGTTCATGGTCAGGGT

CAGGATGAGGGTTTTGTGTTTGTTAGGGTTTTTTCTAGGGGGGAGTTAGTGGGCATGAATGAAAGGAGTTTGGGTTGGAT

GATTAGTGAAAAGGTCAGTCATGAAATAGTTATAATGAATAGTCATGGGTTTGGATTTAGTTGGGGCATATCATTAGGGA

GGGTTGGTAATCCTCTTTCTCTAGCTTAAAAGGCTAGTGCTGTTGCATAGCTTCCTAGTGGTTAAGATGATAGGAAAATT

AACATGATAAACATGATGATGGATGATGATTATTTTTTAATGTAATCTCGGTGTTGAATCTGGGTTAAAGTTAGATGATA

AAATGTGTGATGAAAATATCATGGATAGTATGGAAATTATAACTTAGAACTCCAGTGGTGTTGGTTAGAAAGTTAGAGGA

AGTGAAAAACTTGTAAAATATGTCCGGCAACCATAAATCAAAAAGCAACATAGCTTAGTAAGTATGTGAAAATACCATAA

CCAAATGCAAGACAAAGTGCATCAGTGTCAAAATGAGACTGAATTACACATGAAACCGTCTCATTCAGCAACTCTTGAAG

GAGAGAAAAGGCTCGGGAGCCACTGATGCCCAGGTCAAGAGATTGTGTGCTCCGCGTTAGCCACTGGAAGGGCCTATTGA

GATTACCCAAAAAAAAAGA-GAACCAACC-GCCTAAAAGAAGGGAACCATGCCGCGATTAAGAAAGGTATGAACTATAAA

GTACCAACCAAATGCCTTTGAAAGGAAATCCACAGGGGAGGAGTCTA

11_Campylopterus_curvipennis_ord.01-07

GGATTATGGCTACTGCTACTTCTAGGATGGTTAGTAGGAGTAGTACGCAGGCGGTTAGAGCAGAGATTGCGGGTATGATG

GAGAATAGGACTGCGGTGGCTGTTGAGATGAGTTGGATTAGTAGGTGGCCTGCTGTTAGGTTAGCTGTGAGGCGGACTCC

TAGGGCTAGAGGGCGGATCAGGAGGCTGGTAGTTTCAATTATGATTAAGGCTGGGATTAGTGGAGTTGGGGTTCCTTCGG

GTAGGAGGTGGCCAAGTGAAGCGGATGGTTGGTTTCGGAGGCCTGTGAGTAGAGTGGCTAGTCATAGTGGGAAGGCTAGT

GCTAGGTTTATAGATAATTGTGTGGTAGGTGTGAATGTGTAGGGGAGTAGGCCTAGTAGGTTGATTGAGAGAAGGAAAAT

TATTAGTGATGACAGGATAAGGGCTCATTTGTGGCCTTTTTTGTTCAATGGGATTATCAGTTGTTTGGTGATTATATAGA

TGAATCACGATTGTAGGGTGGATGTTCGGTTGGTGATTCATCGGTTGTGGGGGGAGGGGAGTAGTAGGGCTGGGAATAAT

AGTGAGATGAGAATTAGGGGGATTCCTAGTAGGTGGGGGCTTATAAATTGGTCGAAGAAGCTTAGGTTCATGGTCAGGGT

CAGGATGAGGGTTTTGTGTTTGTTAGGGGTTTTTCTAGGGGGGAGTTAGTGGGCATGAATGAAAGGAGTTTGGGTTGGAT

GATTAGTGAAAAGGTCAGTCATGAAATAGTTATAATGAATAGTCATGGGTTTGGATTTAGTTGGGGCATATCATTAGGGA

GGGTTGGTAATCCTCTTTCTCTAGCTTAAAAGGCTAGTGCTGTTGCATAGCTTCCTAGTGGTTAAGATGATAGGAAAATT

AACATGATAAACATGATGATGGATGATGATTATTTTTTAATGTAATCTCGGTGTTGAATCTGGGTTAAAGTTAGATGATA

AAATGTGTGATGAAAATATCATGGATAGTATGGAAATTATAACTTAGAACTCCAGTGGTGTTGGTTAGAAAGTTAGAGGA

AGTGAAAAACTTGTAAAATATGTCCGGCAACCATAAATCAAAAAGCAACATAGCTTAGTAAGTATGTGAAAATACCATAA

CCAAATGCAAGACAAAGTGCATCAGTGTCAAAATGAGACTGAATTACACGTGAAACCGTCTCATTCAGCAACTCTTGAAG

GAGAGAAAAGGCTCGGGAGCCACTGATGCTCAGGTCAAGAGATTGTGTGCTCCGCGTTAGCCACTGGAAGGGCCTATTGA

GATTACCCAAAAAAAAAGA-GAACCAACC-GCCTAAAAGAAGGGAACCATGCCGCGATTAAGAAAGGTATGAACTATAAA

GTACCAACCAAATGCCTTTGAAAGGAAATCCACAGGGGAGGAGTCTA

12_Campylopterus_curvipennis_ord.3-07

GGATTATGGCTACTGCTACTTCTAGGATGGTTAGTAGGAGTAGTACGCAGGCGGTTAGGGCAGAGATTGCGGGTATGATG

GAGAATAGGACTGCGGTGGCTGTTGAGATGAGTTGGATTAGTAGGTGGCCTGCTGTTAGGTTAGCTGTGAGGCGGACTCC

TAGGGCTAGAGGGCGGATCAGGAGGCTGGTAGTTTCAATTATGATTAAGGCTGGGATTAGTGGAGTTGGGGTTCCTTCGG

GTAGGAGGTGGCCAAGTGAAGCGGATGGTTGGTTTCGGAGGCCTGTGAGTAGAGTGGCTAGTCATAGTGGGAAGGCTAGT

GCTAGGTTTATAGATAATTGTGTGGTAGGTGTGAATGTGTAGGGGAGTAGGCCTAGTAGGTTGATTGAGAGAAGGAAAAT

TATTAGTGATGACAGGATAAGGGCTCATTTGTGGCCTTTTTTGTTCAATGGGATTATCAGTTGTTTGGTGATTATATAGA

TGAATCACGATTGTAGGGTGGATGTTCGGTTGGTGATTCATCGGTTGTGGGGGGAGGGGAGTAGTAGGGCTGGGAATAAT

AGTGAGATGAGAATTAGGGGGATTCCTAGTAGGTGGGGGCTTATAAATTGGTCGAAGAAGCTTAGGTTCATGGTCAGGGT

CAGGATGAGGGTTTTGTGTTTGTTAGGGTTTTTTCTAGGGGGGAGTTAGTGGGCATGAATGAAAGGAGTTTGGGTTGGAT

GATTAGTGAAAAGGTCAGTCATGAAATAGTTATAATGAATAGTCATGGGTTTGGATTTAGTTGGGGCATATCATTAGGGA

GGGTTGGTAATCCTCTTTCTCTAGCTTAAAAGGCTAGTGCTGTTGCATAGCTTCCTAGTGGTTAAGATGATAGGAAAATT

AACATGATAAACATGATGATGGATGATGATTATTTTTTAATGTAATCTCGGTGTTGAATCTGGGTTAAAGTTAGATGATA

AAATGTGTGATGAAAATATCATGGATAGTATGGAAATTATAACTTAGAACTCCAGTGGTGTTGGTTAGAAAGTTAGAGGA

AGTGAAAAACTTGTAAAATATGTCCGGCAACCATAAATCAAAAAGCAACATAGCTTAGTAAGTATGTGAAAATACCATAA

CCAAATGCAAGACAAAGTGCATCAGTGTCAAAATGAGACTGAATTACACATGAAACCGTCTCATTCAGCAACTCTTGAAG

GAGAGAAAAGGCTCGGGAGCCACTGATGCCCAGGTCAAGAGATTGTGTGCTCCGCGTTAGCCACTGGAAGGGCCTATTGA

GATTACCCAAAAAAAAAGA-GAACCAACC-GCCTAAAAGAAGGGAACCATGCCGCGATTAAGAAAGGTATGAACTATAAA

GTACCAACCAAATGCCTTTGAAAGGAAATCCACAGGGGAGGAGTCTA

13_Campylopterus_curvipennis_ord.04-07

GGATTATGGCTACTGCTACTTCTAGGATGGTTAGTAGGAGTAGTACGCAGGCGGTTAGAGCAGAGATTGCGGGTATGATG

GAGAATAGGACTGCGGTGGCTGTTGAGATGAGTTGGATTAGTAGGTGGCCTGCTGTTAGGTTAGCTGTGAGGCGGACTCC

TAGGGCTAGAGGGCGGATCAGGAGGCTGGTAGTTTCAATTATGATTAAGGCTGGGATTAGTGGAGTTGGGGTTCCTTCGG

GTAGGAGGTGGCCAAGTGAAGCGGATGGTTGGTTTCGGAGGCCTGTGAGTAGAGTGGCTAGTCATAGTGGGAAGGCTAGT

GCTAGGTTTATAGATAATTGTGTGGTAGGTGTGAATGTGTAGGGGAGTAGGCCTAGTAGGTTGATTGAGAGAAGGAAAAT

TATTAGTGATGACAGGATAAGGGCTCATTTGTGGCCTTTTTTGTTCAATGGGATTATCAGTTGTTTGGTGATTATATAGA

TGAATCACGATTGTAGGGTGGATGTTCGGTTGGTGATTCATCGGTTGTGGGGGGAGGGGAGTAGTAGGGCTGGGAATAAT

AGTGAGATGAGAATTAGGGGGATTCCTAGTAGGTGGGGGCTTATAAATTGGTCGAAGAAGCTTAGGTTCATGGTCAGGGT

CAGGATGAGGGTTTTGTGTTTGTTAGGGTTTTTTCTAGGGGGGAGTTAGTGGGCATGAATGAAAGGAGTTTGGGTTGGAT

GATTAGTGAAAAGGTCAGTCATGAAATAGTTATAATGAATAGTCATGGGTTTGGATTTAGTTGGGGCATATCATTAGGGA

GGGTTGGTAATCCTCTTTCTCTAGCTTAAAAGGCTAGTGCTGTTGCATAGCTTCCTAGTGGTTAAGATGATAGGAAAATT

AACATGATAAACATGATGATGGATGATGATTATTTTTTAATGTAATCTCGGTGTTGAATCTGGGTTAAAGTTAGATGATA

AAATGTGTGATGAAAATATCATGGATAGTATGGAAATTATAACTTAGAACTCCAGTGGTGTTGGTTAGAAAGTTAGAGGA

AGTGAAAAACTTGTAAAATATGTCCGGCAACCATAAATCAAAAAGCAACATAGCTTAGTAAGTATGTGAAAATACCATAA

CCAAATGCAAGACAAAGTGCATCAGTGTCAAAATGAGACTGAATTACACATGAAACCGTCTCATTCAGCAACTCTTGAAG

GAGAGAAAAGGCTCGGGAGCCACTGATGCCCAGGTCAAGAGATTGTGTGCTCCGCGTTAGCCACTGGAAGGGCCTATTGA

GATTACCCAAAAAAAAAGA-GAACCAACC-GCCTAAAAGAAGGGAACCATGCCGCGATTAAGAAAGGTATGAACTATAAA

GTACCAACCAAATGCCTTTGAAAGGAAATCCACAGGGGAGGAGTCTA

14_Campylopterus_curvipennis_ord.10-07

GGATTATGGCTACTGCTACTTCTAGGATGGTTAGTAGGAGTAGTACGCAGGCGGTTAGAGCAGAGATTGCGGGTATGATG

GAGAATAGGACTGCGGTGGCTGTTGAGATGAGTTGGATTAGTAGGTGGCCTGCTGTTAGGTTAGCTGTGAGGCGGACTCC

TAGGGCTAGAGGGCGGATCAGGAGGCTGGTAGTTTCAATTATGATTAAGGCTGGGATTAGTGGAGTTGGGGTTCCTTCGG

GTAGGAGGTGGCCAAGTGAAGCGGATGGTTGGTTTCGGAGGCCTGTGAGTAGAGTGGCTAGTCATAGTGGGAAGGCTAGT

GCTAGGTTTATAGATAATTGTGTGGTAGGTGTGAATGTGTAGGGGAGTAGGCCTAGTAGGTTGATTGAGAGAAGGAAAAT

TATTAGTGATGACAGGATAAGGGCTCATTTGTGGCCTTTTTTGTTCAATGGGATTATCAGTTGTTTGGTGATTATATAGA

TGAATCACGATTGTAGGGTGGATGTTCGGTTGGTGATTCATCGGTTGTGGGGGGAGGGGAGTAGTAGGGCTGGGAATAAT

AGTGAGATGAGAATTAGGGGGATTCCTAGTAGGTGGGGGCTTATAAATTGGTCGAAGAAGCTTAGGTTCATGGTCAGGGT

CAGGATGAGGGTTTTGTGTTTGTTAGGGGTTTTTCTAGGGGGGAGTTAGTGGGCATGAATGAAAGGAGTTTGGGTTGGAT

GATTAGTGAAAAGGTCAGTCATGAAATAGTTATAATGAATAGTCATGGGTTTGGATTTAGTTGGGGCATATCATTAGGGA

GGGTTGGTAATCCTCTTTCTCTAGCTTAAAAGGCTAGTGCTGTTGCATAGCTTCCTAGTGGTTAAGATGATAGGAAAATT

AACATGATAAACATGATGATGGATGATGATTATTTTTTAATGTAATCTCGGTGTTGAATCTGAGTTAAAGTTAGATGATA

AAATGTGTGATGAAAATATCATGGATAGTATGGAAATTATAACTTAGAACTCCAGTGGTGTTGGTTAGAAAGTTAGAGGA

AGTGAAAAACTTGTAAAATATGTCCGGCAACCATAAATCAAAAAGCAACATAGCTTAGTAAGTATGTGAAAATACCATAA

CCAAATGCAAGACAAAGTGCATCAGTGTCAAAATGAGACTGAATTACACATGAAACCGTCTCATTCAGCAACTCTTGAAG

GAGAGAAAAGGCTCGGGAGCCACTGATGCTCAGGTCAAGAGATTGTGTGCTCCGCGTTAGCCACTGGAAGGGCCTATTGA

GATTACCCAAAAAAAAAGA-GAACCAACC-GCCTAAAAGAAGGGAACCATGCCGCGATTAAGAAAGGTATGAACTATAAA

GTACCAACCAAATGCCTTTGAAAGGAAATCCACAGGGGAGGAGTCTA

15_Campylopterus_curvipennis_ord.11-07

GGATTATGGCTACTGCTACTTCTAGGATGGTTAGTAGGAGTAGTACGCAGGCGGTTAGAGCAGAGATTGCGGGTATGATG

GAGAATAGGACTGCGGTGGCTGTTGAGATGAGTTGGATTAGTAGGTGGCCTGCTGTTAGGTTAGCTGTGAGGCGGACTCC

TAGGGCTAGAGGGCGGATCAGGAGGCTGGTAGTTTCAATTATGATTAAGGCTGGGATTAGTGGAGTTGGGGTTCCTTCGG

GTAGGAGGTGGCCAAGTGAAGCGGATGGTTGGTTTCGGAGGCCTGTGAGTAGAGTGGCTAGCCATAGTGGGAAGGCTAGT

GCTAGGTTTATAGATAATTGTGTGGTAGGTGTGAATGTGTAGGGGAGTAGGCCTAGTAGGTTGATTGAGAGAAGGAAAAT

TATTAGTGATGACAGGATAAGGGCTCATTTGTGGCCTTTTTTGTTCAATGGGATTATCAGTTGTTTGGTGATTATATAGA

TGAATCACGATTGTAGGGTGGATGTTCGGTTGGTGATTCATCGGTTGTGGGGGGAGGGGAGTAGTAGGGCTGGGAATAAT

AGTGAGATGAGAATTAGGGGGATTCCTAGTAGGTGGGGGCTTATAAATTGGTCGAAGAAGCTTAGGTTCATGGTCAGGGT

CAGGATGAGGGTTTTGTGTTTGTTAGGGTTTTTTCTAGGGGGGAGTTAGTGGGCATGAATGAAAGGAGTTTGGGTTGGAT

GATTAGTGAAAAGGTCAGTCATGAAATAGTTATAATGAATAGTCATGGGTTTGGATTTAGTTGGGGCATATCATTAGGGA

GGGTTGGTAATCCTCTTTCTCTAGCTTAAAAGGCTAGTGCTGTTGCATAGCTTCCTAGTGGTTAAGATGATAGGAAAATT

AACATGATAAACATGATGATGGATGATGATTATTTTTTAATGTAATCTCGGTGTTGAATCTGGGTTAAAGTTAGATGATA

AAATGTGTGATGAAAATATCATGGATAGTATGGAAATTATAACTTAGAACTCCAGTGGTGTTGGTTAGAAAGTTAGAGGA

AGTGAAAAACTTGTAAAATATGTCCGGCAACCATAAATCAAAAAGCAACATAGCTTAGTAAGTATGTGAAAATACCATAA

CCAAATGCAAGACAAAGTGCATCAGTGTCAAAATGAGACTGAATTACACATGAAACCGTCTCATTCAGCAACTCTTGAAG

GAGAGAAAAGGCTCGGGAGCCACTGATGCCCAGGTCAAGAGATTGTGTGCTCCGCGTTAGCCACTGGAAGGGCCTATTGA

GATTACCCAAAAAAAAAGA-GAACCAACC-GCCTAAAAGAAGGGAACCATGCCGCGATTAAGAAAGGTATGAACTATAAA

GTACCAACCAAATGCCTTTGAAAGGAAATCCACAGGGGAGGAGTCTA

16_Campylopterus_curvipennis_ord.12-07

GGATTATGGCTACTGCTACTTCTAGGATGGTTAGTAGGAGTAGTACGCAGGCGGTTAGAGCAGAGATTGCGGGTATGATG

GAGAATAGGACTGCGGTGGCTGTTGAGATGAGTTGGATTAGTAGGTGGCCTGCTGTTAGGTTAGCTGTGAGGCGGACTCC

TAGGGCTAGAGGGCGGATCAGGAGGCTGGTAGTTTCAATTATGATTAAGGCTGGGATTAGTGGAGTTGGGGTTCCTTCGG

GTAGGAGGTGGCCAAGTGAAGCGGATGGTTGGTTTCGGAGGCCTGTGAGTAGAGTGGCTAGTCATAGTGGGAAGGCTAGT

GCTAGGTTTATAGATAATTGTGTGGTAGGTGTGAATGTGTAGGGGAGTAGGCCTAGTAGGTTGATTGAGAGAAGGAAAAT

TATTAGTGATGACAGGATAAGGGCTCATTTGTGGCCTTTTTTGTTCAATGGGATTATCAGTTGTTTGGTGATTATATAGA

TGAATCACGATTGTAGGGTGGATGTTCGGTTGGTGATTCATCGGTTGTGGGGGGAGGGGAGTAGTAGGGCTGGGAATAAT

AGTGAGATGAGAATTAGGGGGATTCCTAGTAGGTGGGGGCTTATAAATTGGTCGAAGAAGCTTAGGTTCATGGTCAGGGT

CAGGATGAGGGTTTTGTGTTTGTTAGGGTTTTTTCTAGGGGGGAGTTAGTGGGCATGAATGAAAGGAGTTTGGGTTGGAT

GATTAGTGAAAAGGTCAGTCATGAAATAGTTATAATGAATAGTCATGGGTTTGGATTTAGTTGGGGCATATCATTAGGGA

GGGTTGGTAATCCTCTTTCTCTAGCTTAAAAGGCTAGTGCTGTTGCATAGCTTCCTAGTGGTTAAGATGATAGGAAAATT

AACATGATAAACATGATGATGGATGATGATTATTTTTTAATGTAATCTCGGTGTTGAATCTGGGTTAAAGTTAGATGATA

AAATGTGTGATGAAAATATCATGGATAGTATGGAAATTATAACTTAGAACTCCAGTGGTGTTGGTTAGAAAGTTAGAGGA

AGTGAAAAACTTGTAAAATATGTCCGGCAACCATAAATCAAAAAGCAACATAGCTTAGTAAGTATGTGAAAATACCATAA

CCAAATGCAAGACAAAGTGCATCAGTGTCAAAATGAGACTGAATTACACATGAAACCGTCTCATTCAGCAACTCTTGAAG

GAGAGAAAAGGCTCGGGAGCCACTGATGCCCAGGTCAAGAGATTGTGTGCTCCGCGTTAGCCACTGGAAGGGCCTATTGA

GATTACCCAAAAAAAAAGA-GAACCAACC-GCCTAAAAGAAGGGAACCATGCCGCGATTAAGAAAGGTATGAACTATAAA

GTACCAACCAAATGCCTTTGAAAGGAAATCCACAGGGGAGGAGTCTA

17_Campylopterus_curvipennis_ord. 17-07

GGATTATGGCTACTGCTACTTCTAGGATGGTTAGTAGGAGTAGTACGCAGGCGGTTAGAGCAGAGATTGCGGGTATGATG

GAGAATAGGACTGCGGTGGCTGTTGAGATGAGTTGGATTAGTAGGTGGCCTGCTGTTAGGTTAGCTGTGAGGCGGACTCC

TAGGGCTAGAGGGCGGATCAGGAGGCTGGTAGTTTCAATTATGATTAAGGCTGGGATTAGTGGAGTTGGGGTTCCTTCGG

GTAGGAGGTGGCCAAGTGAAGCGGATGGTTGGTTTCGGAGGCCTGTGAGTAGAGTGGCTAGTCATAGTGGGAAGGCTAGT

GCTAGGTTTATAGATAATTGTGTGGTAGGTGTGAATGTGTAGGGGAGTAGGCCTAGTAGGTTGATTGAGAGAAGGAAAAT

TATTAGTGATGACAGGATAAGGGCTCATTTGTGGCCTTTTTTGTTTAATGGGATTATCAGTTGTTTGGTGATTATATAGA

TGAATCACGATTGTAGGGTGGATGTTCGGTTGGTGATTCATCGGTTGTGGGGGGAGGGGAGTAGTAGGGCTGGGAATAAT

AGTGAGATGAGAATTAGGGGGATTCCTAGTAGGTGGGGGCTTATAAATTGGTCGAAGAAGCTTAGGTTCATGGTCAGGGT

CAGGATGAGGGTTTTGTGTTTGTTAGGGGTTTTTCTAGGGGGGAGTTAGTGGGCATGAATGAAAGGAGTTTGGGTTGGAT

GATTAGTGAAAAGGTCAGTCATGAAATAGTTATAATGAATAGTCATGGGTTTGGATTTAGTTGGGGCATATCATTAGGGA

GGGTTGGTAATCCTCTTTCTCTAGCTTAAAAGGCTAGTGCTGTTGCATAGCTTCCTAGTGGTTAAGATGATAGGAAAATT

AACATGATAAACATGATGATGGATGATGATTATTTTTTAATGTAATCTCGGTGTTGAATCTGGGTTAAAGTTAGATGATA

AAATGTGTGATGAAAATATCATGGATAGTATGGAAATTATAACTTAGAACTCCAGTGGTGTTGGTTAGAAAGTTAGAGGA

AGTGAAAAACTTGTAAAATATGTCCGGCAACCATAAATCAAAAAGCAACATAGCTTAGTAGGTATGTGAAAATACCATAA

CCAAATGCAAGACAAAGTGCATCAGTGTCAAAATGAGACTGAATTACACATGAAACCGTCTCATTCAGCAACTCTTGAAG

GAGAGAAAAGGCCCGGGAGCCACTGATGCTCAGGTCAAGAGATTGTGTGCTCCGCGTTAGCCACTGGAAGGGCCTATTGA

GATTACCCAAAAAAAAAGA-GAACCAACC-GCCTAAAAGAAGGGAACCATGCCGCGATTAAGAAAGGTATGAACTATAAA

GTACCAACCAAATGCCTTTGAAAGGAAATCCACAGGGGAGGAGTCTA

18_Campylopterus_curvipennis_ord.19-07

GGATTATGGCTACTGCTACTTCTAGGATGGTTAGTAGGAGTAGTACGCAGGCGGTTAGAGCAGAGATTGCGGGTATGATG

GAGAATAGGACTGCGGTGGCTGTTGAGATGAGTTGGATTAGTAGGTGGCCTGCTGTTAGGTTAGCTGTGAGGCGGACTCC

TAGGGCTAGAGGGCGGATCAGGAGGCTGGTAGTTTCAATTATGATTAAGGCTGGGATTAGTGGAGTTGGGGTTCCTTCGG

GTAGGAGGTGGCCAAGTGAAGCGGATGGTTGGTTTCGGAGGCCTGTGAGTAGAGTGGCTAGTCATAGTGGGAAGGCTAGT

GCTAGGTTTATAGATAATTGTGTGGTAGGTGTGAATGTGTAGGGGAGTAGGCCTAGTAGGTTGATTGAGAGAAGGAAAAT

TATTAGTGATGACAGGATAAGGGCTCATTTGTGGCCTTTTTTGTTCAATGGGATTATCAGTTGTTTGGTGATTATATAGA

TGAATCACGATTGTAGGGTGGATGTTCGGTTGGTGATTCATCGGTTGTGGGGGGAGGGGAGTAGTAGGGCTGGGAATAAT

AGTGAGATGAGAATTAGGGGGATTCCTAGTAGGTGGGGGCTTATAAATTGGTCGAAGAAGCTTAGGTTCATGGTCAGGGT

CAGGATGAGGGTTTTGTGTTTGTTAGGGGTTTTTCTAGGGGGGAGTTAGTGGGCATGAATGAAAGGAGTTTGGGTTGGAT

GATTAGTGAAAAGGTCAGTCATGAAATAGTTATAATGAATAGTCATGGGTTTGGATTTAGTTGGGGCATATCATTAGGGA

GGGTTGGTAATCCTCTTTCTCTAGCTTAAAAGGCTAGTGCTGTTGCATAGCTTCCTAGTGGTTAGGATGATAGGAAAATT

AACATGATAAACATGATGATGGATGATGATTATTTTTTAATGTAATCTCGGTGTTGAATCTGGGTTAAAGTTAGATGATA

AAATGTGTGATGAAAATATCATGGATAGTATGGAAATTATAACTTAGAACTCCAGTGGTGTTGGTTAGAAAGTTAGAGGA

AGTGAAAAACTTGTAAAATATGTCCGGCAACCATAAATCAAAAAGCAACATAGCTTAGTAAGTATGTGAAAATACCATAA

CCAAATGCAAGACAAAGTGCATCAGTGTCAAAATGAGACTGAATTACACATGAAACCGTCTCATTCAGCAACTCTTGAAG

GAGAGAAAAGGCCCGGGAGCCACTGATGCCCAGGTCAAGAGATTGTGTGCTCCGCGTTAGCCACTGGAAGGGCCTATTGA

GATTACCCAAAAAAAAAGA-GAACCAACC-GCCTAAAAGAAGGGAACCATGCCGCGATTAAGAAAGGTATGAACTATAAA

GTACCAACCAAATGCCTTTGAAAGGAAATCCACAGGGGAGGAATCTA

19_Campylopterus_curvipennis_ord.23-07

GGATTATGGCTACTGCTACTTCTAGGATGGTTAGTAGGAGTAGTACGCAGGCGGTTAGAGCAGAGATTGCGGGTATGATG

GAGAATAGGACTGCGGTGGCTGTTGAGATGAGTTGGATTAGTAGGTGGCCTGCTGTTAGGTTAGCTGTGAGGCGGACTCC

TAGGGCTAGAGGGCGGATCAGGAGGCTGGTAGTTTCAATTATGATTAAGGCTGGGATTAGTGGAGTTGGGGTTCCTTCGG

GTAGGAGGTGGCCAAGTGAAGCGGATGGTTGGTTTCGGAGGCCTGTGAGTAGAGTGGCTAGTCATAGTGGGAAGGCTAGT

GCTAGGTTTATAGATAATTGTGTGGTAGGTGTGAATGTGTAGGGGAGTAGGCCTAGTAGGTTGATTGAGAGAAGGAAAAT

TATTAGTGATGACAGGATAAGGGCTCATTTGTGGCCTTTTTTGTTCAATGGGATTATCAGTTGTTTGGTGATTATATAGA

TGAATCACGATTGTAGGGTGGATGTTCGGTTGGTGATTCATCGGTTGTGGGGGGAGGGGAGTAGTAGGGCTGGGAATAAT

AGTGAGATGAGAATTAGGGGGATTCCTAGTAGGTGGGGGCTTATAAATTGGTCGAAGAAGCTTAGGTTCATGGTCAGGGT

CAGGATGAGGGTTTTGTGTTTGTTAGGGGTTTTTCTAGGGGGGAGTTAGTGGGCATGAATGAAAGGAGTTTGGGTTGGAT

GATTAGTGAAAAGGTCAGTCATGAAATAGTTATAATGAATAGTCATGGGTTTGGATTTAGTTGGGGCATATCATTAGGGA

GGGTTGGTAATCCTCTTTCTCTAGCTTAAAAGGCTAGTGCTGTTGCATAGCTTCCTAGTGGTTAGGATGATAGGAAAATT

AACATGATAAACATGATGATGGATGATGATTATTTTTTAATGTAATCTCGGTGTTGAATCTGGGTTAAAGTTAGATGATA

AAATGTGTGATGAAAATATCATGGATAGTATGGAAATTATAACTTAGAACTCCAGTGGTGTTGGTTAGAAAGTTAGAGGA

AGTGAAAAACTTGTAAAATATGTCCGGCAACCATAAATCAAAAAGCAACATAGCTTAGTAAGTATGTGAAAATACCATAA

CCAAATGCAAGACAAAGTGCATCAGTGTCAAAATGAGACTGAATTACACATGAAACCGTCTCATTCAGCAACTCTTGAAG

GAGAGAAAAGGCCCGGGAGCCACTGATGCCCAGGTCAAGAGATTGTGTGCTCCGCGTTAGCCACTGGAAGGGCCTATTGA

GATTACCCAAAAAAAAAGA-GAACCAACC-GCCTAAAAGAAGGGAACCATGCCGCGATTAAGAAAGGTATGAACTATAAA

GTACCAACCAAATGCCTTTGAAAGGAAATCCACAGGGGAGGAATCTA

20_Campylopterus_curvipennis_ord.25-07

GGATTATGGCTACTGCTACTTCTAGGATGGTTAGTAGGAGTAGTACGCAGGCGGTTAGAGCAGAGATTGCGGGTATGATG

GAGAATAGGACTGCGGTGGCTGTTGAGATGAGTTGGATTAGTAGGTGGCCTGCTGTTAGGTTAGCTGTGAGGCGGACTCC

TAGGGCTAGAGGGCGGATCAGGAGGCTGGTAGTTTCAATTATGATTAAGGCTGGGATTAGTGGAGTTGGGGTTCCTTCGG

GTAGGAGGTGGCCAAGTGAAGCGGATGGTTGGTTTCGGAGGCCTGTGAGTAGAGTGGCTAGTCATAGTGGGAAGGCTAGT

GCTAGGTTTATAGATAATTGTGTGGTAGGTGTGAATGTGTAGGGGAGTAGGCCTAGTAGGTTGATTGAGAGAAGGAAAAT

TATTAGTGATGACAGGATAAGGGCTCATTTGTGGCCTTTTTTGTTTAATGGGATTATCAGTTGTTTGGTGATTATATAGA

TGAATCACGATTGTAGGGTGGATGTTCGGTTGGTGATTCATCGGTTGTGGGGGGAGGGGAGTAGTAGGGCTGGGAATAAT

AGTGAGATGAGAATTAGGGGGATTCCTAGTAGGTGGGGGCTTATAAATTGGTCGAAGAAGCTTAGGTTCATGGTCAGGGT

CAGGATGAGGGTTTTGTGTTTGTTAGGGGTTTTTCTAGGGGGGAGTTAGTGGGCATGAATGAAAGGAGTTTGGGTTGGAT

GATTAGTGAAAAGGTCAGTCATGAAATAGTTATAATGAATAGTCATGGGTTTGGATTTAGTTGGGGCATATCATTAGGGA

GGGTTGGTAATCCTCTTTCTCTAGCTTAAAAGGCTAGTGCTGTTGCATAGCTTCCTAGTGGTTAAGATGATAGGAAAATT

AACATGATAAACATGATGATGGATGATGATTATTTTTTAATGTAATCTCGGTGTTGAATCTGGGTTAAAGTTAGATGATA

AAATGTGTGATGAAAATATCATGGATAGTATGGAAATTATAACTTAGAACTCCAGTGGTGTTGGTTAGAAAGTTAGAGGA

AGTGAAAAACTTGTAAAATATGTCCGGCAACCATAAATCAAAAAGCAACATAGCTTAGTAAGTATGTGAAAATACCATAA

CCAAATGCAAGACAAAGTGCATCAGTGTCAAAATGAGACTGAATTACACATGAAACCGTCTCATTCAGCAACTCTTGAAG

GAGAGAAAAGGCTCGGGAGCCACTGATGCTCAGGTCAAGAGATTGTGTGCTCCGCGTTAGCCACTGGAAGGGCCTATTGA

GATTACCCAAAAAAAAAGA-GAACCAACC-GCCTAAAAGAAGGGAACCATGCCGCGATTAAGAAAGGTATGAACTATAAA

GTACCAACCAAATGCCTTTGAAAGGAAGTCCACAGGGGAGGAGTCTA

21_Campylopterus_curvipennis_ord.27-07

GGATTATGGCTACTGCTACTTCTAGGATGGTTAGTAGGAGTAGTACGCAGGCGGTTAGAGCAGAGATTGCGGGTATGATG

GAGAATAGGACTGCGGTGGCTGTTGAGATGAGTTGGATTAGTAGGTGGCCTGCTGTTAGGTTAGCTGTGAGGCGGACTCC

TAGGGCTAGAGGGCGGATCAGGAGGCTGGTAGTTTCAATTATGATTAAGGCTGGGATTAGTGGAGTTGGGGTTCCTTCGG

GTAGGAGGTGGCCAAGTGAAGCGGATGGTTGGTTTCGGAGGCCTGTGAGTAGAGTGGCTAGTCATAGTGGGAAGGCTAGT

GCTAGGTTTATAGATAATTGTGTGGTAGGTGTGAATGTGTAGGGGAGTAGGCCTAGTAGGTTGATTGAGAGAAGGAAAAT

TATTAGTGATGACAGGATAAGGGCTCATTTGTGGCCTTTTTTGTTTAATGGGATTATCAGTTGTTTGGTGATTATATAGA

TGAATCACGATTGTAGGGTGGATGTTCGGTTGGTGATTCATCGGTTGTGGGGGGAGGGGAGTAGTAGGGCTGGGAATAAT

AGTGAGATGAGAATTAGGGGGATTCCTAGTAGGTGGGGGCTTATAAATTGGTCGAAGAAGCTTAGGTTCATGGTCAGGGT

CAGGATGAGGGTTTTGTGTTTGTTAGGGGTTTTTCTAGGGGGGAGTTAGTGGGCATGAATGAAAGGAGTTTGGGTTGGAT

GATTAGTGAAAAGGTCAGTCATGAAATAGTTATAATGAATAGTCATGGGTTTGGATTTAGTTGGGGCATATCATTAGGGA

GGGTTGGTAATCCTCTTTCTCTAGCTTAAAAGGCTAGTGCTGTTGCATAGCTTCCTAGTGGTTAAGATGATAGGAAAATT

AACATGATAAACATGATGATGGATGATGATTATTTTTTAATGTAATCTCGGTGTTGAATCTGGGTTAAAGTTAGATGATA

AAATGTGTGATGAAAATATCATGGATAGTATGGAAATTATAACTTAGAACTCCAGTGGTGTTGGTTAGAAAGTTAGAGGA

AGTGAAAAACTTGTAAAATATGTCCGGCAACCATAAATCAAAAAGCAACATAGCTTAGTAAGTATGTGAAAATACCATAA

CCAAATGCAAGACAAAGTGCATCAGTGTCAAAATGAGACTGAATTACACATGAAACCGTCTCATTCAGCAACTCTTGAAG

GAGAGAAAAGGCTCGGGAGCCACTGATGCTCAGGTCAAGAGATTGTGTGCTCCGCGTTAGCCACTGGAAGGGCCTATTGA

GATTACCCAAAAAAAAAGA-GAACCAACC-GCCTAAAAGAAGGGAACCATGCCGCGATTAAGAAAGGTATGAACTATAAA

GTACCAACCAAATGCCTTTGAAAGGAAATCCACAGGGGAGGAGTCTA

22_Campylopterus_curvipennis_H-1

GGATTATGGCTACTGCTACTTCTAGGATGGTTAGTAGGAGTAGTACGCAGGCGGTTAGAGCAGAGATTGCGGGTATGATG

GAGAATAGGACTGCGGTGGCTGTTGAGATGAGTTGGATTAGTAGGTGGCCTGCTGTTAGGTTAGCTGTGAGGCGGACTCC

TAGGGCTAGAGGGCGGATCAGGAGGCTGGTAGTTTCAATTATGATTAAGGCTGGGATTAGTGGAGTTGGGGTTCCTTCGG

GTAGGAGGTGGCCAAGTGAAGCGGATGGTTGGTTTCGGAGGCCTGTGAGTAGAGTGGCTAGTCATAGTGGGAAGGCTAGT

GCTAGGTTTATAGATAATTGTGTGGTAGGTGTGAATGTGTAGGGGAGTAGGCCTAGTAGGTTGATTGAGAGAAGGAAAAT

TATTAGTGATGACAGGATAAGGGCTCATTTGTGGCCTTTTTTGTTCAATGGGATTATCAGTTGTTTGGTGATTATATAGA

TGAATCACGATTGTAGGGTGGATGTTCGGTTGGTGATTCATCGGTTGTGGGGGGAGGGGAGTAGTAGGGCTGGGAATAAT

AGTGAGATGAGAATTAGGGGGATTCCTAGTAGGTGGGGGCTTATAAATTGGTCGAAGAAGCTTAGGTTCATGGTCAGGGT

CAGGATGAGGGTTTTGTGTTTGTTAGGGGTTTTTCTAGGGGGGAGTTAGTGGGCATGAATGAAAGGAGTTTGGGTTGGAT

GATTAGTGAAAAGGTCAGTCATGAAATAGTTATAATGAATAGTCATGGGTTTGGATTTAGTTGGGGCATATCATTAGGGA

GGGTTGGTAATCCTCTTTCTCTAGCTTAAAAGGCTAGTGCTGTTGCATAGCTTCCTAGTGGTTAAGATGATAGGAAAATT

AACATGATAAACATGATGATGGATGATGATTATTTTTTAATGTAATCTCGGTGTTGAATCTGGGTTAAAGTTAGATGATA

AAATGTGTGATGAAAATATCATGGATAGTATGGAAATTATAACTTAGAACTCCAGTGGTGTTGGTTAGAAAGTTAGAGGA

AGTGAAAAACTTGTAAAATATGTCCGGCAACCATAAATCAAAAAGCAACATAGCTTAGTAAGTATGTGAAAATACCATAA

CCAAATGCAAGACAAAGTGCATCAGTGTCAAAATGAGACTGAATTACACATGAAACCGTCTCATTCAGCAACTCTTGAAG

GAGAGAAAAGGCTCGGGAGCCACTGATGCTCAGGTCAAGAGATTGTGTGCTCCGCGTTAGCCACTGGAAGGGCCTATTGA

GATTACCCAAAAAAAAAGA-GAACCAACC-GCCTAAAAGAAGGGAACCATGCCGCGATTAAGAAAGGTATGAACTATAAA

GTACCAACCAAATGCCTTTGAAAGGAAATCCACAGGGGAGGAGTCTA

23_Campylopterus_curvipennis_Mac-1

GGATTATGGCTACTGCTACTTCTAGGATGGTTAGTAGGAGTAGTACGCAGGCGGTTAGAGCAGAGATTGCGGGTATGATG

GAGAATAGGACTGCGGTGGCTGTTGAGATGAGTTGGATTAGTAGGTGGCCTGCTGTTAGGTTAGCTGTGAGGCGGACTCC

TAGGGCTAGAGGGCGGATCAGGAGGCTGGTAGTTTCAATTATGATTAAGGCTGGGATTAGTGGAGTTGGGGTTCCTTCGG

GTAGGAGGTGGCCAAGTGAAGCGGATGGTTGGTTTCGGAGGCCTGTGAGTAGAGTGGCTAGTCATAGTGGGAAGGCTAGT

GCTAGGTTTATAGATAATTGTGTGGTAGGTGTGAATGTGTAGGGGAGTAGGCCTAGTAGGTTGATTGAGAGAAGGAAAAT

TATTAGTGATGACAGGATAAGGGCTCATTTGTGGCCTTTTTTGTTCAATGGGATTATCAGTTGTTTGGTGATTATATAGA

TGAATCACGATTGTAGGGTGGATGTTCGGTTGGTGATTCATCGGTTGTGGGGGGAGGGGAGTAGTAGGGCTGGGAATAAT

AGTGAGATGAGAATTAGGGGGATTCCTAGTAGGTGGGGGCTTATAAATTGGTCGAAGAAGCTTAGGTTCATGGTCAGGGT

CAGGATGAGGGTTTTGTGTTTGTTAGGGTTTTTTCTAGGGGGGAGTTAGTGGGCATGAATGAAAGGAGTTTGGGTTGGAT

GATTAGTGAAAAGGTCAGTCATGAAATAGTTATAATGAATAGTCATGGGTTTGGATTTAGTTGGGGCATATCATTAGGGA

GGGTTGGTAATCCTCTTTCTCTAGCTTAAAAGGCTAGTGCTGTTGCATAGCTTCCTAGTGGTTAAGATGATAGGAAAATT

AACATGATAAACATGATGATGGATGATGATTATTTTTTAATGTAATCTCGGTGTTGAATCTGGGTTAAAGTTAGATGATA

AAATGTGTGATGAAAATATCATGGATAGTATGGAAATTATAACTTAGAACTCCAGTGGTGTTGGTTAGAAAGTTAGAGGA

AGTGAAAAACTTGTAAAATATGTCCGGCAACCATAAATCAAAAAGCAACATAGCTTAGTAAGTATGTGAAAATACCATAA

CCAAATGCAAGACAAAGTGCATCAGTGTCAAAATGAGACTGAATTACACATGAAACCGTCTCATTCAGCAACTCTTGAAG

GAGAGAAAAGGCTCGGGAGCCACTGATGCCCAGGTCAAGAGATTGTGTGCTCCGCGTTAGCCACTGGAAGGGCCTATTGA

GATTACCCAAAAAAAAAGA-GAACCAACC-GCCTAAAAGAAGGGAACCATGCCGCGATTAAGAAAGGTATGAACTATAAA

GTACCAACCAAATGCCTTTGAAAGGAAATCCACAGGGGAGGAGTCTA

24_Campylopterus_curvipennis_Mac-2

GGATTATGGCTACTGCTACTTCTAGGATGGTTAGTAGGAGTAGTACGCAGGCGGTTAGAGCAGAGATTGCGGGTATGATG

GAGAATAGGACTGCGGTGGCTGTTGAGATGAGTTGGATTAGTAGGTGGCCTGCTGTTAGGTTAGCTGTGAGGCGGACTCC

TAGGGCTAGAGGGCGGATCAGGAGGCTGGTAGTTTCAATTATGATTAAGGCTGGGATTAGTGGAGTTGGGGTTCCTTCGG

GTAGGAGGTGGCCAAGTGAAGTGGATGGTTGGTTTCGGAGGCCTGTGAGTAGAGTGGCTAGTCATAGTGGGAAGGCTAGT

GCTAGGTTTATAGATAATTGTGTGGTAGGTGTGAATGTGTAGGGGAGTAGGCCTAGTAGGTTGATTGAGAGAAGGAAAAT

TATTAGTGATGACAGGATAAGGGCTCATTTGTGGCCTTTTTTGTTCAATGGGATTATCAGTTGTTTGGTGATTATATAGA

TGAATCACGATTGTAGGGTGGATGTTCGGTTGGTGATTCATCGGTTGTGGGGGGAGGGGAGTAGTAGGGCTGGGAATAAT

AGTGAGATGAGAATTAGGGGGATTCCTAGTAGGTGGGGGCTTATAAATTGGTCGAAGAAGCTTAGGTTCATGGTCAGGGT

CAGGATGAGGGTTTTGTGTTTGTTAGGGTTTTTTCTAGGGGGGAGTTAGTGGGCATGAATGAAAGGAGTTTGGGTTGGAT

GATTAGTGAAAAGGTCAGTCATGAAATAGTTATAATGAATAGTCATGGGTTTGGATTTAGTTGGGGCATATCATTAGGGA

GGGTTGGTAATCCTCTTTCTCTAGCTTAAAAGGCTAGTGCTGTTGCATAGCTTCCTAGTGGTTAAGATGATAGGAAAATT

AACATGATAAACATGATGATGGATGATGATTATTTTTTAATGTAATCTCGGTGTTGAATCTGGGTTAAAGTTAGATGATA

AAATGTGTGATGAAAATATCATGGATAGTATGGAAATTATAACTTAGAACTCCAGTGGTGTTGGTTAGAAAGTTAGAGGA

AGTGAAAAACTTGTAAAATATGTCCGGCAACCATAAATCAAAAAGCAACATAGCTTAGTAAGTATGTGAAAATACCATAA

CCAAATGCAAGACAAAGTGCATCAGTGTCAAAATGAGACTGAATTACACATGAAACCGTCTCATTCAGCAACTCTTGAAG

GAGAGAAAAGGCTCGGGAGCCACTGATGCCCAGGTCAAGAGATTGTGTGCTCCGCGTTAGCCACTGGAAGGGCCTATTGA

GATTACCCAAAAAAAAAGA-GAACCAACC-GCCTAAAAGAAGGGAACCATGCCGCGATTAAGAAAGGTATGAACTATAAA

GTACCAACCAAATGCCTTTGAAAGGAAATCCACAGGGGAGGAGTCTA

25_Campylopterus_curvipennis_Mac-3

GGATTATGGCTACTGCTACTTCTAGGATGGTTAGTAGGAGTAGTACGCAGGCGGTTAGAGCAGAGATTGCGGGTATGATG

GAGAATAGGACTGCGGTGGCTGTTGAGATGAGTTGGATTAGTAGGTGGCCTGCTGTTAGGTTAGCTGTGAGGCGGACTCC

TAGGGCTAGAGGGCGGATCAGGAGGCTGGTAGTTTCAATTATGATTAAGGCTGGGATTAGTGGAGTTGGGGTTCCTTCGG

GTAGGAGGTGGCCAAGTGAAGCGGATGGTTGGTTTCGGAGGCCTGTGAGTAGAGTGGCTAGTCATAGTGGGAAGGCTAGT

GCTAGGTTTATAGATAATTGTGTGGTAGGTGTGAATGTGTAGGGGAGTAGGCCTAGTAGGTTGATTGAGAGAAGGAAAAT

TATTAGTGATGACAGGATAAGGGCTCATTTGTGGCCTTTTTTGTTCAATGGGATTATCAGTTGTTTGGTGATTATATAGA

TGAATCACGATTGTAGGGTGGATGTTCGGTTGGTGATTCATCGGTTGTGGGGGGAGGGGAGTAGTAGGGCTGGGAATAAT

AGTGAGATGAGAATTAGGGGGATTCCTAGTAGGTGGGGGCTTATAAATTGGTCGAAGAAGCTTAGGTTCATGGTCAGGGT

CAGGATGAGGGTTTTGTGTTTGTTAGGGGTTTTTCTAGGGGGGAGTTAGTGGGCATGAATGAAAGGAGTTTGGGTTGGAT

GATTAGTGAAAAGGTCAGTCATGAAATAGTTATAATGAATAGTCATGGGTTTGGATTTAGTTGGGGCATATCATTAGGGA

GGGTTGGTAATCCTCTTTCTCTAGCTTAAAAGGCTAGTGCTGTTGCATAGCTTCCTAGTGGTTAGGATGATAGGAAAATT

AACATGATAAACATGATGATGGATGATGATTATTTTTTAATGTAATCTCGGTGTTGAATCTGGGTTAAAGTTAGATGATA

AAATGTGTGATGAAAATATCATGGATAGTATGGAAATTATAACTTAGAACTCCAGTGGTGTTGGTTAGAAAGTTAGAGGA

AGTGAAAAACTTGTAAAATATGTCCGGCAACCATAAATCAAAAAGCAACATAGCTTAGTAAGTATGTGAAAATACCATAA

CCAAATGCAAGACAAAGTGCATCAGTGTCAAAATGAGACTGAATTACACATGAAACCGTCTCATTCAGCAACTCTTGAAG

GAGAGAAAAGGCCCGGGAGCCACTGATGCCCAGGTCAAGAGATTGTGTGCTCCGCGTTAGCCACTGGAAGGGCCTATTGA

GATTACCCAAAAAAAAAGA-GAACCAACC-GCCTAAAAGAAGGGAACCATGCCGCGATTAAGAAAGGTATGAACTATAAA

GTACCAACCAAATGCCTTTGAAAGGAAATCCACAGGGGAGGAATCTA

26_Campylopterus_curvipennis-UG-1

GGATTATGGCTACTGCTACTTCTAGGATGGTTAGTAGGAGTAGTACGCAGGCGGTTAGAGCAGAGATTGCGGGTATGATG

GAGAATAGGACTGCGGTGGCTGTTGAGATGAGTTGGATTAGTAGGTGGCCTGCTGTTAGGTTAGCTGTGAGGCGGACTCC

TAGGGCTAGAGGGCGGATCAGGAGGCTGGTAGTTTCAATTATGATTAAGGCTGGGATTAGTGGAGTTGGGGTTCCTTCGG

GTAGGAGGTGGCCAAGTGAAGCGGATGGTTGGTTTCGGAGGCCTGTGAGTAGAGTGGCTAGTCATAGTGGGAAGGCTAGT

GCTAGGTTTATAGATAATTGTGTGGTAGGTGTGAATGTGTAGGGGAGTAGGCCTAGTAGGTTGATTGAGAGAAGGAAAAT

TATTAGTGATGACAGGATAAGGGCTCATTTGTGGCCTTTTTTGTTCAATGGGATTATCAGTTGTTTGGTGATTATATAGA

TGAATCACGATTGTAGGGTGGATGTTCGGTTGGTGATTCATCGGTTGTGGGGGGAGGGGAGTAGTAGGGCTGGGAATAAT

AGTGAGATGAGAATTAGGGGGATTCCTAGTAGGTGGGGGCTTATAAATTGGTCGAAGAAGCTTAGGTTCATGGTCAGGGT

CAGGATGAGGGTTTTGTGTTTGTTAGGGTTTTTTCTAGGGGGGAGTTAGTGGGCATGAATGAAAGGAGTTTGGGTTGGAT

GATTAGTGAAAAGGTCAGTCATGAAATAGTTATAATGAATAGTCATGGGTTTGGATTTAGTTGGGGCATATCATTAGGGA

GGGTTGGTAATCCTCTTTCTCTAGCTTAAAAGGCTAGTGCTGTTGCATAGCTTCCTAGTGGTTAAGATGATAGGAAAATT

AACATGATAAACATGATGATGGATGATGATTATTTTTTAATGTAATCTCGGTGTTGAATCTGGGTTAAAGTTAGATGATA

AAATGTGTGATGAAAATATCATGGATAGTATGGAAATTATAACTTAGAACTCCAGTGGTGTTGGTTAGAAAGTTAGAGGA

AGTGAAAAACTTGTAAAATATGTCCGGCAACCATAAATCAAAAAGCAACATAGCTTAGTAAGTATGTGAAAATACCATAA

CCAAATGCAAGACAAAGTGCATCAGTGTCAAAATGAGACTGAATTACACATGAAACCGTCTCATTCAGCAACTCTTGAAG

GAGAGAAAAGGCTCGGGAGCCACTGATGCCCAGGTCAAGAGATTGTGTGCTCCGCGTTAGCCACTGGAAGGGCCTATTGA

GATTACCCAAAAAAAAAGA-GAACCAACC-GCCTAAAAGAAGGGAACCATGCCGCGATTAAGAAAGGTATGAACTATAAA

GTACCAACCAAATGCCTTTGAAAGGAAATCCACAGGGGAAGAGTCTA

27_Campylopterus_curvipennis_UG-2

GGATTATGGCTACTGCTACTTCTAGGATGGTTAGTAGGAGTAGTACGCAGGCGGTTAGAGCAGAGATTGCGGGTATGATG

GAGAATAGGACTGCGGTGGCTGTTGAGATGAGTTGGATTAGTAGGTGGCCTGCTGTTAGGTTAGCTGTGAGGCGGACTCC

TAGGGCTAGAGGGCGGATCAGGAGGCTGGTAGTTTCAATTATGATTAAGGCTGGGATTAGTGGAGTTGGGGTTCCTTCGG

GTAGGAGGTGGCCAAGTGAAGCGGATGGTTGGTTTCGGAGGCCTGTGAGTAGAGTGGCTAGTCATAGCGGGAAGGCTAGT

GCTAGGTTTATAGATAATTGTGTGGTAGGTGTGAATGTGTAGGGGAGTAGGCCTAGTAGGTTGATTGAGAGAAGGAAAAT

TATTAGTGATGACAGGATAAGGGCTCATTTGTGGCCTTTTTTGTTCAATGGGATTATCAGTTGTTTGGTGATTATATAGA

TGAATCACGATTGTAGGGTGGATGTTCGGTTGGTGATTCATCGGTTGTGGGGGGAGGGGAGTAGTAGGGCTGGGAATAAT

AGTGAGATGAGAATTAGGGGGATTCCTAGTAGGTGGGGGCTTATAAATTGGTCGAAGAAGCTTAGGTTCATGGTCAGGGT

CAGGATGAGGGTTTTGTGTTTGTTAGGGTTTTTTCTAGGGGGGAGTTAGTGGGCATGAATGAAAGGAGTTTGGGTTGGAT

GATTAGTGAAAAGGTCAGTCATGAAATAGTTATAATGAATAGTCATGGGTTTGGATTTAGTTGGGGCATATCATTAGGGA

GGGTTGGTAATCCTCTTTCTCTAGCTTAAAAGGCTAGTGCTGTTGCATAGCTTCCTAGTGGTTAAGATGATAGGAAAATT

AACATGATAAACATGATGATGGATGATGATTATTTTTTAATGTAATCTCGGTGTTGAATCTGGGTTAAAGTTAGATGATA

AAATGTGTGATGAAAATATCATGGATAGTATGGAAATTATAACTTAGAACTCCAGTGGTGTTGGTTAGAAAGTTAGAGGA

AGTGAAAAACTTGTAAAATATGTCCGGCAACCATAAATCAAAAAGCAACATAGCTTAGTAAGTATGTGAAAATACCATAA

CCAAATGCAAGACAAAGTGCATCAGTGTCAAAATGAGACTGAATTACACATGAAACCGTCTCATTCAGCAACTCTTGAAG

GAGAGAAAAGGCCCGGGAGCCACTGATGCCCAGGTCAAGAGATTGTGTGCTCCGCGTTAGCCACTGGAAGGGCCTATTGA

GATTACCCAAAAAAAAAGA-GAACCAACC-GCCTAAAAGAAGGGAACCATGCCGCGATTAAGAAAGGTATGAACTATAAA

GTACCAACCAAATGCCTTTGAAAGGAAATCCACAGGGGAGGAGTCTA

28_Campylopterus_curvipennis_UG-3

GGATTATGGCTACTGCTACTTCTAGGATGGTTAGTAGGAGTAGTACGCAGGCGGTTAGAGCAGAGATTGCGGGTATGATG

GAGAATAGGACTGCGGTGGCTGTTGAGATGAGTTGGATTAGTAGGTGGCCTGCTGTTAGGTTAGCTGTGAGGCGGACTCC

TAGGGCTAGAGGGCGGATCAGGAGGCTGGTAGTTTCAATTATGATTAAGGCTGGGATTAGTGGAGTTGGGGTTCCTTCGG

GTAGGAGGTGGCCAAGTGAAGCGGATGGTTGGTTTCGGAGGCCTGTGAGTAGAGTGGCTAGTCATAGTGGGAAGGCTAGT

GCTAGGTTTATAGATAATTGTGTGGTAGGTGTGAATGTGTAGGGGAGTAGGCCTAGTAGGTTGATTGAGAGAAGGAAAAT

TATTAGTGATGACAGGATAAGGGCTCATTTGTGGCCTTTTTTGTTCAATGGGATTATCAGTTGTTTGGTGATTATATAGA

TGAATCACGATTGTAGGGTGGATGTTCGGTTGGTGATTCATCGGTTGTGGGGGGAGGGGAGTAGTAGGGCTGGGAATAAT

AGTGAGATGAGAATTAGGGGGATTCCTAGTAGGTGGGGGCTTATAAATTGGTCGAAGAAGCTTAGGTTCATGGTCAGGGT

CAGGATGAGGGTTTTGTGTTTGTTAGGGTTTTTTCTAGGGGGGAGTTAGTGGGCATGAATGAAAGGAGTTTGGGTTGGAT

GATTAGTGAAAAGGTCAGTCATGAAATAGTTATAATGAATAGTCATGGGTTTGGATTTAGTTGGGGCATATCATTAGGGA

GGGTTGGTAATCCTCTTTCTCTAGCTTAAAAGGCTAGTGCTGTTGCATAGCTTCCTAGTGGTTAAGATGATAGGAAAATT

AACATGATAAACATGATGATGGATGATGATTATTTTTTAATGTAATCTCGGTGTTGAATCTGGGTTAAAGTTAGATGATA

AAATGTGTGATGAAAATATCATGGATAGTATGGAAATTATAACTTAGAACTCCAGTGGTGTTGGTTAGAAAGTTAGAGGA

AGTGAAAAACTTGTAAAATATGTCCGGCAACCATAAATCAAAAAGCAACATAGCTTAGTAAGTATGTGAAAATACCATAA

CCAAATGCAAGACAAAGTGCATCAGTGTCAAAATGAGACTGAATTACACATGAAACCGTCTCATTCAGCAACTCTTGAAG

GAGAGAAAAGGCTCGGGAGCCACTGATGCCCAGGTCAAGAGATTGTGTGCTCCGCGTTAGCCACTGGAAGGGCCTATTGA

GATTACCCAAAAAAAAAGA-GAACCAACC-GCCTAAAAGAAGGGAACCATGCCGCGATTAAGAAAGGTATGAACTATAAA

GTACCAACCAAATGCCTTTGAAAGGAAATCCACAGGGGAGGAGTCTG

29_Campylopterus_curvipennis_UG-4

GGATTATGGCTACTGCTACTTCTAGGATGGTTAGTAGGAGTAGTACGCAGGCGGTTAGAGCAGAGATTGCGGGTATGATG

GAGAATAGGACTGCGGTGGCTGTTGAGATGAGTTGGATTAGTAGGTGGCCTGCTGTTAGGTTAGCTGTGAGGCGGACTCC

TAGGGCTAGAGGGCGGATCAGGAGGCTGGTAGTTTCAATTATGATTAAGGCTGGGATTAGTGGAGTTGGGGTTCCTTCGG

GTAGGAGGTGGCCAAGTGAAGCGGATGGTTGGTTTCGGAGGCCTGTGAGTAGAGTGGCTAGTCATAGTGGGAAGGCTAGT

GCTAGGTTTATAGATAATTGTGTGGTAGGTGTGAATGTGTAGGGGAGTAGGCCTAGTAGGTTGATTGAGAGAAGGAAAAT

TATTAGTGATGACAGGATAAGGGCTCATTTGTGGCCTTTTTTGTTCAATGGGATTATCAGTTGTTTGGTGATTATATAGA

TGAATCACGATTGTAGGGTGGATGTTCGGTTGGTGATTCATCGGTTGTGGGGGGAGGGGAGTAGTAGGGCTGGGAATAAT

AGTGAGATGAGAATTAGGGGGATTCCTAGTAGGTGGGGGCTTATAAATTGGTCGAAGAAGCTTAGGTTCATGGTCAGGGT

CAGGATGAGGGTTTTGTGTTTGTTAGGGTTTTTTCTAGGGGGGAGTTAGTGGGCATGAATGAAAGGAGTTTGGGTTGGAT

GATTAGTGAAAAGGTCAGTCATGAAATAGTTATAATGAATAGTCATGGGTTTGGATTTAGTTGGGGCATATCATTAGGGA

GGGTTGGTAATCCTCTTTCTCTAGCTTAAAAGGCTAGTGCTGTTGCATAGCTTCCTAGTGGTTAAGATGATAGGAAAATT

AACATGATAAACATGATGATGGATGATGATTATTTTTTAATGTAATCTCGGTGTTGAATCTGGGTTAAAGTTAGATGATA

AAATGTGTGATGAAAATATCATGGATAGTATGGAAATTATAACTTAGAACTCCAGTGGTGTTGGTTAGAAAGTTAGAGGA

AGTGAAAAACTTGTAAAATATGTCCGGCAACCATAAATCAAAAAGCAACATAGCTTAGTAAGTATGTGAAGATACCATAA

CCAAATGCAAGACAAAGTGCATCAGTGTCAAAATGAGACTGAATTACACATGAAACCGTCTCATTCAGCAACTCTTGAAG

GAGAGAAAAGGCTCGGGAGCCACTGATGCCCAGGTCAAGAGATTGTGTGCTCCGCGTTAGCCACTGGAAGGGCCTATTGA

GATTACCCAAAAAAAAAGA-GAACCAACC-GCCTAAAAGAAGGGAACCATGCCGCGATTAAGAAAGGTATGAACTATAAA

GTACCAACCAAATGCCTTTGAAAGGAAATCCACAGGGGAGGAGTCTA

30_Campylopterus_curvipennis_UG-5

GGATTATGGCTACTGCTACTTCTAGGATGGTTAGTAGGAGTAGTACGCAGGCGGTTAGAGCAGAGATTGCGGGTATGATG

GAGAATAGGACTGCGGTGGCTGTTGAGATGAGTTGGATTAGTAGGTGGCCTGCTGTTAGGTTAGCTGTGAGGCGGACTCC

TAGGGCTAGAGGGCGGATCAGGAGGCTGGTAGTTTCAATTATGATTAAGGCTGGGATTAGTGGAGTTGGGGTTCCTTCGG

GTAGGAGGTGGCCAAGTGAAGCGGATGGTTGGTTTCGGAGGCCTGTGAGTAGAGTGGCTAGTCATAGCGGGAAGGCTAGT

GCTAGGTTTATAGATAATTGTGTGGTAGGTGTGAATGTGTAGGGGAGTAGGCCTAGTAGGTTGATTGAGAGAAGGAAAAT

TATTAGTGATGACAGGATAAGGGCTCATTTGTGGCCTTTTTTGTTCAATGGGATTATCAGTTGTTTGGTGATTATATAGA

TGAATCACGATTGTAGGGTGGATGTTCGGTTGGTGATTCATCGGTTGTGGGGGGAGGGGAGTAGTAGGGCTGGGAATAAT

AGTGAGATGAGAATTAGGGGGATTCCTAGTAGGTGGGGGCTTATAAATTGGTCGAAGAAGCTTAGGTTCATGGTCAGGGT

CAGGATGAGGGTTTTGTGTTTGTTAGGGTTTTTTCTAGGGGGGAGTTAGTGGGCATGAATGAAAGGAGTTTGGGTTGGAT

GATTAGTGAAAAGGTCAGTCATGAAATAGTTATAATGAATAGTCATGGGTTTGGATTTAGTTGGGGCATATCATTAGGGA

GGGTTGGTAATCCTCTTTCTCTAGCTTAAAAGGCTAGTGCTGTTGCATAGCTTCCTAGTGGTTAAGATGATAGGAAAATT

AACATGATAAACATGATGATGGATGATGATTATTTTTTAATGTAATCTCGGTGTTGAATCTGGGTTAAAGTTAGATGATA

AAATGTGTGATGAAAATATCATGGATAGTATGGAAATTATAACTTAGAACTCCAGTGGTGTTGGTTAGAAAGTTAGAGGA

AGTGAAAAACTTGTAAAATATGTCCGGCAACCATAAATCAAAAAGCAACATAGCTTAGTAAGTATGTGAAAATACCATAA

CCAAATGCAAGACAAAGTGCATCAGTGTCAAAATGAGACTGAATTACACATGAAACCGTCTCATTCAGCAACTCTTGAAG

GAGAGAAAAGGCCCGGGAGCCACTGATGCCCAGGTCAAGAGATTGTGTGCTCCGCGTTAGCCACTGGAAGGGCCTATTGA

GATTACCCAAAAAAAAAGA-GAACCAACC-GCCTAAAAGAAGGGAACCATGCCGCGATTAAGAAAGGTATGAACTATAAA

GTACCAACCAAATGCCTTTGAAAGGAAATCCACAGGGGAGGAGTCTA

31_Campylopterus_curvipennis_UG-6

GGATTATGGCTACTGCTACTTCTAGGATGGTTAGTAGGAGTAGTACGCAGGCGGTTAGAGCAGAGATTGCGGGTATGATG

GAGAATAGGACTGCGGTGGCTGTTGAGATGAGTTGGATTAGTAGGTGGCCTGCTGTTAGGTTAGCTGTGAGGCGGACTCC

TAGGGCTAGAGGGCGGATCAGGAGGCTGGTAGTTTCAATTATGATTAAGGCTGGGATTAGTGGAGTTGGGGTTCCTTCGG

GTAGGAGGTGGCCAAGTGAAGCGGATGGTTGGTTTCGGAGGCCTGTGAGTAGAGTGGCTAGTCATAGTGGGAAGGCTAGT

GCTAGGTTTATAGATAATTGTGTGGTAGGTGTGAATGTGTAGGGGAGTAGGCCTAGTAGGTTGATTGAGAGAAGGAAAAT

TATTAGTGATGACAGGATAAGGGCTCATTTGTGGCCTTTTTTGTTCAATGGGATTATCAGTTGTTTGGTGATTATATAGA

TGAATCACGATTGTAGGGTGGATGTTCGGTTGGTGATTCATCGGTTGTGGGGGGAGGGGAGTAGTAGGGCTGGGAATAAT

AGTGAGATGAGAATTAGGGGGATTCCTAGTAGGTGGGGGCTTATAAATTGGTCGAAGAAGCTTAGGTTCATGGTCAGGGT

CAGGATGAGGGTTTTGTGTTTGTTAGGGTTTTTTCTAGGGGGGAGTTAGTGGGCATGAATGAAAGGAGTTTGGGTTGGAT

GATTAGTGAAAAGGTCAGTCATGAAATAGTTATAATGAATAGTCATGGGTTTGGATTTAGTTGGGGCATATCATTAGGGA

GGGTTGGTAATCCTCTTTCTCTAGCTTAAAAGGCTAGTGCTGTTGCATAGCTTCCTAGTGGTTAAGATGATAGGAAAATT

AACATGATAAACATGATGATGGATGATGATTATTTTTTAATGTAATCTCGGTGTTGAATCTGGGTTAAAGTTAGATGATA

AAATGTGTGATGAAAATATCATGGATAGTATGGAAATTATAACTTAGAACTCCAGTGGTGTTGGTTAGAAAGTTAGAGGA

AGTGAAAAACTTGTAAAATATGTCCGGCAACCATAAATCAAAAAGCAACATAGCTTAGTAAGTATGTGAAAATACCATAA

CCAAATGCAAGACAAAGTGCATCAGTGTCAAAATGAGACTGAATTACACATGAAACCGTCTCATTCAGCAACTCTTGAAG

GAGAGAAAAGGCTCGGGAGCCACTGATGCCCAGGTCAAGAGATTGTGTGCTCCGCGTTAGCCACTGGAAGGGCCTATTGA

GATTACCCAAAAAAAAAGA-GAACCAACC-GCCTAAAAGAAGGGAACCATGCCGCGATTAAGAAAGGTATGAACTATAAA

GTACCAACCAAATGCCTTTGAAAGGAAATCCACAGGGGAGGAGTCTA

32_Campylopterus_curvipennis_UG-7

GGATTATGGCTACTGCTACTTCTAGGATGGTTAGTAGGAGTAGTACGCAGGCGGTTAGAGCAGAGATTGCGGGTATGATG

GAGAATAGGACTGCGGTGGCTGTTGAGATGAGTTGGATTAGTAGGTGGCCTGCTGTTAGGTTAGCTGTGAGGCGGACTCC

TAGGGCTAGAGGGCGGATCAGGAGGCTGGTAGTTTCAATTATGATTAAGGCTGGGATTAGTGGAGTTGGGGTTCCTTCGG

GTAGGAGGTGGCCAAGTGAAGCGGATGGTTGGTTTCGGAGGCCTGTGAGTAGAGTGGCTAGTCATAGTGGGAAGGCTAGT

GCTAGGTTTATAGATAATTGTGTGGTAGGTGTGAATGTGTAGGGGAGTAGGCCTAGTAGGTTGATTGAGAGAAGGAAAAT

TATTAGTGATGACAGGATAAGGGCTCATTTGTGGCCTTTTTTGTTCAATGGGATTATCAGTTGTTTGGTGATTATATAGA

TGAATCACGATTGTAGGGTGGATGTTCGGTTGGTGATTCATCGGTTGTGGGGGGAGGGGAGTAGTAGGGCTGGGAATAAT

AGTGAGATGAGAATTAGGGGGATTCCTAGTAGGTGGGGGCTTATAAATTGGTCGAAGAAGCTTAGGTTCATGGTCAGGGT

CAGGATGAGGGTTTTGTGTTTGTTAGGGTTTTTTCTAGGGGGGAGTTAGTGGGCATGAATGAAAGGAGTTTGGGTTGGAT

GATTAGTGAAAAGGTCAGTCATGAAATAGTTATAATGAATAGTCATGGGTTTGGATTTAGTTGGGGCATATCATTAGGGA

GGGTTGGTAATCCTCTTTCTCTAGCTTAAAAGGCTAGTGCTGTTGCATAGCTTCCTAGTGGTTAAGATGATAGGAAAATT

AACATGATAAACATGATGATGGATGATGATTATTTTTTAATGTAATCTCGGTGTTGAATCTGGGTTAAAGTTAGATGATA

AAATGTGTGATGAAAATATCATGGATAGTATGGAAATTATAACTTAGAACTCCAGTGGTGTTGGTTAGAAAGTTAGAGGA

AGTGAAAAACTTGTAAAATATGTCCGGCAACCATAAATCAAAAAGCAACATAGCTTAGTAAGTATGTGAAAATACCATAA

CCAAATGCAAGACAAAGTGCATCAGTGTCAAAATGAGACTGAATTACACATGAAACCGTCTCATTCAGCAACTCTTGAAG

GAGAGAAAAGGCTCGGGAGCCACTGATGCCCAGGTCAAGAGATTGTGTGCTCCGCGTTAGCCACTGGAAGGGCCTATTGA

GATTACCCAAAAAAAAAGA-GAACCAACC-GCCTAAAAGAAGGGAACCATGCCGCGATTAAGAAAGGTATGAACTATAAA

GTACCAACCAAATGCCTTTGAAAGGAAATCCACAGGGGAGGAGTCTA

33_Campylopterus_curvipennis_UG-8

GGATTATGGCTACTGCTACTTCTAGGATGGTTAGTAGGAGTAGTACGCAGGCGGTTAGAGCAGAGATTGCGGGTATGATG

GAGAATAGGACTGCGGTGGCTGTTGAGATGAGTTGGATTAGTAGGTGGCCTGCTGTTAGGTTAGCTGTGAGGCGGACTCC

TAGGGCTAGAGGGCGGATCAGGAGGCTGGTAGTTTCAATTATGATTAAGGCTGGGATTAGTGGAGTTGGGGTTCCTTCGG

GTAGGAGGTGGCCAAGTGAAGCGGATGGTTGGTTTCGGAGGCCTGTGAGTAGAGTGGCTAGTCATAGTGGGAAGGCTAGT

GCTAGGTTTATAGATAATTGTGTGGTAGGTGTGAATGTGTAGGGGAGTAGGCCTAGTAGGTTGATTGAGAGAAGGAAAAT

TATTAGTGATGACAGGATAAGGGCTCATTTGTGGCCTTTTTTGTTTAATGGGATTATCAGTTGTTTGGTGATTATATAGA

TGAATCACGATTGTAGGGTGGATGTTCGGTTGGTGATTCATCGGTTGTGGGGGGAGGGGAGTAGTAGGGCTGGGAATAAT

AGTGAGATGAGAATTAGGGGGATTCCTAGTAGGTGGGGGCTTATAAATTGGTCGAAGAAGCTTAGGTTCATGGTCAGGGT

CAGGATGAGGGTTTTGTGTTTGTTAGGGGTTTTTCTAGGGGGGAGTTAGTGGGCATGAATGAAAGGAGTTTGGGTTGGAT

GATTAGTGAAAAGGTCAGTCATGAAATAGTTATAATGAATAGTCATGGGTTTGGATTTAGTTGGGGCATATCATTAGGGA

GGGTTGGTAATCCTCTTTCTCTAGCTTAAAAGGCTAGTGCTGTTGCATAGCTTCCTAGTGGTTAAGATGATAGGAAAATT

AACATGATAAACATGATGATGGATGATGATTATTTTTTAATGTAATCTCGGTGTTGAATCTGGGTTAAAGTTAGATGATA

AAATGTGTGATGAAAATATCATGGATAGTATGGAAATTATAACTTAGAACTCCAGTGGTGTTGGTTAGAAAGTTAGAGGA

AGTGAAAAACTTGTAAAATATGTCCGGCAACCATAAATCAAAAAGCAACATAGCTTAGTAAGTATGTGAAAATACCATAA

CCAAATGCAAGACAAAGTGCATCAGTGTCAAAATGAGACTGAATTACACATGAAACCGTCTCATTCAGCAACTCTTGAAG

GAGAGAAAAGGCTCGGGAGCCACTGATGCTCAGGTCAAGAGATTGTGTGCTCCGCGTTAGCCACTGGAAGGGCCTATTGA

GATTACCCAAAAAAAAAGA-GAACCAACC-GCCTAAAAGAAGGGAACCATGCCGCGATTAAGAAAGGTATGAACTATAAA

GTACCAACCAAATGCCTTTGAAAGGAAATCCACAGGGGAGGAGTCTA

34_Campylopterus_curvipennis_UG-9

GGATTATGGCTACTGCTACTTCTAGGATGGTTAGTAGGAGTAGTACGCAGGCGGTTAGAGCAGAGATTGCGGGTATGATG

GAGAATAGGACTGCGGTGGCTGTTGAGATGAGTTGGATTAGTAGGTGGCCTGCTGTTAGGTTAGCTGTGAGGCGGACTCC

TAGGGCTAGAGGGCGGATCAGGAGGCTGGTAGTTTCAATTATGATTAAGGCTGGGATTAGTGGAGTTGGGGTTCCTTCGG

GTAGGAGGTGGCCAAGTGAAGCGGATGGTTGGTTTCGGAGGCCTGTGAGTAGAGTGGCTAGTCATAGCGGGAAGGCTAGT

GCTAGGTTTATAGATAATTGTGTGGTAGGTGTGAATGTGTAGGGGAGTAGGCCTAGTAGGTTGATTGAGAGAAGGAAAAT

TATTAGTGATGACAGGATAAGGGCTCATTTGTGGCCTTTTTTGTTCAATGGGATTATCAGTTGTTTGGTGATTATATAGA

TGAATCACGATTGTAGGGTGGATGTTCGGTTGGTGATTCATCGGTTGTGGGGGGAGGGGAGTAGTAGGGCTGGGAATAAT

AGTGAGATGAGAATTAGGGGGATTCCTAGTAGGTGGGGGCTTATAAATTGGTCGAAGAAGCTTAGGTTCATGGTCAGGGT

CAGGATGAGGGTTTTGTGTTTGTTAGGGTTTTTTCTAGGGGGGAGTTAGTGGGCATGAATGAAAGGAGTTTGGGTTGGAT

GATTAGTGAAAAGGTCAGTCATGAAATAGTTATAATGAATAGTCATGGGTTTGGATTTAGTTGGGGCATATCATTAGGGA

GGGTTGGTAATCCTCTTTCTCTAGCTTAAAAGGCTAGTGCTGTTGCATAGCTTCCTAGTGGTTAAGATGATAGGAAAATT

AACATGATAAACATGATGATGGATGATGATTATTTTTTAATGTAATCTCGGTGTTGAATCTGGGTTAAAGTTAGATGATA

AAATGTGTGATGAAAATATCATGGATAGTATGGAAATTATAACTTAGAACTCCAGTGGTGTTGGTTAGAAAGTTAGAGGA

AGTGAAAAACTTGTAAAATATGTCCGGCAACCATAAATCAAAAAGCAACATAGCTTAGTAAGTATGTGAAAATACCATAA

CCAAATGCAAGACAAAGTGCATCAGTGTCAAAATGAGACTGAATTACACATGAAACCGTCTCATTCAGCAACTCTTGAAG

GAGAGAAAAGGCCCGGGAGCCACTGATGCCCAGGTCAAGAGATTGTGTGCTCCGCGTTAGCCACTGGAAGGGCCTATTGA

GATTACCCAAAAAAAAAGA-GAACCAACC-GCCTAAAAGAAGGGAACCATGCCGCGATTAAGAAAGGTATGAACTATAAA

GTACCAACCAAATGCCTTTGAAAGGAAATCCACAGGGGAGGAGTCTA

35_Campylopterus_curvipennis_UG-10

GGATTATGGCTACTGCTACTTCTAGGATGGTTAGTAGGAGTAGTACGCAGGCGGTTAGAGCAGAGATTGCGGGTATGATG

GAGAATAGGACTGCGGTGGCTGTTGAGATGAGTTGGATTAGTAGGTGGCCTGCTGTTAGGTTAGCTGTGAGGCGGACTCC

TAGGGCTAGAGGGCGGATCAGGAGGCTGGTAGTTTCAATTATGATTAAGGCTGGGATTAGTGGAGTTGGGGTTCCTTCGG

GTAGGAGGTGGCCAAGTGAAGCGGATGGTTGGTTTCGGAGGCCTGTGAGTAGAGTGGCTAGTCATAGTGGGAAGGCTAGT

GCTAGGTTTATAGATAATTGTGTGGTAGGTGTGAATGTGTAGGGGAGTAGGCCTAGTAGGTTGATTGAGAGAAGGAAAAT

TATTAGTGATGACAGGATAAGGGCTCATTTGTGGCCTTTTTTGTTCAATGGGATTATCAGTTGTTTGGTGATTATATAGA

TGAATCACGATTGTAGGGTGGATGTTCGGTTGGTGATTCATCGGTTGTGGGGGGAGGGGAGTAGTAGGGCTGGGAATAAT

AGTGAGATGAGAATTAGGGGGATTCCTAGTAGGTGGGGGCTTATAAATTGGTCGAAGAAGCTTAGGTTCATGGTCAGGGT

CAGGATGAGGGTTTTGTGTTTGTTAGGGTTTTTTCTAGGGGGGAGTTAGTGGGCATGAATGAAAGGAGTTTGGGTTGGAT

GATTAGTGAAAAGGTCAGTCATGAAATAGTTATAATGAATAGTCATGGGTTTGGATTTAGTTGGGGCATATCATTAGGGA

GGGTTGGTAATCCTCTTTCTCTAGCTTAAAAGGCTAGTGCTGTTGCATAGCTTCCTAGTGGTTAAGATGATAGGAAAATT

AACATGATAAACATGATGATGGATGATGATTATTTTTTAATGTAATCTCGGTGTTGAATCTGGGTTAAAGTTAGATGATA

AAATGTGTGATGAAAATATCATGGATAGTATGGAAATTATAACTTAGAACTCCAGTGGTGTTGGTTAGAAAGTTAGAGGA

AGTGAAAAACTTGTAAAATATGTCCGGCAACCATAAATCAAAAAGCAACATAGCTTAGTAAGTATGTGAAAATACCATAA

CCAAATGCAAGACAAAGTGCATCAGTGTCAAAATGAGACTGAATTACACATGAAACCGTCTCATTCAGCAACTCTTGAAG

GAGAGAAAAGGCTCGGGAGCCACTGATGCCCAGGTCAAGAGATTGTGTGCTCCGCGTTAGCCACTGGAAGGGCCTATTGA

GATTACCCAAAAAAAAAGA-GAACCAACC-GCCTAAAAGAAGGGAACCATGCCGCGATTAAGAAAGGTATGAACTATAAA

GTACCAACCAAATGCCTTTGAAAGGAAATCCACAGGGGAGGAGTCTG

36_Campylopterus_curvipennis_UG-11

GGATTATGGCTACTGCTACTTCTAGGATGGTTAGTAGGAGTAGTACGCAGGCGGTTAGAGCAGAGATTGCGGGTATGATG

GAGAATAGGACTGCGGTGGCTGTTGAGATGAGTTGGATTAGTAGGTGGCCTGCTGTTAGGTTAGCTGTGAGGCGGACTCC

TAGGGCTAGAGGGCGGATCAGGAGGCTGGTAGTTTCAATTATGATTAAGGCTGGGATTAGTGGAGTTGGGGTTCCTTCGG

GTAGGAGGTGGCCAAGTGAAGCGGATGGTTGGTTTCGGAGGCCTGTGAGTAGAGTGGCTAGTCATAGTGGGAAGGCTAGT

GCTAGGTTTATAGATAATTGTGTGGTAGGTGTGAATGTGTAGGGGAGTAGGCCTAGTAGGTTGATTGAGAGAAGGAAAAT

TATTAGTGATGACAGGATAAGGGCTCATTTGTGGCCTTTTTTGTTCAATGGGATTATCAGTTGTTTGGTGATTATATAGA

TGAATCACGATTGTAGGGTGGATGTTCGGTTGGTGATTCATCGGTTGTGGGGGGAGGGGAGTAGTAGGGCTGGGAATAAT

AGTGAGATGAGAATTAGGGGGATTCCTAGTAGGTGGGGGCTTATAAATTGGTCGAAGAAGCTTAGGTTCATGGTCAGGGT

CAGGATGAGGGTTTTGTGTTTGTTAGGGTTTTTTCTAGGGGGGAGTTAGTGGGCATGAATGAAAGGAGTTTGGGTTGGAT

GATTAGTGAAAAGGTCAGTCATGAAATAGTTATAATGAATAGTCATGGGTTTGGATTTAGTTGGGGCATATCATTAGGGA

GGGTTGGTAATCCTCTTTCTCTAGCTTAAAAGGCTAGTGCTGTTGCATAGCTTCCTAGTGGTTAAGATGATAGGAAAATT

AACATGATAAACATGATGATGGATGATGATTATTTTTTAATGTAATCTCGGTGTTGAATCTGGGTTAAAGTTAGATGATA

AAATGTGTGATGAAAATATCATGGATAGTATGGAAATTATAACTTAGAACTCCAGTGGTGTTGGTTAGAAAGTTAGAGGA

AGTGAAAAACTTGTAAAATATGTCCGGCAACCATAAATCAAAAAGCAACATAGCTTAGTAAGTATGTGAAAATACCATAA

CCAAATGCAAGACAAAGTGCATCAGTGTCAAAATGAGACTGAATTACACATGAAACCGTCTCATTCAGCAACTCTTGAAG

GAGAGAAAAGGCTCGGGAGCCACTGATGCCCAGGTCAAGAGATTGTGTGCTCCGCGTTAGCCACTGGAAGGGCCTATTGA

GATTACCCAAAAAAAAAGA-GAACCAACC-GCCTAAAAGAAGGGAACCATGCCGCGATTAAGAAAGGTATGAACTATAAA

GTACCAACCAAATGCCTTTGAAAGGAAATCCACAGGGGAGGAGTCTG

37_Campylopterus_curvipennis_UG-12

GGATTATGGCTACTGCTACTTCTAGGATGGTTAGTAGGAGTAGTACGCAGGCGGTTAGAGCAGAGATTGCGGGTATGATG

GAGAATAGGACTGCGGTGGCTGTTGAGATGAGTTGGATTAGTAGGTGGCCTGCTGTTAGGTTAGCTGTGAGGCGGACTCC

TAGGGCTAGAGGGCGGATCAGGAGGCTGGTAGTTTCAATTATGATTAAGGCTGGGATTAGTGGAGTTGGGGTTCCTTCGG

GTAGGAGGTGGCCAAGTGAAGCGGATGGTTGGTTTCGGAGGCCTGTGAGTAGAGTGGCTAGTCATAGTGGGAAGGCTAGT

GCTAGGTTTATAGATAATTGTGTGGTAGGTGTGAATGTGTAGGGGAGTAGGCCTAGTAGGTTGATTGAGAGAAGGAAAAT

TATTAGTGATGACAGGATAAGGGCTCATTTGTGGCCTTTTTTGTTCAATGGGATTATCAGTTGTTTGGTGATTATATAGA

TGAATCACGATTGTAGGGTGGATGTTCGGTTGGTGATTCATCGGTTGTGGGGGGAGGGGAGTAGTAGGGCTGGGAATAAT

AGTGAGATGAGAATTAGGGGGATTCCTAGTAGGTGGGGGCTTATAAATTGGTCGAAGAAGCTTAGGTTCATGGTCAGGGT

CAGGATGAGGGTTTTGTGTTTGTTAGGGTTTTTTCTAGGGGGGAGTTAGTGGGCATGAATGAAAGGAGTTTGGGTTGGAT

GATTAGTGAAAAGGTCAGTCATGAAATAGTTATAATGAATAGTCATGGGTTTGGATTTAGTTGGGGCATATCATTAGGGA

GGGTTGGTAATCCTCTTTCTCTAGCTTAAAAGGCTAGTGCTGTTGCATAGCTTCCTAGTGGTTAAGATGATAGGAAAATT

AACATGATAAACATGATGATGGATGATGATTATTTTTTAATGTAATCTCGGTGTTGAATCTGGGTTAAAGTTAGATGATA

AAATGTGTGATGAAAATATCATGGATAGTATGGAAATTATAACTTAGAACTCCAGTGGTGTTGGTTAGAAAGTTAGAGGA

AGTGAAAAACTTGTAAAATATGTCCGGCAACCATAAATCAAAAAGCAACATAGCTTAGTAAGTATGTGAAAATACCATAA

CCAAATGCAAGACAAAGTGCATCAGTGTCAAAATGAGACTGAATTACACATGAAACCGTCTCATTCAGCAACTCTTGAAG

GAGAGAAAAGGCTCGGGAGCCACTGATGCCCAGGTCAAGAGATTGTGTGCTCCGCGTTAGCCACTGGAAGGGCCTATTGA

GATTACCCAAAAAAAAAGA-GAACCAACC-GCCTAAAAGAAGGGAACCATGCCGCGATTAAGAAAGGTATGAACTATAAA

GTACCAACCAAATGCCTTTGAAAGGAAATCCACAGGGGAGGAGTCTA

38_Campylopterus_curvipennis_UG-13

GGATTATGGCTACTGCTACTTCTAGGATGGTTAGTAGGAGTAGTACGCAGGCGGTTAGAGCAGAGATTGCGGGTATGATG

GAGAATAGGACTGCGGTGGCTGTTGAGATGAGTTGGATTAGTAGGTGGCCTGCTGTTAGGTTAGCTGTGAGGCGGACTCC

TAGGGCTAGAGGGCGGATCAGGAGGCTGGTAGTTTCAATTATGATTAAGGCTGGGATTAGTGGAGTTGGGGTTCCTTCGG

GTAGGAGGTGGCCAAGTGAAGCGGATGGTTGGTTTCGGAGGCCTGTGAGTAGAGTGGCTAGTCATAGTGGGAAGGCTAGT

GCTAGGTTTATAGATAATTGTGTGGTAGGTGTGAATGTGTAGGGGAGTAGGCCTAGTAGGTTGATTGAGAGAAGGAAAAT

TATTAGTGATGACAGGATAAGGGCTCATTTGTGGCCTTTTTTGTTCAATGGGATTATCAGTTGTTTGGTGATTATATAGA

TGAATCACGATTGTAGGGTGGATGTTCGGTTGGTGATTCATCGGTTGTGGGGGGAGGGGAGTAGTAGGGCTGGGAATAAT

AGTGAGATGAGAATTAGGGGGATTCCTAGTAGGTGGGGGCTTATAAATTGGTCGAAGAAGCTTAGGTTCATGGTCAGGGT

CAGGATGAGGGTTTTGTGTTTGTTAGGGTTTTTTCTAGGGGGGAGTTAGTGGGCATGAATGAAAGGAGTTTGGGTTGGAT

GATTAGTGAAAAGGTCAGTCATGAAATAGTTATAATGAATAGTCATGGGTTTGGATTTAGTTGGGGCATATCATTAGGGA

GGGTTGGTAATCCTCTTTCTCTAGCTTAAAAGGCTAGTGCTGTTGCATAGCTTCCTAGTGGTTAAGATGATAGGAAAATT

AACATGATAAACATGATGATGGATGATGATTATTTTTTAATGTAATCTCGGTGTTGAATCTGGGTTAAAGTTAGATGATA

AAATGTGTGATGAAAATATCATGGATAGTATGGAAATTATAACTTAGAACTCCAGTGGTGTTGGTTAGAAAGTTAGAGGA

AGTGAAAAACTTGTAAAATATGTCCGGCAACCATAAATCAAAAAGCAACATAGCTTAGTAAGTATGTGAAAATACCATAA

CCAAATGCAAGACAAAGTGCATCAGTGTCAAAATGAGACTGAATTACACATGAAACCGTCTCATTCAGCAACTCTTGAAG

GAGAGAAAAGGCTCGGGAGCCACTGATGCCCAGGTCAAGAGATTGTGTGCTCCGCGTTAGCCACTGGAAGGGCCTATTGA

GATTACCCAAAAAAAAAGA-GAACCAACC-GCCTAAAAGAAGGGAACCATGCCGCGATTAAGAAAGGTATGAACTATAAA

GTACCAACCAAATGCCTTTGAAAGGAAATCCACAGGGGAGGAGTCTG

39_Campylopterus_curvipennis_Ciel-1

GGATTATGGCTACTGCTACTTCTAGGATGGTTAGTAGGAGTAGTACGCAGGCGGTTAGAGCAGAGATTGCGGGTATGATG

GAGAATAGGACTGCGGTGGCTGTTGAGATGAGTTGGATTAGTAGGTGGCCTGCTGTTAGGTTAGCTGTGAGGCGGACTCC

TAGGGCTAGAGGGCGGATCAGGAGGCTGGTAGTTTCAATTATGATTAAGGCTGGGATTAGTGGAGTTGGGGTTCCTTCGG

GTAGGAGGTGGCCAAGTGAAGCGGATGGTTGGTTTCGGAGGCCTGTGAGTAGAGTGGCTAGTCATAGCGGGAAGGCTAGT

GCTAGGTTTATAGATAATTGTGTGGTAGGTGTGAATGTGTAGGGGAGTAGGCCTAGTAGGTTGATTGAGAGAAGGAAAAT

TATTAGTGATGACAGGATAAGGGCTCATTTGTGGCCTTTTTTGTTCAATGGGATTATCAGTTGTTTGGTGATTATATAGA

TGAATCACGATTGTAGGGTGGATGTTCGGTTGGTGATTCATCGGTTGTGGGGGGAGGGGAGTAGTAGGGCTGGGAATAAT

AGTGAGATGAGAATTAGGGGGATTCCTAGTAGGTGGGGGCTTATAAATTGGTCGAAGAAGCTTAGGTTCATGGTCAGGGT

CAGGATGAGGGTTTTGTGTTTGTTAGGGTTTTTTCTAGGGGGGAGTTAGTGGGCATGAATGAAAGGAGTTTGGGTTGGAT

GATTAGTGAAAAGGTCAGTCATGAAATAGTTATAATGAATAGTCATGGGTTTGGATTTAGTTGGGGCATATCATTAGGGA

GGGTTGGTAATCCTCTTTCTCTAGCTTAAAAGGCTAGTGCTGTTGCATAGCTTCCTAGTGGTTAAGATGATAGGAAAATT

AACATGATAAACATGATGATGGATGATGATTATTTTTTAATGTAATCTCGGTGTTGAATCTGGGTTAAAGTTAGATGATA

AAATGTGTGATGAAAATATCATGGATAGTATGGAAATTATAACTTAGAACTCCAGTGGTGTTGGTTAGAAAGTTAGAGGA

AGTGAAAAACTTGTAAAATATGTCCGGCAACCATAAATCAAAAAGCAACATAGCTTAGTAAGTATGTGAAAATACCATAA

CCAAATGCAAGACAAAGTGCATCAGTGTCAAAATGAGACTGAATTACACATGAAACCGTCTCATTCAGCAACTCTTGAAG

GAGAGAAAAGGCTCGGGAGCCACTGATGCCCAGGTCAAGAGATTGTGTGCTCCGCGTTAGCCACTGGAAGGGCCTATTGA

GATTACCCAAAAAAAAAGA-GAACCAACC-GCCTAAAAGAAGGGAACCATGCCGCGATTAAGAAAGGTATGAACTATAAA

GTACCAACCAAATGCCTTTGAAAGGAAATCCACAGGGGAGGAGTCTA

40_Campylopterus_curvipennis_Ciel-2

GGATTATGGCTACTGCTACTTCTAGGATGGTTAGTAGGAGTAGTACGCAGGCGGTTAGAGCAGAGATTGCGGGTATGATG

GAGAATAGGACTGCGGTGGCTGTTGAGATGAGTTGGATTAGTAGGTGGCCTGCTGTTAGGTTAGCTGTGAGGCGGACTCC

TAGGGCTAGAGGGCGGATCAGGAGGCTGGTAGTTTCAATTATGATTAAGGCTGGGATTAGTGGAGTTGGGGTTCCTTCGG

GTAGGAGGTGGCCAAGTGAAGCGGATGGTTGGTTTCGGAGGCCTGTGAGTAGAGTGGCTAGTCATAGTGGGAAGGCTAGT

GCTAGGTTTATAGATAATTGTGTGGTAGGTGTGAATGTGTAGGGGAGTAGGCCTAGTAGGTTGATTGAGAGAAGGAAAAT

TATTAGTGATGACAGGATAAGGGCTCATTTGTGGCCTTTTTTGTTCAATGGGATTATCAGTTGTTTGGTGATTATATAGA

TGAATCACGATTGTAGGGTGGATGTTCGGTTGGTGATTCATCGGTTGTGGGGGGAGGGGAGTAGTAGGGCTGGGAATAAT

AGTGAGATGAGAATTAGGGGGATTCCTAGTAGGTGGGGGCTTATAAATTGGTCGAAGAAGCTTAGGTTCATGGTCAGGGT

CAGGATGAGGGTTTTGTGTTTGTTAGGGGTTTTTCTAGGGGGGAGTTAGTGGGCATGAATGAAAGGAGTTTGGGTTGGAT

GATTAGTGAAAAGGTCAGTCATGAAATAGTTATAATGAATAGTCATGGGTTTGGATTTAGTTGGGGCATATCATTAGGGA

GGGTTGGTAATCCTCTTTCTCTAGCTTAAAAGGCTAGTGCTGTTGCATAGCTTCCTAGTGGTTAGGATGATAGGAAAATT

AACATGATAAACATGATGATGGATGATGATTATTTTTTAATGTAATCTCGGTGTTGAATCTGGGTTAAAGTTAGATGATA

AAATGTGTGATGAAAATATCATGGATAGTATGGAAATTATAACTTAGAACTCCAGTGGTGTTGGTTAGAAAGTTAGAGGA

AGTGAAAAACTTGTAAAATATGTCCGGCAACCATAAATCAAAAAGCAACATAGCTTAGTAAGTATGTGAAAATACCATAA

CCAAATGCAAGACAAAGTGCATCAGTGTCAAAATGAGACTGAATTACACATGAAACCGTCTCATTCAGCAACTCTTGAAG

GAGAGAAAAGGCCCGGGAGCCACTGATGCCCAGGTCAAGAGATTGTGTGCTCCGCGTTAGCCACTGGAAGGGCCTATTGA

GATTACCCAAAAAAAAAGA-GAACCAACC-GCCTAAAAGAAGGGAACCATGCCGCGATTAAGAAAGGTATGAACTATAAA

GTACCAACCAAATGCCTTTGAAAGGAAATCCACAGGGGAGGAATCTA

41_Campylopterus_curvipennis_Ciel-3

GGATTATGGCTACTGCTACTTCTAGGATGGTTAGTAGGAGTAGTACGCAGGCGGTTAGAGCAGAGATTGCGGGTATGATG

GAGAATAGGACTGCGGTGGCTGTTGAGATGAGTTGGATTAGTAGGTGGCCTGCTGTTAGGTTAGCTGTGAGGCGGACTCC

TAGGGCTAGAGGGCGGATCAGGAGGCTGGTAGTTTCAATTATGATTAAGGCTGGGATTAGTGGAGTTGGGGTTCCTTCGG

GTAGGAGGTGGCCAAGTGAAGCGGATGGTTGGTTTCGGAGGCCTGTGAGTAGAGTGGCTAGTCATAGTGGGAAGGCTAGT

GCTAGGTTTATAGATAATTGTGTGGTAGGTGTGAATGTGTAGGGGAGTAGGCCTAGTAGGTTGATTGAGAGAAGGAAAAT

TATTAGTGATGACAGGATAAGGGCTCATTTGTGGCCTTTTTTGTTCAATGGGATTATCAGTTGTTTGGTGATTATATAGA

TGAATCACGATTGTAGGGTGGATGTTCGGTTGGTGATTCATCGGTTGTGGGGGGAGGGGAGTAGTAGGGCTGGGAATAAT

AGTGAGATGAGAATTAGGGGGATTCCTAGTAGGTGGGGGCTTATAAATTGGTCGAAGAAGCTTAGGTTCATGGTCAGGGT

CAGGATGAGGGTTTTGTGTTTGTTAGGGTTTTTTCTAGGGGGGAGTTAGTGGGCATGAATGAAAGGAGTTTGGGTTGGAT

GATTAGTGAAAAGGTCAGTCATGAAATAGTTATAATGAATAGTCATGGGTTTGGATTTAGTTGGGGCATATCATTAGGGA

GGGTTGGTAATCCTCTTTCTCTAGCTTAAAAGGCTAGTGCTGTTGCATAGCTTCCTAGTGGTTAAGATGATAGGAAAATT

AACATGATAAACATGATGATGGATGATGATTATTTTTTAATGTAATCTCGGTGTTGAATCTGGGTTAAAGTTAGATGATA

AAATGTGTGATGAAAATATCATGGATAGTATGGAAATTATAACTTAGAACTCCAGTGGTGTTGGTTAGAAAGTTAGAGGA

AGTGAAAAACTTGTAAAATATGTCCGGCAACCATAAATCAAAAAGCAACATAGCTTAGTAAGTATGTGAAAATACCATAA

CCAAATGCAAGACAAAGTGCATCAGTGTCAAAATGAGACTGAATTACACATGAAACCGTCTCATTCAGCAACTCTTGAAG

GAGAGAAAAGGCTCGGGAGCCACTGATGCCCAGGTCAAGAGATTGTGTGCTCCGCGTTAGCCACTGGAAGGGCCTATTGA

GATTACCCAAAAAAAAAGA-GAACCAACC-GCCTAAAAGAAGGGAACCATGCCGCGATTAAGAAAGGTATGAACTATAAA

GTACCAACCAAATGCCTTTGAAAGGAAATCCACAGGGGAGGAGTCTA

42_Campylopterus_curvipennis_Ciel-4

GGATTATGGCTACTGCTACTTCTAGGATGGTTAGTAGGAGTAGTACGCAGGCGGTTAGAGCAGAGATTGCGGGTATGATG

GAGAATAGGACTGCGGTGGCTGTTGAGATGAGTTGGATTAGTAGGTGGCCTGCTGTTAGGTTAGCTGTGAGGCGGACTCC

TAGGGCTAGAGGGCGGATCAGGAGGCTGGTAGTTTCAATTATGATTAAGGCTGGGATTAGTGGAGTTGGGGTTCCTTCGG

GTAGGAGGTGGCCAAGTGAAGCGGATGGTTGGTTTCGGAGGCCTGTGAGTAGAGTGGCTAGTCATAGCGGGAAGGCTAGT

GCTAGGTTTATAGATAATTGTGTGGTAGGTGTGAATGTGTAGGGGAGTAGGCCTAGTAGGTTGATTGAGAGAAGGAAAAT

TATTAGTGATGACAGGATAAGGGCTCATTTGTGGCCTTTTTTGTTCAATGGGATTATCAGTTGTTTGGTGATTATATAGA

TGAATCACGATTGTAGGGTGGATGTTCGGTTGGTGATTCATCGGTTGTGGGGGGAGGGGAGTAGTAGGGCTGGGAATAAT

AGTGAGATGAGAATTAGGGGGATTCCTAGTAGGTGGGGGCTTATAAATTGGTCGAAGAAGCTTAGGTTCATGGTCAGGGT

CAGGATGAGGGTTTTGTGTTTGTTAGGGTTTTTTCTAGGGGGGAGTTAGTGGGCATGAATGAAAGGAGTTTGGGTTGGAT

GATTAGTGAAAAGGTCAGTCATGAAATAGTTATAATGAATAGTCATGGGTTTGGATTTAGTTGGGGCATATCATTAGGGA

GGGTTGGTAATCCTCTTTCTCTAGCTTAAAAGGCTAGTGCTGTTGCATAGCTTCCTAGTGGTTAAGATGATAGGAAAATT

AACATGATAAACATGATGATGGATGATGATTATTTTTTAATGTAATCTCGGTGTTGAATCTGGGTTAAAGTTAGATGATA

AAATGTGTGATGAAAATATCATGGATAGTATGGAAATTATAACTTAGAACTCCAGTGGTGTTGGTTAGAAAGTTAGAGGA

AGTGAAAAACTTGTAAAATATGTCCGGCAACCATAAATCAAAAAGCAACATAGCTTAGTAAGTATGTGAAAATACCATAA

CCAAATGCAAGACAAAGTGCATCAGTGTCAAAATGAGACTGAATTACACATGAAACCGTCTCATTCAGCAACTCTTGAAG

GAGAGAAAAGGCTCGGGAGCCACTGATGCCCAGGTCAAGAGATTGTGTGCTCCGCGTTAGCCACTGGAAGGGCCTATTGA

GATTACCCAAAAAAAAAGA-GAACCAACC-GCCTAAAAGAAGGGAACCATGCCGCGATTAAGAAAGGTATGAACTATAAA

GTACCAACCAAATGCCTTTGAAAGGAAATCCACAGGGGAGGAGTCTA

43_Campylopterus_curvipennis_Ciel-5

GGATTATGGCTACTGCTACTTCTAGGATGGTTAGTAGGAGTAGTACGCAGGCGGTTAGAGCAGAGATTGCGGGTATGATG

GAGAATAGGACTGCGGTGGCTGTTGAGATGAGTTGGATTAGTAGGTGGCCTGCTGTTAGGTTAGCTGTGAGGCGGACTCC

TAGGGCTAGAGGGCGGATCAGGAGGCTGGTAGTTTCAATTATGATTAAGGCTGGGATTAGTGGAGTTGGGGTTCCTTCGG

GTAGGAGGTGGCCAAGTGAAGCGGATGGTTGGTTTCGGAGGCCTGTGAGTAGAGTGGCTAGTCATAGCGGGAAGGCTAGT

GCTAGGTTTATAGATAATTGTGTGGTAGGTGTGAATGTGTAGGGGAGTAGGCCTAGTAGGTTGATTGAGAGAAGGAAAAT

TATTAGTGATGACAGGATAAGGGCTCATTTGTGGCCTTTTTTGTTCAATGGGATTATCAGTTGTTTGGTGATTATATAGA

TGAATCACGATTGTAGGGTGGATGTTCGGTTGGTGATTCATCGGTTGTGGGGGGAGGGGAGTAGTAGGGCTGGGAATAAT

AGTGAGATGAGAATTAGGGGGATTCCTAGTAGGTGGGGGCTTATAAATTGGTCGAAGAAGCTTAGGTTCATGGTCAGGGT

CAGGATGAGGGTTTTGTGTTTGTTAGGGTTTTTTCTAGGGGGGAGTTAGTGGGCATGAATGAAAGGAGTTTGGGTTGGAT

GATTAGTGAAAAGGTCAGTCATGAAATAGTTATAATGAATAGTCATGGGTTTGGATTTAGTTGGGGCATATCATTAGGGA

GGGTTGGTAATCCTCTTTCTCTAGCTTAAAAGGCTAGTGCTGTTGCATAGCTTCCTAGTGGTTAAGATGATAGGAAAATT

AACATGATAAACATGATGATGGATGATGATTATTTTTTAATGTAATCTCGGTGTTGAATCTGGGTTAAAGTTAGATGATA

AAATGTGTGATGAAAATATCATGGATAGTATGGAAATTATAACTTAGAACTCCAGTGGTGTTGGTTAGAAAGTTAGAGGA

AGTGAAAAACTTGTAAAATATGTCCGGCAACCATAAATCAAAAAGCAACATAGCTTAGTAAGTATGTGAAAATACCATAA

CCAAATGCAAGACAAAGTGCATCAGTGTCAAAATGAGACTGAATTACACATGAAACCGTCTCATTCAGCAACTCTTGAAG

GAGAGAAAAGGCTCGGGAGCCACTGATGCCCAGGTCAAGAGATTGTGTGCTCCGCGTTAGCCACTGGAAGGGCCTATTGA

GATTACCCAAAAAAAAAGA-GAACCAACC-GCCTAAAAGAAGGGAACCATGCCGCGATTAAGAAAGGTATGAACTATAAA

GTACCAACCAAATGCCTTTGAAAGGAAATCCACAGGGGAGGAGTCTA

44_Campylopterus_curvipennis_Ciel-6

GGATTATGGCTACTGCTACTTCTAGGATGGTTAGTAGGAGTAGTACGCAGGCGGTTAGAGCAGAGATTGCGGGTATGATG

GAGAATAGGACTGCGGTGGCTGTTGAGATGAGTTGGATTAGTAGGTGGCCTGCTGTTAGGTTAGCTGTGAGGCGGACTCC

TAGGGCTAGAGGGCGGATCAGGAGGCTGGTAGTTTCAATTATGATTAAGGCTGGGATTAGTGGAGTTGGGGTTCCTTCGG

GTAGGAGGTGGCCAAGTGAAGCGGATGGTTGGTTTCGGAGGCCTGTGAGTAGAGTGGCTAGTCATAGTGGGAAGGCTAGT

GCTAGGTTTATAGATAATTGTGTGGTAGGTGTGAATGTGTAGGGGAGTAGGCCTAGTAGGTTGATTGAGAGAAGGAAAAT

TATTAGTGATGACAGGATAAGGGCTCATTTGTGGCCTTTTTTGTTCAATGGGATTATCAGTTGTTTGGTGATTATATAGA

TGAATCACGATTGTAGGGTGGATGTTCGGTTGGTGATTCATCGGTTGTGGGGGGAGGGGAGTAGTAGGGCTGGGAATAAT

AGTGAGATGAGAATTAGGGGGATTCCTAGTAGGTGGGGGCTTATAAATTGGTCGAAGAAGCTTAGGTTCATGGTCAGGGT

CAGGATGAGGGTTTTGTGTTTGTTAGGGGTTTTTCTAGGGGGGAGTTAGTGGGCATGAATGAAAGGAGTTTGGGTTGGAT

GATTAGTGAAAAGGTCAGTCATGAAATAGTTATAATGAATAGTCATGGGTTTGGATTTAGTTGGGGCATATCATTAGGGA

GGGTTGGTAATCCTCTTTCTCTAGCTTAAAAGGCTAGTGCTGTTGCATAGCTTCCTAGTGGTTAGGATGATAGGAAAATT

AACATGATAAACATGATGATGGATGATGATTATTTTTTAATGTAATCTCGGTGTTGAATCTGGGTTAAAGTTAGATGATA

AAATGTGTGATGAAAATATCATGGATAGTATGGAAATTATAACTTAGAACTCCAGTGGTGTTGGTTAGAAAGTTAGAGGA

AGTGAAAAACTTGTAAAATATGTCCGGCAACCATAAATCAAAAAGCAACATAGCTTAGTAAGTATGTGAAAATACCATAA

CCAAATGCAAGACAAAGTGCATCAGTGTCAAAATGAGACTGAATTACACATGAAACCGTCTCATTCAGCAACTCTTGAAG

GAGAGAAAAGGCCCGGGAGCCACTGATGCCCAGGTCAAGAGATTGTGTGCTCCGCGTTAGCCACTGGAAGGGCCTATTGA

GATTACCCAAAAAAAAAGA-GAACCAACC-GCCTAAAAGAAGGGAACCATGCCGCGATTAAGAAAGGTATGAACTATAAA

GTACCAACCAAATGCCTTTGAAAGGAAATCCACAGGGGAGGAATCTA

45_Campylopterus_curvipennis_Ciel-7

GGATTATGGCTACTGCTACTTCTAGGATGGTTAGTAGGAGTAGTACGCAGGCGGTTAGAGCAGAGATTGCGGGTATGATG

GAGAATAGGACTGCGGTGGCTGTTGAGATGAGTTGGATTAGTAGGTGGCCTGCTGTTAGGTTAGCTGTGAGGCGGACTCC

TAGGGCTAGAGGGCGGATCAGGAGGCTGGTAGTTTCAATTATGATTAAGGCTGGGATTAGTGGAGTTGGGGTTCCTTCGG

GTAGGAGGTGGCCAAGTGAAGCGGATGGTTGGTTTCGGAGGCCTGTGAGTAGAGTGGCTAGTCATAGTGGGAAGGCTAGT

GCTAGGTTTATAGATAATTGTGTGGTAGGTGTGAATGTGTAGGGGAGTAGGCCTAGTAGGTTGATTGAGAGAAGGAAAAT

TATTAGTGATGACAGGATAAGGGCTCATTTGTGGCCTTTTTTGTTCAATGGGATTATCAGTTGTTTGGTGATTATATAGA

TGAATCACGATTGTAGGGTGGATGTTCGGTTGGTGATTCATCGGTTGTGGGGGGAGGGGAGTAGTAGGGCTGGGAATAAT

AGTGAGATGAGAATTAGGGGGATTCCTAGTAGGTGGGGGCTTATAAATTGGTCGAAGAAGCTTAGGTTCATGGTCAGGGT

CAGGATGAGGGTTTTGTGTTTGTTAGGGGTTTTTCTAGGGGGGAGTTAGTGGGCATGAATGAAAGGAGTTTGGGTTGGAT

GATTAGTGAAAAGGTCAGTCATGAAATAGTTATAATGAATAGTCATGGGTTTGGATTTAGTTGGGGCATATCATTAGGGA

GGGTTGGTAATCCTCTTTCTCTAGCTTAAAAGGCTAGTGCTGTTGCATAGCTTCCTAGTGGTTAGGATGATAGGAAAATT

AACATGATAAACATGATGATGGATGATGATTATTTTTTAATGTAATCTCGGTGTTGAATCTGGGTTAAAGTTAGATGATA

AAATGTGTGATGAAAATATCATGGATAGTATGGAAATTATAACTTAGAACTCCAGTGGTGTTGGTTAGAAAGTTAGAGGA

AGTGAAAAACTTGTAAAATATGTCCGGCAACCATAAATCAAAAAGCAACATAGCTTAGTAAGTATGTGAAAATACCATAA

CCAAATGCAAGACAAAGTGCATCAGTGTCAAAATGAGACTGAATTACACATGAAACCGTCTCATTCAGCAACTCTTGAAG

GAGAGAAAAGGCCCGGGAGCCACTGATGCCCAGGTCAAGAGATTGTGTGCTCCGCGTTAGCCACTGGAAGGGCCTATTGA

GATTACCCAAAAAAAAAGA-GAACCAACC-GCCTAAAAGAAGGGAACCATGCCGCGATTAAGAAAGGTATGAACTATAAA

GTACCAACCAAATGCCTTTGAAAGGAAATCCACAGGGGAGGAATCTA

46_Campylopterus_curvipennis_Ciel-8

GGATTATGGCTACTGCTACTTCTAGGATGGTTAGTAGGAGTAGTACGCAGGCGGTTAGAGCAGAGATTGCGGGTATGATG

GAGAATAGGACTGCGGTGGCTGTTGAGATGAGTTGGATTAGTAGGTGGCCTGCTGTTAGGTTAGCTGTGAGGCGGACTCC

TAGGGCTAGAGGGCGGATCAGGAGGCTGGTAGTTTCAATTATGATTAAGGCTGGGATTAGTGGAGTTGGGGTTCCTTCGG

GTAGGAGGTGGCCAAGTGAAGCGGATGGTTGGTTTCGGAGGCCTGTGAGTAGAGTGGCTAGTCATAGTGGGAAGGCTAGT

GCTAGGTTTATAGATAATTGTGTGGTAGGTGTGAATGTGTAGGGGAGTAGGCCTAGTAGGTTGATTGAGAGAAGGAAAAT

TATTAGTGATGACAGGATAAGGGCTCATTTGTGGCCTTTTTTGTTCAATGGGATTATCAGTTGTTTGGTGATTATATAGA

TGAATCACGATTGTAGGGTGGATGTTCGGTTGGTGATTCATCGGTTGTGGGGGGAGGGGAGTAGTAGGGCTGGGAATAAT

AGTGAGATGAGAATTAGGGGGATTCCTAGTAGGTGGGGGCTTATAAATTGGTCGAAGAAGCTTAGGTTCATGGTCAGGGT

CAGGATGAGGGTTTTGTGTTTGTTAGGGTTTTTTCTAGGGGGGAGTTAGTGGGCATGAATGAAAGGAGTTTGGGTTGGAT

GATTAGTGAAAAGGTCAGTCATGAAATAGTTATAATGAATAGTCATGGGTTTGGATTTAGTTGGGGCATATCATTAGGGA

GGGTTGGTAATCCTCTTTCTCTAGCTTAAAAGGCTAGTGCTGTTGCATAGCTTCCTAGTGGTTAAGATGATAGGAAAATT

AACATGATAAACATGATGATGGATGATGATTATTTTTTAATGTAATCTCGGTGTTGAATCTGGGTTAAAGTTAGATGATA

AAATGTGTGATGAAAATATCATGGATAGTATGGAAATTATAACTTAGAACTCCAGTGGTGTTGGTTAGAAAGTTAGAGGA

AGTGAAAAACTTGTAAAATATGTCCGGCAACCATAAATCAAAAAGCAACATAGCTTAGTAAGTATGTGAAAATACCATAA

CCAAATGCAAGACAAAGTGCATCAGTGTCAAAATGAGACTGAATTACACATGAAACCGTCTCATTCAGCAACTCTTGAAG

GAGAGAAAAGGCTCGGGAGCCACTGATGCCCAGGTCAAGAGATTGTGTGCTCCGCGTTAGCCACTGGAAGGGCCTATTGA

GATTACCCAAAAAAAAAGA-GAACCAACC-GCCTAAAAGAAGGGAACCATGCCGCGATTAAGAAAGGTATGAACTATAAA

GTACCAACCAAATGCCTTTGAAAGGAAATCCACAGGGGAGGAGTCTA

47_Campylopterus_curvipennis_Ciel-9

GGATTATGGCTACTGCTACTTCTAGGATGGTTAGTAGGAGTAGTACGCAGGCGGTTAGAGCAGAGATTGCGGGTATGATG

GAGAATAGGACTGCGGTGGCTGTTGAGATGAGTTGGATTAGTAGGTGGCCTGCTGTTAGGTTAGCTGTGAGGCGGACTCC

TAGGGCTAGAGGGCGGATCAGGAGGCTGGTAGTTTCAATTATGATTAAGGCTGGGATTAGTGGAGTTGGGGTTCCTTCGG

GTAGGAGGTGGCCAAGTGAAGCGGATGGTTGGTTTCGGAGGCCTGTGAGTAGAGTGGCTAGTCATAGTGGGAAGGCTAGT

GCTAGGTTTATAGATAATTGTGTGGTAGGTGTGAATGTGTAGGGGAGTAGGCCTAGTAGGTTGATTGAGAGAAGGAAAAT

TATTAGTGATGACAGGATAAGGGCTCATTTGTGGCCTTTTTTGTTCAATGGGATTATCAGTTGTTTGGTGATTATATAGA

TGAATCACGATTGTAGGGTGGATGTTCGGTTGGTGATTCATCGGTTGTGGGGGGAGGGGAGTAGTAGGGCTGGGAATAAT

AGTGAGATGAGAATTAGGGGGATTCCTAGTAGGTGGGGGCTTATAAATTGGTCGAAGAAGCTTAGGTTCATGGTCAGGGT

CAGGATGAGGGTTTTGTGTTTGTTAGGGTTTTTTCTAGGGGGGAGTTAGTGGGCATGAATGAAAGGAGTTTGGGTTGGAT

GATTAGTGAAAAGGTCAGTCATGAAATAGTTATAATGAATAGTCATGGGTTTGGATTTAGTTGGGGCATATCATTAGGGA

GGGTTGGTAATCCTCTTTCTCTAGCTTAAAAGGCTAGTGCTGTTGCATAGCTTCCTAGTGGTTAAGATGATAGGAAAATT

AACATGATAAACATGATGATGGATGATGATTATTTTTTAATGTAATCTCGGTGTTGAATCTGGGTTAAAGTTAGATGATA

AAATGTGTGATGAAAATATCATGGATAGTATGGAAATTATAACTTAGAACTCCAGTGGTGTTGGTTAGAAAGTTAGAGGA

AGTGAAAAACTTGTAAAATATGTCCGGCAACCATAAATCAAAAAGCAACATAGCTTAGTAAGTATGTGAAAATACCATAA

CCAAATGCAAGACAAAGTGCATCAGTGTCAAAATGAGACTGAATTACACATGAAACCGTCTCATTCAGCAACTCTTGAAG

GAGAGAAAAGGCTCGGGGGCCACTGATGCCCAGGTCAAGAGATTGTGTGCTCCGCGTTAGCCACTGGAAGGGCCTATTGA

GATTACCCAAAAAAAAAGA-GAACCAACC-GCCTAAAAGAAGGGAACCATGCCGCGATTAAGAAAGGTATGAACTATAAA

GTACCAACCAAATGCCTTTGAAAGGAAATCCACAGGGGAGGAGTCTA

48_Campylopterus_curvipennis_Ciel-10

GGATTATGGCTACTGCTACTTCTAGGATGGTTAGTAGGAGTAGTACGCAGGCGGTTAGAGCAGAGATTGCGGGTATGATG

GAGAATAGGACTGCGGTGGCTGTTGAGATGAGTTGGATTAGTAGGTGGCCTGCTGTTAGGTTAGCTGTGAGGCGGACTCC

TAGGGCTAGAGGGCGGATCAGGAGGCTGGTAGTTTCAATTATGATTAAGGCTGGGATTAGTGGAGTTGGGGTTCCTTCGG

GTAGGAGGTGGCCAAGTGAAGCGGATGGTTGGTTTCGGAGGCCTGTGAGTAGAGTGGCTAGTCATAGCGGGAAGGCTAGT

GCTAGGTTTATAGATAATTGTGTGGTAGGTGTGAATGTGTAGGGGAGTAGGCCTAGTAGGTTGATTGAGAGAAGGAAAAT

TATTAGTGATGACAGGATAAGGGCTCATTTGTGGCCTTTTTTGTTCAATGGGATTATCAGTTGTTTGGTGATTATATAGA

TGAATCACGATTGTAGGGTGGATGTTCGGTTGGTGATTCATCGGTTGTGGGGGGAGGGGAGTAGTAGGGCTGGGAATAAT

AGTGAGATGAGAATTAGGGGGATTCCTAGTAGGTGGGGGCTTATAAATTGGTCGAAGAAGCTTAGGTTCATGGTCAGGGT

CAGGATGAGGGTTTTGTGTTTGTTAGGGTTTTTTCTAGGGGGGAGTTAGTGGGCATGAATGAAAGGAGTTTGGGTTGGAT

GATTAGTGAAAAGGTCAGTCATGAAATAGTTATAATGAATAGTCATGGGTTTGGATTTAGTTGGGGCATATCATTAGGGA

GGGTTGGTAATCCTCTTTCTCTAGCTTAAAAGGCTAGTGCTGTTGCATAGCTTCCTAGTGGTTAAGATGATAGGAAAATT

AACATGATAAACATGATGATGGATGATGATTATTTTTTAATGTAATCTCGGTGTTGAATCTGGGTTAAAGTTAGATGATA

AAATGTGTGATGAAAATATCATGGATAGTATGGAAATTATAACTTAGAACTCCAGTGGTGTTGGTTAGAAAGTTAGAGGA

AGTGAAAAACTTGTAAAATATGTCCGGCAACCATAAATCAAAAAGCAACATAGCTTAGTAAGTATGTGAAAATACCATAA

CCAAATGCAAGACAAAGTGCATCAGTGTCAAAATGAGACTGAATTACACATGAAACCGTCTCATTCAGCAACTCTTGAAG

GAGAGAAAAGGCTCGGGAGCCACTGATGCCCAGGTCAAGAGATTGTGTGCTCCGCGTTAGCCACTGGAAGGGCCTATTGA

GATTACCCAAAAAAAAAGA-GAACCAACC-GCCTAAAAGAAGGGAACCATGCCGCGATTAAGAAAGGTATGAACTATAAA

GTACCAACCAAATGCCTTTGAAAGGAAATCCACAGGGGAGGAGTCTA

49_Campylopterus_curvipennis_Ciel-11

GGATTATGGCTACTGCTACTTCTAGGATGGTTAGTAGGAGTAGTACGCAGGCGGTTAGAGCAGAGATTGCGGGTATGATG

GAGAATAGGACTGCGGTGGCTGTTGAGATGAGTTGGATTAGTAGGTGGCCTGCTGTTAGGTTAGCTGTGAGGCGGACTCC

TAGGGCTAGAGGGCGGATCAGGAGGCTGGTAGTTTCAATTATGATTAAGGCTGGGATTAGTGGAGTTGGGGTTCCTTCGG

GTAGGAGGTGGCCAAGTGAAGCGGATGGTTGGTTTCGGAGGCCTGTGAGTAGAGTGGCTAGTCATAGTGGGAAGGCTAGT

GCTAGGTTTATAGATAATTGTGTGGTAGGTGTGAATGTGTAGGGGAGTAGGCCTAGTAGGTTGATTGAGAGAAGGAAAAT

TATTAGTGATGACAGGATAAGGGCTCATTTGTGGCCTTTTTTGTTCAATGGGATTATCAGTTGTTTGGTGATTATATAGA

TGAATCACGATTGTAGGGTGGATGTTCGGTTGGTGATTCATCGGTTGTGGGGGGAGGGGAGTAGTAGGGCTGGGAATAAT

AGTGAGATGAGAATTAGGGGGATTCCTAGTAGGTGGGGGCTTATAAATTGGTCGAAGAAGCTTAGGTTCATGGTCAGGGT

CAGGATGAGGGTTTTGTGTTTGTTAGGGGTTTTTCTAGGGGGGAGTTAGTGGGCATGAATGAAAGGAGTTTGGGTTGGAT

GATTAGTGAAAAGGTCAGTCATGAAATAGTTATAATGAATAGTCATGGGTTTGGATTTAGTTGGGGCATATCATTAGGGA

GGGTTGGTAATCCTCTTTCTCTAGCTTAAAAGGCTAGTGCTGTTGCATAGCTTCCTAGTGGTTAGGATGATAGGAAAATT

AACATGATAAACATGATGATGGATGATGATTATTTTTTAATGTAATCTCGGTGTTGAATCTGGGTTAAAGTTAGATGATA

AAATGTGTGATGAAAATATCATGGATAGTATGGAAATTATAACTTAGAACTCCAGTGGTGTTGGTTAGAAAGTTAGAGGA

AGTGAAAAACTTGTAAAATATGTCCGGCAACCATAAATCAAAAAGCAACATAGCTTAGTAAGTATGTGAAAATACCATAA

CCAAATGCAAGACAAAGTGCATCAGTGTCAAAATGAGACTGAGTTACACATGAAACCGTCTCATTCAGCAACTCTTGAAG

GAGAGAAAAGGCCCGGGAGCCACTGATGCCCAGGTCAAGAGATTGTGTGCTCCGCGTTAGCCACTGGAAGGGCCTATTGA

GATTACCCAAAAAAAAAGA-GAACCAACC-GCCTAAAAGAAGGGAACCATGCCGCGATTAAGAAAGGTATGAACTATAAA

GTACCAACCAAATGCCTTTGAAAGGAAATCCACAGGGGAGGAATCTA

50_Campylopterus_curvipennis_Ciel-12

GGATTATGGCTACTGCTACTTCTAGGATGGTTAGTAGGAGTAGTACGCAGGCGGTTAGAGCAGAGATTGCGGGTATGATG

GAGAATAGGACTGCGGTGGCTGTTGAGATGAGTTGGATTAGTAGGTGGCCTGCTGTTAGGTTAGCTGTGAGGCGGACTCC

TAGGGCTAGAGGGCGGATCAGGAGGCTGGTAGTTTCAATTATGATTAAGGCTGGGATTAGTGGAGTTGGGGTTCCTTCGG

GTAGGAGGTGGCCAAGTGAAGCGGATGGTTGGTTTCGGAGGCCTGTGAGTAGAGTGGCTAGTCATAGTGGGAAGGCTAGT

GCTAGGTTTATAGATAATTGTGTGGTAGGTGTGAATGTGTAGGGGAGTAGGCCTAGTAGGTTGATTGAGAGAAGGAAAAT

TATTAGTGATGACAGGATAAGGGCTCATTTGTGGCCTTTTTTGTTCAATGGGATTATCAGTTGTTTGGTGATTATATAGA

TGAATCACGATTGTAGGGTGGATGTTCGGTTGGTGATTCATCGGTTGTGGGGGGAGGGGAGTAGTAGGGCTGGGAATAAT

AGTGAGATGAGAATTAGGGGGATTCCTAGTAGGTGGGGGCTTATAAATTGGTCGAAGAAGCTTAGGTTCATGGTCAGGGT

CAGGATGAGGGTTTTGTGTTTGTTAGGGGTTTTTCTAGGGGGGAGTTAGTGGGCATGAATGAAAGGAGTTTGGGTTGGAT

GATTAGTGAAAAGGTCAGTCATGAAATAGTTATAATGAATAGTCATGGGTTTGGATTTAGTTGGGGCATATCATTAGGGA

GGGTTGGTAATCCTCTTTCTCTAGCTTAAAAGGCTAGTGCTGTTGCATAGCTTCCTAGTGGTTAGGATGATAGGAAAATT

AACATGATAAACATGATGATGGATGATGATTATTTTTTAATGTAATCTCGGTGTTGAATCTGGGTTAAAGTTAGATGATA

AAATGTGTGATGAAAATATCATGGATAGTATGGAAATTATAACTTAGAACTCCAGTGGTGTTGGTTAGAAAGTTAGAGGA

AGTGAAAAACTTGTAAAATATGTCCGGCAACCATAAATCAAAAAGCAACATAGCTTAGTAAGTATGTGAAAATACCATAA

CCAAATGCAAGACAAAGTGCATCAGTGTCAAAATGAGACTGAGTTACACATGAAACCGTCTCATTCAGCAACTCTTGAAG

GAGAGAAAAGGCCCGGGAGCCACTGATGCCCAGGTCAAGAGATTGTGTGCTCCGCGTTAGCCACTGGAAGGGCCTATTGA

GATTACCCAAAAAAAAAGA-GAACCAACC-GCCTAAAAGAAGGGAACCATGCCGCGATTAAGAAAGGTATGAACTATAAA

GTACCAACCAAATGCCTTTGAAAGGAAATCCACAGGGGAGGAATCTA

51_Campylopterus_curvipennis_Ciel-13

GGATTATGGCTACTGCTACTTCTAGGATGGTTAGTAGGAGTAGTACGCAGGCGGTTAGAGCAGAGATTGCGGGTATGATG

GAGAATAGGACTGCGGTGGCTGTTGAGATGAGTTGGATTAGTAGGTGGCCTGCTGTTAGGTTAGCTGTGAGGCGGACTCC

TAGGGCTAGAGGGCGGATCAGGAGGCTGGTAGTTTCAATTATGATTAAGGCTGGGATTAGTGGAGTTGGGGTTCCTTCGG

GTAGGAGGTGGCCAAGTGAAGCGGATGGTTGGTTTCGGAGGCCTGTGAGTAGAGTGGCTAGTCATAGTGGGAAGGCTAGT

GCTAGGTTTATAGATAATTGTGTGGTAGGTGTGAATGTGTAGGGGAGTAGGCCTAGTAGGTTGATTGAGAGAAGGAAAAT

TATTAGTGATGACAGGATAAGGGCTCATTTGTGGCCTTTTTTGTTCAATGGGATTATCAGTTGTTTGGTGATTATATAGA

TGAATCACGATTGTAGGGTGGATGTTCGGTTGGTGATTCATCGGTTGTGGGGGGAGGGGAGTAGTAGGGCTGGGAATAAT

AGTGAGATGAGAATTAGGGGGATTCCTAGTAGGTGGGGGCTTATAAATTGGTCGAAGAAGCTTAGGTTCATGGTCAGGGT

CAGGATGAGGGTTTTGTGTTTGTTAGGGTTTTTTCTAGGGGGGAGTTAGTGGGCATGAATGAAAGGAGTTTGGGTTGGAT

GATTAGTGAAAAGGTCAGTCATGAAATAGTTATAATGAATAGTCATGGGTTTGGATTTAGTTGGGGCATATCATTAGGGA

GGGTTGGTAATCCTCTTTCTCTAGCTTAAAAGGCTAGTGCTGTTGCATAGCTTCCTAGTGGTTAAGATGATAGGAAAATT

AACATGATAAACATGATGATGGATGATGATTATTTTTTAATGTAATCTCGGTGTTGAATCTGGGTTAAAGTTAGATGATA

AAATGTGTGATGAAAATATCATGGATAGTATGGAAATTATAACTTAGAACTCCAGTGGTGTTGGTTAGAAAGTTAGAGGA

AGTGAAAAACTTGTAAAATATGTCCGGCAACCATAAATCAAAAAGCAACATAGCTTAGTAAGTATGTGAAAATACCATAA

CCAAATGCAAGACAAAGTGCATCAGTGTCAAAATGAGACTGAATTACACATGAAACCGTCTCATTCAGCAACTCTTGAAG

GAGAGAAAAGGCTCGGGAGCCACTGATGCCCAGGTCAAGAGATTGTGTGCTCCGCGTTAGCCACTGGAAGGGCCTATTGA

GATTACCCAAAAAAAAAGA-GAACCAACC-GCCTAAAAGAAGGGAACCATGCCGCGATTAAGAAAGGTATGAACTATAAA

GTACCAACCAAATGCCTTTGAAAGGAAATCCACAGGGGAGGAGTCTA

52_Campylopterus_curvipennis_Ciel-14

GGATTATGGCTACTGCTACTTCTAGGATGGTTAGTAGGAGTAGTACGCAGGCGGTTAGAGCAGAGATTGCGGGTATGATG

GAGAATAGGACTGCGGTGGCTGTTGAGATGAGTTGGATTAGTAGGTGGCCTGCTGTTAGGTTAGCTGTGAGGCGGACTCC

TAGGGCTAGAGGGCGGATCAGGAGGCTGGTAGTTTCAATTATGATTAAGGCTGGGATTAGTGGAGTTGGGGTTCCTTCGG

GTAGGAGGTGGCCAAGTGAAGCGGATGGTTGGTTTCGGAGGCCTGTGAGTAGAGTGGCTAGTCATAGCGGGAAGGCTAGT

GCTAGGTTTATAGATAATTGTGTGGTAGGTGTGAATGTGTAGGGGAGTAGGCCTAGTAGGTTGATTGAGAGAAGGAAAAT

TATTAGTGATGACAGGATAAGGGCTCATTTGTGGCCTTTTTTGTTCAATGGGATTATCAGTTGTTTGGTGATTATATAGA

TGAATCACGATTGTAGGGTGGATGTTCGGTTGGTGATTCATCGGTTGTGGGGGGAGGGGAGTAGTAGGGCTGGGAATAAT

AGTGAGATGAGAATTAGGGGGATTCCTAGTAGGTGGGGGCTTATAAATTGGTCGAAGAAGCTTAGGTTCATGGTCAGGGT

CAGGATGAGGGTTTTGTGTTTGTTAGGGTTTTTTCTAGGGGGGAGTTAGTGGGCATGAATGAAAGGAGTTTGGGTTGGAT

GATTAGTGAAAAGGTCAGTCATGAAATAGTTATAATGAATAGTCATGGGTTTGGATTTAGTTGGGGCATATCATTAGGGA

GGGTTGGTAATCCTCTTTCTCTAGCTTAAAAGGCTAGTGCTGTTGCATAGCTTCCTAGTGGTTAAGATGATAGGAAAATT

AACATGATAAACATGATGATGGATGATGATTATTTTTTAATGTAATCTCGGTGTTGAATCTGGGTTAAAGTTAGATGATA

AAATGTGTGATGAAAATATCATGGATAGTATGGAAATTATAACTTAGAACTCCAGTGGTGTTGGTTAGAAAGTTAGAGGA

AGTGAAAAACTTGTAAAATATGTCCGGCAACCATAAATCAAAAAGCAACATAGCTTAGTAAGTATGTGAAAATACCATAA

CCAAATGCAAGACAAAGTGCATCAGTGTCAAAATGAGACTGAATTACACATGAAACCGTCTCATTCAGCAACTCTTGAAG

GAGAGAAAAGGCTCGGGAGCCACTGATGCCCAGGTCAAGAGATTGTGTGCTCCGCGTTAGCCACTGGAAGGGCCTATTGA

GATTACCCAAAAAAAAAGA-GAACCAACC-GCCTAAAAGAAGGGAACCATGCCGCGATTAAGAAAGGTATGAACTATAAA

GTACCAACCAAATGCCTTTGAAAGGAAATCCACAGGGGAGGAGTCTA

53_Campylopterus_curvipennis_Ciel-15

GGATTATGGCTACTGCTACTTCTAGGATGGTTAGTAGGAGTAGTACGCAGGCGGTTAGAGCAGAGATTGCGGGTATGATG

GAGAATAGGACTGCGGTGGCTGTTGAGATGAGTTGGATTAGTAGGTGGCCTGCTGTTAGGTTAGCTGTGAGGCGGACTCC

TAGGGCTAGAGGGCGGATCAGGAGGCTGGTAGTTTCAATTATGATTAAGGCTGGGATTAGTGGAGTTGGGGTTCCTTCGG

GTAGGAGGTGGCCAAGTGAAGCGGATGGTTGGTTTCGGAGGCCTGTGAGTAGAGTGGCTAGTCATAGTGGGAAGGCTAGT

GCTAGGTTTATAGATAATTGTGTGGTAGGTGTGAATGTGTAGGGGAGTAGGCCTAGTAGGTTGATTGAGAGAAGGAAAAT

TATTAGTGATGACAGGATAAGGGCTCATTTGTGGCCTTTTTTGTTCAATGGGATTATCAGTTGTTTGGTGATTATATAGA

TGAATCACGATTGTAGGGTGGATGTTCGGTTGGTGATTCATCGGTTGTGGGGGGAGGGGAGTAGTAGGGCTGGGAATAAT

AGTGAGATGAGAATTAGGGGGATTCCTAGTAGGTGGGGGCTTATAAATTGGTCGAAGAAGCTTAGGTTCATGGTCAGGGT

CAGGATGAGGGTTTTGTGTTTGTTAGGGGTTTTTCTAGGGGGGAGTTAGTGGGCATGAATGAAAGGAGTTTGGGTTGGAT

GATTAGTGAAAAGGTCAGTCATGAAATAGTTATAATGAATAGTCATGGGTTTGGATTTAGTTGGGGCATATCATTAGGGA

GGGTTGGTAATCCTCTTTCTCTAGCTTAAAAGGCTAGTGCTGTTGCATAGCTTCCTAGTGGTTAGGATGATAGGAAAATT

AACATGATAAACATGATGATGGATGATGATTATTTTTTAATGTAATCTCGGTGTTGAATCTGGGTTAAAGTTAGATGATA

AAATGTGTGATGAAAATATCATGGATAGTATGGAAATTATAACTTAGAACTCCAGTGGTGTTGGTTAGAAAGTTAGAGGA

AGTGAAAAACTTGTAAAATATGTCCGGCAACCATAAATCAAAAAGCAACATAGCTTAGTAAGTATGTGAAAATACCATAA

CCAAATGCAAGACAAAGTGCATCAGTGTCAAAATGAGACTGAATTACACATGAAACCGTCTCATTCAGCAACTCTTGAAG

GAGAGAAAAGGCCCGGGAGCCACTGATGCCCAGGTCAAGAGATTGTGTGCTCCGCGTTAGCCACTGGAAGGGCCTATTGA

GATTACCCAAAAAAAAAGA-GAACCAACC-GCCTAAAAGAAGGGAACCATGCCGCGATTAAGAAAGGTATGAACTATAAA

GTACCAACCAAATGCCTTTGAAAGGAAATCCACAGGGGAGGAATCTA

54_Campylopterus_curvipennis_Ciel-16

GGATTATGGCTACTGCTACTTCTAGGATGGTTAGTAGGAGTAGTACGCAGGCGGTTAGAGCAGAGATTGCGGGTATGATG

GAGAATAGGACTGCGGTGGCTGTTGAGATGAGTTGGATTAGTAGGTGGCCTGCTGTTAGGTTAGCTGTGAGGCGGACTCC

TAGGGCTAGAGGGCGGATCAGGAGGCTGGTAGTTTCAATTATGATTAAGGCTGGGATTAGTGGAGTTGGGGTTCCTTCGG

GTAGGAGGTGGCCAAGTGAAGCGGATGGTTGGTTTCGGAGGCCTGTGAGTAGAGTGGCTAGTCATAGTGGGAAGGCTAGT

GCTAGGTTTATAGATAATTGTGTGGTAGGTGTGAATGTGTAGGGGAGTAGGCCTAGTAGGTTGATTGAGAGAAGGAAAAT

TATTAGTGATGACAGGATAAGGGCTCATTTGTGGCCTTTTTTGTTCAATGGGATTATCAGTTGTTTGGTGATTATATAGA

TGAATCACGATTGTAGGGTGGATGTTCGGTTGGTGATTCATCGGTTGTGGGGGGAGGGGAGTAGTAGGGCTGGGAATAAT

AGTGAGATGAGAATTAGGGGGATTCCTAGTAGGTGGGGGCTTATAAATTGGTCGAAGAAGCTTAGGTTCATGGTCAGGGT

CAGGATGAGGGTTTTGTGTTTGTTAGGGGGTTTTCTAGGGGGGAGTTAGTGGGCATGAATGAAAGGAGTTTGGGTTGGAT

GATTAGTGAAAAGGTCAGTCATGAAATAGTTATAATGAATAGTCATGGGTTTGGATTTAGTTGGGGCATATCATTAGGGA

GGGTTGGTAATCCTCTTTCTCTAGCTTAAAAGGCTAGTGCTGTTGCATAGCTTCCTAGTGGTTAAGATGATAGGAAAATT

AACATGATAAACATGATGATGGATGATGATTATTTTTTAATGTAATCTCGGTGTTGAATCTGGGTTAAAGTTAGATGATA

AAATGTGTGATGAAAATATCATGGATAGTATGGAAATTATAACTTAGAACTCCAGTGGTGTTGGTTAGAAAGTTAGAGGA

AGTGAAAAACTTGTAAAATATGTCCGGCAACCATAAATCAAAAAGCAACATAGCTTAGTAAGTATGTGAAAATACCATAA

CCAAATGCAAGACAAAGTGCATCAGTGTCAAAATGAGACTGAATTACACATGAAACCGTCTCATTCAGCAACTCTTGAAG

GAGAGAAAAGGCTCGGGAGCCACTGATGCCCAGGTCAAGAGATTGTGTGCTCCGCGTTAGCCACTGGAAGGGCCTATTGA

GATTACCCAAAAAAAAAGA-GAACCAACC-GCCTAAAAGAAGGGAACCATGCCGCGATTAAGAAAGGTATGAACTATAAA

GTACCAACCAAATGCCTTTGAAAGGAAATTCACAGGGGAGGAGTCTA

55_Campylopterus_curvipennis_Ciel-17

GGATTATGGCTACTGCTACTTCTAGGATGGTTAGTAGGAGTAGTACGCAGGCGGTTAGAGCAGAGATTGCGGGTATGATG

GAGAATAGGACTGCGGTGGCTGTTGAGATGAGTTGGATTAGTAGGTGGCCTGCTGTTAGGTTAGCTGTGAGGCGGACTCC

TAGGGCTAGAGGGCGGATCAGGAGGCTGGTAGTTTCAATTATGATTAAGGCTGGGATTAGTGGAGTTGGGGTTCCTTCGG

GTAGGAGGTGGCCAAGTGAAGCGGATGGTTGGTTTCGGAGGCCTGTGAGTAGAGTGGCTAGTCATAGTGGGAAGGCTAGT

GCTAGGTTTATAGATAATTGTGTGGTAGGTGTGAATGTGTAGGGGAGTAGGCCTAGTAGGTTGATTGAGAGAAGGAAAAT

TATTAGTGATGACAGGATAAGGGCTCATTTGTGGCCTTTTTTGTTCAATGGGATTATCAGTTGTTTGGTGATTATATAGA

TGAATCACGATTGTAGGGTGGATGTTCGGTTGGTGATTCATCGGTTGTGGGGGGAGGGGAGTAGTAGGGCTGGGAATAAT

AGTGAGATGAGAATTAGGGGGATTCCTAGTAGGTGGGGGCTTATAAATTGGTCGAAGAAGCTTAGGTTCATGGTCAGGGT

CAGGATGAGGGTTTTGTGTTTGTTAGGGTTTTTTCTAGGGGGGAGTTAGTGGGCATGAATGAAAGGAGTTTGGGTTGGAT

GATTAGTGAAAAGGTCAGTCATGAAATAGTTATAATGAATAGTCATGGGTTTGGATTTAGTTGGGGCATATCATTAGGGA

GGGTTGGTAATCCTCTTTCTCTAGCTTAAAAGGCTAGTGCTGTTGCATAGCTTCCTAGTGGTTAAGATGATAGGAAAATT

AACATGATAAACATGATGATGGATGATGATTATTTTTTAATGTAATCTCGGTGTTGAATCTGGGTTAAAGTTAGATGATA

AAATGTGTGATGAAAATATCATGGATAGTATGGAAATTATAACTTAGAACTCCAGTGGTGTTGGTTAGAAAGTTAGAGGA

AGTGAAAAACTTGTAAAATATGTCCGGCAACCATAAATCAAAAAGCAACATAGCTTAGTAAGTATGTGAAAATACCATAA

CCAAATGCAAGACAAAGTGCATCAGTGTCAAAATGAGACTGAATTACACATGAAACCGTCTCATTCAGCAACTCTTGAAG

GAGAGAAAAGGCTCGGGAGCCACTGATGCCCAGGTCAAGAGATTGTGTGCTCCGCGTTAGCCACTGGAAGGGCCTATTGA

GATTACCCAAAAAAAAAGA-GAACCAACC-GCCTAAAAGAAGGGAACCATGCCGCGATTAAGAAAGGTATGAACTATAAA

GTACCAACCAAATGCCTTTGAAAGGAAATCCACAGGGGAGGAGTCTA

56_Campylopterus_curvipennis_Ciel-18

GGATTATGGCTACTGCTACTTCTAGGATGGTTAGTAGGAGTAGTACGCAGGCGGTTAGAGCAGAGATTGCGGGTATGATG

GAGAATAGGACTGCGGTGGCTGTTGAGATGAGTTGGATTAGTAGGTGGCCTGCTGTTAGGTTAGCTGTGAGGCGGACTCC

TAGGGCTAGAGGGCGGATCAGGAGGCTGGTAGTTTCAATTATGATTAAGGCTGGGATTAGTGGAGTTGGGGTTCCTTCGG

GTAGGAGGTGGCCAAGTGAAGCGGATGGTTGGTTTCGGAGGCCTGTGAGTAGAGTGGCTAGTCATAGTGGGAAGGCTAGT

GCTAGGTTTATAGATAATTGTGTGGTAGGTGTGAATGTGTAGGGGAGTAGGCCTAGTAGGTTGATTGAGAGAAGGAAAAT

TATTAGTGATGACAGGATAAGGGCTCATTTGTGGCCTTTTTTGTTCAATGGGATTATCAGTTGTTTGGTGATTATATAGA

TGAATCACGATTGTAGGGTGGATGTTCGGTTGGTGATTCATCGGTTGTGGGGGGAGGGGAGTAGTAGGGCTGGGAATAAT

AGTGAGATGAGAATTAGGGGGATTCCTAGTAGGTGGGGGCTTATAAATTGGTCGAAGAAGCTTAGGTTCATGGTCAGGGT

CAGGATGAGGGTTTTGTGTTTGTTAGGGGTTTTTCTAGGGGGGAGTTAGTGGGCATGAATGAAAGGAGTTTGGGTTGGAT

GATTAGTGAAAAGGTCAGTCATGAAATAGTTATAATGAATAGTCATGGGTTTGGATTTAGTTGGGGCATATCATTAGGGA

GGGTTGGTAATCCTCTTTCTCTAGCTTAAAAGGCTAGTGCTGTTGCATAGCTTCCTAGTGGTTAGGATGATAGGAAAATT

AACATGATAAACATGATGATGGATGATGATTATTTTTTAATGTAATCTCGGTGTTGAATCTGGGTTAAAGTTAGATGATA

AAATGTGTGATGAAAATATCATGGATAGTATGGAAATTATAACTTAGAACTCCAGTGGTGTTGGTTAGAAAGTTAGAGGA

AGTGAAAAACTTGTAAAATATGTCCGGCAACCATAAATCAAAAAGCAACATAGCTTAGTAAGTATGTGAAAATACCATAA

CCAAATGCAAGACAAAGTGCATCAGTGTCAAAATGAGACTGAATTACACATGAAACCGTCTCATTCAGCAACTCTTGAAG

GAGAGAAAAGGCCCGGGAGCCACTGATGCCCAGGTCAAGAGATTGTGTGCTCCGCGTTAGCCACTGGAAGGGCCTATTGA

GATTACCCAAAAAAAAAGA-GAACCAACC-GCCTAAAAGAAGGGAACCATGCCGCGATTAAGAAAGGTATGAACTATAAA

GTACCAACCAAATGCCTTTGAAAGGAAATCCACAGGGGAGGAATCTA

57_Campylopterus_curvipennis_GF-1

GGATTATGGCTACTGCTACTTCTAGGATGGTTAGTAGGAGTAGTACGCAGGCGGTTAGAGCAGAGATTGCGGGTATGATG

GAGAATAGGACTGCGGTGGCTGTTGAGATGAGTTGGATTAGTAGGTGGCCTGCTGTTAGGTTAGCTGTGAGGCGGACTCC

TAGGGCTAGAGGGCGGATCAGGAGGCTGGTAGTTTCAATTATGATTAAGGCTGGGATTAGTGGAGTTGGGGTTCCTTCGG

GTAGGAGGTGGCCAAGTGAAGCGGATGGTTGGTTTCGGAGGCCTGTGAGTAGAGTGGCTAGTCATAGTGGGAAGGCTAGT

GCTAGGTTTATAGATAATTGTGTGGTAGGTGTGAATGTGTAGGGGAGTAGGCCTAGTAGGTTGATTGAGAGAAGGAAAAT

TATTAGTGATGACAGGATAAGGGCTCATTTGTGGCCTTTTTTGTTCAATGGGATTATCAGTTGTTTGGTGATTATATAGA

TGAATCACGATTGTAGGGTGGATGTTCGGTTGGTGATTCATCGGTTGTGGGGGGAGGGGAGTAGTAGGGCTGGGAATAAT

AGTGAGATGAGAATTAGGGGGATTCCTAGTAGGTGGGGGCTTATAAATTGGTCGAAGAAGCTTAGGTTCATGGTCAGGGT

CAGGATGAGGGTTTTGTGTTTGTTAGGGTTTTTTCTAGGGGGGAGTTAGTGGGCATGAATGAAAGGAGTTTGGGTTGGAT

GATTAGTGAAAAGGTCAGTCATGAAATAGTTATAATGAATAGTCATGGGTTTGGATTTAGTTGGGGCATATCATTAGGGA

GGGTTGGTAATCCTCTTTCTCTAGCTTAAAAGGCTAGTGCTGTTGCATAGCTTCCTAGTGGTTAAGATGATAGGAAAATT

AACATGATAAACATGATGATGGATGATGATTATTTTTTAATGTAATCTCGGTGTTGAATCTGGGTTAAAGTTAGATGATA

AAATGTGTGATGAAAATATCATGGATAGTATGGAAATTATAACTTAGAACTCCAGTGGTGTTGGTTAGAAAGTTAGAGGA

AGTGAAAAACTTGTAAAATATGTCCGGCAACCATAAATCAAAAAGCAACATAGCTTAGTAAGTATGTGAAAATACCATAA

CCAAATGCAAGACAAAGTGCATCAGTGTCAAAATGAGACTGAATTACACATGAAACCGTCTCATTCAGCAACTCTTGAAG

GAGAGAAAAGGCTCGGGAGCCACTGATGCCCAGGTCAAGAGATTGTGTGCTCCGCGTTAGCCACTGGAAGGGCCTATTGA

GATTACCCAAAAAAAAAGA-GAACCAACC-GCCTAAAAGAAGGGAACCATGCCGCGATTAAGAAAGGTATGAACTATAAA

GTACCAACCAAATGCCTTTGAAAGGAAATCCACAGGGGAGGAGTCTA

58_Campylopterus_curvipennis_GF-2

GGATTATGGCTACTGCTACTTCTAGGATGGTTAGTAGGAGTAGTACGCAGGCGGTTAGAGCAGAGATTGCGGGTATGATG

GAGAATAGGACTGCGGTGGCTGTTGAGATGAGTTGGATTAGTAGGTGGCCTGCTGTTAGGTTAGCTGTGAGGCGGACTCC

TAGGGCTAGAGGGCGGATCAGGAGGCTGGTAGTTTCAATTATGATTAAGGCTGGGATTAGTGGAGTTGGGGTTCCTTCGG

GTAGGAGGTGGCCAAGTGAAGCGGATGGTTGGTTTCGGAGGCCTGTGAGTAGAGTGGCTAGTCATAGTGGGAAGGCTAGT

GCTAGGTTTATAGATAATTGTGTGGTAGGTGTGAATGTGTAGGGGAGTAGGCCTAGTAGGTTGATTGAGAGAAGGAAAAT

TATTAGTGATGACAGGATAAGGGCTCATTTGTGGCCTTTTTTGTTCAATGGGATTATCAGTTGTTTGGTGATTATATAGA

TGAATCACGATTGTAGGGTGGATGTTCGGTTGGTGATTCATCGGTTGTGGGGGGAGGGGAGTAGTAGGGCTGGGAATAAT

AGTGAGATGAGAATTAGGGGGATTCCTAGTAGGTGGGGGCTTATAAATTGGTCGAAGAAGCTTAGGTTCATGGTCAGGGT

CAGGATGAGGGTTTTGTGTTTGTTAGGGTTTTTTCTAGGGGGGAGTTAGTGGGCATGAATGAAAGGAGTTTGGGTTGGAT

GATTAGTGAAAAGGTCAGTCATGAAATAGTTATAATGAATAGTCATGGGTTTGGATTTAGTTGGGGCATATCATTAGGGA

GGGTTGGTAATCCTCTTTCTCTAGCTTAAAAGGCTAGTGCTGTTGCATAGCTTCCTAGTGGTTAAGATGATAGGAAAATT

AACATGATAAACATGATGATGGATGATGATTATTTTTTAATGTAATCTCGGTGTTGAATCTGGGTTAAAGTTAGATGATA

AAATGTGTGATGAAAATATCATGGATAGTATGGAAATTATAACTTAGAACTCCAGTGGTGTTGGTTAGAAAGTTAGAGGA

AGTGAAAAACTTGTAAAATATGTCCGGCAACCATAAATCAAAAAGCAACATAGCTTAGTAAGTATGTGAAAATACCATAA

CCAAATGCAAGACAAAGTGCATCAGTGTCAAAATGAGACTGAATTACACATGAAACCGTCTCATTCAGCAACTCTTGAAG

GAGAGAAAAGGCTCGGGAGCCACTGATGCCCAGGTCAAGAGATTGTGTGCTCCGCGTTAGCCACTGGAAGGGCCTATTGA

GATTACCCAAAAAAAAAGA-GAACCAACC-GCCTAAAAGAAGGGAACCATGCCGCGATTAAGAAAGGTATGAACTATAAA

GTACCAACCAAATGCCTTTGAAAGGAAATCCACAGGGGAGGAGTCTA

59_Campylopterus_curvipennis_GF-3

GGATTATGGCTACTGCTACTTCTAGGATGGTTAGTAGGAGTAGTACGCAGGCGGTTAGAGCAGAGATTGCGGGTATGATG

GAGAATAGGACTGCGGTGGCTGTTGAGATGAGTTGGATTAGTAGGTGGCCTGCTGTTAGGTTAGCTGTGAGGCGGACTCC

TAGGGCTAGAGGGCGGATCAGGAGGCTGGTAGTTTCAATTATGATTAAGGCTGGGATTAGTGGAGTTGGGGTTCCTTCGG

GTAGGAGGTGGCCAAGTGAAGCGGATGGTTGGTTTCGGAGGCCTGTGAGTAGAGTGGCTAGTCATAGTGGGAAGGCTAGT

GCTAGGTTTATAGATAATTGTGTGGTAGGTGTGAATGTGTAGGGGAGTAGGCCTAGTAGGTTGATTGAGAGAAGGAAAAT

TATTAGTGATGACAGGATAAGGGCTCATTTGTGGCCTTTTTTGTTCAATGGGATTATCAGTTGTTTGGTGATTATATAGA

TGAATCACGATTGTAGGGTGGATGTTCGGTTGGTGATTCATCGGTTGTGGGGGGAGGGGAGTAGTAGGGCTGGGAATAAT

AGTGAGATGAGAATTAGGGGGATTCCTAGTAGGTGGGGGCTTATAAATTGGTCGAAGAAGCTTAGGTTCATGGTCAGGGT

CAGGATGAGGGTTTTGTGTTTGTTAGGGTTTTTTCTAGGGGGGAGTTAGTGGGCATGAATGAAAGGAGTTTGGGTTGGAT

GATTAGTGAAAAGGTCAGTCATGAAATAGTTATAATGAATAGTCATGGGTTTGGATTTAGTTGGGGCATATCATTAGGGA

GGGTTGGTAATCCTCTTTCTCTAGCTTAAAAGGCTAGTGCTGTTGCATAGCTTCCTAGTGGTTAAGATGATAGGAAAATT

AACATGATAAACATGATGATGGATGATGATTATTTTTTAATGTAATCTCGGTGTTGAATCTGGGTTAAAGTTAGATGATA

AAATGTGTGATGAAAATATCATGGATAGTATGGAAATTATAACTTAGAACTCCAGTGGTGTTGGTTAGAAAGTTAGAGGA

AGTGAAAAACTTGTAAAATATGTCCGGCAACCATAAATCAAAAAGCAACATAGCTTAGTAAGTATGTGAAAATACCATAA

CCAAATGCAAGACAAAGTGCATCAGTGTCAAAATGAGACTGAATTACACATGAAACCGTCTCATTCAGCAACTCTTGAAG

GAGAGAAAAGGCTCGGGAGCCACTGATGCCCAGGTCAAGAGATTGTGTGCTCCGCGTTAGCCACTGGAAGGGCCTATTGA

GATTACCCAAAAAAAAAGA-GAACCAACC-GCCTAAAAGAAGGGAACCATGCCGCGATTAAGAAAGGTATGAACTATAAA

GTACCAACCAAATGCCTTTGAAAGGAAATCCACAGGGGAGGAGTCTA

60_Campylopterus_curvipennis_GF-4

GGATTATGGCTACTGCTACTTCTAGGATGGTTAGTAGGAGTAGTACGCAGGCGGTTAGAGCAGAGATTGCGGGTATGATG

GAGAATAGGACTGCGGTGGCTGTTGAGATGAGTTGGATTAGTAGGTGGCCTGCTGTTAGGTTAGCTGTGAGGCGGACTCC

TAGGGCTAGAGGGCGGATCAGGAGGCTGGTAGTTTCAATTATGATTAAGGCTGGGATTAGTGGAGTTGGGGTTCCTTCGG

GTAGGAGGTGGCCAAGTGAAGCGGATGGTTGGTTTCGGAGGCCTGTGAGTAGAGTGGCTAGTCATAGTGGGAAGGCTAGT

GCTAGGTTTATAGATAATTGTGTGGTAGGTGTGAATGTGTAGGGGAGTAGGCCTAGTAGGTTGATTGAGAGAAGGAAAAT

TATTAGTGATGACAGGATAAGGGCTCATTTGTGGCCTTTTTTGTTCAATGGGATTATCAGTTGTTTGGTGATTATATAGA

TGAATCACGATTGTAGGGTGGATGTTCGGTTGGTGATTCATCGGTTGTGGGGGGAGGGGAGTAGTAGGGCTGGGAATAAT

AGTGAGATGAGAATTAGGGGGATTCCTAGTAGGTGGGGGCTTATAAATTGGTCGAAGAAGCTTAGGTTCATGGTCAGGGT

CAGGATGAGGGTTTTGTGTTTGTTAGGGTTTTTTCTAGGGGGGAGTTAGTGGGCATGAATGAAAGGAGTTTGGGTTGGAT

GATTAGTGAAAAGGTCAGTCATGAAATAGTTATAATGAATAGTCATGGGTTTGGATTTAGTTGGGGCATATCATTAGGGA

GGGTTGGTAATCCTCTTTCTCTAGCTTAAAAGGCTAGTGCTGTTGCATAGCTTCCTAGTGGTTAAGATGATAGGAAAATT

AACATGATAAACATGATGATGGATGATGATTATTTTTTAATGTAATCTCGGTGTTGAATCTGGGTTAAAGTTAGATGATA

AAATGTGTGATGAAAATATCATGGATAGTATGGAAATTATAACTTAGAACTCCAGTGGTGTTGGTTAGAAAGTTAGAGGA

AGTGAAAAACTTGTAAAATATGTCCGGCAACCATAAATCAAAAAGCAACATAGCTTAGTAAGTATGTGAAAATACCATAA

CCAAATGCAAGACAAAGTGCATCAGTGTCAAAATGAGACTGAATTACACATGAAACCGTCTCATTCAGCAACTCTTGAAG

GAGAGAAAAGGCTCGGGGGCCACTGATGCCCAGGTCAAGAGATTGTGTGCTCCGCGTTAGCCACTGGAAGGGCCTATTGA

GATTACCCAAAAAAAAAGA-GAACCAACC-GCCTAAAAGAAGGGAACCATGCCGCGATTAAGAAAGGTATGAACTATAAA

GTACCAACCAAATGCCTTTGAAAGGAAATCCACAGGGGAGGAGTCTA

61_Campylopterus_curvipennis_EN-1

GGATTATGGCTACTGCTACTTCTAGGATGGTTAGTAGGAGTAGTACGCAGGCGGTTAGAGCAGAGATTGCGGGTATGATG

GAGAATAGGACTGCGGTGGCTGTTGAGATGAGTTGGATTAGTAGGTGGCCTGCTGTTAGGTTAGCTGTGAGGCGGACTCC

TAGGGCTAGAGGGCGGATCAGGAGGCTGGTAGTTTCAATTATGATTAAGGCTGGGATTAGTGGAGTTGGGGTTCCTTCGG

GTAGGAGGTGGCCAAGTGAAGCGGATGGTTGGTTTCGGAGGCCTGTGAGTAGAGTGGCTAGTCATAGTGGGAAGGCTAGT

GCTAGGTTTATAGATAATTGTGTGGTAGGTGTGAATGTGTAGGGGAGTAGGCCTAGTAGGTTGATTGAGAGAAGGAAAAT

TATTAGTGATGACAGGATAAGGGCTCATTTGTGGCCTTTTTTGTTTAATGGGATTATCAGTTGTTTGGTGATTATATAGA

TGAATCACGATTGTAGGGTGGATGTTCGGTTGGTGATTCATCGGTTGTGGGGGGAGGGGAGTAGTAGGGCTGGGAATAAT

AGTGAGATGAGAATTAGGGGGATTCCTAGTAGGTGGGGGCTTATAAATTGGTCGAAGAAGCTTAGGTTCATGGTCAGGGT

CAGGATGAGGGTTTTGTGTTTGTTAGGGGTTTTTCTAGGGGGGAGTTAGTGGGCATGAATGAAAGGAGTTTGGGTTGGAT

GATTAGTGAAAAGGTCAGTCATGAAATAGTTATAATGAATAGTCATGGGTTTGGATTTAGTTGGGGCATATCATTAGGGA

GGGTTGGTAATCCTCTTTCTCTAGCTTAAAAGGCTAGTGCTGTTGCATAGCTTCCTAGTGGTTAAGATGATAGGAAAATT

AACATGATAAACATGATGATGGATGATGATTATTTTTTAATGTAATCTCGGTGTTGAATCTGGGTTAAAGTTAGATGATA

AAATGTGTGATGAAAATATCATGGATAGTATGGAAATTATAACTTAGAACTCCAGTGGTGTTGGTTAGAAAGTTAGAGGA

AGTGAAAAACTTGTAAAATATGTCCGGCAACCATAAATCAAAAAGCAACATAGCTTAGTAAGTATGTGAAAATACCATAA

CCAAATGCAAGACAAAGTGCATCAGTGTCAAAATGAGACTGAATTACACATGAAACCGTCTCATTCAGCAACTCTTGAAG

GAGAGAAAAGGCTCGGGAGCCACTGATGCTCAGGTCAAGAGATTGTGTGCTCCGCGTTAGCCACTGGAAGGGCCTATTGA

GATTACCCAAAAAAAAAGA-GAACCAACC-GCCTAAAAGAAGGGAACCATGCCGCGATTAAGAAAGGTATGAACTATAAA

GTACCAACCAAATGCCTTTGAAAGGAAGTCCACAGGGGAGTAGTCTA

62_Campylopterus_curvipennis_EN-2

GGATTATGGCTACTGCTACTTCTAGGATGGTTAGTAGGAGTAGTACGCAGGCGGTTAGAGCAGAGATTGCGGGTATGATG

GAGAATAGGACTGCGGTGGCTGTTGAGATGAGTTGGATTAGTAGGTGGCCTGCTGTTAGGTTAGCTGTGAGGCGGACTCC

TAGGGCTAGAGGGCGGATCAGGAGGCTGGTAGTTTCAATTATGATTAAGGCTGGGATTAGTGGAGTTGGGGTTCCTTCGG

GTAGGAGGTGGCCAAGTGAAGCGGATGGTTGGTTTCGGAGGCCTGTGAGTAGAGTGGCTAGTCATAGTGGGAAGGCTAGT

GCTAGGTTTATAGATAATTGTGTGGTAGGTGTGAATGTGTAGGGGAGTAGGCCTAGTAGGTTGATTGAGAGAAGGAAAAT

TATTAGTGATGACAGGATAAGGGCTCATTTGTGGCCTTTTTTGTTTAATGGGATTATCAGTTGTTTGGTGATTATATAGA

TGAATCACGATTGTAGGGTGGATGTTCGGTTGGTGATTCATCGGTTGTGGGGGGAGGGGAGTAGTAGGGCTGGGAATAAT

AGTGAGATGAGAATTAGGGGGATTCCTAGTAGGTGGGGGCTTATAAATTGGTCGAAGAAGCTTAGGTTCATGGTCAGGGT

CAGGATGAGGGTTTTGTGTTTGTTAGGGGTTTTTCTAGGGGGGAGTTAGTGGGCATGAATGAAAGGAGTTTGGGTTGGAT

GATTAGTGAAAAGGTCAGTCATGAAATAGTTATAATGAATAGTCATGGGTTTGGATTTAGTTGGGGCATATCATTAGGGA

GGGTTGGTAATCCTCTTTCTCTAGCTTAAAAGGCTAGTGCTGTTGCATAGCTTCCTAGTGGTTAAGATGATAGGAAAATT

AACATGATAAACATGATGATGGATGATGATTATTTTTTAATGTAATCTCGGTGTTGAATCTGGGTTAAAGTTAGATGATA

AAATGTGTGATGAAAATATCATGGATAGTATGGAAATTATAACTTAGAACTCCAGTGGTGTTGGTTAGAAAGTTAGAGGA

AGTGAAAAACTTGTAAAATATGTCCGGCAACCATAAATCAAAAAGCAACATAGCTTAGTAAGTATGTGAAAATACCATAA

CCAAATGCAAGACAAAGTGCATCAGTGTCAAAATGAGACTGAATTACACATGAAACCGTCTCATTCAGCAACTCTTGAAG

GAGAGAAAAGGCTCGGGAGCCACTGATGCTCAGGTCAAGAGATTGTGTGCTCCGCGTTAGCCACTGGAAGGGCCTATTGA

GATTACCCAAAAAAAAAGA-GAACCAACC-GCCTAAAAGAAGGGAACCATGCCGCGATTAAGAAAGGTATGAACTATAAA

GTACCAACCAAATGCCTTTGAAAGGAAGTCCACAGGGGAGGAGTCTA

63_Campylopterus_curvipennis_EN-3

GGATTATGGCTACTGCTACTTCTAGGATGGTTAGTAGGAGTAGTACGCAGGCGGTTAGAGCAGAGATTGCGGGTATGATG

GAGAATAGGACTGCGGTGGCTGTTGAGATGAGTTGGATTAGTAGGTGGCCTGCTGTTAGGTTAGCTGTGAGGCGGACTCC

TAGGGCTAGAGGGCGGATCAGGAGGCTGGTAGTTTCAATTATGATTAAGGCTGGGATTAGTGGAGTTGGGGTTCCTTCGG

GTAGGAGGTGGCCAAGTGAAGCGGATGGTTGGTTTCGGAGGCCTGTGAGTAGAGTGGCTAGTCATAGTGGGAAGGCTAGT

GCTAGGTTTATAGATAATTGTGTGGTAGGTGTGAATGTGTAGGGGAGTAGGCCTAGTAGGTTGATTGAGAGAAGGAAAAT

TATTAGTGATGACAGGATAAGGGCTCATTTGTGGCCTTTTTTGTTCAATGGGATTATCAGTTGTTTGGTGATTATATAGA

TGAATCACGACTGTAGGGTGGATGTTCGGTTGGTGATTCATCGGTTGTGGGGGGAGGGGAGTAGTAGGGCTGGGAATAAT

AGTGAGATGAGAATTAGGGGGATTCCTAGTAGGTGGGGGCTTATAAATTGGTCGAAGAAGCTTAGGTTCATGGTCAGGGT

CAGGATGAGGGTTTTGTGTTTGTTAGGGTTTTTTCTAGGGGGGAGTTAGTGGGCATGAATGAAAGGAGTTTGGGTTGGAT

GATTAGTGAAAAGGTCAGTCATGAAATAGTTATAATGAATAGTCATGGGTTTGGATTTAGTTGGGGCATATCATTAGGGA

GGGTTGGTAATCCTCTTTCTCTAGCTTAAAAGGCTAGTGCTGTTGCATAGCTTCCTAGTGGTTAAGATGATAGGAAAATT

AACATGATAAACATGATGATGGATGATGATTATTTTTTAATGTAATCTCGGTGTTGAATCTGGGTTAAAGTTAGATGATA

AAATGTGTGATGAAAATATCATGGATAGTATGGAAATTATAACTTAGAACTCCAGTGGTGTTGGTTAGAAAGTTAGAGGA

AGTGAAAAACTTGTAAAATATGTCCGGCAACCATAAATCAAAAAGCAACATAGCTTAGTAAGTATGTGAAAATACCATAA

CCAAATGCAAGACAAAGTGCATCAGTGTCAAAATGAGACTGAATTACACATGAAACCGTCTCATTCAGCAACTCTTGAAG

GAGAGAAAAGGCTCGGGAGCCACTGATGCTCAGGTCAAGAGATTGTGTGCTCCGCGTTAGCCACTGGAAGGGCCTATTGA

GATTACCCAAAAAAAAAGA-GAACCAACC-GCCTAAAAGAAGGGAACCATGCCGCGATTAAGAAAGGTATGAACTATAAA

GTACCAACCAAATGCCTTTGAAAGGAAATCCACAGGGGAGGAGTCTA

64_Campylopterus_curvipennis_EN-4

GGATTATGGCTACTGCTACTTCTAGGATGGTTAGTAGGAGTAGTACGCAGGCGGTTAGAGCAGAGATTGCGGGTATGATG

GAGAATAGGACTGCGGTGGCTGTTGAGATGAGTTGGATTAGTAGGTGGCCTGCTGTTAGGTTAGCTGTGAGGCGGACTCC

TAGGGCTAGAGGGCGGATCAGGAGGCTGGTAGTTTCAATTATGATTAAGGCTGGGATTAGTGGAGTTGGGGTTCCTTCGG

GTAGGAGGTGGCCAAGTGAAGCGGATGGTTGGTTTCGGAGGCCTGTGAGTAGAGTGGCTAGTCATAGTGGGAAGGCTAGT

GCTAGGTTTATAGATAATTGTGTGGTAGGTGTGAATGTGTAGGGGAGTAGGCCTAGTAGGTTGATTGAGAGAAGGAAAAT

TATTAGTGATGACAGGATAAGGGCTCATTTGTGGCCTTTTTTGTTCAATGGGATTATCAGTTGTTTGGTGATTATATAGA

TGAATCACGATTGTAGGGTGGATGTTCGGTTGGTGATTCATCGGTTGTGGGGGGAGGGGAGTAGTAGGGCTGGGAATAAT

AGTGAGATGAGAATTAGGGGGATTCCTAGTAGGTGGGGGCTTATAAATTGGTCGAAGAAGCTTAGGTTCATGGTCAGGGT

CAGGATGAGGGTTTTGTGTTTGTTAGGGTTTTTTCTAGGGGGGAGTTAGTGGGCATGAATGAAAGGAGTTTGGGTTGGAT

GATTAGTGAAAAGGTCAGTCATGAAATAGTTATAATGAATAGTCATGGGTTTGGATTTAGTTGGGGCATATCATTAGGGA

GGGTTGGTAATCCTCTTTCTCTAGCTTAAAAGGCTAGTGCTGTTGCATAGCTTCCTAGTGGTTAAGATGATAGGAAAATT

AACATGATAAACATGATGATGGATGATGATTATTTTTTAATGTAATCTCGGTGTTGAATCTGGGTTAAAGTTAGATGATA

AAATGTGTGATGAAAATATCATGGATAGTATGGAAATTATAACTTAGAACTCCAGTGGTGTTGGTTAGAAAGTTAGAGGA

AGTGAAAAACTTGTAAAATATGTCCGGCAACCATAAATCAAAAAGCAACATAGCTTAGTAAGTATGTGAAAATACCATAA

CCAAATGCAAGACAAAGTGCATCAGTGTCAAAATGAGACTGAATTACACATGAAACCGTCTCATTCAGCAACTCTTGAAG

GAGAGAAAAGGCTCGGGAGCCACTGATGCCCAGGTCAAGAGATTGTGTGCTCCGCGTTAGCCACTGGAAGGGCCTATTGA

GATTACCCAAAAAAAAAGA-GAACCAACC-GCCTAAAAGAAGGGAACCATGCCGCGATTAAGAAAGGTATGAACTATAAA

GTACCAACCAAATGCCTTTGAAAGGAAATCCACAGGGGAGGAGTCTA

65_Campylopterus_curvipennis_EN-5

GGATTATGGCTACTGCTACTTCTAGGATGGTTAGTAGGAGTAGTACGCAGGCGGTTAGAGCAGAGATTGCGGGTATGATG

GAGAATAGGACTGCGGTGGCTGTTGAGATGAGTTGGATTAGTAGGTGGCCTGCTGTTAGGTTAGCTGTGAGGCGGACTCC

TAGGGCTAGAGGGCGGATCAGGAGGCTGGTAGTTTCAATTATGATTAAGGCTGGGATTAGTGGAGTTGGGGTTCCTTCGG

GTAGGAGGTGGCCAAGTGAAGCGGATGGTTGGTTTCGGAGGCCTGTGAGTAGAGTGGCTAGTCATAGTGGGAAGGCTAGT

GCTAGGTTTATAGATAATTGTGTGGTAGGTGTGAATGTGTAGGGGAGTAGGCCTAGTAGGTTGATTGAGAGAAGGAAAAT

TATTAGTGATGACAGGATAAGGGCTCATTTGTGGCCTTTTTTGTTCAATGGGATTATCAGTTGTTTGGTGATTATATAGA

TGAATCACGATTGTAGGGTGGATGTTCGGTTGGTGATTCATCGGTTGTGGGGGGAGGGGAGTAGTAGGGCTGGGAATAAT

AGTGAGATGAGAATTAGGGGGATTCCTAGTAGGTGGGGGCTTATAAATTGGTCGAAGAAGCTTAGGTTCATGGTCAGGGT

CAGGATGAGGGTTTTGTGTTTGTTAGGGGTTTTTCTAGGGGGGAGTTAGTGGGCATGAATGAAAGGAGTTTGGGTTGGAT

GATTAGTGAAAAGGTCAGTCATGAAATAGTTATAATGAATAGTCATGGGTTTGGATTTAGTTGGGGCATATCATTAGGGA

GGGTTGGTAATCCTCTTTCTCTAGCTTAAAAGGCTAGTGCTGTTGCATAGCTTCCTAGTGGTTAAGATGATAGGAAAATT

AACATGATAAACATGATGATGGATGATGATTATTTTTTAATGTAATCTCGGTGTTGAATCTGGGTTAAAGTTAGATGATA

AAATGTGTGATGAAAATATCATGGATAGTATGGAAATTATAACTTAGAACTCCAGTGGTGTTGGTTAGAAAGTTAGAGGA

AGTGAAAAACTTGTAAAATATGTCCGGCAACCATAAATCAAAAAGCAACATAGCTTAGTAAGTATGTGAAAATACCATAA

CCAAATGCAAGACAAAGTGCATCAGTGTCAAAATGAGACTGAATTACACATGAAACCGTCTCATTCAGCAACTCCTGAAG

GAGAGAAAAGGCTCGGGAGCCACTGATGCTCAGGTCAAGAGATTGTGTGCTCCGCGTTAGCCACTGGAAGGGCCTATTGA

GATTACCCAAAAAAAAAGA-GAACCAACC-GCCTAAAAGAAGGGAACCATGCCGCGATTAAGAAAGGTATGAACTATAAA

GTACCAACCAAATGCCTTTGAAAGGAAATCCACAGGGGAGGAGTCTA

66_Campylopterus_curvipennis_EN-6

GGATTATGGCTACTGCTACTTCTAGGATGGTTAGTAGGAGTAGTACGCAGGCGGTTAGGGCAGAGATTGCGGGTATGATG

GAGAATAGGACTGCGGTGGCTGTTGAGATGAGTTGGATTAGTAGGTGGCCTGCTGTTAGGTTAGCTGTGAGGCGGACTCC

TAGGGCTAGAGGGCGGATCAGGAGGCTGGTAGTTTCAATTATGATTAAGGCTGGGATTAGTGGAGTTGGGGTTCCTTCGG

GTAGGAGGTGGCCAAGTGAAGCGGATGGTTGGTTTCGGAGGCCTGTGAGTAGAGTGGCTAGTCATAGTGGGAAGGCTAGT

GCTAGGTTTATAGATAATTGTGTGGTAGGTGTGAATGTGTAGGGGAGTAGGCCTAGTAGGTTGATTGAGAGAAGGAAAAT

TATTAGTGATGACAGGATAAGGGCTCATTTGTGGCCTTTTTTGTTCAATGGGATTATCAGTTGTTTGGTGATTATATAGA

TGAATCACGATTGTAGGGTGGATGTTCGGTTGGTGATTCATCGGTTGTGGGGGGAGGGGAGTAGTAGGGCTGGGAATAAT

AGTGAGATGAGAATTAGGGGGATTCCTAGTAGGTGGGGGCTTATAAATTGGTCGAAGAAGCTTAGGTTCATGGTCAGGGT

CAGGATGAGGGTTTTGTGTTTGTTAGGGTTTTTTCTAGGGGGGAGTTAGTGGGCATGAATGAAAGGAGTTTGGGTTGGAT

GATTAGTGAAAAGGTCAGTCATGAAATAGTTATAATGAATAGTCATGGGTTTGGATTTAGTTGGGGCATATCATTAGGGA

GGGTTGGTAATCCTCTTTCTCTAGCTTAAAAGGCTAGTGCTGTTGCATAGCTTCCTAGTGGTTAAGATGATAGGAAAATT

AACATGATAAACATGATGATGGATGATGATTATTTTTTAATGTAATCTCGGTGTTGAATCTGGGTTAAAGTTAGATGATA

AAATGTGTGATGAAAATATCATGGATAGTATGGAAATTATAACTTAGAACTCCAGTGGTGTTGGTTAGAAAGTTAGAGGA

AGTGAAAAACTTGTAAAATATGTCCGGCAACCATAAATCAAAAAGCAACATAGCTTAGTAAGTATGTGAAAATACCATAA

CCAAATGCAAGACAAAGTGCATCAGTGTCAAAATGAGACTGAATTACACATGAAACCGTCTCATTCAGCAACTCTTGAAG

GAGAGAAAAGGCTCGGGAGCCACTGATGCCCAGGTCAAGAGATTGTGTGCTCCGCGTTAGCCACTGGAAGGGCCTATTGA

GATTACCCAAAAAAAAAGA-GAACCAACC-GCCTAAAAGAAGGGAACCATGCCGCGATTAAGAAAGGTATGAACTATAAA

GTACCAACCAAATGCCTTTGAAAGGAAATCCACAGGGGAGGAGTCTA

67_Campylopterus_curvipennis_AQM-1

GGATTATGGCTACTGCTACTTCTAGGATGGTTAGTAGGAGTAGTACGCAGGCGGTTAGAGCAGAGATTGCGGGTATGATG

GAGAATAGGACTGCGGTGGCTGTTGAGATGAGTTGGATTAGTAGGTGGCCTGCTGTTAGGTTAGCTGTGAGGCGGACTCC

TAGGGCTAGAGGGCGGATCAGGAGGCTGGTAGTTTCAATTATGATTAAGGCTGGGATTAGTGGAGTTGGGGTTCCTTCGG

GTAGGAGGTGGCCAAGTGAAGCGGATGGTTGGTTTCGGAGGCCTGTGAGTAGAGTGGCTAGTCATAGTGGGAAGGCTAGT

GCTAGGTTTATAGATAATTGTGTGGTAGGTGTGAATGTGTAGGGGAGTAGGCCTAGTAGGTTGATTGAGAGAAGGAAAAT

TATTAGTGATGACAGGATAAGGGCTCATTTGTGGCCTTTTTTGTTCAATGGGATTATCAGTTGTTTGGTGATTATATAGA

TGAATCACGATTGTAGGGTGGATGTTCGGTTGGTGATTCATCGGTTGTGGGGGGAGGGGAGTAGTAGGGCTGGGAATAAT

AGTGAGATGAGAATTAGGGGGATTCCTAGTAGGTGGGGGCTTATAAATTGGTCGAAGAAGCTTAGGTTCATGGTCAGGGT

CAGGATGAGGGTTTTGTGTTTGTTAGGGTTTTTTCTAGGGGGGAGTTAGTGGGCATGAATGAAAGGAGTTTGGGTTGGAT

GATTAGTGAAAAGGTCAGTCATGAAATAGTTATAATGAATAGTCATGGGTTTGGATTTAGTTGGGGCATATCATTAGGGA

GGGTTGGTAATCCTCTTTCTCTAGCTTAAAAGGCTAGTGCTGTTGCATAGCTTCCTAGTGGTTAAGATGATAGGAAAATT

AACATGATAAACATGATGATGGATGATGATTATTTTTTAATGTAATCTCGGTGTTGAATCTGGGTTAAAGTTAGATGATA

AAATGTGTGATGAAAATATCATGGATAGTATGGAAATTATAACTTAGAACTCCAGTGGTGTTGGTTAGAAAGTTAGAGGA

AGTGAAAAACTTGTAAAATATGTCCGGCAACCATAAATCAAAAAGCAACATAGCTTAGTAAGTATGTGAAAATACCATAA

CCAAATGCAAGACAAAGTGCATCAGTGTCAAAATGAGACTGAATTACACATGAAACCGTCTCATTCAGCAACTCTTGAAG

GAGAGAAAAGGCTCGGGAGCCACTGATGCCCAGGTCAAGAGATTGTGTGCTCCGCGTTAGCCACTGGAAGGGCCTATTGA

GATTACCCAAAAAAAAAGA-GAACCAACC-GCCTAAAAGAAGGGAACCATGCCGCGATTAAGAAAGGTATGAACTATAAA

GTACCAACCAAATGCCTTTGAAAGGAAATCCACAGGGGAGGAGTCTA

68_Campylopterus_curvipennis_AQM-2

GGATTATGGCTACTGCTACTTCTAGGATGGTTAGTAGGAGTAGTACGCAGGCGGTTAGAGCAGAGATTGCGGGTATGATG

GAGAATAGGACTGCGGTGGCTGTTGAGATGAGTTGGATTAGTAGGTGGCCTGCTGTTAGGTTAGCTGTGAGGCGGACTCC

TAGGGCTAGAGGGCGGATCAGGAGGCTGGTAGTTTCAATTATGATTAAGGCTGGGATTAGTGGAGTTGGGGTTCCTTCGG

GTAGGAGGTGGCCAAGTGAAGCGGATGGTTGGTTTCGGAGGCCTGTGAGTAGAGTGGCTAGTCATAGTGGGAAGGCTAGT

GCTAGGTTTATAGATAATTGTGTGGTAGGTGTGAATGTGTAGGGGAGTAGGCCTAGTAGGTTGATTGAGAGAAGGAAAAT

TATTAGTGATGACAGGATAAGGGCTCATTTGTGGCCTTTTTTGTTCAATGGGATTATCAGTTGTTTGGTGATTATATAGA

TGAATCACGATTGTAGGGTGGATGTTCGGTTGGTGATTCATCGGTTGTGGGGGGAGGGGAGTAGTAGGGCTGGGAATAAT

AGTGAGATGAGAATTAGGGGGATTCCTAGTAGGTGGGGGCTTATAAATTGGTCGAAGAAGCTTAGGTTCATGGTCAGGGT

CAGGATGAGGGTTTTGTGTTTGTTAGGGTTTTTTCTAGGGGGGAGTTAGTGGGCATGAATGAAAGGAGTTTGGGTTGGAT

GATTAGTGAAAAGGTCAGTCATGAAATAGTTATAATGAATAGTCATGGGTTTGGATTTAGTTGGGGCATATCATTAGGGA

GGGTTGGTAATCCTCTTTCTCTAGCTTAAAAGGCTAGTGCTGTTGCATAGCTTCCTAGTGGTTAAGATGATAGGAAAATT

AACATGATAAACATGATGATGGATGATGATTATTTTTTAATGTAATCTCGGTGTTGAATCTGGGTTAAAGTTAGATGATA

AAATGTGTGATGAAAATATCATGGATAGTATGGAAATTATAACTTAGAACTCCAGTGGTGTTGGTTAGAAAGTTAGAGGA

AGTGAAAAACTTGTAAAATATGTCCGGCAACCATAAATCAAAAAGCAACATAGCTTAGTAAGTATGTGAAAATACCATAA

CCAAATGCAAGACAAAGTGCATCAGTGTCAAAATGAGACTGAATTACACATGAAACCGTCTCATTCAGCAACTCTTGAAG

GAGAGAAAAGGCTCGGGAGCCACTGATGCCCAGGTCAAGAGATTGTGTGCTCCGCGTTAGCCACTGGAAGGGCCTATTGA

GATTACCCAAAAAAAAAGA-GAACCAACC-GCCTAAAAGAAGGGAACCATGCCGCGATTAAGAAAGGTATGAACTATAAA

GTACCAACCAAATGCCTTTGAAAGGAAATCCACAGGGGAGGAGTCTA

69_Campylopterus_curvipennis_AQM-3

GGATTATGGCTACTGCTACTTCTAGGATGGTTAGTAGGAGTAGTACGCAGGCGGTTAGAGCAGAGATTGCGGGTATGATG

GAGAATAGGACTGCGGTGGCTGTTGAGATGAGTTGGATTAGTAGGTGGCCTGCTGTTAGGTTAGCTGTGAGGCGGACTCC

TAGGGCTAGAGGGCGGATCAGGAGGCTGGTAGTTTCAATTATGATTAAGGCTGGGATTAGTGGAGTTGGGGTTCCTTCGG

GTAGGAGGTGGCCAAGTGAAGCGGATGGTTGGTTTCGGAGGCCTGTGAGTAGAGTGGCTAGTCATAGCGGGAAGGCTAGT

GCTAGGTTTATAGATAATTGTGTGGTAGGTGTGAATGTGTAGGGGAGTAGGCCTAGTAGGTTGATTGAGAGAAGGAAAAT

TATTAGTGATGACAGGATAAGGGCTCATTTGTGGCCTTTTTTGTTCAATGGGATTATCAGTTGTTTGGTGATTATATAGA

TGAATCACGATTGTAGGGTGGATGTTCGGTTGGTGATTCATCGGTTGTGGGGGGAGGGGAGTAGTAGGGCTGGGAATAAT

AGTGAGATGAGAATTAGGGGGATTCCTAGTAGGTGGGGGCTTATAAATTGGTCGAAGAAGCTTAGGTTCATGGTCAGGGT

CAGGATGAGGGTTTTGTGTTTGTTAGGGTTTTTTCTAGGGGGGAGTTAGTGGGCATGAATGAAAGGAGTTTGGGTTGGAT

GATTAGTGAAAAGGTCAGTCATGAAATAGTTATAATGAATAGTCATGGGTTTGGATTTAGTTGGGGCATATCATTAGGGA

GGGTTGGTAATCCTCTTTCTCTAGCTTAAAAGGCTAGTGCTGTTGCATAGCTTCCTAGTGGTTAAGATGATAGGAAAATT

AACATGATAAACATGATGATGGATGATGATTATTTTTTAATGTAATCTCGGTGTTGAATCTGGGTTAAAGTTAGATGATA

AAATGTGTGATGAAAATATCATGGATAGTATGGAAATTATAACTTAGAACTCCAGTGGTGTTGGTTAGAAAGTTAGAGGA

AGTGAAAAACTTGTAAAATATGTCCGGCAACCATAAATCAAAAAGCAACATAGCTTAGTAAGTATGTGAAAATACCATAA

CCAAATGCAAGACAAAGTGCATCAGTGTCAAAATGAGACTGAATTACACATGAAACCGTCTCATTCAGCAACTCTTGAAG

GAGAGAAAAGGCTCGGGAGCCACTGATGCCCAGGTCAAGAGATTGTGTGCTCCGCGTTAGCCACTGGAAGGGCCTATTGA

GATTACCCAAAAAAAAAGA-GAACCAACC-GCCTAAAAGAAGGGAACCATGCCGCGATTAAGAAAGGTATGAACTATAAA

GTACCAACCAAATGCCTTTGAAAGGAAATCCACAGGGGAGGAGTCTA

70_Campylopterus_curvipennis_AQM-4

GGATTATGGCTACTGCTACTTCTAGGATGGTTAGTAGGAGTAGTACGCAGGCGGTTAGAGCAGAGATTGCGGGTATGATG

GAGAATAGGACTGCGGTGGCTGTTGAGATGAGTTGGATTAGTAGGTGGCCTGCTGTTAGGTTAGCTGTGAGGCGGACTCC

TAGGGCTAGAGGGCGGATCAGGAGGCTGGTAGTTTCAATTATGATTAAGGCTGGGATTAGTGGAGTTGGGGTTCCTTCGG

GTAGGAGGTGGCCAAGTGAAGCGGATGGTTGGTTTCGGAGGCCTGTGAGTAGAGTGGCTAGTCATAGTGGGAAGGCTAGT

GCTAGGTTTATAGATAATTGTGTGGTAGGTGTGAATGTGTAGGGGAGTAGGCCTAGTAGGTTGATTGAGAGAAGGAAAAT

TATTAGTGATGACAGGATAAGGGCTCATTTGTGGCCTTTTTTGTTCAATGGGATTATCAGTTGTTTGGTGATTATATAGA

TGAATCACGATTGTAGGGTGGATGTTCGGTTGGTGATTCATCGGTTGTGGGGGGAGGGGAGTAGTAGGGCTGGGAATAAT

AGTGAGATGAGAATTAGGGGGATTCCTAGTAGGTGGGGGCTTATAAATTGGTCGAAGAAGCTTAGGTTCATGGTCAGGGT

CAGGATGAGGGTTTTGTGTTTGTTAGGGTTTTTTCTAGGGGGGAGTTAGTGGGCATGAATGAAAGGAGTTTGGGTTGGAT

GATTAGTGAAAAGGTCAGTCATGAAATAGTTATAATGAATAGTCATGGGTTTGGATTTAGTTGGGGCATATCATTAGGGA

GGGTTGGTAATCCTCTTTCTCTAGCTTAAAAGGCTAGTGCTGTTGCATAGCTTCCTAGTGGTTAAGATGATAGGAAAATT

AACATGATAAACATGATGATGGATGATGATTATTTTTTAATGTAATCTCGGTGTTGAATCTGGGTTAAAGTTAGATGATA

AAATGTGTGATGAAAATATCATGGATAGTATGGAAATTATAACTTAGAACTCCAGTGGTGTTGGTTAGAAAGTTAGAGGA

AGTGAAAAACTTGTAAAATATGTCCGGCAACCATAAATCAAAAAGCAACATAGCTTAGTAAGTATGTGAAAATACCATAA

CCAAATGCAAGACAAAGTGCATCAGTGTCAAAATGAGACTGAATTACACATGAAACCGTCTCATTCAGCAACTCTTGAAG

GAGAGAAAAGGCTCGGGAGCCACTGATGCCCAGGTCAAGAGATTGTGTGCTCCGCGTTAGCCACTGGAAGGGCCTATTGA

GATTACCCAAAAAAAAAGA-GAACCAACC-GCCTAAAAGAAGGGAACCATGCCGCGATTAAGAAAGGTATGAACTATAAA

GTACCAACCAAATGCCTTTGAAAGGAAATCCACAGGGGAGGAGTCTA

71_Campylopterus_curvipennis_Xil-1

GGATTATGGCTACTGCTACTTCTAGGATGGTTAGTAGGAGTAGTACGCAGGCGGTTAGAGCAGAGATTGCGGGTATGATG

GAGAATAGGACTGCGGTGGCTGTTGAGATGAGTTGGATTAGTAGGTGGCCTGCTGTTAGGTTAGCTGTGAGGCGGACTCC

TAGGGCTAGAGGGCGGATCAGGAGGCTGGTAGTTTCAATTATGATTAAGGCTGGGATTAGTGGAGTTGGGGTTCCTTCGG

GTAGGAGGTGGCCAAGTGAAGCGGATGGTTGGTTTCGGAGGCCTGTGAGTAGAGTGGCTAGTCATAGCGGGAAGGCTAGT

GCTAGGTTTATAGATAATTGTGTGGTAGGTGTGAATGTGTAGGGGAGTAGGCCTAGTAGGTTGATTGAGAGAAGGAAAAT

TATTAGTGATGACAGGATAAGGGCTCATTTGTGGCCTTTTTTGTTCAATGGGATTATCAGTTGTTTGGTGATTATATAGA

TGAATCACGATTGTAGGGTGGATGTTCGGTTGGTGATTCATCGGTTGTGGGGGGAGGGGAGTAGTAGGGCTGGGAATAAT

AGTGAGATGAGAATTAGGGGGATTCCTAGTAGGTGGGGGCTTATAAATTGGTCGAAGAAGCTTAGGTTCATGGTCAGGGT

CAGGATGAGGGTTTTGTGTTTGTTAGGGTTTTTTCTAGGGGGGAGTTAGTGGGCATGAATGAAAGGAGTTTGGGTTGGAT

GATTAGTGAAAAGGTCAGTCATGAAATAGTTATAATGAATAGTCATGGGTTTGGATTTAGTTGGGGCATATCATTAGGGA

GGGTTGGTAATCCTCTTTCTCTAGCTTAAAAGGCTAGTGCTGTTGCATAGCTTCCTAGTGGTTAAGATGATAGGAAAATT

AACATGATAAACATGATGATGGATGATGATTATTTTTTAATGTAATCTCGGTGTTGAATCTGGGTTAAAGTTAGATGATA

AAATGTGTGATGAAAATATCATGGATAGTATGGAAATTATAACTTAGAACTCCAGTGGTGTTGGTTAGAAAGTTAGAGGA

AGTGAAAAACTTGTAAAATATGTCCGGCAACCATAAATCAAAAAGCAACATAGCTTAGTAAGTATGTGAAAATACCATAA

CCAAATGCAAGACAAAGTGCATCAGTGTCAAAATGAGACTGAATTACACATGAAACCGTCTCATTCAGCAACTCTTGAAG

GAGAGAAAAGGCTCGGGAGCCACTGATGCCCAGGTCAAGAGATTGTGTGCTCCGCGTTAGCCACTGGAAGGGCCTATTGA

GATTACCCAAAAAAAAAGA-GAACCAACC-GCCTAAAAGAAGGGAACCATGCCGCGATTAAGAAAGGTATGAACTATAAA

GTACCAACCAAATGCCTTTGAAAGGAAATCCACAGGGGAGGAGTCTA

72_Campylopterus_curvipennis_Xil-2

GGATTATGGCTACTGCTACTTCTAGGATGGTTAGTAGGAGTAGTACGCAGGCGGTTAGAGCAGAGATTGCGGGTATGATG

GAGAATAGGACTGCGGTGGCTGTTGAGATGAGTTGGATTAGTAGGTGGCCTGCTGTTAGGTTAGCTGTGAGGCGGACTCC

TAGGGCTAGAGGGCGGATCAGGAGGCTGGTAGTTTCAATTATGATTAAGGCTGGGATTAGTGGAGTTGGGGTTCCTTCGG

GTAGGAGGTGGCCAAGTGAAGCGGATGGTTGGTTTCGGAGGCCTGTGAGTAGAGTGGCTAGTCATAGTGGGAAGGCTAGT

GCTAGGTTTATAGATAATTGTGTGGTAGGTGTGAATGTGTAGGGGAGTAGGCCTAGTAGGTTGATTGAGAGAAGGAAAAT

TATTAGTGATGACAGGATAAGGGCTCATTTGTGGCCTTTTTTGTTCAATGGGATTATCAGTTGTTTGGTGATTATATAGA

TGAATCACGATTGTAGGGTGGATGTTCGGTTGGTGATTCATCGGTTGTGGGGGGAGGGGAGTAGTAGGGCTGGGAATAAT

AGTGAGATGAGAATTAGGGGGATTCCTAGTAGGTGGGGGCTTATAAATTGGTCGAAGAAGCTTAGGTTCATGGTCAGGGT

CAGGATGAGGGTTTTGTGTTTGTTAGGGTTTTTTCTAGGGGGGAGTTAGTGGGCATGAATGAAAGGAGTTTGGGTTGGAT

GATTAGTGAAAAGGTCAGTCATGAAATAGTTATAATGAATAGTCATGGGTTTGGATTTAGTTGGGGCATATCATTAGGGA

GGGTTGGTAATCCTCTTTCTCTAGCTTAAAAGGCTAGTGCTGTTGCATAGCTTCCTAGTGGTTAAGATGATAGGAAAATT

AACATGATAAACATGATGATGGATGATGATTATTTTTTAATGTAATCTCGGTGTTGAATCTGGGTTAAAGTTAGATGATA

AAATGTGTGATGAAAATATCATGGATAGTATGGAAATTATAACTTAGAACTCCAGTGGTGTTGGTTAGAAAGTTAGAGGA

AGTGAAAAACTTGTAAAATATGTCCGGCAACCATAAATCAAAAAGCAACATAGCTTAGTAAGTATGTGAAAATACCATAA

CCAAATGCAAGACAAAGTGCATCAGTGTCAAAATGAGACTGAATTACACATGAAACCGTCTCATTCAGCAACTCTTGAAG

GAGAGAAAAGGCTCGGGAGCCACTGATGCCCAGGTCAAGAGATTGTGTGCTCCGCGTTAGCCACTGGAAGGGCCTATTGA

GATTACCCAAAAAAAAAGA-GAACCAACC-GCCTAAAAGAAGGGAACCATGCCGCGATTAAGAAAGGTATGAACTATAAA

GTACCAACCAAATGCCTTTGAAAGGAAATCCACAGGGGAGGAGCCTA

73_Campylopterus_curvipennis_Xil-3

GGATTATGGCTACTGCTACTTCTAGGATGGTTAGTAGGAGTAGTACGCAGGCGGTTAGAGCAGAGATTGCGGGTATGATG

GAGAATAGGACTGCGGTGGCTGTTGAGATGAGTTGGATTAGTAGGTGGCCTGCTGTTAGGTTAGCTGTGAGGCGGACTCC

TAGGGCTAGAGGGCGGATCAGGAGGCTGGTAGTTTCAATTATGATTAAGGCTGGGATTAGTGGAGTTGGGGTTCCTTCGG

GTAGGAGGTGGCCAAGTGAAGCGGATGGTTGGTTTCGGAGGCCTGTGAGTAGAGTGGCTAGTCATAGTGGGAAGGCTAGT

GCTAGGTTTATAGATAATTGTGTGGTAGGTGTGAATGTGTAGGGGAGTAGGCCTAGTAGGTTGATTGAGAGAAGGAAAAT

TATTAGTGATGACAGGATAAGGGCTCATTTGTGGCCTTTTTTGTTCAATGGGATTATCAGTTGTTTGGTGATTATATAGA

TGAATCACGATTGTAGGGTGGATGTTCGGTTGGTGATTCATCGGTTGTGGGGGGAGGGGAGTAGTAGGGCTGGGAATAAT

AGTGAGATGAGAATTAGGGGGATTCCTAGTAGGTGGGGGCTTATAAATTGGTCGAAGAAGCTTAGGTTCATGGTCAGGGT

CAGGATGAGGGTTTTGTGTTTGTTAGGGTTTTTTCTAGGGGGGAGTTAGTGGGCATGAATGAAAGGAGTTTGGGTTGGAT

GATTAGTGAAAAGGTCAGTCATGAAATAGTTATAATGAATAGTCATGGGTTTGGATTTAGTTGGGGCATATCATTAGGGA

GGGTTGGTAATCCTCTTTCTCTAGCTTAAAAGGCTAGTGCTGTTGCATAGCTTCCTAGTGGTTAAGATGATAGGAAAATT

AACATGATAAACATGATGATGGATGATGATTATTTTTTAATGTAATCTCGGTGTTGAATCTGGGTTAAAGTTAGATGATA

AAATGTGTGATGAAAATATCATGGATAGTATGGAAATTATAACTTAGAACTCCAGTGGTGTTGGTTAGAAAGTTAGAGGA

AGTGAAAAACTTGTAAAATATGTCCGGCAACCATAAATCAAAAAGCAACATAGCTTAGTAAGTATGTGAAAATACCATAA

CCAAATGCAAGACAAAGTGCATCAGTGTCAAAATGAGACTGAATTACACATGAAACCGTCTCATTCAGCAACTCTTGAAG

GAGAGAAAAGGCTCGGGAGCCACTGATGCCCAGGTCAAGAGATTGTGTGCTCCGCGTTAGCCACTGGAAGGGCCTATTGA

GATTACCCAAAAAAAAAGA-GAACCAACC-GCCTAAAAGAAGGGAACCATGCCGCGATTAAGAAAGGTATGAACTATAAA

GTACCAACCAAATGCCTTTGAAAGGAAATCCACAGGGGAGGAGTCTA

74_Campylopterus_curvipennis_Xil-4

GGATTATGGCTACTGCTACTTCTAGGATGGTTAGTAGGAGTAGTACGCAGGCGGTTAGAGCAGAGATTGCGGGTATGATG

GAGAATAGGACTGCGGTGGCTGTTGAGATGAGTTGGATTAGTAGGTGGCCTGCTGTTAGGTTAGCTGTGAGGCGGACTCC

TAGGGCTAGAGGGCGGATCAGGAGGCTGGTAGTTTCAATTATGATTAAGGCTGGGATTAGTGGAGTTGGGGTTCCTTCGG

GTAGGAGGTGGCCAAGTGAAGCGGATGGTTGGTTTCGGAGGCCTGTGAGTAGAGTGGCTAGTCATAGTGGGAAGGCTAGT

GCTAGGTTTATAGATAATTGTGTGGTAGGTGTGAATGTGTAGGGGAGTAGGCCTAGTAGGTTGATTGAGAGAAGGAAAAT

TATTAGTGATGACAGGATAAGGGCTCATTTGTGGCCTTTTTTGTTCAATGGGATTATCAGTTGTTTGGTGATTATATAGA

TGAATCACGATTGTAGGGTGGATGTTCGGTTGGTGATTCATCGGTTGTGGGGGGAGGGGAGTAGTAGGGCTGGGAATAAT

AGTGAGATGAGAATTAGGGGGATTCCTAGTAGGTGGGGGCTTATAAATTGGTCGAAGAAGCTTAGGTTCATGGTCAGGGT

CAGGATGAGGGTTTTGTGTTTGTTAGGGGTTTTTCTAGGGGGGAGTTAGTGGGCATGAATGAAAGGAGTTTGGGTTGGAT

GATTAGTGAAAAGGTCAGTCATGAAATAGTTATAATGAATAGTCATGGGTTTGGATTTAGTTGGGGCATATCATTAGGGA

GGGTTGGTAATCCTCTTTCTCTAGCTTAAAAGGCTAGTGCTGTTGCATAGCTTCCTAGTGGTTAAGATGATAGGAAAATT

AACATGATAAACATGATGATGGATGATGATTATTTTTTAATGTAATCTCGGTGTTGAATCTGAGTTAAAGTTAGATGATA

AAATGTGTGATGAAAATATCATGGATAGTATGGAAATTATAACTTAGAACTCCAGTGGTGTTGGTTAGAAAGTTAGAGGA

AGTGAAAAACTTGTAAAATATGTCCGGCAACCATAAATCAAAAAGCAACATAGCTTAGTAAGTATGTGAAAATACCATAA

CCAAATGCAAGACAAAGTGCATCAGTGTCAAAATGAGACTGAATTACACATGAAACCGTCTCATTCAGCAACTCTTGAAG

GAGAGAAAAGGCTCGGGAGCCACTGATGCTCAGGTCAAGAGATTGTGTGCTCCGCGTTAGCCACTGGAAGGGCCTATTGA

GATTACCCAAAAAAAAAGA-GAACCAACC-GCCTAAAAGAAGGGAACCATGCCGCGATTAAGAAAGGTATGAACTATAAA

GTACCAACCAAATGCCTTTGAAAGGAAATCCACAGGGGAGGAGTCTA

75_Campylopterus_curvipennis_Xil-5

GGATTATGGCTACTGCTACTTCTAGGATGGTTAGTAGGAGTAGTACGCAGGCGGTTAGAGCAGAGATTGCGGGTATGATG

GAGAATAGGACTGCGGTGGCTGTTGAGATGAGTTGGATTAGTAGGTGGCCTGCTGTTAGGTTAGCTGTGAGGCGGACTCC

TAGGGCTAGAGGGCGGATCAGGAGGCTGGTAGTTTCAATTATGATTAAGGCTGGGATTAGTGGAGTTGGGGTTCCTTCGG

GTAGGAGGTGGCCAAGTGAAGCGGATGGTTGGTTTCGGAGGCCTGTGAGTAGAGTGGCTAGTCATAGTGGGAAGGCTAGT

GCTAGGTTTATAGATAATTGTGTGGTAGGTGTGAATGTGTAGGGGAGTAGGCCTAGTAGGTTGATTGAGAGAAGGAAAAT

TATTAGTGATGACAGGATAAGGGCTCATTTGTGGCCTTTTTTGTTCAATGGGATTATCAGTTGTTTGGTGATTATATAGA

TGAATCACGATTGTAGGGTGGATGTTCGGTTGGTGATTCATCGGTTGTGGGGGGAGGGGAGTAGTAGGGCTGGGAATAAT

AGTGAGATGAGAATTAGGGGGATTCCTAGTAGGTGGGGGCTTATAAATTGGTCGAAGAAGCTTAGGTTCATGGTCAGGGT

CAGGATGAGGGTTTTGTGTTTGTTAGGGTTTTTTCTAGGGGGGAGTTAGTGGGCATGAATGAAAGGAGTTTGGGTTGGAT

GATTAGTGAAAAGGTCAGTCATGAAATAGTTATAATGAATAGTCATGGGTTTGGATTTAGTTGGGGCATATCATTAGGGA

GGGTTGGTAATCCTCTTTCTCTAGCTTAAAAGGCTAGTGCTGTTGCATAGCTTCCTAGTGGTTAAGATGATAGGAAAATT

AACATGATAAACATGATGATGGATGATGATTATTTTTTAATGTAATCTCGGTGTTGAATCTGGGTTAAAGTTAGATGATA

AAATGTGTGATGAAAATATCATGGATAGTATGGAAATTATAACTTAGAACTCCAGTGGTGTTGGTTAGAAAGTTAGAGGA

AGTGAAAAACTTGTAAAATATGTCCGGCAACCATAAATCAAAAAGCAACATAGCTTAGTAAGTATGTGAAAATACCATAA

CCAAATGCAAGACAAAGTGCATCAGTGTCAAAATGAGACTGAATTACACGTGAAACCGTCTCATTCAGCAACTCTTGAAG

GAGAGAAAAGGCTCGGGAGCCACTGATGCCCAGGTCAAGAGATTGTGTGCTCCGCGTTAGCCACTGGAAGGGCCTATTGA

GATTACCCAAAAAAAAAGA-GAACCAACC-GCCTAAAAGAAGGGAACCATGCCGCGATTAAGAAAGGTATGAACTATAAA

GTACCAACCAAATGCCTTTGAAAGGAAATCCACAGGGGAGGAGTCTA

76_Campylopterus_curvipennis_Xil-6

GGATTATGGCTACTGCTACTTCTAGGATGGTTAGTAGGAGTAGTACGCAGGCGGTTAGAGCAGAGATTGCGGGTATGATG

GAGAATAGGACTGCGGTGGCTGTTGAGATGAGTTGGATTAGTAGGTGGCCTGCTGTTAGGTTAGCTGTGAGGCGGACTCC

TAGGGCTAGAGGGCGGATCAGGAGGCTGGTAGTTTCAATTATGATTAAGGCTGGGATTAGTGGAGTTGGGGTTCCTTCGG

GTAGGAGGTGGCCAAGTGAAGCGGATGGTTGGTTTCGGAGGCCTGTGAGTAGAGTGGCTAGTCATAGTGGGAAGGCTAGT

GCTAGGTTTATAGATAATTGTGTGGTAGGTGTGAATGTGTAGGGGAGTAGGCCTAGTAGGTTGATTGAGAGAAGGAAAAT

TATTAGTGATGACAGGATAAGGGCTCATTTGTGGCCTTTTTTGTTCAATGGGATTATCAGTTGTTTGGTGATTATATAGA

TGAATCACGATTGTAGGGTGGATGTTCGGTTGGTGATTCATCGGTTGTGGGGGGAGGGGAGTAGTAGGGCTGGGAATAAT

AGTGAGATGAGAATTAGGGGGATTCCTAGTAGGTGGGGGCTTATAAATTGGTCGAAGAAGCTTAGGTTCATGGTCAGGGT

CAGGATGAGGGTTTTGTGTTTGTTAGGGTTTTTTCTAGGGGGGAGTTAGTGGGCATGAATGAAAGGAGTTTGGGTTGGAT

GATTAGTGAAAAGGTCAGTCATGAAATAGTTATAATGAATAGTCATGGGTTTGGATTTAGTTGGGGCATATCATTAGGGA

GGGTTGGTAATCCTCTTTCTCTAGCTTAAAAGGCTAGTGCTGTTGCATAGCTTCCTAGTGGTTAAGATGATAGGAAAATT

AACATGATAAACATGATGATGGATGATGATTATTTTTTAATGTAATCTCGGTGTTGAATCTGGGTTAAAGTTAGATGATA

AAATGTGTGATGAAAATATCATGGATAGTATGGAAATTATAACTTAGAACTCCAGTGGTGTTGGTTAGAAAGTTAGAGGA

AGTGAAAAACTTGTAAAATATGTCCGGCAACCATAAATCAAAAAGCAACATAGCTTAGTAAGTATGTGAAGATACCATAA

CCAAATGCAAGACAAAGTGCATCAGTGTCAAAATGAGACTGAATTACACATGAAACCGTCTCATTCAGCAACTCTTGAAG

GAGAGAAAAGGCTCGGGAGCCACTGATGCCCAGGTCAAGAGATTGTGTGCTCCGCGTTAGCCACTGGAAGGGCCTATTGA

GATTACCCAAAAAAAAAGA-GAACCAACC-GCCTAAAAGAAGGGAACCATGCCGCGATTAAGAAAGGTATGAACTATAAA

GTACCAACCAAATGCCTTTGAAAGGAAATCCACAGGGGAGGAGTCTA

77_Campylopterus_curvipennis_Xil-7

GGATTATGGCTACTGCTACTTCTAGGATGGTTAGTAGGAGTAGTACGCAGGCGGTTAGAGCAGAGATTGCGGGTATGATG

GAGAATAGGACTGCGGTGGCTGTTGAGATGAGTTGGATTAGTAGGTGGCCTGCTGTTAGGTTAGCTGTGAGGCGGACTCC

TAGGGCTAGAGGGCGGATCAGGAGGCTGGTAGTTTCAATTATGATTAAGGCTGGGATTAGTGGAGTTGGGGTTCCTTCGG

GTAGGAGGTGGCCAAGTGAAGCGGATGGTTGGTTTCGGAGGCCTGTGAGTAGAGTGGCTAGTCATAGTGGGAAGGCTAGT

GCTAGGTTTATAGATAATTGTGTGGTAGGTGTGAATGTGTAGGGGAGTAGGCCTAGTAGGTTGATTGAGAGAAGGAAAAT

TATTAGTGATGACAGGATAAGGGCTCATTTGTGGCCTTTTTTGTTCAATGGGATTATCAGTTGTTTGGTGATTATATAGA

TGAATCACGATTGTAGGGTGGATGTTCGGTTGGTGATTCATCGGTTGTGGGGGGAGGGGAGTAGTAGGGCTGGGAATAAT

AGTGAGATGAGAATTAGGGGGATTCCTAGTAGGTGGGGGCTTATAAATTGGTCGAAGAAGCTTAGGTTCATGGTCAGGGT

CAGGATGAGGGTTTTGTGTTTGTTAGGGTTTTTTCTAGGGGGGAGTTAGTGGGCATGAATGAAAGGAGTTTGGGTTGGAT

GATTAGTGAAAAGGTCAGTCATGAAATAGTTATAATGAATAGTCATGGGTTTGGATTTAGTTGGGGCATATCATTAGGGA

GGGTTGGTAATCCTCTTTCTCTAGCTTAAAAGGCTAGTGCTGTTGCATAGCTTCCTAGTGGTTAAGATGATAGGAAAATT

AACATGATAAACATGATGATGGATGATGATTATTTTTTAATGTAATCTCGGTGTTGAATCTGGGTTAAAGTTAGATGATA

AAATGTGTGATGAAAATATCATGGATAGTATGGAAATTATAACTTAGAACTCCAGTGGTGTTGGTTAGAAAGTTAGAGGA

AGTGAAAAACTTGTAAAATATGTCCGGCAACCATAAATCAAAAAGCAACATAGCTTAGTAAGTATGTGAAAATACCATAA

CCAAATGCAAGACAAAGTGCATCAGTGTCAAAATGAGACTGAATTACACATGAAACCGTCTCATTCAGCAACTCTTGAAG

GAGAGAAAAGGCTCGGGAGCCACTGATGCCCAGGTCAAGAGATTGTGTGCTCCGCGTTAGCCACTGGAAGGGCCTATTGA

GATTACCCAAAAAAAAAGA-GAACCAACC-GCCTAAAAGAAGGGAACCATGCCGCGATTAAGAAAGGTATGAACTATAAA

GTACCAACCAAATGCCTTTGAAAGGAAATCCACAGGGGAGGAGTCTA

78_Campylopterus_curvipennis_Xil-8

GGATTATGGCTACTGCTACTTCTAGGATGGTTAGTAGGAGTAGTACGCAGGCGGTTAGAGCAGAGATTGCGGGTATGATG

GAGAATAGGACTGCGGTGGCTGTTGAGATGAGTTGGATTAGTAGGTGGCCTGCTGTTAGGTTAGCTGTGAGGCGGACTCC

TAGGGCTAGAGGGCGGATCAGGAGGCTGGTAGTTTCAATTATGATTAAGGCTGGGATTAGTGGAGTTGGGGTTCCTTCGG

GTAGGAGGTGGCCAAGTGAAGCGGATGGTTGGTTTCGGAGGCCTGTGAGTAGAGTGGCTAGTCATAGTGGGAAGGCTAGT

GCTAGGTTTATAGATAATTGTGTGGTAGGTGTGAATGTGTAGGGGAGTAGGCCTAGTAGGTTGATTGAGAGAAGGAAAAT

TATTAGTGATGACAGGATAAGGGCTCATTTGTGGCCTTTTTTGTTCAATGGGATTATCAGTTGTTTGGTGATTATATAGA

TGAATCACGATTGTAGGGTGGATGTTCGGTTGGTGATTCATCGGTTGTGGGGGGAGGGGAGTAGTAGGGCTGGGAATAAT

AGTGAGATGAGAATTAGGGGGATTCCTAGTAGGTGGGGGCTTATAAATTGGTCGAAGAAGCTTAGGTTCATGGTCAGGGT

CAGGATGAGGGTTTTGTGTTTGTTAGGGTTTTTTCTAGGGGGGAGTTAGTGGGCATGAATGAAAGGAGTTTGGGTTGGAT

GATTAGTGAAAAGGTCAGTCATGAAATAGTTATAATGAATAGTCATGGGTTTGGATTTAGTTGGGGCATATCATTAGGGA

GGGTTGGTAATCCTCTTTCTCTAGCTTAAAAGGCTAGTGCTGTTGCATAGCTTCCTAGTGGTTAAGATGATAGGAAAATT

AACATGATAAACATGATGATGGATGATGATTATTTTTTAATGTAATCTCGGTGTTGAATCTGGGTTAAAGTTAGATGATA

AAATGTGTGATGAAAATATCATGGATAGTATGGAAATTATAACTTAGAACTCCAGTGGTGTTGGTTAGAAAGTTAGAGGA

AGTGAAAAACTTGTAAAATATGTCCGGCAACCATAAATCAAAAAGCAACATAGCTTAGTAAGTATGTGAAGATACCATAA

CCAAATGCAAGACAAAGTGCATCAGTGTCAAAATGAGACTGAATTACACATGAAACCGTCTCATTCAGCAACTCTTGAAG

GAGAGAAAAGGCTCGGGAGCCACTGATGCCCAGGTCAAGAGATTGTGTGCTCCGCGTTAGCCACTGGAAGGGCCTATTGA

GATTACCCAAAAAAAAAGA-GAACCAACC-GCCTAAAAGAAGGGAACCATGCCGCGATTAAGAAAGGTATGAACTATAAA

GTACCAACCAAATGCCTTTGAAAGGAAATCCACAGGGGAGGAGTCTA

79_Campylopterus_curvipennis_Coap-1

GGATTATGGCTACTGCTACTTCTAGGATGGTTAGTAGGAGTAGTACGCAGGCGGTTAGAGCAGAGATTGCGGGTATGATG

GAGAATAGGACTGCGGTGGCTGTTGAGATGAGTTGGATTAGTAGGTGGCCTGCTGTTAGGTTAGCTGTGAGGCGGACTCC

TAGGGCTAGAGGGCGGATCAGGAGGCTGGTAGTTTCAATTATGATTAAGGCTGGGATTAGTGGAGTTGGGGTTCCTTCGG

GTAGGAGGTGGCCAAGTGAAGCGGATGGTTGGTTTCGGAGGCCTGTGAGTAGAGTGGCTAGTCATAGTGGGAAGGCTAGT

GCTAGGTTTATAGATAATTGTGTGGTAGGTGTGAATGTGTAGGGGAGTAGGCCTAGTAGGTTGATTGAGAGAAGGAAAAT

TATTAGTGATGACAGGATAAGGGCTCATTTGTGGCCTTTTTTGTTCAATGGGATTATCAGTTGTTTGGTGATTATATAGA

TGAATCACGATTGTAGGGTGGATGTTCGGTTGGTGATTCATCGGTTGTGGGGGGAGGGGAGTAGTAGGGCTGGGAATAAT

AGTGAGATGAGAATTAGGGGGATTCCTAGTAGGTGGGGGCTTATAAATTGGTCGAAGAAGCTTAGGTTCATGGTCAGGGT

CAGGATGAGGGTTTTGTGTTTGTTAGGGGTTTTTCTAGGGGGGAGTTAGTGGGCATGAATGAAAGGAGTTTGGGTTGGAT

GATTAGTGAAAAGGTCAGTCATGAAATAGTTATAATGAATAGTCATGGGTTTGGATTTAGTTGGGGCATATCATTAGGGA

GGGTTGGTAATCCTCTTTCTCTAGCTTAAAAGGCTAGTGCTGTTGCATAGCTTCCTAGTGGTTAAGATGATAGGAAAATT

AACATGATAAACATGATGATGGATGATGATTATTTTTTAATGTAATCTCGGTGTTGAATCTGGGTTAAAGTTAGATGATA

AAATGTGTGATGAAAATATCATGGATAGTATGGAAATTATAACTTAGAACTCCAGTGGTGTTGGTTAGAAAGTTAGAGGA

AGTGAAAAACTTGTAAAATATGTCCGGCAACCATAAATCAAAAAGCAACATAGCTTAGTAGGTATGTGAAAATACCATAA

CCAAATGCAAGACAAAGTGCATCAGTGTCAAAATGAGACTGAATTACACATGAAACCGTCTCATTCAGCAACTCTTGAAG

GAGAGAAAAGGCCCGGGAGCCACTGATGCTCAGGTCAAGAGATTGTGTGCTCCGCGTTAGCCACTGGAAGGGCCTATTGA

GATTACCCAAAAAAAAAGA-GAACCAACC-GCCTAAAAGAAGGGAACCATGCCGCGATTAAGAAAGGTATGAACTATAAA

GTACCAACCAAATGCCTTTGAAAGGAAATCCACAGGGGAGGAGTCTA

80_Campylopterus_curvipennis_Coap-2

GGATTATGGCTACTGCTACTTCTAGGATGGTTAGTAGGAGTAGTACGCAGGCGGTTAGAGCAGAGATTGCGGGTATGATG

GAGAATAGGACTGCGGTGGCTGTTGAGATGAGTTGGATTAGTAGGTGGCCTGCTGTTAGGTTAGCTGTGAGGCGGACTCC

TAGGGCTAGAGGGCGGATCAGGAGGCTGGTAGTTTCAATTATGATTAAGGCTGGGATTAGTGGAGTTGGGGTCCCTTCGG

GTAGGAGGTGGCCAAGTGAAGCGGATGGTTGGTTTCGGAGGCCTGTGAGTAGAGTGGCTAGTCATAGTGGGAAGGCTAGT

GCTAGGTTTATAGATAATTGTGTGGTAGGTGTGAATGTGTAGGGGAGTAGGCCTAGTAGGTTGATTGAGAGAAGGAAAAT

TATTAGTGATGACAGGATAAGGGCTCATTTGTGGCCTTTTTTGTTCAATGGGATTATCAGTTGTTTGGTGATTATATAGA

TGAATCACGATTGTAGGGTGGATGTTCGGTTGGTGATTCATCGGTTGTGGGGGGAGGGGAGTAGTAGGGCTGGGAATAAT

AGTGAGATGAGAATTAGGGGGATTCCTAGTAGGTGGGGGCTTATAAATTGGTCGAAGAAGCTTAGGTTCATGGTCAGGGT

CAGGATGAGGGTTTTGTGTTTGTTAGGGTTTTTTCTAGGGGGGAGTTAGTGGGCATGAATGAAAGGAGTTTGGGTTGGAT

GATTAGTGAAAAGGTCAGTCATGAAATAGTTATAATGAATAGTCATGGGTTTGGATTTAGTTGGGGCATATCATTAGGGA

GGGTTGGTAATCCTCTTTCTCTAGCTTAAAAGGCTAGTGCTGTTGCATAGCTTCCTAGTGGTTAAGATGATAGGAAAATT

AACATGATAAACATGATGATGGATGATGATTATTTTTTAATGTAATCTCGGTGTTGAATCTGGGTTAAAGTTAGATGATA

AAATGTGTGATGAAAATATCATGGATAGTATGGAAATTATAACTTAGAACTCCAGTGGTGTTGGTTAGAAAGTTAGAGGA

AGTGAAAAACTTGTAAAATATGTCCGGCAACCATAAATCAAAAAGCAACATAGCTTAGTAAGTATGTGAAAATACCATAA

CCAAATGCAAGACAAAGTGCATCAGTGTCAAAATGAGACTGAATTACACACGAAACCGTCTCATTCAGCAACTCTTGAAG

GAGAGAAAAGGCTCGGGAGCCACTGATGCCCAGGTCAAGAGATTGTGTGCTCCGCGTTAGCCACTGGAAGGGCCTATTGA

GATTACCCAAAAAAAAAGA-GAACCAACC-GCCTAAAAGAAGGGAACCATGCCGCGATTAAGAAAGGTATGAACTATAAA

GTACCAACCAAATGCCTTTGAAAGGAAATCCACAGGGGAGGAGTCTA

81_Campylopterus_curvipennis_Coap-3

GGATTATGGCTACTGCTACTTCTAGGATGGTTAGTAGGAGTAGTACGCAGGCGGTTAGAGCAGAGATTGCGGGTATGATG

GAGAATAGGACTGCGGTGGCTGTTGAGATGAGTTGGATTAGTAGGTGGCCTGCTGTTAGGTTAGCTGTGAGGCGGACTCC

TAGGGCTAGAGGGCGGATCAGGAGGCTGGTAGTTTCAATTATGATTAAGGCTGGGATTAGTGGAGTTGGGGTTCCTTCGG

GTAGGAGGTGGCCAAGTGAAGCGGATGGTTGGTTTCGGAGGCCTGTGAGTAGAGTGGCTAGTCATAGTGGGAAGGCTAGT

GCTAGGTTTATAGATAATTGTGTGGTAGGTGTGAATGTGTAGGGGAGTAGGCCTAGTAGGTTGATTGAGAGAAGGAAAAT

TATTAGTGATGACAGGATAAGGGCTCATTTGTGGCCTTTTTTGTTCAATGGGATTATCAGTTGTTTGGTGATTATATAGA

TGAATCACGATTGTAGGGTGGATGTTCGGTTGGTGATTCATCGGTTGTGGGGGGAGGGGAGTAGTAGGGCTGGGAATAAT

AGTGAGATGAGAATTAGGGGGATTCCTAGTAGGTGGGGGCTTATAAATTGGTCGAAGAAGCTTAGGTTCATGGTCAGGGT

CAGGATGAGGGTTTTGTGTTTGTTAGGGTTTTTTCTAGGGGGGAGTTAGTGGGCATGAATGAAAGGAGTTTGGGTTGGAT

GATTAGTGAAAAGGTCAGTCATGAAATAGTTATAATGAATAGTCATGGGTTTGGATTTAGTTGGGGCATATCATTAGGGA

GGGTTGGTAATCCTCTTTCTCTAGCTTAAAAGGCTAGTGCTGTTGCATAGCTTCCTAGTGGTTAAGATGATAGGAAAATT

AACATGATAAACATGATGATGGATGATGATTATTTTTTAATGTAATCTCGGTGTTGAATCTGGGTTAAAGTTAGATGATA

AAATGTGTGATGAAAATATCATGGATAGTATGGAAATTATAACTTAGAACTCCAGTGGTGTTGGTTAGAAAGTTAGAGGA

AGTGAAAAACTTGTAAAATATGTCCGGCAACCATAAATCAAAAAGCAACATAGCTTAGTAAGTATGTGAAGATACCATAA

CCAAATGCAAGACAAAGTGCATCAGTGTCAAAATGAGACTGAATTACACATGAAACCGTCTCATTCAGCAACTCTTGAAG

GAGAGAAAAGGCTCGGGAGCCACTGATGCCCAGGTCAAGAGATTGTGTGCTCCGCGTTAGCCACTGGAAGGGCCTATTGA

GATTACCCAAAAAAAAAGA-GAACCAACC-GCCTAAAAGAAGGGAACCATGCCGCGATTAAGAAAGGTATGAACTATAAA

GTACCAACCAAATGCCTTTGAAAGGAAATCCACAGGGGAGGAGTCTA

82_Campylopterus_curvipennis_Coap-4

GGATTATGGCTACTGCTACTTCTAGGATGGTTAGTAGGAGTAGTACGCAGGCGGTTAGAGCAGAGATTGCGGGTATGATG

GAGAATAGGACTGCGGTGGCTGTTGAGATGAGTTGGATTAGTAGGTGGCCTGCTGTTAGGTTAGCTGTGAGGCGGACTCC

TAGGGCTAGAGGGCGGATCAGGAGGCTGGTAGTTTCAATTATGATTAAGGCTGGGATTAGTGGAGTTGGGGTTCCTTCGG

GTAGGAGGTGGCCAAGTGAAGCGGATGGTTGGTTTCGGAGGCCTGTGAGTAGAGTGGCTAGTCATAGTGGGAAGGCTAGT

GCTAGGTTTATAGATAATTGTGTGGTAGGTGTGAATGTGTAGGGGAGTAGGCCTAGTAGGTTGATTGAGAGAAGGAAAAT

TATTAGTGATGACAGGATAAGGGCTCATTTGTGGCCTTTTTTGTTCAATGGGATTATCAGTTGTTTGGTGATTATATAGA

TGAATCACGATTGTAGGGTGGATGTTCGGTTGGTGATTCATCGGTTGTGGGGGGAGGGGAGTAGTAGGGCTGGGAATAAT

AGTGAGATGAGAATTAGGGGGATTCCTAGTAGGTGGGGGCTTATAAATTGGTCGAAGAAGCTTAGGTTCATGGTCAGGGT

CAGGATGAGGGTTTTGTGTTTGTTAGGGTTTTTTCTAGGGGGGAGTTAGTGGGCATGAATGAAAGGAGTTTGGGTTGGAT

GATTAGTGAAAAGGTCAGTCATGAAATAGTTATAATGAATAGTCATGGGTTTGGATTTAGTTGGGGCATATCATTAGGGA

GGGTTGGTAATCCTCTTTCTCTAGCTTAAAAGGCTAGTGCTGTTGCATAGCTTCCTAGTGGTTAAGATGATAGGAAAATT

AACATGATAAACATGATGATGGATGATGATTATTTTTTAATGTAATCTCGGTGTTGAATCTGGGTTAAAGTTAGATGATA

AAATGTGTGATGAAAATATCATGGATAGTATGGAAATTATAACTTAGAACTCCAGTGGTGTTGGTTAGAAAGTTAGAGGA

AGTGAAAAACTTGTAAAATATGTCCGGCAACCATAAATCAAAAAGCAACATAGCTTAGTAAGTATGTGAAAATACCATAA

CCAAATGCAAGACAAAGTGCATCAGTGTCAAAATGAGACTGAATTACACATGAAACCGTCTCATTCAGCAACTCCTGAAG

GAGAGAAAAGGCTCGGGAGCCACTGATGCCCAGGTCAAGAGATTGTGTGCTCCGCGTTAGCCACTGGAAGGGCCTATTGA

GATTACCCAAAAAAAAAGA-GAACCAACC-GCCTAAAAGAAGGGAACCATGCCGCGATTAAGAAAGGTATGAACTATAAA

GTACCAACCAAATGCCTTTGAAAGGAAATCCACAGGGGAGGAGTCTA

83_Campylopterus_curvipennis_Coap-5

GGATTATGGCTACTGCTACTTCTAGGATGGTTAGTAGGAGTAGTACGCAGGCGGTTAGAGCAGAGATTGCGGGTATGATG

GAGAATAGGACTGCGGTGGCTGTTGAGATGAGTTGGATTAGTAGGTGGCCTGCTGTTAGGTTAGCTGTGAGGCGGACTCC

TAGGGCTAGAGGGCGGATCAGGAGGCTGGTAGTTTCAATTATGATTAAGGCTGGGATTAGTGGAGTTGGGGTTCCTTCGG

GTAGGAGGTGGCCAAGTGAAGCGGATGGTTGGTTTCGGAGGCCTGTGAGTAGAGTGGCTAGTCATAGTGGGAAGGCTAGT

GCTAGGTTTATAGATAATTGTGTGGTAGGTGTGAATGTGTAGGGGAGTAGGCCTAGTAGGTTGATTGAGAGAAGGAAAAT

TATTAGTGATGACAGGATAAGGGCTCATTTGTGGCCTTTTTTGTTCAATGGGATTATCAGTTGTTTGGTGATTATATAGA

TGAATCACGATTGTAGGGTGGATGTTCGGTTGGTGATTCATCGGTTGTGGGGGGAGGGGAGTAGTAGGGCTGGGAATAAT

AGTGAGATGAGAATTAGGGGGATTCCTAGTAGGTGGGGGCTTATAAATTGGTCGAAGAAGCTTAGGTTCATGGTCAGGGT

CAGGATGAGGGTTTTGTGTTTGTTAGGGTTTTTTCTAGGGGGGAGTTAGTGGGCATGAATGAAAGGAGTTTGGGTTGGAT

GATTAGTGAAAAGGTCAGTCATGAAATAGTTATAATGAATAGTCATGGGTTTGGATTTAGTTGGGGCATATCATTAGGGA

GGGTTGGTAATCCTCTTTCTCTAGCTTAAAAGGCTAGTGCTGTTGCATAGCTTCCTAGTGGTTAAGATGATAGGAAAATT

AACATGATAAACATGATGATGGATGATGATTATTTTTTAATGTAATCTCGGTGTTGAATCTGGGTTAAAGTTAGATGATA

AAATGTGTGATGAAAATATCATGGATAGTATGGAAATTATAACTTAGAACTCCAGTGGTGTTGGTTAGAAAGTTAGAGGA

AGTGAAAAACTTGTAAAATATGTCCGGCAACCATAAATCAAAAAGCAACATAGCTTAGTAAGTATGTGAAAATACCATAA

CCAAATGCAAGACAAAGTGCATCAGTGTCAAAATGAGACTGAATTACACATGAAACCGTCTCATTCAGCAACTCTTGAAG

GAGAGAAAAGGCTCGGGAGCCACTGATGCCCAGGTCAAGAGATTGTGTGCTCCGCGTTAGCCACTGGAAGGGCCTATTGA

GATTACCCAAAAAAAAAGA-GAACCAACC-GCCTAAAAGAAGGGAACCATGCCGCGATTAAGAAAGGTATGAACTATAAA

GTACCAACCAAATGCCTTTGAAAGGAAATCCACAGGGGAGGAGTCTA

84_Campylopterus_curvipennis_Coap-6

GGATTATGGCTACTGCTACTTCTAGGATGGTTAGTAGGAGTAGTACGCAGGCGGTTAGAGCAGAGATTGCGGGTATGATG

GAGAATAGGACTGCGGTGGCTGTTGAGATGAGTTGGATTAGTAGGTGGCCTGCTGTTAGGTTAGCTGTGAGGCGGACTCC

TAGGGCTAGAGGGCGGATCAGGAGGCTGGTAGTTTCAATTATGATTAAGGCTGGGATTAGTGGAGTTGGGGTTCCTTCGG

GTAGGAGGTGGCCAAGTGAAGCGGATGGTTGGTTTCGGAGGCCTGTGAGTAGAGTGGCTAGTCATAGTGGGAAGGCTAGT

GCTAGGTTTATAGATAATTGTGTGGTAGGTGTGAATGTGTAGGGGAGTAGGCCTAGTAGGTTGATTGAGAGAAGGAAAAT

TATTAGTGATGACAGGATAAGGGCTCATTTGTGGCCTTTTTTGTTCAATGGGATTATCAGTTGTTTGGTGATTATATAGA

TGAATCACGATTGTAGGGTGGATGTTCGGTTGGTGATTCATCGGTTGTGGGGGGAGGGGAGTAGTAGGGCTGGGAATAAT

AGTGAGATGAGAATTAGGGGGATTCCTAGTAGGTGGGGGCTTATAAATTGGTCGAAGAAGCTTAGGTTCATGGTCAGGGT

CAGGATGAGGGTTTTGTGTTTGTTAGGGGGTTTTCTAGGGGGGAGTTAGTGGGCATGAATGAAAGGAGTTTGGGTTGGAT

GATTAGTGAAAAGGTCAGTCATGAAATAGTTATAATGAATAGTCATGGGTTTGGATTTAGTTGGGGCATATCATTAGGGA

GGGTTGGTAATCCTCTTTCTCTAGCTTAAAAGGCTAGTGCTGTTGCATAGCTTCCTAGTGGTTAAGATGATAGGAAAATT

AACATGATAAACATGATGATGGATGATGATTATTTTTTAATGTAATCTCGGTGTTGAATCTGGGTTAAAGTTAGATGATA

AAATGTGTGATGAAAATATCATGGATAGTATGGAAATTATAACTTAGAACTCCAGTGGTGTTGGTTAGAAAGTTAGAGGA

AGTGAAAAACTTGTAAAATATGTCCGGCAACCATAAATCAAAAAGCAACATAGCTTAGTAAGTATGTGAAAATACCATAA

CCAAATGCAAGACAAAGTGCATCAGTGTCAAAATGAGACTGAATTACACGTGAAACCGTCTCATTCAGCAACTCTTGAAG

GAGAGAAAAGGCTCGGGAGCCACTGATGCCCAGGTCAAGAGATTGTGTGCTCCGCGTTAGCCACTGGAAGGGCCTATTGA

GATTACCCAAAAAAAAAGA-GAACCAACC-GCCTAAAAGAAGGGAACCATGCCGCGATTAAGAAAGGTATGAACTATAAA

GTACCAACCAAATGCCTTTGAAAGGAAATTCACAGGGGAGGAGTCTA

85_Campylopterus_curvipennis_Risc-1

GGATTATGGCTACTGCTACTTCTAGGATGGTTAGTAGGAGTAGTACGCAGGCGGTTAGGGCAGAGATTGCGGGTATGATG

GAGAATAGGACTGCGGTGGCTGTTGAGATGAGTTGGATTAGTAGGTGGCCTGCTGTTAGGTTAGCTGTGAGGCGGACTCC

TAGGGCTAGAGGGCGGATCAGGAGGCTGGTAGTTTCAATTATGATTAAGGCTGGGATTAGTGGAGTTGGGGTTCCTTCGG

GTAGGAGGTGGCCAAGTGAAGCGGATGGTTGGTTTCGGAGGCCTGTGAGTAGAGTGGCTAGTCATAGTGGGAAGGCTAGT

GCTAGGTTTATAGATAATTGTGTGGTAGGTGTGAATGTGTAGGGGAGTAGGCCTAGTAGGTTGATTGAGAGAAGGAAAAT

TATTAGTGATGACAGGATAAGGGCTCATTTGTGGCCTTTTTTGTTCAATGGGATTATCAGTTGTTTGGTGATTATATAGA

TGAATCACGATTGTAGGGTGGATGTTCGGTTGGTGATTCATCGGTTGTGGGGGGAGGGGAGTAGTAGGGCTGGGAATAAT

AGTGAGATGAGAATTAGGGGGATTCCTAGTAGGTGGGGGCTTATAAATTGGTCGAAGAAGCTTAGGTTCATGGTCAGGGT

CAGGATGAGGGTTTTGTGTTTGTTAGGGTTTTTTCTAGGGGGGAGTTAGTGGGCATGAATGAAAGGAGTTTGGGTTGGAT

GATTAGTGAAAAGGTCAGTCATGAAATAGTTATAATGAATAGTCATGGGTTTGGATTTAGTTGGGGCATATCATTAGGGA

GGGTTGGTAATCCTCTTTCTCTAGCTTAAAAGGCTAGTGCTGTTGCATAGCTTCCTAGTGGTTAAGATGATAGGAAAATT

AACATGATAAACATGATGATGGATGATGATTATTTTTTAATGTAATCTCGGTGTTGAATCTGGGTTAAAGTTAGATGATA

AAATGTGTGATGAAAATATCATGGATAGTATGGAAATTATAACTTAGAACTCCAGTGGTGTTGGTTAGAAAGTTAGAGGA

AGTGAAAAACTTGTAAAATATGTCCGGCAACCATAAATCAAAAAGCAACATAGCTTAGTAAGTATGTGAAAATACCATAA

CCAAATGCAAGACAAAGTGCATCAGTGTCAAAATGAGACTGAATTACACATGAAACCGTCTCATTCAGCAACTCTTGAAG

GAGAGAAAAGGCTCGGGAGCCACTGATGCCCAGGTCAAGAGATTGTGTGCTCCGCGTTAGCCACTGGAAGGGCCTATTGA

GATTACCCAAAAAAAAAGA-GAACCAACC-GCCTAAAAGAAGGGAACCATGCCGCGATTAAGAAAGGTATGAACTATAAA

GTACCAACCAAATGCCTTTGAAAGGAAATCCACAGGGGAGGAGTCTA

86_Campylopterus_curvipennis_Risc-2

GGATTATGGCTACTGCTACTTCTAGGATGGTTAGTAGGAGTAGTACGCAGGCGGTTAGAGCAGAGATTGCGGGTATGATG

GAGAATAGGACTGCGGTGGCTGTTGAGATGAGTTGGATTAGTAGGTGGCCTGCTGTTAGGTTAGCTGTGAGGCGGACTCC

TAGGGCTAGAGGGCGGATCAGGAGGCTGGTAGTTTCAATTATGATTAAGGCTGGGATTAGTGGAGTTGGGGTTCCTTCGG

GTAGGAGGTGGCCAAGTGAAGCGGATGGTTGGTTTCGGAGGCCTGTGAGTAGAGTGGCTAGTCATAGTGGGAAGGCTAGT

GCTAGGTTTATAGATAATTGTGTGGTAGGTGTGAATGTGTAGGGGAGTAGGCCTAGTAGGTTGATTGAGAGAAGGAAAAT

TATTAGTGATGACAGGATAAGGGCTCATTTGTGGCCTTTTTTGTTCAATGGGATTATCAGTTGTTTGGTGATTATATAGA

TGAATCACGATTGTAGGGTGGATGTTCGGTTGGTGATTCATCGGTTGTGGGGGGAGGGGAGTAGTAGGGCTGGGAATAAT

AGTGAGATGAGAATTAGGGGGATTCCTAGTAGGTGGGGGCTTATAAATTGGTCGAAGAAGCTTAGGTTCATGGTCAGGGT

CAGGATGAGGGTTTTGTGTTTGTTAGGGTTTTTTCTAGGGGGGAGTTAGTGGGCATGAATGAAAGGAGTTTGGGTTGGAT

GATTAGTGAAAAGGTCAGTCATGAAATAGTTATAATGAATAGTCATGGGTTTGGATTTAGTTGGGGCATATCATTAGGGA

GGGTTGGTAATCCTCTTTCTCTAGCTTAAAAGGCTAGTGCTGTTGCATAGCTTCCTAGTGGTTAAGATGATAGGAAAATT

AACATGATAAACATGATGATGGATGATGATTATTTTTTAATGTAATCTCGGTGTTGAATCTGGGTTAAAGTTAGATGATA

AAATGTGTGATGAAAATATCATGGATAGTATGGAAATTATAACTTAGAACTCCAGTGGTGTTGGTTAGAAAGTTAGAGGA

AGTGAAAAACTTGTAAAATATGTCCGGCAACCATAAATCAAAAAGCAACATAGCTTAGTAAGTATGTGAAAATACCATAA

CCAAATGCAAGACAAAGTGCATCAGTGTCAAAATGAGACTGAATTACACATGAAACCGTCTCATTCAGCAACTCTTGAAG

GAGAGAAAAGGCTCGGGAGCCACTGATGCCCAGGTCAAGAGATTGTGTGCTCCGCGTTAGCCACTGGAAGGGCCTATTGA

GATTACCCAAAAAAAAAGA-GAACCAACC-GCCTAAAAGAAGGGAACCATGCCGCGATTAAGAAAGGTATGAACTATAAA

GTACCAACCAAATGCCTTTGAAAGGAAATCCACAGGGGAGGAGTCTA

87_Campylopterus_curvipennis_Bris-1

GGATTATGGCTACTGCTACTTCTAGGATGGTTAGTAGGAGTAGTACGCAGGCGGTTAGAGCAGAGATTGCGGGTATGATG

GAGAATAGGACTGCGGTGGCTGTTGAGATGAGTTGGATTAGTAGGTGGCCTGCTGTTAGGTTAGCTGTGAGGCGGACTCC

TAGGGCTAGAGGGCGGATCAGGAGGCTGGTAGTTTCAATTATGATTAAGGCTGGGATTAGTGGAGTTGGGGTTCCTTCGG

GTAGGAGGTGGCCAAGTGAAGCGGATGGTTGGTTTCGGAGGCCTGTGAGTAGAGTGGCTAGTCATAGTGGGAAGGCTAGT

GCTAGGTTTATAGATAATTGTGTGGTAGGTGTGAATGTGTAGGGGAGTAGGCCTAGTAGGTTGATTGAGAGAAGGAAAAT

TATTAGTGATGACAGGATAAGGGCTCATTTGTGGCCTTTTTTGTTCAATGGGATTATCAGTTGTTTGGTGATTATATAGA

TGAATCACGATTGTAGGGTGGATGTTCGGTTGGTGATTCATCGGTTGTGGGGGGAGGGGAGTAGTAGGGCTGGGAATAAT

AGTGAGATGAGAATTAGGGGGATTCCTAGTAGGTGGGGGCTTATAAATTGGTCGAAGAAGCTTAGGTTCATGGTCAGGGT

CAGGATGAGGGTTTTGTGTTTGTTAGGGTTTTTTCTAGGGGGGAGTTAGTGGGCATGAATGAAAGGAGTTTGGGTTGGAT

GATTAGTGAAAAGGTCAGTCATGAAATAGTTATAATTAATAGTCATGGGTTTGGATTTAGTTGGGGCATATCATTAGGGA

GGGTTGGTAATCCTCTTTCTCTAGCTTAAAAGGCTAGTGCTGTTGCATAGCTTCCTAGTGGTTAAGATGATAGGAAAATT

AACATGATAAACATGATGATGGATGATGATTATTTTTTAATGTAATCTCGGTGTTGAATCTGGGTTAAAGTTAGATGATA

AAATGTGTGATGAAAATATCATGGATAGTATGGAAATTATAACTTAGAACTCCAGTGGTGTTGGTTAGAAAGTTAGAGGA

AGTGAAAAACTTGTAAAATATGTCCGGCAACCATAAATCAAAAAGCAACATAGCTTAGTAAGTATGTGAAAATACCATAA

CCAAATGCAAGACAAAGTGCATCAGTGTCAAAATGAGACTGAATTACACATGAAACCGTCTCATTCAGCAACTCTTGAAG

GAGAGAAAAGGCTCGGGAGCCACTGATGCCCAGGTCAAGAGATTGTGTGCTCCGCGTTAGCCACTGGAAGGGCCTATTGA

GATTACCCAAAAAAAAAGA-GAACCAACC-GCCTAAAAGAAGGGAACCATGCCGCGATTAAGAAAGGTATGAACTATAAA

GTACCAACCAAATGCCTTTGAAAGGAAATCCACAGGGGAGGAGTCTA

88_Campylopterus_curvipennis_Bris-2

GGATTATGGCTACTGCTACTTCTAGGATGGTTAGTAGGAGTAGTACGCAGGCGGTTAGAGCAGAGATTGCGGGTATGATG

GAGAATAGGACTGCGGTGGCTGTTGAGATGAGTTGGATTAGTAGGTGGCCTGCTGTTAGGTTAGCTGTGAGGCGGACTCC

TAGGGCTAGAGGGCGGATCAGGAGGCTGGTAGTTTCAATTATGATTAAGGCTGGGATTAGTGGAGTTGGGGTTCCTTCGG

GTAGGAGGTGGCCAAGTGAAGCGGATGGTTGGTTTCGGAGGCCTGTGAGTAGAGTGGCTAGTCATAGTGGGAAGGCTAGT

GCTAGGTTTATAGATAATTGTGTGGTAGGTGTGAATGTGTAGGGGAGTAGGCCTAGTAGGTTGATTGAGAGAAGGAAAAT

TATTAGTGATGACAGGATAAGGGCTCATTTGTGGCCTTTTTTGTTCAATGGGATTATCAGTTGTTTGGTGATTATATAGA

TGAATCACGATTGTAGGGTGGATGTTCGGTTGGTGATTCATCGGTTGTGGGGGGAGGGGAGTAGTAGGGCTGGGAATAAT

AGTGAGATGAGAATTAGGGGGATTCCTAGTAGGTGGGGGCTTATAAATTGGTCGAAGAAGCTTAGGTTCATGGTCAGGGT

CAGGATGAGGGTTTTGTGTTTGTTAGGGTTTTTTCTAGGGGGGAGTTAGTGGGCATGAATGAAAGGAGTTTGGGTTGGAT

GATTAGTGAAAAGGTCAGTCATGAAATAGTTATAATGAATAGTCATGGGTTTGGATTTAGTTGGGGCATATCATTAGGGA

GGGTTGGTAATCCTCTTTCTCTAGCTTAAAAGGCTAGTGCTGTTGCATAGCTTCCTAGTGGTTAAGATGATAGGAAAATT

AACATGATAAACATGATGATGGATGATGATTATTTTTTAATGTAATCTCGGTGTTGAATCTGGGTTAAAGTTAGATGATA

AAATGTGTGATGAAAATATCATGGATAGTATGGAAATTATAACTTAGAACTCCAGTGGTGTTGGTTAGAAAGTTAGAGGA

AGTGAAAAACTTGTAAAATATGTCCGGCAACCATAAATCAAAAAGCAACATAGCTTAGTAAGTATGTGAAAATACCATAA

CCAAATGCAAGACAAAGTGCATCAGTGTCAAAATGAGACTGAATTACACATGAAACCGTCTCATTCAGCAACTCTTGAAG

GAGAGAAAAGGCTCGGGAGCCACTGATGCCCAGGTCAAGAGATTGTGTGCTCCGCGTTAGCCACTGGAAGGGCCTATTGA

GATTACCCAAAAAAAAAGA-GAACCAACC-GCCTAAAAGAAGGGAACCATGCCGCGATTAAGAAAGGTATGAACTATAAA

GTACCAACCAAATGCCTTTGAAAGGAAATCCACAGGGGAGGAGTCTA

89_Campylopterus_curvipennis_Bris-3

GGATTATGGCTACTGCTACTTCTAGGATGGTTAGTAGGAGTAGTACGCAGGCGGTTAGAGCAGAGATTGCGGGTATGATG

GAGAATAGGACTGCGGTGGCTGTTGAGATGAGTTGGATTAGTAGGTGGCCTGCTGTTAGGTTAGCTGTGAGGCGGACTCC

TAGGGCTAGAGGGCGGATCAGGAGGCTGGTAGTTTCAATTATGATTAAGGCTGGGATTAGTGGAGTTGGGGTTCCTTCGG

GTAGGAGGTGGCCAAGTGAAGCGGATGGTTGGTTTCGGAGGCCTGTGAGTAGAGTGGCTAGTCATAGTGGGAAGGCTAGT

GCTAGGTTTATAGATAATTGTGTGGTAGGTGTGAATGTGTAGGGGAGTAGGCCTAGTAGGTTGATTGAGAGAAGGAAAAT

TATTAGTGATGACAGGATAAGGGCTCATTTGTGGCCTTTTTTGTTCAATGGGATTATCAGTTGTTTGGTGATTATATAGA

TGAATCACGATTGTAGGGTGGATGTTCGGTTGGTGATTCATCGGTTGTGGGGGGAGGGGAGTAGTAGGGCTGGGAATAAT

AGTGAGATGAGAATTAGGGGGATTCCTAGTAGGTGGGGGCTTATAAATTGGTCGAAGAAGCTTAGGTTCATGGTCAGGGT

CAGGATGAGGGTTTTGTGTTTGTTAGGGGTTTTTCTAGGGGGGAGTTAGTGGGCATGAATGAAAGGAGTTTGGGTTGGAT

GATTAGTGAAAAGGTCAGTCATGAAATAGTTATAATGAATAGTCATGGGTTTGGATTTAGTTGGGGCATATCATTAGGGA

GGGTTGGTAATCCTCTTTCTCTAGCTTAAAAGGCTAGTGCTGTTGCATAGCTTCCTAGTGGTTAGGATGATAGGAAAATT

AACATGATAAACATGATGATGGATGATGATTATTTTTTAATGTAATCTCGGTGTTGAATCTGGGTTAAAGTTAGATGATA

AAATGTGTGATGAAAATATCATGGATAGTATGGAAATTATAACTTAGAACTCCAGTGGTGTTGGTTAGAAAGTTAGAGGA

AGTGAAAAACTTGTAAAATATGTCCGGCAACCATAAATCAAAAAGCAACATAGCTTAGTAAGTATGTGAAAATACCATAA

CCAAATGCAAGACAAAGTGCATCAGTGTCAAAATGAGACTGAATTACACATGAAACCGTCTCATTCAGCAATTCTTGAAG

GAGAGAAAAGGCCCGGGAGCCACTGATGCCCAGGTCAAGAGATTGTGTGCTCCGCGTTAGCCACTGGAAGGGCCTATTGA

GATTACCCAAAAAAAAAGA-GAACCAACC-GCCTAAAAGAAGGGAACCATGCCGCGATTAAGAAAGGTATGAACTATAAA

GTACCAACCAAATGCCTTTGAAAGGAAATCCACAGGGGAGGAATCTA

90_Campylopterus_curvipennis_Bris-4

GGATTATGGCTACTGCTACTTCTAGGATGGTTAGTAGGAGTAGTACGCAGGCGGTTAGAGCAGAGATTGCGGGTATGATG

GAGAATAGGACTGCGGTGGCTGTTGAGATGAGTTGGATTAGTAGGTGGCCTGCTGTTAGGTTAGCTGTGAGGCGGACTCC

TAGGGCTAGAGGGCGGATCAGGAGGCTGGTAGTTTCAATTATGATTAAGGCTGGGATTAGTGGAGTTGGGGTTCCTTCGG

GTAGGAGGTGGCCAAGTGAAGCGGATGGTTGGTTTCGGAGGCCTGTGAGTAGAGTGGCTAGTCATAGTGGGAAGGCTAGT

GCTAGGTTTATAGATAATTGTGTGGTAGGTGTGAATGTGTAGGGGAGTAGGCCTAGTAGGTTGATTGAGAGAAGGAAAAT

TATTAGTGATGACAGGATAAGGGCTCATTTGTGGCCTTTTTTGTTCAATGGGATTATCAGTTGTTTGGTGATTATATAGA

TGAATCACGATTGTAGGGTGGATGTTCGGTTGGTGATTCATCGGTTGTGGGGGGAGGGGAGTAGTAGGGCTGGGAATAAT

AGTGAGATGAGAATTAGGGGGATTCCTAGTAGGTGGGGGCTTATAAATTGGTCGAAGAAGCTTAGGTTCATGGTCAGGGT

CAGGATGAGGGTTTTGTGTTTGTTAGGGGTTTTTCTAGGGGGGAGTTAGTGGGCATGAATGAAAGGAGTTTGGGTTGGAT

GATTAGTGAAAAGGTCAGTCATGAAATAGTTATAATGAATAGTCATGGGTTTGGATTTAGTTGGGGCATATCATTAGGGA

GGGTTGGTAATCCTCTTTCTCTAGCTTAAAAGGCTAGTGCTGTTGCATAGCTTCCTAGTGGTTAGGATGATAGGAAAATT

AACATGATAAACATGATGATGGATGATGATTATTTTTTAATGTAATCTCGGTGTTGAATCTGGGTTAAAGTTAGATGATA

AAATGTGTGATGAAAATATCATGGATAGTATGGAAATTATAACTTAGAACTCCAGTGGTGTTGGTTAGAAAGTTAGAGGA

AGTGAAAAACTTGTAAAATATGTCCGGCAACCATAAATCAAAAAGCAACATAGCTTAGTAAGTATGTGAAAATACCATAA

CCAAATGCAAGACAAAGTGCATCAGTGTCAAAATGAGACTGAATTACACATGAAACCGTCTCATTCAGCAACTCTTGAAG

GAGAGAAAAGGCCCGGGAGCCACTGATGCCCAGGTCAAGAGATTGTGTGCTCCGCGTTAGCCACTGGAAGGGCCTATTGA

GATTACCCAAAAAAAAAGA-GAACCAACC-GCCTAAAAGAAGGGAACCATGCCGCGATTAAGAAAGGTATGAACTATAAA

GTACCAACCAAATGCCTTTGAAAGGAAATCCACAGGGGAGGAATCTA

91_Campylopterus_curvipennis_Bris-5

GGATTATGGCTACTGCTACTTCTAGGATGGTTAGTAGGAGTAGTACGCAGGCGGTTAGAGCAGAGATTGCGGGTATGATG

GAGAATAGGACTGCGGTGGCTGTTGAGATGAGTTGGATTAGTAGGTGGCCTGCTGTTAGGTTAGCTGTGAGGCGGACTCC

TAGGGCTAGAGGGCGGATCAGGAGGCTGGTAGTTTCAATTATGATTAAGGCTGGGATTAGTGGAGTTGGGGTTCCTTCGG

GTAGGAGGTGGCCAAGTGAAGCGGATGGTTGGTTTCGGAGGCCTGTGAGTAGAGTGGCTAGTCATAGTGGGAAGGCTAGT

GCTAGGTTTATAGATAATTGTGTGGTAGGTGTGAATGTGTAGGGGAGTAGGCCTAGTAGGTTGATTGAGAGAAGGAAAAT

TATTAGTGATGACAGGATAAGGGCTCATTTGTGGCCTTTTTTGTTCAATGGGATTATCAGTTGTTTGGTGATTATATAGA

TGAATCACGATTGTAGGGTGGATGTTCGGTTGGTGATTCATCGGTTGTGGGGGGAGGGGAGTAGTAGGGCTGGGAATAAT

AGTGAGATGAGAATTAGGGGGATTCCTAGTAGGTGGGGGCTTATAAATTGGTCGAAGAAGCTTAGGTTCATGGTCAGGGT

CAGGATGAGGGTTTTGTGTTTGTTAGGGGTTTTTCTAGGGGGGAGTTAGTGGGCATGAATGAAAGGAGTTTGGGTTGGAT

GATTAGTGAAAAGGTCAGTCATGAAATAGTTATAATGAATAGTCATGGGTTTGGATTTAGTTGGGGCATATCATTAGGGA

GGGTTGGTAATCCTCTTTCTCTAGCTTAAAAGGCTAGTGCTGTTGCATAGCTTCCTAGTGGTTAGGATGATAGGAAAATT

AACATGATAAACATGATGATGGATGATGATTATTTTTTAATGTAATCTCGGTGTTGAATCTGGGTTAAAGTTAGATGATA

AAATGTGTGATGAAAATATCATGGATAGTATGGAAATTATAACTTAGAACTCCAGTGGTGTTGGTTAGAAAGTTAGAGGA

AGTGAAAAACTTGTAAAATATGTCCGGCAACCATAAATCAAAAAGCAACATAGCTTAGTAAGTATGTGAAAATACCATAA

CCAAATGCAAGACAAAGTGCATCAGTGTCAAAATGAGACTGAATTACACATGAAACCGTCTCATTCAGCAACTCTTGAAG

GAGAGAAAAGGCCCGGGAGCCACTGATGCCCAGGTCAAGAGATTGTGTGCTCCGCGTTAGCCACTGGAAGGGCCTATTGA

GATTACCCAAAAAAAAAGA-GAACCAACC-GCCTAAAAGAAGGGAACCATGCCGCGATTAAGAAAGGTATGAACTATAAA

GTACCAACCAAATGCCTTTGAAAGGAAATCCACAGGGGAGGAATCTA

92_Campylopterus_curvipennis_Bris-6

GGATTATGGCTACTGCTACTTCTAGGATGGTTAGTAGGAGTAGTACGCAGGCGGTTAGAGCAGAGATTGCGGGTATGATG

GAGAATAGGACTGCGGTGGCTGTTGAGATGAGTTGGATTAGTAGGTGGCCTGCTGTTAGGTTAGCTGTGAGGCGGACTCC

TAGGGCTAGAGGGCGGATCAGGAGGCTGGTAGTTTCAATTATGATTAAGGCTGGGATTAGTGGAGTTGGGGTTCCTTCGG

GTAGGAGGTGGCCAAGTGAAGCGGATGGTTGGTTTCGGAGGCCTGTGAGTAGAGTGGCTAGTCATAGTGGGAAGGCTAGT

GCTAGGTTTATAGATAATTGTGTGGTAGGTGTGAATGTGTAGGGGAGTAGGCCTAGTAGGTTGATTGAGAGAAGGAAAAT

TATTAGTGATGACAGGATAAGGGCTCATTTGTGGCCTTTTTTGTTCAATGGGATTATCAGTTGTTTGGTGATTATATAGA

TGAATCACGATTGTAGGGTGGATGTTCGGTTGGTGATTCATCGGTTGTGGGGGGAGGGGAGTAGTAGGGCTGGGAATAAT

AGTGAGATGAGAATTAGGGGGATTCCTAGTAGGTGGGGGCTTATAAATTGGTCGAAGAAGCTTAGGTTCATGGTCAGGGT

CAGGATGAGGGTTTTGTGTTTGTTAGGGGTTTTTCTAGGGGGGAGTTAGTGGGCATGAATGAAAGGAGTTTGGGTTGGAT

GATTAGTGAAAAGGTCAGTCATGAAATAGTTATAATGAATAGTCATGGGTTTGGATTTAGTTGGGGCATATCATTAGGGA

GGGTTGGTAATCCTCTTTCTCTAGCTTAAAAGGCTAGTGCTGTTGCATAGCTTCCTAGTGGTTAAGATGATAGGAAAATT

AACATGATAAACATGATGATGGATGATGATTATTTTTTAATGTAATCTCGGTGTTGAATCTGGGTTAAAGTTAGATGATA

AAATGTGTGATGAAAATATCATGGATAGTATGGAAATTATAACTTAGAACTCCAGTGGTGTTGGTTAGAAAGTTAGAGGA

AGTGAAAAACTTGTAAAATATGTCCGGCAACCATAAATCAAAAAGCAACATAGCTTAGTAAGTATGTGAAAATACCATAA

CCAAATGCAAGACAAAGTGCATCAGTGTCAAAATGAGACTGAATTACACATGAAACCGTCTCATTCAGCAACTCTTGAAG

GAGAGAAAAGGCTCGGGAGCCACTGATGCTCAGGTCAAGAGATTGTGTGCTCCGCGTTAGCCACTGGAAGGGCCTATTGA

GATTACCCAAAAAAAAAGA-GAACCAACC-GCCTAAAAGAAGGGAACCATGCCGCGATTAAGAAAGGTATGAACTATAAA

GTACCAACCAAATGCCTTTGAAAGGAAATCCACAGGGGAGGAGTCTA

93_Campylopterus_curvipennis_Bris-7

GGATTATGGCTACTGCTACTTCTAGGATGGTTAGTAGGAGTAGTACGCAGGCGGTTAGAGCAGAGATTGCGGGTATGATG

GAGAATAGGACTGCGGTGGCTGTTGAGATGAGTTGGATTAGTAGGTGGCCTGCTGTTAGGTTAGCTGTGAGGCGGACTCC

TAGGGCTAGAGGGCGGATCAGGAGGCTGGTAGTTTCAATTATGATTAAGGCTGGGATTAGTGGAGTTGGGGTTCCTTCGG

GTAGGAGGTGGCCAAGTGAAGCGGATGGTTGGTTTCGGAGGCCTGTGAGTAGAGTGGCTAGTCATAGTGGGAAGGCTAGT

GCTAGGTTTATAGATAATTGTGTGGTAGGTGTGAATGTGTAGGGGAGTAGGCCTAGTAGGTTGATTGAGAGAAGGAAAAT

TATTAGTGATGACAGGATAAGGGCTCATTTGTGGCCTTTTTTGTTCAATGGGATTATCAGTTGTTTGGTGATTATATAGA

TGAATCACGATTGTAGGGTGGATGTTCGGTTGGTGATTCATCGGTTGTGGGGGGAGGGGAGTAGTAGGGCTGGGAATAAT

AGTGAGATGAGAATTAGGGGGATTCCTAGTAGGTGGGGGCTTATAAATTGGTCGAAGAAGCTTAGGTTCATGGTCAGGGT

CAGGATGAGGGTTTTGTGTTTGTTAGGGGTTTTTCTAGGGGGGAGTTAGTGGGCATGAATGAAAGGAGTTTGGGTTGGAT

GATTAGTGAAAAGGTCAGTCATGAAATAGTTATAATGAATAGTCATGGGTTTGGATTTAGTTGGGGCATATCATTAGGGA

GGGTTGGTAATCCTCTTTCTCTAGCTTAAAAGGCTAGTGCTGTTGCATAGCTTCCTAGTGGTTAGGATGATAGGAAAATT

AACATGATAAACATGATGATGGATGATGATTATTTTTTAATGTAATCTCGGTGTTGAATCTGGGTTAAAGTTAGATGATA

AAATGTGTGATGAAAATATCATGGATAGTATGGAAATTATAACTTAGAACTCCAGTGGTGTTGGTTAGAAAGTTAGAGGA

AGTGAAAAACTTGTAAAATATGTCCGGCAACCATAAATCAAAAAGCAACATAGCTTAGTAAGTATGTGAAAATACCATAA

CCAAATGCAAGACAAAGTGCATCAGTGTCAAAATGAGACTGAATTACACATGAAACCGTCTCATTCAGCAACTCTTGAAG

GAGAGAAAAGGCTCGGGAGCCACTGATGCCCAGGTCAAGAGATTGTGTGCTCCGCGTTAGCCACTGGAAGGGCCTATTGA

GATTACCCAAAAAAAAAGA-GAACCAACC-GCCTAAAAGAAGGGAACCATGCCGCGATTAAGAAAGGTATGAACTATAAA

GTACCAACCAAATGCCTTTGAAAGGAAATCCACAGGGGAGGAGTCTA

94_Campylopterus_curvipennis_Bris-10

GGATTATGGCTACTGCTACTTCTAGGATGGTTAGTAGGAGTAGTACGCAGGCGGTTAGAGCAGAGATTGCGGGTATGATG

GAGAATAGGACTGCGGTGGCTGTTGAGATGAGTTGGATTAGTAGGTGGCCTGCTGTTAGGTTAGCTGTGAGGCGGACTCC

TAGGGCTAGAGGGCGGATCAGGAGGCTGGTAGTTTCAATTATGATTAAGGCTGGGATTAGTGGAGTTGGGGTTCCTTCGG

GTAGGAGGTGGCCAAGTGAAGCGGATGGTTGGTTTCGGAGGCCTGTGAGTAGAGTGGCTAGTCATAGTGGGAAGGCTAGT

GCTAGGTTTATAGATAATTGTGTGGTAGGTGTGAATGTGTAGGGGAGTAGGCCTAGTAGGTTGATTGAGAGAAGGAAAAT

TATTAGTGATGACAGGATAAGGGCTCATTTGTGGCCTTTTTTGTTCAATGGGATTATCAGTTGTTTGGTGATTATATAGA

TGAATCACGATTGTAGGGTGGATGTTCGGTTGGTGATTCATCGGTTGTGGGGGGAGGGGAGTAGTAGGGCTGGGAATAAT

AGTGAGATGAGAATTAGGGGGATTCCTAGTAGGTGGGGGCTTATAAATTGGTCGAAGAAGCTTAGGTTCATGGTCAGGGT

CAGGATGAGGGTTTTGTGTTTGTTAGGGTTTTTTCTAGGGGGGAGTTAGTGGGCATGAATGAAAGGAGTTTGGGTTGGAT

GATTAGTGAAAAGGTCAGTCATGAAATAGTTATAATGAATAGTCATGGGTTTGGATTTAGTTGGGGCATATCATTAGGGA

GGGTTGGTAATCCTCTTTCTCTAGCTTAAAAGGCTAGTGCTGTTGCATAGCTTCCTAGTGGTTAGGATGATAGGAAAATT

AACATGATAAACATGATGATGGATGATGATTATTTTTTAATGTAATCTCGGTGTTGAATCTGGGTTAAAGTTAGATGATA

AAATGTGTGATGAAAATATCATGGATAGTATGGAAATTATAACTTAGAACTCCAGTGGTGTTGGTTAGAAAGTTAGAGGA

AGTGAAAAACTTGTAAAATATGTCCGGCAACCATAAATCAAAAAGCAACATAGCTTAGTAAGTATGTGAAAATACCATAA

CCAAATGCAAGACAAAGTGCATCAGTGTCAAAATGAGACTGAATTACACATGAAACCGTCTCATTCAGCAACTCTTGAAG

GAGAGAAAAGGCTCGGGAGCCACTGATGCCCAGGTCAAGAGATTGTGTGCTCCGCGTTAGCCACTGGAAGGGCCTATTGA

GATTACCCAAAAAAAAAGA-GAACCAACC-GCCTAAAAGAAGGGAACCATGCCGCGATTAAGAAAGGTATGAACTATAAA

GTACCAACCAAATGCCTTTGAAAGGAAGTCCACAGGGGAGGAGTCTA

95_Campylopterus_curvipennis_Bris-11

GGATTATGGCTACTGCTACTTCTAGGATGGTTAGTAGGAGTAGTACGCAGGCGGTTAGAGCAGAGATTGCGGGTATGATG

GAGAATAGGACTGCGGTGGCTGTTGAGATGAGTTGGATTAGTAGGTGGCCTGCTGTTAGGTTAGCTGTGAGGCGGACTCC

TAGGGCTAGAGGGCGGATCAGGAGGCTGGTAGTTTCAATTATGATTAAGGCTGGGATTAGTGGAGTTGGGGTTCCTTCGG

GTAGGAGGTGGCCAAGTGAAGCGGATGGTTGGTTTCGGAGGCCTGTGAGTAGAGTGGCTAGTCATAGTGGGAAGGCTAGT

GCAAGGTTTATAGATAATTGTGTGGTAGGTGTGAATGTGTAGGGGAGTAGGCCTAGTAGGTTGATTGAGAGAAGGAAAAT

TATTAGTGATGACAGGATAAGGGCTCATTTGTGGCCTTTTTTGTTCAATGGGATTATCAGTTGTTTGGTGATTATATAGA

TGAATCACGATTGTAGGGTGGATGTTCGGTTGGTGATTCATCGGTTGTGGGGGGAGGGGAGTAGTAGGGCTGGGAATAAT

AGTGAGATGAGAATTAGGGGGATTCCTAGTAGGTGGGGGCTTATAAATTGGTCGAAGAAGCTTAGGTTCATGGTCAGGGT

CAGGATGAGGGTTTTGTGTTTGTTAGGGGTTTTTCTAGGGGGGAGTTAGTGGGCATGAATGAAAGGAGTTTGGGTTGGAT

GATTAGTGAAAAGGTCAGTCATGAAATAGTTATAATGAATAGTCATGGGTTTGGATTTAGTTGGGGCATATCATTAGGGA

GGGTTGGTAATCCTCTTTCTCTAGCTTAAAAGGCTAGTGCTGTTGCATAGCTTCCTAGTGGTTAGGATGATAGGAAAATT

AACATGATAAACATGATGATGGATGATGATTATTTTTTAATGTAATCTCGGTGTTGAATCTGGGTTAAAGTTAGATGATA

AAATGTGTGATGAAAATATCATGGATAGTATGGAAATTATAACTTAGAACTCCAGTGGTGTTGGTTAGAAAGTTAGAGGA

AGTGAAAAACTTGTAAAATATGTCCGGCAACCATAAATCAAAAAGCAACATAGCTTAGTAAGTATGTGAAAATACCATAA

CCAAATGCAAGACAAAGTGCATCAGTGTCAAAATGAGACTGAATTACACATGAAACCGTCTCATTCAGCAACTCCTGAAG

GAGAGAAAAGGCCCGGGAGCCACTGATGCCCAGGTCAAGAGATTGTGTGCTCCGCGTTAGCCACTGGAAGGGCCTATTGA

GATTACCCAAAAAAAAAGA-GAACCAACC-GCCTAAAAGAAGGGAACCATGCCGCGATTAAGAAAGGTATGAACTATAAA

GTACCAACCAAATGCCTTTGAAAGGAAATCCACAGGGGAGGAATCTA

96_Campylopterus_curvipennis_Bris-12

GGATTATGGCTACTGCTACTTCTAGGATGGTTAGTAGGAGTAGTACGCAGGCGGTTAGAGCAGAGATTGCGGGTATGATG

GAGAATAGGACTGCGGTGGCTGTTGAGATGAGTTGGATTAGTAGGTGGCCTGCTGTTAGGTTAGCTGTGAGGCGGACTCC

TAGGGCTAGAGGGCGGATCAGGAGGCTGGTAGTTTCAATTATGATTAAGGCTGGGATTAGTGGAGTTGGGGTTCCTTCGG

GTAGGAGGTGGCCAAGTGAAGCGGATGGTTGGTTTCGGAGGCCTGTGAGTAGAGTGGCTAGTCATAGTGGGAAGGCTAGT

GCTAGGTTTATAGATAATTGTGTGGTAGGTGTGAATGTGTAGGGGAGTAGGCCTAGTAGGTTGATTGAGAGAAGGAAAAT

TATTAGTGATGACAGGATAAGGGCTCATTTGTGGCCTTTTTTGTTCAATGGGATTATCAGTTGTTTGGTGATTATATAGA

TGAATCACGATTGTAGGGTGGATGTTCGGTTGGTGATTCATCGGTTGTGGGGGGAGGGGAGTAGTAGGGCTGGGAATAAT

AGTGAGATGAGAATTAGGGGGATTCCTAGTAGGTGGGGGCTTATAAATTGGTCGAAGAAGCTTAGGTTCATGGTCAGGGT

CAGGATGAGGGTTTTGTGTTTGTTAGGGTTTTTTCTAGGGGGGAGTTAGTGGGCATGAATGAAAGGAGTTTGGGTTGGAT

GATTAGTGAAAAGGTCAGTCATGAAATAGTTATAATGAATAGTCATGGGTTTGGATTTAGTTGGGGCATATCATTAGGGA

GGGTTGGTAATCCTCTTTCTCTAGCTTAAAAGGCTAGTGCTGTTGCATAGCTTCCTAGTGGTTAAGATGATAGGAAAATT

AACATGATAAACATGATGATGGATGATGATTATTTTTTAATGTAATCTCGGTGTTGAATCTGGGTTAAAGTTAGATGATA

AAATGTGTGATGAAAATATCATGGATAGTATGGAAATTATAACTTAGAACTCCAGTGGTGTTGGTTAGAAAGTTAGAGGA

AGTGAAAAACTTGTAAAATATGTCCGGCAACCATAAATCAAAAAGCAACATAGCTTAGTAAGTATGTGAAGATACCATAA

CCAAATGCAAGACAAAGTGCATCAGTGTCAAAATGAGACTGAATTACACATGAAACCGTCTCATTCAGCAACTCTTGAAG

GAGAGAAAAGGCTCGGGAGCCACTGATGCCCAGGTCAAGAGATTGTGTGCTCCGCGTTAGCCACTGGAAGGGCCTATTGA

GATTACCCAAAAAAAAAGA-GAACCAACC-GCCTAAAAGAAGGGAACCATGCCGCGATTAAGAAAGGTATGAACTATAAA

GTACCAACCAAATGCCTTTGAAAGGAAATCTACAGGGGAGGAGTCTA

97_Campylopterus_curvipennis_Bris-13

GGATTATGGCTACTGCTACTTCTAGGATGGTTAGTAGGAGTAGTACGCAGGCGGTTAGAGCAGAGATTGCGGGTATGATG

GAGAATAGGACTGCGGTGGCTGTTGAGATGAGTTGGATTAGTAGGTGGCCTGCTGTTAGGTTAGCTGTGAGGCGGACTCC

TAGGGCTAGAGGGCGGATCAGGAGGCTGGTAGTTTCAATTATGATTAAGGCTGGGATTAGTGGAGTTGGGGTTCCTTCGG

GTAGGAGGTGGCCAAGTGAAGCGGATGGTTGGTTTCGGAGGCCTGTGAGTAGAGTGGCTAGTCATAGTGGGAAGGCTAGT

GCTAGGTTTATAGATAATTGTGTGGTAGGTGTGAATGTGTAGGGGAGTAGGCCTAGTAGGTTGATTGAGAGAAGGAAAAT

TATTAGTGATGACAGGATAAGGGCTCATTTGTGGCCTTTTTTGTTCAATGGGATTATCAGTTGTTTGGTGATTATATAGA

TGAATCACGATTGTAGGGTGGATGTTCGGTTGGTGATTCATCGGTTGTGGGGGGAGGGGAGTAGTAGGGCTGGGAATAAT

AGTGAGATGAGAATTAGGGGGATTCCTAGTAGGTGGGGGCTTATAAATTGGTCGAAGAAGCTTAGGTTCATGGTCAGGGT

CAGGATGAGGGTTTTGTGTTTGTTAGGGTTTTTTCTAGGGGGGAGTTAGTGGGCATGAATGAAAGGAGTTTGGGTTGGAT

GATTAGTGAAAAGGTCAGTCATGAAATAGTTATAATGAATAGTCATGGGTTTGGATTTAGTTGGGGCATATCATTAGGGA

GGGTTGGTAATCCTCTTTCTCTAGCTTAAAAGGCTAGTGCTGTTGCATAGCTTCCTAGTGGTTAAGATGATAGGAAAATT

AACATGATAAACATGATGATGGATGATGATTATTTTTTAATGTAATCTCGGTGTTGAATCTGGGTTAAAGTTAGATGATA

AAATGTGTGATGAAAATATCATGGATAGTATGGAAATTATAACTTAGAACTCCAGTGGTGTTGGTTAGAAAGTTAGAGGA

AGTGAAAAACTTGTAAAATATGTCCGGCAACCATAAATCAAAAAGCAACATAGCTTAGTAAGTATGTGAAAATACCATAA

CCAAATGCAAGACAAAGTGCATCAGTGTCAAAATGAGACTGAATTACACATGAAACCGTCTCATTCAGCAACTCTTGAAG

GAGAGAAAAGGCTCGGGAGCCACTGATGCCCAGGTCAAGAGATTGTGTGCTCCGCGTTAGCCACTGGAAGGGCCTATTGA

GATTACCCAAAAAAAAAGA-GAACCAACC-GCCTAAAAGAAGGGAACCATGCCGCGATTAAGAAAGGTATGAACTATAAA

GTACCAACCAAATGCCTTTGAAAGGAAATCCACAGGGGAGGAGTCTA

98_Campylopterus_curvipennis_Bris-14

GGATTATGGCTACTGCTACTTCTAGGATGGTTAGTAGGAGTAGTACGCAGGCGGTTAGAGCAGAGATTGCGGGTATGATG

GAGAATAGGACTGCGGTGGCTGTTGAGATGAGTTGGATTAGTAGGTGGCCTGCTGTTAGGTTAGCTGTGAGGCGGACTCC

TAGGGCTAGAGGGCGGATCAGGAGGCTGGTAGTTTCAATTATGATTAAGGCTGGGATTAGTGGAGTTGGGGTTCCTTCGG

GTAGGAGGTGGCCAAGTGAAGCGGATGGTTGGTTTCGGAGGCCTGTGAGTAGAGTGGCTAGTCATAGTGGGAAGGCTAGT

GCAAGGTTTATAGATAATTGTGTGGTAGGTGTGAATGTGTAGGGGAGTAGGCCTAGTAGGTTGATTGAGAGAAGGAAAAT

TATTAGTGATGACAGGATAAGGGCTCATTTGTGGCCTTTTTTGTTCAATGGGATTATCAGTTGTTTGGTGATTATATAGA

TGAATCACGATTGTAGGGTGGATGTTCGGTTGGTGATTCATCGGTTGTGGGGGGAGGGGAGTAGTAGGGCTGGGAATAAT

AGTGAGATGAGAATTAGGGGGATTCCTAGTAGGTGGGGGCTTATAAATTGGTCGAAGAAGCTTAGGTTCATGGTCAGGGT

CAGGATGAGGGTTTTGTGTTTGTTAGGGGTTTTTCTAGGGGGGAGTTAGTGGGCATGAATGAAAGGAGTTTGGGTTGGAT

GATTAGTGAAAAGGTCAGTCATGAAATAGTTATAATGAATAGTCATGGGTTTGGATTTAGTTGGGGCATATCATTAGGGA

GGGTTGGTAATCCTCTTTCTCTAGCTTAAAAGGCTAGTGCTGTTGCATAGCTTCCTAGTGGTTAGGATGATAGGAAAATT

AACATGATAAACATGATGATGGATGATGATTATTTTTTAATGTAATCTCGGTGTTGAATCTGGGTTAAAGTTAGATGATA

AAATGTGTGATGAAAATATCATGGATAGTATGGAAATTATAACTTAGAACTCCAGTGGTGTTGGTTAGAAAGTTAGAGGA

AGTGAAAAACTTGTAAAATATGTCCGGCAACCATAAATCAAAAAGCAACATAGCTTAGTAAGTATGTGAAAATACCATAA

CCAAATGCAAGACAAAGTGCATCAGTGTCAAAATGAGACTGAATTACACATGAAACCGTCTCATTCAGCAACTCTTGAAG

GAGAGAAAAGGCCCGGGAGCCACTGATGCCCAGGTCAAGAGATTGTGTGCTCCGCGTTAGCCACTGGAAGGGCCTATTGA

GATTACCCAAAAAAAAAGA-GAACCAACC-GCCTAAAAGAAGGGAACCATGCCGCGATTAAGAAAGGTATGAACTATAAA

GTACCAACCAAATGCCTTTGAAAGGAAATCCACAGGGGAGGAATCTA

99_Campylopterus_curvipennis_Bris-15

GGATTATGGCTACTGCTACTTCTAGGATGGTTAGTAGGAGTAGTACGCAGGCGGTTAGAGCAGAGATTGCGGGTATGATG

GAGAATAGGACTGCGGTGGCTGTTGAGATGAGTTGGATTAGTAGGTGGCCTGCTGTTAGGTTAGCTGTGAGGCGGACTCC

TAGGGCTAGAGGGCGGATCAGGAGGCTGGTAGTTTCAATTATGATTAAGGCTGGGATTAGTGGAGTTGGGGTTCCTTCGG

GTAGGAGGTGGCCAAGTGAAGCGGATGGTTGGTTTCGGAGGCCTGTGAGTAGAGTGGCTAGTCATAGTGGGAAGGCTAGT

GCTAGGTTTATAGATAATTGTGTGGTAGGTGTGAATGTGTAGGGGAGTAGGCCTAGTAGGTTGATTGAGAGAAGGAAAAT

TATTAGTGATGACAGGATAAGGGCTCATTTGTGGCCTTTTTTGTTCAATGGGATTATCAGTTGTTTGGTGATTATATAGA

TGAATCACGATTGTAGGGTGGATGTTCGGTTGGTGATTCATCGGTTGTGGGGGGAGGGGAGTAGTAGGGCTGGGAATAAT

AGTGAGATGAGAATTAGGGGGATTCCTAGTAGGTGGGGGCTTATAAATTGGTCGAAGAAGCTTAGGTTCATGGTCAGGGT

CAGGATGAGGGTTTTGTGTTTGTTAGGGGTTTTTCTAGGGGGGAGTTAGTGGGCATGAATGAAAGGAGTTTGGGTTGGAT

GATTAGTGAAAAGGTCAGTCATGAAATAGTTATAATGAATAGTCATGGGTTTGGATTTAGTTGGGGCATATCATTAGGGA

GGGTTGGTAATCCTCTTTCTCTAGCTTAAAAGGCTAGTGCTGTTGCATAGCTTCCTAGTGGTTAAGATGATAGGAAAATT

AACATGATAAACATGATGATGGATGATGATTATTTTTTAATGTAATCTCGGTGTTGAATCTGGGTTAAAGTTAGATGATA

AAATGTGTGATGAAAATATCATGGATAGTATGGAAATTATAACTTAGAACTCCAGTGGTGTTGGTTAGAAAGTTAGAGGA

AGTGAAAAACTTGTAAAATATGTCCGGCAACCATAAATCAAAAAGCAACATAGCTTAGTAAGTATGTGAAAATACCATAA

CCAAATGCAAGACAAAGTGCATCAGTGTCAAAATGAGACTGAATTACACATGAAACCGTCTCATTCAGCAACTCTTGAAG

GAGAGAAAAGGCTCGGGAGCCACTGATGCCCAGGTCAAGAGATTGTGTGCTCCGCGTTAGCCACTGGAAGGGCCTATTGA

GATTACCCAAAAAAAAAGA-GAACCAACC-GCCTAAAAGAAGGGAACCATGCCGCGATTAAGAAAGGTATGAACTATAAA

GTACCAACCAAATGCCTTTGAAAGGAAATTCACAGGGGAGGAGTCTA

100_Campylopterus_curvipennis_Bris-16

GGATTATGGCTACTGCTACTTCTAGGATGGTTAGTAGGAGTAGTACGCAGGCGGTTAGAGCAGAGATTGCGGGTATGATG

GAGAATAGGACTGCGGTGGCTGTTGAGATGAGTTGGATTAGTAGGTGGCCTGCTGTTAGGTTAGCTGTGAGGCGGACTCC

TAGGGCTAGAGGGCGGATCAGGAGGCTGGTAGTTTCAATTATGATTAAGGCTGGGATTAGTGGAGTTGGGGTTCCTTCGG

GTAGGAGGTGGCCAAGTGAAGCGGATGGTTGGTTTCGGAGGCCTGTGAGTAGAGTGGCTAGTCATAGTGGGAAGGCTAGT

GCTAGGTTTATAGATAATTGTGTGGTAGGTGTGAATGTGTAGGGGAGTAGGCCTAGTAGGTTGATTGAGAGAAGGAAAAT

TATTAGTGATGACAGGATAAGGGCTCATTTGTGGCCTTTTTTGTTCAATGGGATTATCAGTTGTTTGGTGATTATATAGA

TGAATCACGATTGTAGGGTGGATGTTCGGTTGGTGATTCATCGGTTGTGGGGGGAGGGGAGTAGTAGGGCTGGGAATAAT

AGTGAGATGAGAATTAGGGGGATTCCTAGTAGGTGGGGGCTTATAAATTGGTCGAAGAAGCTTAGGTTCATGGTCAGGGT

CAGGATGAGGGTTTTGTGTTTGTTAGGGGTTTTTCTAGGGGGGAGTTAGTGGGCATGAATGAAAGGAGTTTGGGTTGGAT

GATTAGTGAAAAGGTCAGTCATGAAATAGTTATAATGAATAGTCATGGGTTTGGATTTAGTTGGGGCATATCATTAGGGA

GGGTTGGTAATCCTCTTTCTCTAGCTTAAAAGGCTAGTGCTGTTGCATAGCTTCCTAGTGGTTAAGATGATAGGAAAATT

AACATGATAAACATGATGATGGATGATGATTATTTTTTAATGTAATCTCGGTGTTGAATCTGAGTTAAAGTTAGATGATA

AAATGTGTGATGAAAATATCATGGATAGTATGGAAATTATAACTTAGAACTCCAGTGGTGTTGGTTAGAAAGTTAGAGGA

AGTGAAAAACTTGTAAAATATGTCCGGCAACCATAAATCAAAAAGCAACATAGCTTAGTAAGTATGTGAAAATACCATAA

CCAAATGCAAGACAAAGTGCATCAGTGTCAAAATGAGACTGAATTACACATGAAACCGTCTCATTCAGCAACTCTTGAAG

GAGAGAAAAGGCTCGGGAGCCACTGATGCTCAGGTCAAGAGATTGTGTGCTCCGCGTTAGCCACTGGAAGGGCCTATTGA

GATTACCCAAAAAAAAAGA-GAACCAACC-GCCTAAAAGAAGGGAACCATGCCGCGATTAAGAAAGGTATGAACTATAAA

GTACCAACCAAATGCCTTTGAAAGGAAATCCACAGGGGAGGAGTCTA

101_Campylopterus_curvipennis_Bris-17

GGATTATGGCTACTGCTACTTCTAGGATGGTTAGTAGGAGTAGTACGCAGGCGGTTAGAGCAGAGATTGCGGGTATGATG

GAGAATAGGACTGCGGTGGCTGTTGAGATGAGTTGGATTAGTAGGTGGCCTGCTGTTAGGTTAGCTGTGAGGCGGACTCC

TAGGGCTAGAGGGCGGATCAGGAGGCTGGTAGTTTCAATTATGATTAAGGCTGGGATTAGTGGAGTTGGGGTTCCTTCGG

GTAGGAGGTGGCCAAGTGAAGCGGATGGTTGGTTTCGGAGGCCTGTGAGTAGAGTGGCTAGTCATAGCGGGAAGGCTAGT

GCTAGGTTTATAGATAATTGTGTGGTAGGTGTGAATGTGTAGGGGAGTAGGCCTAGTAGGTTGATTGAGAGAAGGAAAAT

TATTAGTGATGACAGGATAAGGGCTCATTTGTGGCCTTTTTTGTTCAATGGGATTATCAGTTGTTTGGTGATTATATAGA

TGAATCACGATTGTAGGGTGGATGTTCGGTTGGTGATTCATCGGTTGTGGGGGGAGGGGAGTAGTAGGGCTGGGAATAAT

AGTGAGATGAGAATTAGGGGGATTCCTAGTAGGTGGGGGCTTATAAATTGGTCGAAGAAGCTTAGGTTCATGGTCAGGGT

CAGGATGAGGGTTTTGTGTTTGTTAGGGTTTTTTCTAGGGGGGAGTTAGTGGGCATGAATGAAAGGAGTTTGGGTTGGAT

GATTAGTGAAAAGGTCAGTCATGAAATAGTTATAATGAATAGTCATGGGTTTGGATTTAGTTGGGGCATATCATTAGGGA

GGGTTGGTAATCCTCTTTCTCTAGCTTAAAAGGCTAGTGCTGTTGCATAGCTTCCTAGTGGTTAAGATGATAGGAAAATT

AACATGATAAACATGATGATGGATGATGATTATTTTTTAATGTAATCTCGGTGTTGAATCTGGGTTAAAGTTAGATGATA

AAATGTGTGATGAAAATATCATGGATAGTATGGAAATTATAACTTAGAACTCCAGTGGTGTTGGTTAGAAAGTTAGAGGA

AGTGAAAAACTTGTAAAATATGTCCGGCAACCATAAATCAAAAAGCAACATAGCTTAGTAAGTATGTGAAAATACCATAA

CCAAATGCAAGACAAAGTGCATCAGTGTCAAAATGAGACTGAATTACACATGAAACCGTCTCATTCAGCAACTCTTGAAG

GAGAGAAAAGGCTCGGGAGCCACTGATGCCCAGGTCAAGAGATTGTGTGCTCCGCGTTAGCCACTGGAAGGGCCTATTGA

GATTACCCAAAAAAAAAGA-GAACCAACC-GCCTAAAAGAAGGGAACCATGCCGCGATTAAGAAAGGTATGAACTATAAA

GTACCAACCAAATGCCTTTGAAAGGAAATCCACAGGGGAGGAGTCTA

102_Campylopterus_curvipennis_Bris-18

GGATTATGGCTACTGCTACTTCTAGGATGGTTAGTAGGAGTAGTACGCAGGCGGTTAGAGCAGAGATTGCGGGTATGATG

GAGAATAGGACTGCGGTGGCTGTTGAGATGAGTTGGATTAGTAGGTGGCCTGCTGTTAGGTTAGCTGTGAGGCGGACTCC

TAGGGCTAGAGGGCGGATCAGGAGGCTGGTAGTTTCAATTATGATTAAGGCTGGGATTAGTGGAGTTGGGGTTCCTTCGG

GTAGGAGGTGGCCAAGTGAAGCGGATGGTTGGTTTCGGAGGCCTGTGAGTAGAGTGGCTAGTCATAGTGGGAAGGCTAGT

GCTAGGTTTATAGATAATTGTGTGGTAGGTGTGAATGTGTAGGGGAGTAGGCCTAGTAGGTTGATTGAGAGAAGGAAAAT

TATTAGTGATGATAGGATAAGGGCTCATTTGTGGCCTTTTTTGTTTAATGGGATTATCAGTTGTTTGGTGATTATATAGA

TGAATCACGATTGTAGGGTGGATGTTCGGTTGGTGATTCATCGGTTGTGGGGGGAGGGGAGTAGTAGGGCTGGGAATAAT

AGTGAGATGAGAATTAGGGGGATTCCTAGTAGGTGGGGGCTTATAAATTGGTCGAAGAAGCTTAGGTTCATGGTCAGGGT

CAGGATGAGGGTTTTGTGTTTGTTAGGGGTTTTTCTAGGGGGGAGTTAGTGGGCATGAATGAAAGGAGTTTGGGTTGGAT

GATTAGTGAAAAGGTCAGTCATGAAATAGTTATAATGAATAGTCATGGGTTTGGATTTAGTTGGGGCATATCATTAGGGA

GGGTTGGTAATCCTCTTTCTCTAGCTTAAAAGGCTAGTGCTGTTGCATAGCTTCCTAGTGGTTAAGATGATAGGAAAATT

AACATGATAAACATGATGATGGATGATGATTATTTTTTAATGTAATCTCGGTGTTGAATCTGGGTTAAAGTTAGATGATA

AAATGTGTGATGAAAATATCATGGATAGTATGGAAATTATAACTTAGAACTCCAGTGGTGTTGGTTAGAAAGTTAGAGGA

AGTGAAAAACTTGTAAAATATGTCCGGCAACCATAAATCAAAAAGCAACATAGCTTAGTAAGTATGTGAAAATACCATAA

CCAAATGCAAGACAAAGTGCATCAGTGTCAAAATGAGACTGAATTACACATGAAACCGTCTCATTCAGCAACTCCTGAAG

GAGAGAAAAGGCTCGGGAGCCACTGATGCTCAGGTCAAGAGATTGTGTGCTCCGCGTTAGCCACTGGAAGGGCCTATTGA

GATTACCCAAAAAAAAAGA-GAACCAACC-GCCTAAAAGAAGGGAACCATGCCGCGATTAAGAAAGGTATGAACTATAAA

GTACCAACCAAATGCCTTTGAAAGGAAATCCACAGGGGAGGAGTCTA

103_Campylopterus_curvipennis_Bris-19

GGATTATGGCTACTGCTACTTCTAGGATGGTTAGTAGGAGTAGTACGCAGGCGGTTAGAGCAGAGATTGCGGGTATGATG

GAGAATAGGACTGCGGTGGCTGTTGAGATGAGTTGGATTAGTAGGTGGCCTGCTGTTAGGTTAGCTGTGAGGCGGACTCC

TAGGGCTAGAGGGCGGATCAGGAGGCTGGTAGTTTCAATTATGATTAAGGCTGGGATTAGTGGAGTTGGGGTTCCTTCGG

GTAGGAGGTGGCCAAGTGAAGCGGATGGTTGGTTTCGGAGGCCTGTGAGTAGAGTGGCTAGTCATAGTGGGAAGGCTAGT

GCTAGGTTTATAGATAATTGTGTGGTAGGTGTGAATGTGTAGGGGAGTAGGCCTAGTAGGTTGATTGAGAGAAGGAAAAT

TATTAGTGATGACAGGATAAGGGCTCATTTGTGGCCTTTTTTGTTCAATGGGATTATCAGTTGTTTGGTGATTATATAGA

TGAATCACGATTGTAGGGTGGATGTTCGGTTGGTGATTCATCGGTTGTGGGGGGAGGGGAGTAGTAGGGCTGGGAATAAT

AGTGAGATGAGAATTAGGGGGATTCCTAGTAGGTGGGGGCTTATAAATTGGTCGAAGAAGCTTAGGTTCATGGTCAGGGT

CAGGATGAGGGTTTTGTGTTTGTTAGGGTTTTTTCTAGGGGGGAGTTAGTGGGCATGAATGAAAGGAGTTTGGGTTGGAT

GATTAGTGAAAAGGTCAGTCATGAAATAGTTATAATGAATAGTCATGGGTTTGGATTTAGTTGGGGCATATCATTAGGGA

GGGTTGGTAATCCTCTTTCTCTAGCTTAAAAGGCTAGTGCTGTTGCATAGCTTCCTAGTGGTTAAGATGATAGGAAAATT

AACATGATAAACATGATGATGGATGATGATTATTTTTTAATGTAATCTCGGTGTTGAATCTGGGTTAAAGTTAGATGATA

AAATGTGTGATGAAAATATCATGGATAGTATGGAAATTATAACTTAGAACTCCAGTGGTGTTGGTTAGAAAGTTAGAGGA

AGTGAAAAACTTGTAAAATATGTCCGGCAACCATAAATCAAAAAGCAACATAGCTTAGTAAGTATGTGAAAATACCATAA

CCAAATGCAAGACAAAGTGCATCAGTGTCAAAATGAGACTGAATTACACATGAAACCGTCTCATTCAGCAACTCTTGAAG

GAGAGAAAAGGCTCGGGAGCCACTGATGCCCAGGTCAAGAGATTGTGTGCTCCGCGTTAGCCACTGGAAGGGCCTATTGA

GATTACCCAAAAAAAAAGA-GAACCAACC-GCCTAAAAGAAGGGAACCATGCCGCGATTAAGAAAGGTATGAACTATAAA

GTACCAACCAAATGCCTTTGAAAGGAAATCCACAGGGGAGGAGTCTA

104_Campylopterus_curvipennis_Bris-20

GGATTATGGCTACTGCTACTTCTAGGATGGTTAGTAGGAGTAGTACGCAGGCGGTTAGAGCAGAGATTGCGGGTATGATG

GAGAATAGGACTGCGGTGGCTGTTGAGATGAGTTGGATTAGTAGGTGGCCTGCTGTTAGGTTAGCTGTGAGGCGGACTCC

TAGGGCTAGAGGGCGGATCAGGAGGCTGGTAGTTTCAATTATGATTAAGGCTGGGATTAGTGGAGTTGGGGTTCCTTCGG

GTAGGAGGTGGCCAAGTGAAGCGGATGGTTGGTTTCGGAGGCCTGTGAGTAGAGTGGCTAGTCATAGTGGGAAGGCTAGT

GCTAGGTTTATAGATAATTGTGTGGTAGGTGTGAATGTGTAGGGGAGTAGGCCTAGTAGGTTGATTGAGAGAAGGAAAAT

TATTAGTGATGACAGGATAAGGGCTCATTTGTGGCCTTTTTTGTTCAATGGGATTATCAGTTGTTTGGTGATTATATAGA

TGAATCACGATTGTAGGGTGGATGTTCGGTTGGTGATTCATCGGTTGTGGGGGGAGGGGAGTAGTAGGGCTGGGAATAAT

AGTGAGATGAGAATTAGGGGGATTCCTAGTAGGTGGGGGCTTATAAATTGGTCGAAGAAGCTTAGGTTCATGGTCAGGGT

CAGGATGAGGGTTTTGTGTTTGTTAGGGTTTTTTCTAGGGGGGAGTTAGTGGGCATGAATGAAAGGAGTTTGGGTTGGAT

GATTAGTGAAAAGGTCAGTCATGAAATAGTTATAATGAATAGTCATGGGTTTGGATTTAGTTGGGGCATATCATTAGGGA

GGGTTGGTAATCCTCTTTCTCTAGCTTAAAAGGCTAGTGCTGTTGCATAGCTTCCTAGTGGTTAAGATGATAGGAAAATT

AACATGATAAACATGATGATGGATGATGATTATTTTTTAATGTAATCTCGGTGTTGAATCTGGGTTAAAGTTAGATGATA

AAATGTGTGATGAAAATATCATGGATAGTATGGAAATTATAACTTAGAACTCCAGTGGTGTTGGTTAGAAAGTTAGAGGA

AGTGAAAAACTTGTAAAATATGTCCGGCAACCATAAATCAAAAAGCAACATAGCTTAGTAAGTATGTGAAAATACCATAA

CCAAATGCAAGACAAAGTGCATCAGTGTCAAAATGAGACTGAATTACACATGAAACCGTCTCATTCAGCAACTCTTGAAG

GAGAGAAAAGGCTCGGGAGCCACTGATGCCCAGGTCAAGAGATTGTGTGCTCCGCGTTAGCCACTGGAAGGGCCTATTGA

GATTACCCAAAAAAAAAGA-GAACCAACC-GCCTAAAAGAAGGGAACCATGCCGCGATTAAGAAAGGTATGAACTATAAA

GTACCAACCAAATGCCTTTGAAAGGAAATCCACAGGGGAGGAGTCTA

105_Campylopterus_curvipennis_Bris-22

GGATTATGGCTACTGCTACTTCTAGGATGGTTAGTAGGAGTAGTACGCAGGCGGTTAGAGCAGAGATTGCGGGTATGATG

GAGAATAGGACTGCGGTGGCTGTTGAGATGAGTTGGATTAGTAGGTGGCCTGCTGTTAGGTTAGCTGTGAGGCGGACTCC

TAGGGCTAGAGGGCGGATCAGGAGGCTGGTAGTTTCAATTATGATTAAGGCTGGGATTAGTGGAGTTGGGGTTCCTTCGG

GTAGGAGGTGGCCAAGTGAAGCGGATGGTTGGTTTCGGAGGCCTGTGAGTAGAGTGGCTAGTCATAGTGGGAAGGCTAGT

GCTAGGTTTATAGATAATTGTGTGGTAGGTGTGAATGTGTAGGGGAGTAGGCCTAGTAGGTTGATTGAGAGAAGGAAAAT

TATTAGTGATGACAGGATAAGGGCTCATTTGTGGCCTTTTTTGTTCAATGGGATTATCAGTTGTTTGGTGATTATATAGA

TGAATCACGATTGTAGGGTGGATGTTCGGTTGGTGATTCATCGGTTGTGGGGGGAGGGGAGTAGTAGGGCTGGGAATAAT

AGTGAGATGAGAATTAGGGGGATTCCTAGTAGGTGGGGGCTTATAAATTGGTCGAAGAAGCTTAGGTTCATGGTCAGGGT

CAGGATGAGGGTTTTGTGTTTGTTAGGGTTTTTTCTAGGGGGGAGTTAGTGGGCATGAATGAAAGGAGTTTGGGTTGGAT

GATTAGTGAAAAGGTCAGTCATGAAATAGTTATAATGAATAGTCATGGGTTTGGATTTAGTTGGGGCATATCATTAGGGA

GGGTTGGTAATCCTCTTTCTCTAGCTTAAAAGGCTAGTGCTGTTGCATAGCTTCCTAGTGGTTAAGATGATAGGAAAATT

AACATGATAAACATGATGATGGATGATGATTATTTTTTAATGTAATCTCGGTGTTGAATCTGGGTTAAAGTTAGATGATA

AAATGTGTGATGAAAATATCATGGATAGTATGGAAATTATAACTTAGAACTCCAGTGGTGTTGGTTAGAAAGTTAGAGGA

AGTGAAAAACTTGTAAAATATGTCCGGCAACCATAAATCAAAAAGCAACATAGCTTAGTAAGTATGTGAAAATACCATAA

CCAAATGCAAGACAAAGTGCATCAGTGTCAAAATGAGACTGAATTACACATGAAACCGTCTCATTCAGCAACTCTTGAAG

GAGAGAAAAGGCTCGGGAGCCACTGATGCCCAGGTCAAGAGATTGTGTGCTCCGCGTTAGCCACTGGAAGGGCCTATTGA

GATTACCCAAAAAAAAAGA-GAACCAACC-GCCTAAAAGAAGGGAACCATGCCGCGATTAAGAAAGGTATGAACTATAAA

GTACCAACCAAATGCCTTTGAAAGGAAATCCACAGGGGAGGAGTCTA

106_Campylopterus_curvipennis_Bris-23

GGATTATGGCTACTGCTACTTCTAGGATGGTTAGTAGGAGTAGTACGCAGGCGGTTAGAGCAGAGATTGCGGGTATGATG

GAGAATAGGACTGCGGTGGCTGTTGAGATGAGTTGGATTAGTAGGTGGCCTGCTGTTAGGTTAGCTGTGAGGCGGACTCC

TAGGGCTAGAGGGCGGATCAGGAGGCTGGTAGTTTCAATTATGATTAAGGCTGGGATTAGTGGAGTTGGGGTTCCTTCGG

GTAGGAGGTGGCCAAGTGAAGCGGATGGTTGGTTTCGGAGGCCTGTGAGTAGAGTGGCTAGTCATAGCGGGAAGGCTAGT

GCTAGGTTTATAGATAATTGTGTGGTAGGTGTGAATGTGTAGGGGAGTAGGCCTAGTAGGTTGATTGAGAGAAGGAAAAT

TATTAGTGATGACAGGATAAGGGCTCATTTGTGGCCTTTTTTGTTCAATGGGATTATCAGTTGTTTGGTGATTATATAGA

TGAATCACGATTGTAGGGTGGATGTTCGGTTGGTGATTCATCGGTTGTGGGGGGAGGGGAGTAGTAGGGCTGGGAATAAT

AGTGAGATGAGAATTAGGGGGATTCCTAGTAGGTGGGGGCTTATAAATTGGTCGAAGAAGCTTAGGTTCATGGTCAGGGT

CAGGATGAGGGTTTTGTGTTTGTTAGGGTTTTTTCTAGGGGGGAGTTAGTGGGCATGAATGAAAGGAGTTTGGGTTGGAT

GATTAGTGAAAAGGTCAGTCATGAAATAGTTATAATGAATAGTCATGGGTTTGGATTTAGTTGGGGCATATCATTAGGGA

GGGTTGGTAATCCTCTTTCTCTAGCTTAAAAGGCTAGTGCTGTTGCATAGCTTCCTAGTGGTTAAGATGATAGGAAAATT

AACATGATAAACATGATGATGGATGATGATTATTTTTTAATGTAATCTCGGTGTTGAATCTGGGTTAAAGTTAGATGATA

AAATGTGTGATGAAAATATCATGGATAGTATGGAAATTATAACTTAGAACTCCAGTGGTGTTGGTTAGAAAGTTAGAGGA

AGTGAAAAACTTGTAAAATATGTCCGGCAACCATAAATCAAAAAGCAACATAGCTTAGTAAGTATGTGAAAATACCATAA

CCAAATGCAAGACAAAGTGCATCAGTGTCAAAATGAGACTGAATTACACATGAAACCGTCTCATTCAGCAACTCTTGAAG

GAGAGAAAAGGCTCGGGAGCCACTGATGCCCAGGTCAAGAGATTGTGTGCTCCGCGTTAGCCACTGGAAGGGCCTATTGA

GATTACCCAAAAAAAAAGA-GAACCAACC-GCCTAAAAGAAGGGAACCATGCCGCGATTAAGAAAGGTATGAACTATAAA

GTACCAACCAAATGCCTTTGAAAGGAAATCCACAGGGGAGGAGTCTA

107_Campylopterus_curvipennis_Bris-24

GGATTATGGCTACTGCTACTTCTAGGATGGTTAGTAGGAGTAGTACGCAGGCGGTTAGAGCAGAGATTGCGGGTATGATG

GAGAATAGGACTGCGGTGGCTGTTGAGATGAGTTGGATTAGTAGGTGGCCTGCTGTTAGGTTAGCTGTGAGGCGGACTCC

TAGGGCTAGAGGGCGGATCAGGAGGCTGGTAGTTTCAATTATGATTAAGGCTGGGATTAGTGGAGTTGGGGTTCCTTCGG

GTAGGAGGTGGCCAAGTGAAGCGGATGGTTGGTTTCGGAGGCCTGTGAGTAGAGTGGCTAGCCATAGTGGGAAGGCTAGT

GCTAGGTTTATAGATAATTGTGTGGTAGGTGTGAATGTGTAGGGGAGTAGGCCTAGTAGGTTGATTGAGAGAAGGAAAAT

TATTAGTGATGACAGGATAAGGGCTCATTTGTGGCCTTTTTTGTTCAATGGGATTATCAGTTGTTTGGTGATTATATAGA

TGAATCACGATTGTAGGGTGGATGTTCGGTTGGTGATTCATCGGTTGTGGGGGGAGGGGAGTAGTAGGGCTGGGAATAAT

AGTGAGATGAGAATTAGGGGGATTCCTAGTAGGTGGGGGCTTATAAATTGGTCGAAGAAGCTTAGGTTCATGGTCAGGGT

CAGGATGAGGGTTTTGTGTTTGTTAGGGTTTTTTCTAGGGGGGAGTTAGTGGGCATGAATGAAAGGAGTTTGGGTTGGAT

GATTAGTGAAAAGGTCAGTCATGAAATAGTTATAATGAATAGTCATGGGTTTGGATTTAGTTGGGGCATATCATTAGGGA

GGGTTGGTAATCCTCTTTCTCTAGCTTAAAAGGCTAGTGCTGTTGCATAGCTTCCTAGTGGTTAAGATGATAGGAAAATT

AACATGATAAACATGATGATGGATGATGATTATTTTTTAATGTAATCTCGGTGTTGAATCTGGGTTAAAGTTAGATGATA

AAATGTGTGATGAAAATATCATGGATAGTATGGAAATTATAACTTAGAACTCCAGTGGTGTTGGTTAGAAAGTTAGAGGA

AGTGAAAAACTTGTAAAATATGTCCGGCAACCATAAATCAAAAAGCAACATAGCTTAGTAAGTATGTGAAAATACCATAA

CCAAATGCAAGACAAAGTGCATCAGTGTCAAAATGAGACTGAATTACACATGAAACCGTCTCATTCAGCAACTCTTGAAG

GAGAGAAAAGGCTCGGGAGCCACTGATGCCCAGGTCAAGAGATTGTGTGCTCCGCGTTAGCCACTGGAAGGGCCTATTGA

GATTACCCAAAAAAAAAGA-GAACCAACC-GCCTAAAAGAAGGGAACCATGCCGCGATTAAGAAAGGTATGAACTATAAA

GTACCAACCAAATGCCTTTGAAAGGAAATCCACAGGGGAGGAGTCTA

108_Campylopterus_curvipennis_Bris-25

GGATTATGGCTACTGCTACTTCTAGGATGGTTAGTAGGAGTAGTACGCAGGCGGTTAGAGCAGAGATTGCGGGTATGATG

GAGAATAGGACTGCGGTGGCTGTTGAGATGAGTTGGATTAGTAGGTGGCCTGCTGTTAGGTTAGCTGTGAGGCGGACTCC

TAGGGCTAGAGGGCGGATCAGGAGGCTGGTAGTTTCAATTATGATTAAGGCTGGGATTAGTGGAGTTGGGGTTCCTTCGG

GTAGGAGGTGGCCAAGTGAAGCGGATGGTTGGTTTCGGAGGCCTGTGAGTAGAGTGGCTAGTCATAGCGGGAAGGCTAGT

GCTAGGTTTATAGATAATTGTGTGGTAGGTGTGAATGTGTAGGGGAGTAGGCCTAGTAGGTTGATTGAGAGAAGGAAAAT

TATTAGTGATGACAGGATAAGGGCTCATTTGTGGCCTTTTTTGTTCAATGGGATTATCAGTTGTTTGGTGATTATATAGA

TGAATCACGATTGTAGGGTGGATGTTCGGTTGGTGATTCATCGGTTGTGGGGGGAGGGGAGTAGTAGGGCTGGGAATAAT

AGTGAGATGAGAATTAGGGGGATTCCTAGTAGGTGGGGGCTTATAAATTGGTCGAAGAAGCTTAGGTTCATGGTCAGGGT

CAGGATGAGGGTTTTGTGTTTGTTAGGGTTTTTTCTAGGGGGGAGTTAGTGGGCATGAATGAAAGGAGTTTGGGTTGGAT

GATTAGTGAAAAGGTCAGTCATGAAATAGTTATAATGAATAGTCATGGGTTTGGATTTAGTTGGGGCATATCATTAGGGA

GGGTTGGTAATCCTCTTTCTCTAGCTTAAAAGGCTAGTGCTGTTGCATAGCTTCCTAGTGGTTAAGATGATAGGAAAATT

AACATGATAAACATGATGATGGATGATGATTATTTTTTAATGTAATCTCGGTGTTGAATCTGGGTTAAAGTTAGATGATA

AAATGTGTGATGAAAATATCATGGATAGTATGGAAATTATAACTTAGAACTCCAGTGGTGTTGGTTAGAAAGTTAGAGGA

AGTGAAAAACTTGTAAAATATGTCCGGCAACCATAAATCAAAAAGCAACATAGCTTAGTAAGTATGTGAAAATACCATAA

CCAAATGCAAGACAAAGTGCATCAGTGTCAAAATGAGACTGAATTACACGTGAAACCGTCTCATTCAGCAACTCTTGAAG

GAGAGAAAAGGCTCGGGAGCCACTGATGCCCAGGTCAAGAGATTGTGTGCTCCGCGTTAGCCACTGGAAGGGCCTATTGA

GATTACCCAAAAAAAAAGA-GAACCAACC-GCCTAAAAGAAGGGAACCATGCCGCGATTAAGAAAGGTATGAACTATAAA

GTACCAACCAAATGCCTTTGAAAGGAAATCCACAGGGGAGGAGTCTA

109_Campylopterus_curvipennis_Bris-26

GGATTATGGCTACTGCTACTTCTAGGATGGTTAGTAGGAGTAGTACGCAGGCGGTTAGAGCAGAGATTGCGGGTATGATG

GAGAATAGGACTGCGGTGGCTGTTGAGATGAGTTGGATTAGTAGGTGGCCTGCTGTTAGGTTAGCTGTGAGGCGGACTCC

TAGGGCTAGAGGGCGGATCAGGAGGCTGGTAGTTTCAATTATGATTAAGGCTGGGATTAGTGGAGTTGGGGTTCCTTCGG

GTAGGAGGTGGCCAAGTGAAGCGGATGGTTGGTTTCGGAGGCCTGTGAGTAGAGTGGCTAGTCATAGTGGGAAGGCTAGT

GCTAGGTTTATAGATAATTGTGTGGTAGGTGTGAATGTGTAGGGGAGTAGGCCTAGTAGGTTGATTGAGAGAAGGAAAAT

TATTAGTGATGACAGGATAAGGGCTCATTTGTGGCCTTTTTTGTTCAATGGGATTATCAGTTGTTTGGTGATTATATAGA

TGAATCACGATTGTAGGGTGGATGTTCGGTTGGTGATTCATCGGTTGTGGGGGGAGGGGAGTAGTAGGGCTGGGAATAAT

AGTGAGATGAGAATTAGGGGGATTCCTAGTAGGTGGGGGCTTATAAATTGGTCGAAGAAGCTTAGGTTCATGGTCAGGGT

CAGGATGAGGGTTTTGTGTTTGTTAGGGGTTTTTCTAGGGGGGAGTTAGTGGGCATGAATGAAAGGAGTTTGGGTTGGAT

GATTAGTGAAAAGGTCAGTCATGAAATAGTTATAATGAATAGTCATGGGTTTGGATTTAGTTGGGGCATATCATTAGGGA

GGGTTGGTAATCCTCTTTCTCTAGCTTAAAAGGCTAGTGCTGTTGCATAGCTTCCTAGTGGTTAGGATGATAGGAAAATT

AACATGATAAACATGATGATGGATGATGATTATTTTTTAATGTAATCTCGGTGTTGAATCTGGGTTAAAGTTAGATGATA

AAATGTGTGATGAAAATATCATGGATAGTATGGAAATTATAACTTAGAACTCCAGTGGTGTTGGTTAGAAAGTTAGAGGA

AGTGAAAAACTTGTAAAATATGTCCGGCAACCATAAATCAAAAAGCAACATAGCTTAGTAAGTATGTGAAAATACCATAA

CCAAATGCAAGACAAAGTGCATCAGTGTCAAAATGAGACTGAATTACACATGAAACCGTCTCATTCAGCAACTCTTGAAG

GAGAGAAAAGGCCCGGGAGCCACTGATGCCCAGGTCAAGAGATTGTGTGCTCCGCGTTAGCCACTGGAAGGGCCTATTGA

GATTACCCAAAAAAAAAGA-GAACCAACC-GCCTAAAAGAAGGGAACCATGCCGCGATTAAGAAAGGTATGAACTATAAA

GTACCAACCAAATGCCTTTGAAAGGAAATCCACAGGGGAGGAATCTA

110_Campylopterus_curvipennis_Bris-27

GGATTATGGCTACTGCTACTTCTAGGATGGTTAGTAGGAGTAGTACGCAGGCGGTTAGAGCAGAGATTGCGGGTATGATG

GAGAATAGGACTGCGGTGGCTGTTGAGATGAGTTGGATTAGTAGGTGGCCTGCTGTTAGGTTAGCTGTGAGGCGGACTCC

TAGGGCTAGAGGGCGGATCAGGAGGCTGGTAGTTTCAATTATGATTAAGGCTGGGATTAGTGGAGTTGGGGTTCCTTCGG

GTAGGAGGTGGCCAAGTGAAGCGGATGGTTGGTTTCGGAGGCCTGTGAGTAGAGTGGCTAGTCATAGTGGGAAGGCTAGT

GCTAGGTTTATAGATAATTGTGTGGTAGGTGTGAATGTGTAGGGGAGTAGGCCTAGTAGGTTGATTGAGAGAAGGAAAAT

TATTAGTGATGACAGGATAAGGGCTCATTTGTGGCCTTTTTTGTTCAATGGGATTATCAGTTGTTTGGTGATTATATAGA

TGAATCACGATTGTAGGGTGGATGTTCGGTTGGTGATTCATCGGTTGTGGGGGGAGGGGAGTAGTAGGGCTGGGAATAAT

AGTGAGATGAGAATTAGGGGGATTCCTAGTAGGTGGGGGCTTATAAATTGGTCGAAGAAGCTTAGGTTCATGGTCAGGGT

CAGGATGAGGGTTTTGTGTTTGTTAGGGTTTTTTCTAGGGGGGAGTTAGTGGGCATGAATGAAAGGAGTTTGGGTTGGAT

GATTAGTGAAAAGGTCAGTCATGAAATAGTTATAATGAATAGTCATGGGTTTGGATTTAGTTGGGGCATATCATTAGGGA

GGGTTGGTAATCCTCTTTCTCTAGCTTAAAAGGCTAGTGCTGTTGCATAGCTTCCTAGTGGTTAAGATGATAGGAAAATT

AACATGATAAACATGATGATGGATGATGATTATTTTTTAATGTAATCTCGGTGTTGAATCTGGGTTAAAGTTAGATGATA

AAATGTGTGATGAAAATATCATGGATAGTATGGAAATTATAACTTAGAACTCCAGTGGTGTTGGTTAGAAAGTTAGAGGA

AGTGAAAAACTTGTAAAATATGTCCGGCAACCATAAATCAAAAAGCAACATAGCTTAGTAAGTATGTGAAAATACCATAA

CCAAATGCAAGACAAAGTGCATCAGTGTCAAAATGAGACTGAATTACACATGAAACCGTCTCATTCAGCAACTCTTGAAG

GAGAGAAAAGGCTCGGGAGCCACTGATGCCCAGGTCAAGAGATTGTGTGCTCCGCGTTAGCCACTGGAAGGGCCTATTGA

GATTACCCAAAAAAAAAGA-GAACCAACC-GCCTAAAAGAAGGGAACCATGCCGCGATTAAGAAAGGTATGAACTATAAA

GTACCAACCAAATGCCTTTGAAAGGAAATCCACAGGGGAGGAGTCTA

111_Campylopterus_curvipennis_Bris-28

GGATTATGGCTACTGCTACTTCTAGGATGGTTAGTAGGAGTAGTACGCAGGCGGTTAGAGCAGAGATTGCGGGTATGATG

GAGAATAGGACTGCGGTGGCTGTTGAGATGAGTTGGATTAGTAGGTGGCCTGCTGTTAGGTTAGCTGTGAGGCGGACTCC

TAGGGCTAGAGGGCGGATCAGGAGGCTGGTAGTTTCAATTATGATTAAGGCTGGGATTAGTGGAGTTGGGGTTCCTTCGG

GTAGGAGGTGGCCAAGTGAAGCGGATGGTTGGTTTCGGAGGCCTGTGAGTAGAGTGGCTAGTCATAGCGGGAAGGCTAGT

GCTAGGTTTATAGATAATTGTGTGGTAGGTGTGAATGTGTAGGGGAGTAGGCCTAGTAGGTTGATTGAGAGAAGGAAAAT

TATTAGTGATGACAGGATAAGGGCTCATTTGTGGCCTTTTTTGTTCAATGGGATTATCAGTTGTTTGGTGATTATATAGA

TGAATCACGATTGTAGGGTGGATGTTCGGTTGGTGATTCATCGGTTGTGGGGGGAGGGGAGTAGTAGGGCTGGGAATAAT

AGTGAGATGAGAATTAGGGGGATTCCTAGTAGGTGGGGGCTTATAAATTGGTCGAAGAAGCTTAGGTTCATGGTCAGGGT

CAGGATGAGGGTTTTGTGTTTGTTAGGGTTTTTTCTAGGGGGGAGTTAGTGGGCATGAATGAAAGGAGTTTGGGTTGGAT

GATTAGTGAAAAGGTCAGTCATGAAATAGTTATAATGAATAGTCATGGGTTTGGATTTAGTTGGGGCATATCATTAGGGA

GGGTTGGTAATCCTCTTTCTCTAGCTTAAAAGGCTAGTGCTGTTGCATAGCTTCCTAGTGGTTAAGATGATAGGAAAATT

AACATGATAAACATGATGATGGATGATGATTATTTTTTAATGTAATCTCGGTGTTGAATCTGGGTTAAAGTTAGATGATA

AAATGTGTGATGAAAATATCATGGATAGTATGGAAATTATAACTTAGAACTCCAGTGGTGTTGGTTAGAAAGTTAGAGGA

AGTGAAAAACTTGTAAAATATGTCCGGCAACCATAAATCAAAAAGCAACATAGCTTAGTAAGTATGTGAAAATACCATAA

CCAAATGCAAGACAAAGTGCATCAGTGTCAAAATGAGACTGAATTACACATGAAACCGTCTCATTCAGCAACTCTTGAAG

GAGAGAAAAGGCTCGGGAGCCACTGATGCCCAGGTCAAGAGATTGTGTGCTCCGCGTTAGCCACTGGAAGGGCCTATTGA

GATTACCCAAAAAAAAAGA-GAACCAACC-GCCTAAAAGAAGGGAACCATGCCGCGATTAAGAAAGGTATGAACTATAAA

GTACCAACCAAATGCCTTTGAAAGGAAATCCACAGGGGAGGAGTCTA

112_Campylopterus_curvipennis_Bris-29

GGATTATGGCTACTGCTACTTCTAGGATGGTTAGTAGGAGTAGTACGCAGGCGGTTAGAGCAGAGATTGCGGGTATGATG

GAGAATAGGACTGCGGTGGCTGTTGAGATGAGTTGGATTAGTAGGTGGCCTGCTGTTAGGTTAGCTGTGAGGCGGACTCC

TAGGGCTAGAGGGCGGATCAGGAGGCTGGTAGTTTCAATTATGATTAAGGCTGGGATTAGTGGAGTTGGGGTTCCTTCGG

GTAGGAGGTGGCCAAGTGAAGCGGATGGTTGGTTTCGGAGGCCTGTGAGTAGAGTGGCTAGTCATAGTGGGAAGGCTAGT

GCTAGGTTTATAGATAATTGTGTGGTAGGTGTGAATGTGTAGGGGAGTAGGCCTAGTAGGTTGATTGAGAGAAGGAAAAT

TATTAGTGATGACAGGATAAGGGCTCATTTGTGGCCTTTTTTGTTCAATGGGATTATCAGTTGTTTGGTGATTATATAGA

TGAATCACGATTGTAGGGTGGATGTTCGGTTGGTGATTCATCGGTTGTGGGGGGAGGGGAGTAGTAGGGCTGGGAATAAT

AGTGAGATGAGAATTAGGGGGATTCCTAGTAGGTGGGGGCTTATAAATTGGTCGAAGAAGCTTAGGTTCATGGTCAGGGT

CAGGATGAGGGTTTTGTGTTTGTTAGGGTTTTTTCTAGGGGGGAGTTAGTGGGCATGAATGAAAGGAGTTTGGGTTGGAT

GATTAGTGAAAAGGTCAGTCATGAAATAGTTATAATGAATAGTCATGGGTTTGGATTTAGTTGGGGCATATCATTAGGGA

GGGTTGGTAATCCTCTTTCTCTAGCTTAAAAGGCTAGTGCTGTTGCATAGCTTCCTAGTGGTTAAGATGATAGGAAAATT

AACATGATAAACATGATGATGGATGATGATTATTTTTTAATGTAATCTCGGTGTTGAATCTGGGTTAAAGTTAGATGATA

AAATGTGTGATGAAAATATCATGGATAGTATGGAAATTATAACTTAGAACTCCAGTGGTGTTGGTTAGAAAGTTAGAGGA

AGTGAAAAACTTGTAAAATATGTCCGGCAACCATAAATCAAAAAGCAACATAGCTTAGTAAGTATGTGAAAATACCATAA

CCAAATGCAAGACAAAGTGCATCAGTGTCAAAATGAGACTGAATTACACATGAAACCGTCTCATTCAGCAACTCTTGAAG

GAGAGAAAAGGCTCGGGAGCCACTGATGCCCAGGTCAAGAGATTGTGTGCTCCGCGTTAGCCACTGGAAGGGCCTATTGA

GATTACCCAAAAAAAAAGA-GAACCAACC-GCCTAAAAGAAGGGAACCATGCCGCGATTAAGAAAGGTATGAACTATAAA

GTACCAACCAAATGCCTTTGAAAGGAAATCCACAGGGGAGGAGTCTA

113_Campylopterus_curvipennis_Bris-30

GGATTATGGCTACTGCTACTTCTAGGATGGTTAGTAGGAGTAGTACGCAGGCGGTTAGAGCAGAGATTGCGGGTATGATG

GAGAATAGGACTGCGGTGGCTGTTGAGATGAGTTGGATTAGTAGGTGGCCTGCTGTTAGGTTAGCTGTGAGGCGGACTCC

TAGGGCTAGAGGGCGGATCAGGAGGCTGGTAGTTTCAATTATGATTAAGGCTGGGATTAGTGGAGTTGGGGTTCCTTCGG

GTAGGAGGTGGCCAAGTGAAGCGGATGGTTGGTTTCGGAGGCCTGTGAGTAGAGTGGCTAGTCATAGTGGGAAGGCTAGT

GCTAGGTTTATAGATAATTGTGTGGTAGGTGTGAATGTGTAGGGGAGTAGGCCTAGTAGGTTGATTGAGAGAAGGAAAAT

TATTAGTGATGACAGGATAAGGGCTCATTTGTGGCCTTTTTTGTTTAATGGGATTATCAGTTGTTTGGTGATTATATAGA

TGAATCACGATTGTAGGGTGGATGTTCGGTTGGTGATTCATCGGTTGTGGGGGGAGGGGAGTAGTAGGGCTGGGAATAAT

AGTGAGATGAGAATTAGGGGGATTCCTAGTAGGTGGGGGCTTATAAATTGGTCGAAGAAGCTTAGGTTCATGGTCAGGGT

CAGGATGAGGGTTTTGTGTTTGTTAGGGGTTTTTCTAGGGGGGAGTTAGTGGGCATGAATGAAAGGAGTTTGGGTTGGAT

GATTAGTGAAAAGGTCAGTCATGAAATAGTTATAATGAATAGTCATGGGTTTGGATTTAGTTGGGGCATATCATTAGGGA

GGGTTGGTAATCCTCTTTCTCTAGCTTAAAAGGCTAGTGCTGTTGCATAGCTTCCTAGTGGTTAAGATGATAGGAAAATT

AACATGATAAACATGATGATGGATGATGATTATTTTTTAATGTAATCTCGGTGTTGAATCTGGGTTAAAGTTAGATGATA

AAATGTGTGATGAAAATATCATGGATAGTATGGAAATTATAACTTAGAACTCCAGTGGTGTTGGTTAGAAAGTTAGAGGA

AGTGAAAAACTTGTAAAATATGTCCGGCAACCATAAATCAAAAAGCAACATAGCTTAGTAAGTATGTGAAAATACCATAA

CCAAATGCAAGACAAAGTGCATCAGTGTCAAAATGAGACTGAATTACACATGAAACCGTCTCATTCAGCAACTCTTGAAG

GAGAGAAAAGGCTCGGGAGCCACTGATGCTCAGGTCAAGAGATTGTGTGCTCCGCGTTAGCCACTGGAAGGGCCTATTGA

GATTACCCAAAAAAAAAGA-GAACCAACC-GCCTAAAAGAAGGGAACCATGCCGCGATTAAGAAAGGTATGAACTATAAA

GTACCAACCAAATGCCTTTGAAAGGAAATCCACAGGGGAGAAGTCTA

114_Campylopterus_curvipennis_Xico-1

GGATTATGGCTACTGCTACTTCTAGGATGGTTAGTAGGAGTAGTACGCAGGCGGTTAGAGCAGAGATTGCGGGTATGATG

GAGAATAGGACTGCGGTGGCTGTTGAGATGAGTTGGATTAGTAGGTGGCCTGCTGTTAGGTTAGCTGTGAGGCGGACTCC

TAGGGCTAGAGGGCGGATCAGGAGGCTGGTAGTTTCAATTATGATTAAGGCTGGGATTAGTGGAGTTGGGGTTCCTTCGG

GTAGGAGGTGGCCAAGTGAAGCGGATGGTTGGTTTCGGAGGCCTGTGAGTAGAGTGGCTAGTCATAGTGGGAAGGCTAGT

GCTAGGTTTATAGATAATTGTGTGGTAGGTGTGAATGTGTAGGGGAGTAGGCCTAGTAGGTTGATTGAGAGAAGGAAAAT

TATTAGTGATGACAGGATAAGGGCTCATTTGTGGCCTTTTTTGTTCAATGGGATTATCAGTTGTTTGGTGATTATATAGA

TGAATCACGATTGTAGGGTGGATGTTCGGTTGGTGATTCATCGGTTGTGGGGGGAGGGGAGTAGTAGGGCTGGGAATAAT

AGTGAGATGAGAATTAGGGGGATTCCTAGTAGGTGGGGGCTTATAAATTGGTCGAAGAAGCTTAGGTTCATGGTCAGGGT

CAGGATGAGGGTTTTGTGTTTGTTAGGGGTTTTTCTAGGGGGGAGTTAGTGGGCATGAATGAAAGGAGTTTGGGTTGGAT

GATTAGTGAAAAGGTCAGTCATGAAATAGTTATAATGAATAGTCATGGGTTTGGATTTAGTTGGGGCATATCATTAGGGA

GGGTTGGTAATCCTCTTTCTCTAGCTTAAAAGGCTAGTGCTGTTGCATAGCTTCCTAGTGGTTAGGATGATAGGAAAATT

AACATGATAAACATGATGATGGATGATGATTATTTTTTAATGTAATCTCGGTGTTGAATCTGGGTTAAAGTTAGATGATA

AAATGTGTGATGAAAATATCATGGATAGTATGGAAATTATAACTTAGAACTCCAGTGGTGTTGGTTAGAAAGTTAGAGGA

AGTGAAAAACTTGTAAAATATGTCCGGCAACCATAAATCAAAAAGCAACATAGCTTAGTAAGTATGTGAAAATACCATAA

CCAAATGCAAGACAAAGTGCATCAGTGTCAAAATGAGACTGAATTACACATGAAACCGTCTCATTCAGCAACTCTTGAAG

GAGAGAAAAGGCCCGGGAGCCACTGATGCCCAGGTCAAGAGATTGTGTGCTCCGCGTTAGCCACTGGAAGGGCCTATTGA

GATTACCCAAAAAAAAAGA-GAACCAACC-GCCTAAAAGAAGGGAACCATGCCGCGATTAAGAAAGGTATGAACTATAAA

GTACCAACCAAATGCCTTTGAAAGGAAATCCACAGGGGAGGAATCTA

115_Campylopterus_curvipennis_Xico-2

GGATTATGGCTACTGCTACTTCTAGGATGGTTAGTAGGAGTAGTACGCAGGCGGTTAGAGCAGAGATTGCGGGTATGATG

GAGAATAGGACTGCGGTGGCTGTTGAGATGAGTTGGATTAGTAGGTGGCCTGCTGTTAGGTTAGCTGTGAGGCGGACTCC

TAGGGCTAGAGGGCGGATCAGGAGGCTGGTAGTTTCAATTATAATTAAGGCTGGGATTAGTGGAGTTGGGGTTCCTTCGG

GTAGGAGGTGGCCAAGTGAAGCGGATGGTTGGTTTCGGAGGCCTGTGAGTAGAGTGGCTAGTCATAGTGGGAAGGCTAGT

GCTAGGTTTATAGATAATTGTGTGGTAGGTGTGAATGTGTAGGGGAGTAGGCCTAGTAGGTTGATTGAGAGAAGGAAAAT

TATTAGTGATGACAGGATAAGGGCTCATTTGTGGCCTTTTTTGTTCAATGGGATTATCAGTTGTTTGGTGATTATATAGA

TGAATCACGATTGTAGGGTGGATGTTCGGTTGGTGATTCATCGGTTGTGGGGGGAGGGGAGTAGTAGGGCTGGGAATAAT

AGTGAGATGAGAATTAGGGGGATTCCTAGTAGGTGGGGGCTTATAAATTGGTCGAAGAAGCTTAGGTTCATGGTCAGGGT

CAGGATGAGGGTTTTGTGTTTGTTAGGGGTTTTTCTAGGGGGGAGTTAGTGGGCATGAATGAAAGGAGTTTGGGTTGGAT

GATTAGTGAAAAGGTCAGTCATGAAATAGTTATAATGAATAGTCATGGGTTTGGATTTAGTTGGGGCATATCATTAGGGA

GGGTTGGTAATCCTCTTTCTCTAGCTTAAAAGGCTAGTGCTGTTGCATAGCTTCCTAGTGGTTAAGATGATAGGAAAATT

AACATGATAAACATGATGATGGATGATGATTATTTTTTAATGTAATCTCGGTGTTGAATCTGGGTTAAAGTTAGATGATA

AAATGTGTGATGAAAATATCATGGATAGTATGGAAATTATAACTTAGAACTCCAGTGGTGTTGGTTAGAAAGTTAGAGGA

AGTGAAAAACTTGTAAAATATGTCCGGCAACCATAAATCAAAAAGCAACATAGCTTAGTAAGTATGTGAAAATACCATAA

CCAAATGCAAGACAAAGTGCATCAGTGTCAAAATGAGACTGAATTACACATGAAACCGTCTCATTCAGCAACTCTTGAAG

GAGAGAAAAGGCTCGGGAGCCACTGATGCTCAGGTCAAGAGATTGTGTGCTCCGCGTTAGCCACTGGAAGGGCCTATTGA

GATTACCCAAAAAAAAAGA-GAACCAACC-GCCTAAAAGAAGGGAACCATGCCGCGATTAAGAAAGGTATGAACTATAAA

GTACCAACCAAATGCCTTTGAAAGGAAGCCCACAGGGGAGGAGTCTA

116_Campylopterus_curvipennis_Xico-3

GGATTATGGCTACTGCTACTTCTAGGATGGTTAGTAGGAGTAGTACGCAGGCGGTTAGAGCAGAGATTGCGGGTATGATG

GAGAATAGGACTGCGGTGGCTGTTGAGATGAGTTGGATTAGTAGGTGGCCTGCTGTTAGGTTAGCTGTGAGGCGGACTCC

TAGGGCTAGAGGGCGGATCAGGAGGCTGGTAGTTTCAATTATAATTAAGGCTGGGATTAGTGGAGTTGGGGTTCCTTCGG

GTAGGAGGTGGCCAAGTGAAGCGGATGGTTGGTTTCGGAGGCCTGTGAGTAGAGTGGCTAGTCATAGTGGGAAGGCTAGT

GCTAGGTTTATAGATAATTGTGTGGTAGGTGTGAATGTGTAGGGGAGTAGGCCTAGTAGGTTGATTGAGAGAAGGAAAAT

TATTAGTGATGACAGGATAAGGGCTCATTTGTGGCCTTTTTTGTTCAATGGGATTATCAGTTGTTTGGTGATTATATAGA

TGAATCACGATTGTAGGGTGGATGTTCGGTTGGTGATTCATCGGTTGTGGGGGGAGGGGAGTAGTAGGGCTGGGAATAAT

AGTGAGATGAGAATTAGGGGGATTCCTAGTAGGTGGGGGCTTATAAATTGGTCGAAGAAGCTTAGGTTCATGGTCAGGGT

CAGGATGAGGGTTTTGTGTTTGTTAGGGGTTTTTCTAGGGGGGAGTTAGTGGGCATGAATGAAAGGAGTTTGGGTTGGAT

GATTAGTGAAAAGGTCAGTCATGAAATAGTTATAATGAATAGTCATGGGTTTGGATTTAGTTGGGGCATATCATTAGGGA

GGGTTGGTAATCCTCTTTCTCTAGCTTAAAAGGCTAGTGCTGTTGCATAGCTTCCTAGTGGTTAAGATGATAGGAAAATT

AACATGATAAACATGATGATGGATGATGATTATTTTTTAATGTAATCTCGGTGTTGAATCTGGGTTAAAGTTAGATGATA

AAATGTGTGATGAAAATATCATGGATAGTATGGAAATTATAACTTAGAACTCCAGTGGTGTTGGTTAGAAAGTTAGAGGA

AGTGAAAAACTTGTAAAATATGTCCGGCAACCATAAATCAAAAAGCAACATAGCTTAGTAAGTATGTGAAAATACCATAA

CCAAATGCAAGACAAAGTGCATCAGTGTCAAAATGAGACTGAATTACACATGAAACCGTCTCATTCAGCAACTCTTGAAG

GAGAGAAAAGGCTCGGGAGCCACTGATGCTCAGGTCAAGAGATTGTGTGCTCCGCGTTAGCCACTGGAAGGGCCTATTGA

GATTACCCAAAAAAAAAGA-GAACCAACC-GCCTAAAAGAAGGGAACCATGCCGCGATTAAGAAAGGTATGAACTATAAA

GTACCAACCAAATGCCTTTGAAAGGAAGCCCACAGGGGAGGAGTCTA

117_Campylopterus_curvipennis_Xico-4

GGATTATGGCTACTGCTACTTCTAGGATGGTTAGTAGGAGTAGTACGCAGGCGGTTAGAGCAGAGATTGCGGGTATGATG

GAGAATAGGACTGCGGTGGCTGTTGAGATGAGTTGGATTAGTAGGTGGCCTGCTGTTAGGTTAGCTGTGAGGCGGACTCC

TAGGGCTAGAGGGCGGATCAGGAGGCTGGTAGTTTCAATTATGATTAAGGCTGGGATTAGTGGAGTTGGGGTTCCTTCGG

GTAGGAGGTGGCCAAGTGAAGCGGATGGTTGGTTTCGGAGGCCTGTGAGTAGAGTGGCTAGTCATAGTGGGAAGGCTAGT

GCTAGGTTTATAGATAATTGTGTGGTAGGTGTGAATGTGTAGGGGAGTAGGCCTAGTAGGTTGATTGAGAGAAGGAAAAT

TATTAGTGATGACAGGATAAGGGCTCATTTGTGGCCTTTTTTGTTCAATGGGATTATCAGTTGTTTGGTGATTATATAGA

TGAATCACGATTGTAGGGTGGATGTTCGGTTGGTGATTCATCGGTTGTGGGGGGAGGGGAGTAGTAGGGCTGGGAATAAT

AGTGAGATGAGAATTAGGGGGATTCCTAGTAGGTGGGGGCTTATAAATTGGTCGAAGAAGCTTAGGTTCATGGTCAGGGT

CAGGATGAGGGTTTTGTGTTTGTTAGGGTTTTTTCTAGGGGGGAGTTAGTGGGCATGAATGAAAGGAGTTTGGGTTGGAT

GATTAGTGAAAAGGTCAGTCATGAAATAGTTATAATGAATAGTCATGGGTTTGGATTTAGTTGGGGCATATCATTAGGGA

GGGTTGGTAATCCTCTTTCTCTAGCTTAAAAGGCTAGTGCTGTTGCATAGCTTCCTAGTGGTTAAGATGATAGGAAAATT

AACATGATAAACATGATGATGGATGATGATTATTTTTTAATGTAATCTCGGTGTTGAATCTGGGTTAAAGTTAGATGATA

AAATGTGTGATGAAAATATCATGGATAGTATGGAAATTATAACTTAGAACTCCAGTGGTGTTGGTTAGAAAGTTAGAGGA

AGTGAAAAACTTGTAAAATATGTCCGGCAACCATAAATCAAAAAGCAACATAGCTTAGTAAGTATGTGAAAATACCATAA

CCAAATGCAAGACAAAGTGCATCAGTGTCAAAATGAGACTGAATTACACATGAAACCGTCTCATTCAGCAACTCTTGAAG

GAGAGAAAAGGCTCGGGAGCCACTGATGCCCAGGTCAAGAGATTGTGTGCTCCGCGTTAGCCACTGGAAGGGCCTATTGA

GATTACCCAAAAAAAAAGA-GAACCAACC-GCCTAAAAGAAGGGAACCATGCCGCGATTAAGAAAGGTATGAACTATAAA

GTACCAACCAAATGCCTTTGAAAGGAAATCCACAGGGGAGGAGTCTA

118_Campylopterus_curvipennis_Ama-1

GGATTATGGCTACTGCTACTTCTAGGATGGTTAGTAGGAGTAGTACGCAGGCGGTTAGAGCAGAGATTGCGGGTATGATG

GAGAATAGGACTGCGGTGGCTGTTGAGATGAGTTGGATTAGTAGGTGGCCTGCTGTTAGGTTAGCTGTGAGGCGGACTCC

TAGGGCTAGAGGGCGGATCAGGAGGCTGGTAGTTTCAATTATGATTAAGGCTGGGATTAGTGGAGTTGGGGTTCCTTCGG

GTAGGAGGTGGCCAAGTGAAGCGGATGGTTGGTTTCGGAGGCCTGTGAGTAGAGTGGCTAGTCATAGTGGGAAGGCTAGT

GCTAGGTTTATAGATAATTGTGTGGTAGGTGTGAATGTGTAGGGGAGTAGGCCTAGTAGGTTGATTGAGAGAAGGAAAAT

TATTAGTGATGACAGGATAAGGGCTCATTTGTGGCCTTTTTTGTTTAATGGGATTATCAGTTGTTTGGTGATTATATAGA

TGAATCACGATTGTAGGGTGGATGTTCGGTTGGTGATTCATCGGTTGTGGGGGGAGGGGAGTAGTAGGGCTGGGAATAAT

AGTGAGATGAGAATTAGGGGGATTCCTAGTAGGTGGGGGCTTATAAATTGGTCGAAGAAGCTTAGGTTCATGGTCAGGGT

CAGGATGAGGGTTTTGTGTTTGTTAGGGGTTTTTCTAGGGGGGAGTTAGTGGGCATGAATGAAAGGAGTTTGGGTTGGAT

GATTAGTGAAAAGGTCAGTCATGAAATAGTTATAATGAATAGTCATGGGTTTGGATTTAGTTGGGGCATATCATTAGGGA

GGGTTGGTAATCCTCTTTCTCTAGCTTAAAAGGCTAGTGCTGTTGCATAGCTTCCTAGTGGTTAAGATGATAGGAAAATT

AACATGATAAACATGATGATGGATGATGATTATTTTTTAATGTAATCTCGGTGTTGAATCTGGGTTAAAGTTAGATGATA

AAATGTGTGATGAAAATATCATGGATAGTATGGAAATTATAACTTAGAACTCCAGTGGTGTTGGTTAGAAAGTTAGAGGA

AGTGAAAAACTTGTAAAATATGTCCGGCAACCATAAATCAAAAAGCAACATAGCTTAGTAGGTATGTGAAAATACCATAA

CCAAATGCAAGACAAAGTGCATCAGTGTCAAAATGAGACTGAATTACACATGAAACCGTCTCATTCAGCAACTCTTGAAG

GAGAGAAAAGGCCCGGGAGCCACTGATGCTCAGGTCAAGAGATTGTGTGCTCCGCGTTAGCCACTGGAAGGGCCTATTGA

GATTACCCAAAAAAAAAGA-GAACCAACC-GCCTAAAAGAAGGGAACCATGCCGCGATTAAGAAAGGTATGAACTATAAA

GTACCAACCAAATGCCTTTGAAAGGAAATCCACAGGGGAGGAGTCTA

119_Campylopterus_curvipennis_Ama-2

GGATTATGGCTACTGCTACTTCTAGGATGGTTAGTAGGAGTAGTACGCAGGCGGTTAGAGCAGAGATTGCGGGTATGATG

GAGAATAGGACTGCGGTGGCTGTTGAGATGAGTTGGATTAGTAGGTGGCCTGCTGTTAGGTTAGCTGTGAGGCGGACTCC

TAGGGCTAGAGGGCGGATCAGGAGGCTGGTAGTTTCAATTATGATTAAGGCTGGGATTAGTGGAGTTGGGGTTCCTTCGG

GTAGGAGGTGGCCAAGTGAAGCGGATGGTTGGTTTCGGAGGCCTGTGAGTAGAGTGGCTAGTCATAGTGGGAAGGCTAGT

GCTAGGTTTATAGATAATTGTGTGGTAGGTGTGAATGTGTAGGGGAGTAGGCCTAGTAGGTTGATTGAGAGAAGGAAAAT

TATTAGTGATGACAGGATAAGGGCTCATTTGTGGCCTTTTTTGTTCAATGGGATTATCAGTTGTTTGGTGATTATATAGA

TGAATCACGATTGTAGGGTGGATGTTCGGTTGGTGATTCATCGGTTGTGGGGGGAGGGGAGTAGTAGGGCTGGGAATAAT

AGTGAGATGAGAATTAGGGGGATTCCTAGTAGGTGGGGGCTTATAAATTGGTCGAAGAAGCTTAGGTTCATGGTCAGGGT

CAGGATGAGGGTTTTGTGTTTGTTAGGGTTTTTTCTAGGGGGGAGTTAGTGGGCATGAATGAAAGGAGTTTGGGTTGGAT

GATTAGTGAAAAGGTCAGTCATGAAATAGTTATAATGAATAGTCATGGGTTTGGATTTAGTTGGGGCATATCATTAGGGA

GGGTTGGTAATCCTCTTTCTCTAGCTTAAAAGGCTAGTGCTGTTGCATAGCTTCCTAGTGGTTAAGATGATAGGAAAATT

AACATGATAAACATGATGATGGATGATGATTATTTTTTAATGTAATCTCGGTGTTGAATCTGGGTTAAAGTTAGATGATA

AAATGTGTGATGAAAATATCATGGATAGTATGGAAATTATAACTTAGAACTCCAGTGGTGTTGGTTAGAAAGTTAGAGGA

AGTGAAAAACTTGTAAAATATGTCCGGCAACCATAAATCAAAAAGCAACATAGCTTAGTAAGTATGTGAAAATACCATAA

CCAAATGCAAGACAAAGTGCATCAGTGTCAAAATGAGACTGAATTACACATGAAACCGTCTCATTCAGCAACTCTTGAAG

GAGAGAAAAGGCTCGGGAGCCACTGATGCCCAGGTCAAGAGATTGTGTGCTCCGCGTTAGCCACTGGAAGGGCCTATTGA

GATTACCCAAAAAAAAAGA-GAACCAACC-GCCTAAAAGAAGGGAACCATGCCGCGATTAAGAAAGGTATGAACTATAAA

GTACCAACCAAATGCCTTTGAAAGGAAATCCACAGGGGAGGAGTCTA

120_Campylopterus_curvipennis_Ama-3

GGATTATGGCTACTGCTACTTCTAGGATGGTTAGTAGGAGTAGTACGCAGGCGGTTAGAGCAGAGATTGCGGGTATGATG

GAGAATAGGACTGCGGTGGCTGTTGAGATGAGTTGGATTAGTAGGTGGCCTGCTGTTAGGTTAGCTGTGAGGCGGACTCC

TAGGGCTAGAGGGCGGATCAGGAGGCTGGTAGTTTCAATTATGATTAAGGCTGGGATTAGTGGAGTTGGGGTTCCTTCGG

GTAGGAGGTGGCCAAGTGAAGCGGATGGTTGGTTTCGGAGGCCTGTGAGTAGAGTGGCTAGTCATAGCGGGAAGGCTAGT

GCTAGGTTTATAGATAATTGTGTGGTAGGTGTGAATGTGTAGGGGAGTAGGCCTAGTAGGTTGATTGAGAGAAGGAAAAT

TATTAGTGATGACAGGATAAGGGCTCATTTGTGGCCTTTTTTGTTCAATGGGATTATCAGTTGTTTGGTGATTATATAGA

TGAATCACGATTGTAGGGTGGATGTTCGGTTGGTGATTCATCGGTTGTGGGGGGAGGGGAGTAGTAGGGCTGGGAATAAT

AGTGAGATGAGAATTAGGGGGATTCCTAGTAGGTGGGGGCTTATAAATTGGTCGAAGAAGCTTAGGTTCATGGTCAGGGT

CAGGATGAGGGTTTTGTGTTTGTTAGGGTTTTTTCTAGGGGGGAGTTAGTGGGCATGAATGAAAGGAGTTTGGGTTGGAT

GATTAGTGAAAAGGTCAGTCATGAAATAGTTATAATGAATAGTCATGGGTTTGGATTTAGTTGGGGCATATCATTAGGGA

GGGTTGGTAATCCTCTTTCTCTAGCTTAAAAGGCTAGTGCTGTTGCATAGCTTCCTAGTGGTTAAGATGATAGGAAAATT

AACATGATAAACATGATGATGGATGATGATTATTTTTTAATGTAATCTCGGTGTTGAATCTGGGTTAAAGTTAGATGATA

AAATGTGTGATGAAAATATCATGGATAGTATGGAAATTATAACTTAGAACTCCAGTGGTGTTGGTTAGAAAGTTAGAGGA

AGTGAAAAACTTGTAAAATATGTCCGGCAACCATAAATCAAAAAGCAACATAGCTTAGTAAGTATGTGAAAATACCATAA

CCAAATGCAAGACAAAGTGCATCAGTGTCAAAATGAGACTGAATTACACATGAAACCGTCTCATTCAGCAACTCTTGAAG

GAGAGAAAAGGCTCGGGAGCCACTGATGCCCAGGTCAAGAGATTGTGTGCTCCGCGTTAGCCACTGGAAGGGCCTATTGA

GATTACCCAAAAAAAAAGA-GAACCAACC-GCCTAAAAGAAGGGAACCATGCCGCGATTAAGAAAGGTATGAACTATAAA

GTACCAACCAAATGCCTTTGAAAGGAAATCCACAGGGGAGGAGTCTA

121_Campylopterus_curvipennis_INECOL-1

GGATTATGGCTACTGCTACTTCTAGGATGGTTAGTAGGAGTAGTACGCAGGCGGTTAGAGCAGAGATTGCGGGTATGATG

GAGAATAGGACTGCGGTGGCTGTTGAGATGAGTTGGATTAGTAGGTGGCCTGCTGTTAGGTTAGCTGTGAGGCGGACTCC

TAGGGCTAGAGGGCGGATCAGGAGGCTGGTAGTTTCAATTATGATTAAGGCTGGGATTAGTGGAGTTGGGGTTCCTTCGG

GTAGGAGGTGGCCAAGTGAAGCGGATGGTTGGTTTCGGAGGCCTGTGAGTAGAGTGGCTAGTCATAGTGGGAAGGCTAGT

GCTAGGTTTATAGATAATTGTGTGGTAGGTGTGAATGTGTAGGGGAGTAGGCCTAGTAGGTTGATTGAGAGAAGGAAAAT

TATTAGTGATGACAGGATAAGGGCTCATTTGTGGCCTTTTTTGTTCAATGGGATTATCAGTTGTTTGGTGATTATATAGA

TGAATCACGATTGTAGGGTGGATGTTCGGTTGGTGATTCATCGGTTGTGGGGGGAGGGGAGTAGTAGGGCTGGGAATAAT

AGTGAGATGAGAATTAGGGGGATTCCTAGTAGGTGGGGGCTTATAAATTGGTCGAAGAAGCTTAGGTTCATGGTCAGGGT

CAGGATGAGGGTTTTGTGTTTGTTAGGGGTTTTTCTAGGGGGGAGTTAGTGGGCATGAATGAAAGGAGTTTGGGTTGGAT

GATTAGTGAAAAGGTCAGTCATGAAATAGTTATAATGAATAGTCATGGGTTTGGATTTAGTTGGGGCATATCATTAGGGA

GGGTTGGTAATCCTCTTTCTCTAGCTTAAAAGGCTAGTGCTGTTGCATAGCTTCCTAGTGGTTAAGATGATAGGAAAATT

AACATGATAAACATGATGATGGATGATGATTATTTTTTAATGTAATCTCGGTGTTGAATCTGGGTTAAAGTTAGATGATA

AAATGTGTGATGAAAATATCATGGATAGTATGGAAATTATAACTTAGAACTCCAGTGGTGTTGGTTAGAAAGTTAGAGGA

AGTGAAAAACTTGTAAAATATGTCCGGCAACCATAAATCAAAAAGCAACATAGCTTAGTAAGTATGTGAAAATACCATAA

CCAAATGCAAGACAAAGTGCATCAGTGTCAAAATGAGACTGAATTACACATGAAACCGTCTCATTCAGCAACTCTTGAAG

GAGAGAAAAGGCTCGGGAGCCACTGATGCCCAGGTCAAGAGATTGTGTGCTCCGCGTTAGCCACTGGAAGGGCCTATTGA

GATTACCCAAAAAAAAAGA-GAACCAACC-GCCTAAAAGAAGGGAACCATGCCGCGATTAAGAAAGGTATGAACTATAAA

GTACCAACCAAATGCCTTTGAAAGGAAGTCCACAGGGGAGGAGTCTA

122_Campylopterus_curvipennis_INECOL-A

GGATTATGGCTACTGCTACTTCTAGGATGGTTAGTAGGAGTAGTACGCAGGCGGTTAGAGCAGAGATTGCGGGTATGATG

GAGAATAGGACTGCGGTGGCTGTTGAGATGAGTTGGATTAGTAGGTGGCCTGCTGTTAGGTTAGCTGTGAGGCGGACTCC

TAGGGCTAGAGGGCGGATCAGGAGGCTGGTAGTTTCAATTATGATTAAGGCTGGGATTAGTGGAGTTGGGGTTCCTTCGG

GTAGGAGGTGGCCAAGTGAAGCGGATGGTTGGTTTCGGAGGCCTGTGAGTAGAGTGGCTAGTCATAGTGGGAAGGCTAGT

GCTAGGTTTATAGATAATTGTGTGGTAGGTGTGAATGTGTAGGGGAGTAGGCCTAGTAGGTTGATTGAGAGAAGGAAAAT

TATTAGTGATGACAGGATAAGGGCTCATTTGTGGCCTTTTTTGTTCAATGGGATTATCAGTTGTTTGGTGATTATATAGA

TGAATCACGATTGTAGGGTGGATGTTCGGTTGGTGATTCATCGGTTGTGGGGGGAGGGGAGTAGTAGGGCTGGGAATAAT

AGTGAGATGAGAATTAGGGGGATTCCTAGTAGGTGGGGGCTTATAAATTGGTCGAAGAAGCTTAGGTTCATGGTCAGGGT

CAGGATGAGGGTTTTGTGTTTGTTAGGGGTTTTTCTAGGGGGGAGTTAGTGGGCATGAATGAAAGGAGTTTGGGTTGGAT

GATTAGTGAAAAGGTCAGTCATGAAATAGTTATAATGAATAGTCATGGGTTTGGATTTAGTTGGGGCATATCATTAGGGA

GGGTTGGTAATCCTCTTTCTCTAGCTTAAAAGGCTAGTGCTGTTGCATAGCTTCCTAGTGGTTAAGATGATAGGAAAATT

AACATGATAAACATGATGATGGATGATGATTATTTTTTAATGTAATCTCGGTGTTGAATCTGGGTTAAAGTTAGATGATA

AAATGTGTGATGAAAATATCATGGATAGTATGGAAATTATAACTTAGAACTCCAGTGGTGTTGGTTAGAAAGTTAGAGGA

AGTGAAAAACTTGTAAAATATGTCCGGCAACCATAAATCAAAAAGCAACATAGCTTAGTAAGTATGTGAAAATACCATAA

CCAAATGCAAGACAAAGTGCATCAGTGTCAAAATGAGACTGAATTACACATGAAACCGTCTCATTCAGCAACTCTTGAAG

GAGAGAAAAGGCCCGGGAGCCACTGATGCCCAGGTCAAGAGATTGTGTGCTCCGCGTTAGCCACTGGAAGGGCCTATTGA

GATTACCCAAAAAAAAAGA-GAACCAACC-GCCTAAAAGAAGGGAACCATGCCGCGATTAAGAAAGGTATGAACTATAAA

GTACCAACCAAATGCCTTTGAAAGGAAATCCACAGGGGAGGAATCTA

123_Campylopterus_curvipennis_INECOL-B

GGATTATGGCTACTGCTACTTCTAGGATGGTTAGTAGGAGTAGTACGCAGGCGGTTAGAGCAGAGATTGCGGGTATGATG

GAGAATAGGACTGCGGTGGCTGTTGAGATGAGTTGGATTAGTAGGTGGCCTGCTGTTAGGTTAGCTGTGAGGCGGACTCC

TAGGGCTAGAGGGCGGATCAGGAGGCTGGTAGTTTCAATTATGATTAAGGCTGGGATTAGTGGAGTTGGGGTTCCTTCGG

GTAGGAGGTGGCCAAGTGAAGCGGATGGTTGGTTTCGGAGGCCTGTGAGTAGAGTGGCTAGTCATAGTGGGAAGGCTAGT

GCTAGGTTTATAGATAATTGTGTGGTAGGTGTGAATGTGTAGGGGAGTAGGCCTAGTAGGTTGATTGAGAGAAGGAAAAT

TATTAGTGATGACAGGATAAGGGCTCATTTGTGGCCTTTTTTGTTCAATGGGATTATCAGTTGTTTGGTGATTATATAGA

TGAATCACGATTGTAGGGTGGATGTTCGGTTGGTGATTCATCGGTTGTGGGGGGAGGGGAGTAGTAGGGCTGGGAATAAT

AGTGAGATGAGAATTAGGGGGATTCCTAGTAGGTGGGGGCTTATAAATTGGTCGAAGAAGCTTAGGTTCATGGTCAGGGT

CAGGATGAGGGTTTTGTGTTTGTTAGGGTTTTTTCTAGGGGGGAGTTAGTGGGCATGAATGAAAGGAGTTTGGGTTGGAT

GATTAGTGAAAAGGTCAGTCATGAAATAGTTATAATGAATAGTCATGGGTTTGGATTTAGTTGGGGCATATCATTAGGGA

GGGTTGGTAATCCTCTTTCTCTAGCTTAAAAGGCTAGTGCTGTTGCATAGCTTCCTAGTGGTTAAGATGATAGGAAAATT

AACATGATAAACATGATGATGGATGATGATTATTTTTTAATGTAATCTCGGTGTTGAATCTGGGTTAAAGTTAGATGATA

AAATGTGTGATGAAAATATCATGGATAGTATGGAAATTATAACTTAGAACTCCAGTGGTGTTGGTTAGAAAGTTAGAGGA

AGTGAAAAACTTGTAAAATATGTCCGGCAACCATAAATCAAAAAGCAACATAGCTTAGTAAGTATGTGAAAATACCATAA

CCAAATGCAAGACAAAGTGCATCAGTGTCAAAATGAGACTGAATTACACATGAAACCGTCTCATTCAGCAACTCTTGAAG

GAGAGAAAAGGCTCGGGAGCCACTGATGCCCAGGTCAAGAGATTGTGTGCTCCGCGTTAGCCACTGGAAGGGCCTATTGA

GATTACCCAAAAAAAAAGA-GAACCAACC-GCCTAAAAGAAGGGAACCATGCCGCGATTAAGAAAGGTATGAACTATAAA

GTACCAACCAAATGCCTTTGAAAGGAAATCCACAGGGGAGGAGTCTA

124_Campylopterus_curvipennis_INECOL-C

GGATTATGGCTACTGCTACTTCTAGGATGGTTAGTAGGAGTAGTACGCAGGCGGTTAGAGCAGAGATTGCGGGTATGATG

GAGAATAGGACTGCGGTGGCTGTTGAGATGAGTTGGATTAGTAGGTGGCCTGCTGTTAGGTTAGCTGTGAGGCGGACTCC

TAGGGCTAGAGGGCGGATCAGGAGGCTGGTAGTTTCAATTATGATTAAGGCTGGGATTAGTGGAGTTGGGGTTCCTTCGG

GTAGGAGGTGGCCAAGTGAAGCGGATGGTTGGTTTCGGAGGCCTGTGAGTAGAGTGGCTAGTCATAGTGGGAAGGCTAGT

GCTAGGTTTATAGATAATTGTGTGGTAGGTGTGAATGTGTAGGGGAGTAGGCCTAGTAGGTTGATTGAGAGAAGGAAAAT

TATTAGTGATGACAGGATAAGGGCTCATTTGTGGCCTTTTTTGTTTAATGGGATTATCAGTTGTTTGGTGATTATATAGA

TGAATCACGATTGTAGGGTGGATGTTCGGTTGGTGATTCATCGGTTGTGGGGGGAGGGGAGTAGTAGGGCTGGGAATAAT

AGTGAGATGAGAATTAGGGGGATTCCTAGTAGGTGGGGGCTTATAAATTGGTCGAAGAAGCTTAGGTTCATGGTCAGGGT

CAGGATGAGGGTTTTGTGTTTGTTAGGGGTTTTTCTAGGGGGGAGTTAGTGGGCATGAATGAAAGGAGTTTGGGTTGGAT

GATTAGTGAAAAGGTCAGTCATGAAATAGTTATAATGAATAGTCATGGGTTTGGATTTAGTTGGGGCATATCATTAGGGA

GGGTTGGTAATCCTCTTTCTCTAGCTTAAAAGGCTAGTGCTGTTGCATAGCTTCCTAGTGGTTAAGATGATAGGAAAATT

AACATGATAAACATGATGATGGATGATGATTATTTTTTAATGTAATCTCGGTGTTGAATCTGGGTTAAAGTTAGATGATA

AAATGTGTGATGAAAATATCATGGATAGTATGGAAATTATAACTTAGAACTCCAGTGGTGTTGGTTAGAAAGTTAGAGGA

AGTGAAAAACTTGTAAAATATGTCCGGCAACCATAAATCAAAAAGCAACATAGCTTAGTAAGTATGTGAAAATACCATAA

CCAAATGCAAGACAAAGTGCATCAGTGTCAAAATGAGACTGAATTACACATGAAACCGTCTCATTCAGCAACTCTTGAAG

GAGAGAAAAGGCTCGGGAGCCACTGATGCTCAGGTCAAGAGATTGTGTGCTCCGCGTTAGCCACTGGAAGGGCCTATTGA

GATTACCCAAAAAAAAAGA-GAACCAACC-GCCTAAAAGAAGGGAACCATGCCGCGATTAAGAAAGGTATGAACTATAAA

GTACCAACCAAATGCCTTTGAAAGGAAGTCCACAGGGGAGGAGTCTA

125_Campylopterus_curvipennis_INECOL-D

GGATTATGGCTACTGCTACTTCTAGGATGGTTAGTAGGAGTAGTACGCAGGCGGTTAGAGCAGAGATTGCGGGTATGATG

GAGAATAGGACTGCGGTGGCTGTTGAGATGAGTTGGATTAGTAGGTGGCCTGCTGTTAGGTTAGCTGTGAGGCGGACTCC

TAGGGCTAGAGGGCGGATCAGGAGGCTGGTAGTTTCAATTATGATTAAGGCTGGGATTAGTGGAGTTGGGGTTCCTTCGG

GTAGGAGGTGGCCAAGTGAAGCGGATGGTTGGTTTCGGAGGCCTGTGAGTAGAGTGGCTAGTCATAGTGGGAAGGCTAGT

GCTAGGTTTATAGATAATTGTGTGGTAGGTGTGAATGTGTAGGGGAGTAGGCCTAGTAGGTTGATTGAGAGAAGGAAAAT

TATTAGTGATGACAGGATAAGGGCTCATTTGTGGCCTTTTTTGTTTAATGGGATTATCAGTTGTTTGGTGATTATATAGA

TGAATCACGATTGTAGGGTGGATGTTCGGTTGGTGATTCATCGGTTGTGGGGGGAGGGGAGTAGTAGGGCTGGGAATAAT

AGTGAGATGGGAATTAGGGGGATTCCTAGTAGGTGGGGGCTTATAAATTGGTCGAAGAAGCTTAGGTTCATGGTCAGGGT

CAGGATGAGGGTTTTGTGTTTGTTAGGGGTTTTTCTAGGGGGGAGTTAGTGGGCATGAATGAAAGGAGTTTGGGTTGGAT

GATTAGTGAAAAGGTCAGTCATGAAATAGTTATAATGAATAGTCATGGGTTTGGATTTAGTTGGGGCATATCATTAGGGA

GGGTTGGTAATCCTCTTTCTCTAGCTTAAAAGGCTAGTGCTGTTGCATAGCTTCCTAGTGGTTAAGATGATAGGAAAATT

AACATGATAAACATGATGATGGATGATGATTATTTTTTAATGTAATCTCGGTGTTGAATCTGGGTTAAAGTTAGATGATA

AAATGTGTGATGAAAATATCATGGATAGTATGGAAATTATAACTTAGAACTCCAGTGGTGTTGGTTAGAAAGTTAGAGGA

AGTGAAAAACTTGTAAAATATGTCCGGCAACCATAAATCAAAAAGCAACATAGCTTAGTAAGTATGTGAAAATACCATAA

CCAAATGCAAGACAAAGTGCATCAGTGTCAAAATGAGACTGAATTACACATGAAACCGTCTCATTCAGCAACTCTTGAAG

GAGAGAAAAGGCTCGGGAGCCACTGATGCTCAGGTCAAGAGATTGTGTGCTCCGCGTTAGCCACTGGAAGGGCCTATTGA

GATTACCCAAAAAAAAAGA-GAACCAACC-GCCTAAAAGAAGGGAACCATGCCGCGATTAAGAAAGGTATGAACTATAAA

GTACCAACCAAATGCCTTTGAAAGGAAGTCCACAGGGGAGGAGTCTA

126_Campylopterus_curvipennis_CACU-156

GGATTATGGCTACTGCTACTTCTAGGATGGTTAGTAGGAGTAGTACGCAGGCGGTTAGAGCAGAGATTGCGGGTATGATG

GAGAATAGGACTGCGGTGGCTGTTGAGATGAGTTGGATTAGTAGGTGGCCTGCTGTTAGGTTAGCTGTGAGGCGGACTCC

TAGGGCTAGAGGGCGGATCAGGAGGCTGGTAGTTTCAATTATGATTAAGGCTGGGATTAGTGGAGTTGGGGTTCCTTCGG

GTAGGAGGTGGCCAAGTGAAGCGGATGGTTGGTTTCGGAGGCCTGTGAGTAGAGTGGCTAGTCATAGCGGGAAGGCTAGT

GCTAGGTTTATAGATAATTGTGTGGTAGGTGTGAATGTGTAGGGGAGTAGGCCTAGTAGGTTGATTGAGAGAAGGAAAAT

TATTAGTGATGACAGGATAAGGGCTCATTTGTGGCCTTTTTTGTTCAATGGGATTATCAGTTGTTTGGTGATTATATAGA

TGAATCACGATTGTAGGGTGGATGTTCGGTTGGTGATTCATCGGTTGTGGGGGGAGGGGAGTAGTAGGGCTGGGAATAAT

AGTGAGATGAGAATTAGGGGGATTCCTAGTAGGTGGGGGCTTATAAATTGGTCGAAGAAGCTTAGGTTCATGGTCAGGGT

CAGGATGAGGGTTTTGTGTTTGTTAGGGTTTTTTCTAGGGGGGAGTTAGTGGGCATGAATGAAAGGAGTTTGGGTTGGAT

GATTAGTGAAAAGGTCAGTCATGAAATAGTTATAATGAATAGTCATGGGTTTGGATTTAGTTGGGGCATATCATTAGGGA

GGGTTGGTAATCCTCTTTCTCTAGCTTAAAAGGCTAGTGCTGTTGCATAGCTTCCTAGTGGTTAAGATGATAGGAAAATT

AACATGATAAACATGATGATGGATGATGATTATTTTTTAATGTAATCTCGGTGTTGAATCTGGGTTAAAGTTAGATGATA

AAATGTGTGATGAAAATATCATGGATAGTATGGAAATTATAACTTAGAACTCCAGTGGTGTTGGTTAGAAAGTTAGAGGA

AGTGAAAAACTTGTAAAATATGTCCGGCAACCATAAATCAAAAAGCAACATAGCTTAGTAAGTATGTGAAAATACCATAA

CCAAATGCAAGACAAAGTGCATCAGTGTCAAAATGAGACTGAATTACACATGAAACCGTCTCATTCAGCAACTCTTGAAG

GAGAGAAAAGGCTCGGGAGCCACTGATGCCCAGGTCAAGAGATTGTGTGCTCCGCGTTAGCCACTGGAAGGGCCTATTGA

GATTACCCAAAAAAAAAGA-GAACCAACC-GCCTAAAAGAAGGGAACCATGCCGCGATTAAGAAAGGTATGAACTATAAA

GTACCAACCAAATGCCTTTGAAAGGAAATCCACAGGGGAGGAGTCTA

127_Campylopterus_curvipennis_CACU-157

GGATTATGGCTACTGCTACTTCTAGGATGGTTAGTAGGAGTAGTACGCAGGCGGTTAGAGCAGAGATTGCGGGTATGATG

GAGAATAGGACTGCGGTGGCTGTTGAGATGAGTTGGATTAGTAGGTGGCCTGCTGTTAGGTTAGCTGTGAGGCGGACTCC

TAGGGCTAGAGGGCGGATCAGGAGGCTGGTAGTTTCAATTATGATTAAGGCTGGGATTAGTGGAGTTGGGGTTCCTTCGG

GTAGGAGGTGGCCAAGTGAAGCGGATGGTTGGTTTCGGAGGCCTGTGAGTAGAGTGGCTAGTCATAGTGGGAAGGCTAGT

GCTAGGTTTATAGATAATTGTGTGGTAGGTGTGAATGTGTAGGGGAGTAGGCCTAGTAGGTTGATTGAGAGAAGGAAAAT

TATTAGTGATGACAGGATAAGGGCTCATTTGTGGCCTTTTTTGTTCAATGGGATTATCAGTTGTTTGGTGATTATATAGA

TGAATCACGATTGTAGGGTGGATGTTCGGTTGGTGATTCATCGGTTGTGGGGGGAGGGGAGTAGTAGGGCTGGGAATAAT

AGTGAGATGAGAATTAGGGGGATTCCTAGTAGGTGGGGGCTTATAAATTGGTCGAAGAAGCTTAGGTTCATGGTCAGGGT

CAGGATGAGGGTTTTGTGTTTGTTAGGGTTTTTTCTAGGGGGGAGTTAGTGGGCATGAATGAAAGGAGTTTGGGTTGGAT

GATTAGTGAAAAGGTCAGTCATGAAATAGTTATAATGAATAGTCATGGGTTTGGATTTAGTTGGGGCATATCATTAGGGA

GGGTTGGTAATCCTCTTTCTCTAGCTTAAAAGGCTAGTGCTGTTGCATAGCTTCCTAGTGGTTAAGATGATAGGAAAATT

AACATGATAAACATGATGATGGATGATGATTATTTTTTAATGTAATCTCGGTGTTGAATCTGGGTTAAAGTTAGATGATA

AAATGTGTGATGAAAATATCATGGATAGTATGGAAATTATAACTTAGAACTCCAGTGGTGTTGGTTAGAAAGTTAGAGGA

AGTGAAAAACTTGTAAAATATGTCCGGCAACCATAAATCAAAAAGCAACATAGCTTAGTAAGTATGTGAAAATACCATAA

CCAAATGCAAGACAAAGTGCATCAGTGTCAAAATGAGACTGAATTACACATGAAACCGTCTCATTCAGCAACTCTTGAAG

GAGAGAAAAGGCTCGGGAGCCACTGATGCCCAGGTCAAGAGATTGTGTGCTCCGCGTTAGCCACTGGAAGGGCCTATTGA

GATTACCCAAAAAAAAAGA-GAACCAACC-GCCTAAAAGAAGGGAACCATGCCGCGATTAAGAAAGGTATGAACTATAAA

GTACCAACCAAATGCCTTTGAAAGGAAGTCCACAGGGGAGGAGTCTA

128_Campylopterus_curvipennis_Tux-1

GGATTATGGCTACTGCTACTTCTAGGATGGTTAGTAGGAGTAGTACGCAGGCGGTTAGAGCAGAGATTGCGGGTATGATG

GAGAATAGGACTGCGGTGGCTGTTGAGATGAGTTGGATTAGTAGGTGGCCTGCTGTTAGGTTAGCTGTGAGGCGGACTCC

TAGGGCTAGAGGGCGGATCAGGAGGCTGGTAGTTTCAATTATGATTAAGGCTGGGATTAGTGGAGTTGGGGTTCCTTCGG

GTAGGAGGTGGCCAAGTGAAGCGGATGGTTGGTTTCGGAGGCCTGTAAGTAGAGTGGCTAGTCATAGTGGGAAGGCTAGT

GCTAGGTTTATAGATAATTGTGTGGTAGGTGTGAATGTGTAGGGGAGTAGGCCTAGTAGGTTGATTGAGAGAAGGAAAAT

TATTAGTGATGACAGGATAAGGGCTCATTTGTGGCCTTTTTTGTTCAATGGGATTATCAGTTGTTTGGTGATTATATAGA

TGAATCACGATTGTAGGGTGGATGTTCGGTTGGTGATTCATCGGTTGTGGGGGGAGGGGAGTAGTAGGGCTGGGAATAAT

AGTGAGATAAGAATTAGGGGGATTCCTAGTAGGTGGGGGCTTATAAATTGGTCGAAGAAGCTTAGGTTCATGGTCAGGGT

CAGGATGAGGGTTTTGTGTTTGTTAGGGGTTTTTCTAGGGGGGAGTTAGTGGGCATGAATGAAAGGAGTTTGGGTTGGAT

GATTAGTGAAAAGGTCAGTCATGAAATAGTTATAATGAATAGTCATGGGTTTGGATTTAGTTGGGGCATATCATTAGGGA

GGGTTGGTAATCCTCTTTCTCTAGCTTAAAAGGCTAGTGCTGTTGCATAGCTTCCTAGTGGTTAAGATGATAGGAAAATT

AACATGATAAACATGATGATGGATAATGATTATTTTTTAATGTAATCTCGGTGTTGAATCTGGGTTAAAGTTAGATGATA

AAATGTGTGATGAAAATATCATGGATAGTATGGAAATTATAACTTAGAACTCCAGTGGTGTTGGTTAGAAAGTTAGAGGA

AGTGAAAAACTTGTAAAATATGTCCGGCAACCATAAATCAAAAAGCAACATAGCTTAGTAGGTATGTGAAAATACCATAA

CCAAATGCAAGACAAAGTGCATCAGTGTCAAGATGAGACTGAATTACACATGAAACCGTCTCATTCAGCAACTCCTGAAG

GAGAGAAAAGGCCCGGAAGCCACTGATGCCCAGGTCAAGAGATTGTGTGCTCCGCGTTAGCCACTGGAAGGGCCTATTGA

GATTACCCAAAAAAAAAGA-GAACCAACC-GCCTAAAAGAAGGGAACCATGCCGCGATTAAGAAAGGTATGAACTATAAA

GTACCAACCAAATGCCTTTGAAAGGAAATCCACAGGGGAGGAGTCTA

129_Campylopterus_curvipennis_Tux-2

GGATTATGGCTACTGCTACTTCTAGGATGGTTAGTAGGAGTAGTACGCAGGCGGTTAGAGCAGAGATTGCGGGTATGATG

GAGAATAGGACTGCGGTGGCTGTTGAGATGAGTTGGATTAGTAGGTGGCCTGCTGTTAGGTTAGCTGTGAGGCGGACTCC

TAGGGCTAGAGGGCGGATCAGGAGGCTGGTAGTTTCAATTATGATTAAGGCTGGGATTAGTGGAGTTGGGGTTCCTTCGG

GTAGGAGGTGGCCAAGTGAAGCGGATGGTTGGTTTCGGAGGCCTGTAAGTAGAGTGGCTAGTCATAGTGGGAAGGCTAGT

GCTAGGTTTATAGATAATTGTGTGGTAGGTGTGAATGTGTAGGGGAGTAGGCCTAGTAGGTTGATTGAGAGAAGGAAAAT

TATTAGTGATGACAGGATAAGGGCTCATTTGTGGCCTTTTTTGTTCAATGGGATTATCAGTTGTTTGGTGATTATATAGA

TGAATCACGATTGTAGGGTGGATGTTCGGTTGGTGATTCATCGGTTGTGGGGGGAGGGGAGTAGTAGGGCTGGGAATAAT

AGTGAGATAAGAATTAGGGGGATTCCTAGTAGGTGGGGGCTTATAAATTGGTCGAAGAAGCTTAGGTTCATGGTCAGGGT

CAGGATGAGGGTTTTGTGTTTGTTAGGGGTTTTTCTAGGGGGGAGTTAGTGGGCATGAATGAAAGGAGTTTGGGTTGGAT

GATTAGTGAAAAGGTCAGTCATGAAATAGTTATAATGAATAGTCATGGGTTTGGATTTAGTTGGGGCATATCATTAGGGA

GGGTTGGTAATCCTCTTTCTCTAGCTTAAAAGGCTAGTGCTGTTGCATAGCTTCCTAGTGGTTAAGATGATAGGAAAATT

AACATGATAAACATGATGATGGATGATGATTATTTTTTAATGTAATCTCGGTGTTGAATCTGGGTTAAAGTTAGATGATA

AAATGTGTGATGAAAATATCATGGATAGTATGGAAATTATAACTTAGAACTCCAGTGGTGTTGGTTAGAAAGTTAGAGGA

AGTGAAAAACTTGTAAAATATGTCCGGCAACCATAAATCAAAAAGCAACATAGCTTAGTAGGTATGTGAAAATACCATAA

CCAAATGCAAGACAAAGTGCATCAGTGTCAAGATGAGACTGAATTACACATGAAACCGTCTCATTCAGCAACTCCTGAAG

GAGAGAAAAGGCCCGGAAGCCACTGATGCCCAGGTCAAGAGATTGTGTGCTCCGCGTTAGCCACTGGAAGGGCCTATTGA

GATTACCCAAAAAAAAAGA-GAACCAACC-GCCTAAAAGAAGGGAACCATGCCGCGATTAAGAAAGGTATGAACTATAAA

GTACCAACCAAATGCCTTTGAAAGGAAATCCACAGGGGAGGAGTCTA

130_Campylopterus_curvipennis_Tux-3

GGATTATGGCTACTGCTACTTCTAGGATGGTTAGTAGGAGTAGTACGCAGGCGGTTAGAGCAGAGATTGCGGGTATGATG

GAGAATAGGACTGCGGTGGCTGTTGAGATGAGTTGGATTAGTAGGTGGCCTGCTGTTAGGTTAGCTGTGAGGCGGACTCC

TAGGGCTAGAGGGCGGATCAGGAGGCTGGTAGTTTCAATTATGATTAAGGCTGGGATTAGTGGAGTTGGGGTTCCTTCGG

GTAGGAGGTGGCCAAGTGAAGCGGATGGTTGGTTTCGGAGGCCTGTAAGTAGAGTGGCTAGTCATAGTGGGAAGGCTAGT

GCTAGGTTTATAGATAATTGTGTGGTAGGTGTGAATGTGTAGGGGAGTAGGCCTAGTAGGTTGATTGAGAGAAGGAAAAT

TATTAGTGATGACAGGATAAGGGCTCATTTGTGGCCTTTTTTGTTCAATGGGATTATCAGTTGTTTGGTGATTATATAGA

TGAATCACGATTGTAGGGTGGATGTTCGGTTGGTGATTCATCGGTTGTGGGGGGAGGGGAGTAGTAGGGCTGGGAATAAT

AGTGAGATAAGAATTAGGGGGATTCCTAGTAGGTGGGGGCTTATAAATTGGTCGAAGAAGCTTAGGTTCATGGTCAGGGT

CAGGATGAGGGTTTTGTGTTTGTTAGGGGTTTTTCTAGGGGGGAGTTAGTGGGCATGAATGAAAGGAGTTTGGGTTGGAT

GATTAGTGAAAAGGTCAGTCATGAAATAGTTATAATGAATAGTCATGGGTTTGGATTTAGTTGGGGCATATCATTAGGGA

GGGTTGGTAATCCTCTTTCTCTAGCTTAAAAGGCTAGTGCTGTTGCATAGCTTCCTAGTGGTTAAGATGATAGGAAAATT

AACATGATAAACATGATGATGGATGATGATTATTTTTTAATGTAATCTCGGTGTTGAATCTGGGTTAAAGTTAGATGATA

AAATGTGTGATGAAAATATCATGGATAGTATGGAAATTATAACTTAGAACTCCAGTGGTGTTGGTTAGAAAGTTAGAGGA

AGTGAAAAACTTGTAAAATATGTCCGGCAACCATAAATCAAAAAGCAACATAGCTTAGTAGGTATGTGAAAATACCATAA

CCAAATGCAAGACAAAGTGCATCAGTGTCAAGATGAGACTGAATTACACATGAAACCGTCTCATTCAGCAACTCTTGAAG

GAGAGAAAAGGCCCGGAAGCCACTGATGCCCAGGTCAAGAGATTGTGTGCTCCGCGTTAGCCACTGGAAGGGCCTATTGA

GATTACCCAAAAAAAAAGA-GAACCAACC-GCCTAAAAGAAGGGAACCATGCCGCGATTAAGAAAGGTATGAACTATAAA

GTACCAACCAAATGCCTTTGAAAGGAAATCCACAGGGGAGGAGTCTA

131_Campylopterus_curvipennis_Tux-4

GGATTATGGCTACTGCTACTTCTAGGATGGTTAGTAGGAGTAGTACGCAGGCGGTTAGAGCAGAGATTGCGGGTATGATG

GAGAATAGGACTGCGGTGGCTGTTGAGATGAGTTGGATTAGTAGGTGGCCTGCTGTTAGGTTAGCTGTGAGGCGGACTCC

TAGGGCTAGAGGGCGGATCAGGAGGCTGGTAGTTTCAATTATGATTAAGGCTGGGATTAGTGGAGTTGGGGTTCCTTCGG

GTAGGAGGTGGCCAAGTGAAGCGGATGGTTGGTTTCGGAGGCCTGTAAGTAGAGTGGCTAGTCATAGTGGGAAGGCTAGT

GCTAGGTTTATAGATAATTGTGTGGTAGGTGTGAATGTGTAGGGGAGTAGGCCTAGTAGGTTGATTGAGAGAAGGAAAAT

TATTAGTGATGACAGGATAAGGGCTCATTTGTGGCCTTTTTTGTTCAATGGGATTATCAGTTGTTTGGTGATTATATAGA

TGAATCACGATTGTAGGGTGGATGTTCGGTTGGTGATTCATCGGTTGTGGGGGGAGGGGAGTAGTAGGGCTGGGAATAAT

AGTGAGATAAGAATTAGGGGGATTCCTAGTAGGTGGGGGCTTATAAATTGGTCGAAGAAGCTTAGGTTCATGGTCAGGGT

CAGGATGAGGGTTTTGTGTTTGTTAGGGGTTTTTCTAGGGGGGAGTTAGTGGGCATGAATGAAAGGAGTTTGGGTTGGAT

GATTAGTGAAAAGGTCAGTCATGAAATAGTTATAATGAATAGTCATGGGTTTGGATTTAGTTGGGGCATATCATTAGGGA

GGGTTGGTAATCCTCTTTCTCTAGCTTAAAAGGCTAGTGCTGTTGCATAGCTTCCTAGTGGTTAAGATGATAGGAAAATT

AACATGATAAACATGATGATGGATGATGATTATTTTTTAATGTAATCTCGGTGTTGAATCTGGGTTAAAGTTAGATGATA

AAATGTGTGATGAAAATATCATGGATAGTATGGAAATTATAACTTAGAACTCCAGTGGTGTTGGTTAGAAAGTTAGAGGA

AGTGAAAAACTTGTAAAATATGTCCGGCAACCATAAATCAAAAAGCAACATAGCTTAGTAGGTATGTGAAAATACCATAA

CCAAATGCAAGACAAAGTGCATCAGTGTCAAGATGAGACTGAATTACACATGAAACCGTCTCATTCAGCAACTCCTGAAG

GAGAGAAAAGGCCCGGAAGCCACTGATGCCCAGGTCAAGAGATTGTGTGCTCCGCGTTAGCCACTGGAAGGGCCTATTGA

GATTACCCAAAAAAAAAGA-GAACCAACC-GCCTAAAAGAAGGGAACCATGCCGCGATTAAGAAAGGTATGAACTATAAA

GTACCAACCAAATGCCTTTGAAAGGAAATCCACAGGGGAGGAGTCTA

132_Campylopterus_curvipennis_Tux-5

GGATTATGGCTACTGCTACTTCTAGGATGGTTAGTAGGAGTAGTACGCAGGCGGTTAGAGCAGAGATTGCGGGTATGATG

GAGAATAGGACTGCGGTGGCTGTTGAGATGAGTTGGATTAGTAGGTGGCCTGCTGTTAGGTTAGCTGTGAGGCGGACTCC

TAGGGCTAGAGGGCGGATCAGGAGGCTGGTAGTTTCAATTATGATTAAGGCTGGGATTAGTGGAGTTGGGGTTCCTTCGG

GTAGGAGGTGGCCAAGTGAAGCGGATGGTTGGTTACGGAGGCCTGTAAGTAGAGTGGCTAGTCATAGTGGGAAGGCTAGT

GCTAGGTTTATAGATAATTGTGTGGTAGGTGTGAATGTGTAGGGGAGTAGGCCTAGTAGGTTGATTGAGAGAAGGAAAAT

TATTAGTGATGACAGGATAAGGGCTCATTTGTGGCCTTTTTTGTTCAATGGGATTATCAGTTGTTTGGTGATTATATAGA

TGAATCACGATTGTAGGGTGGATGTTCGGTTGGTGATTCATCGGTTGTGGGGGGAGGGGAGTAGTAGGGCTGGGAATAAT

AGTGAGATAAGAATTAGGGGGATTCCTAGTAGGTGGGGGCTTATAAATTGGTCGAAGAAGCTTAGGTTCATGGTCAGGGT

CAGGATGAGGGTTTTGTGTTTGTTAGGGGTTTTTCTAGGGGGGAGTTAGTGGGCATGAATGAAAGGAGTTTGGGTTGGAT

GATTAGTGAAAAGGTCAGTCATGAAATAGTTATAATGAATAGTCATGGGTTTGGATTTAGTTGGGGCATATCATTAGGGA

GGGTTGGTAATCCTCTTTCTCTAGCTTAAAAGGCTAGTGCTGTTGCATAGCTTCCTAGTGGTTAAGATGATAGGAAAATT

AACATGATAAACATGATGATGGATGATGATTATTTTTTAATGTAATCTCGGTGTTGAATCTGGGTTAAAGTTAGATGATA

AAATGTGTGATGAAAATATCATGGATAGTATGGAAATTATAACTTAGAACTCCAGTGGTGTTGGTTAGAAAGTTAGAGGA

AGTGAAAAACTTGTAAAATATGTCCGGCAACCATAAATCAAAAAGCAACATAGCTTAGTAGGTATGTGAAAATACCATAA

CCAAATGCAAGACAAAGTGCATCAGTGTCAAGATGAGACTGAATTACACATGAAACCGTCTCATTCAGCAACTCCTGAAG

GAGAGAAAAGGCCCGGAAGCCACTGATGCCCAGGTCAAGAGATTGTGTGCTCCGCGTTAGCCACTGGAAGGGCCTATTGA

GATTACCCAAAAAAAAAGA-GAACCAACC-GCCTAAAAGAAGGGAACCATGCCGCGATTAAGAAAGGTATGAACTATAAA

GTACCAACCAAATGCCTTTGAAAGGAAATCCACAGGGGAGGAGTCTA

133_Campylopterus_curvipennis_Tux-6

GGATTATGGCTACTGCTACTTCTAGGATGGTTAGTAGGAGTAGTACGCAGGCGGTTAGAGCAGAGATTGCGGGTATGATG

GAGAATAGGACTGCGGTGGCTGTTGAGATGAGTTGGATTAGTAGGTGGCCTGCTGTTAGGTTAGCTGTGAGGCGGACTCC

TAGGGCTAGAGGGCGGATCAGGAGGCTGGTAGTTTCAATTATGATTAAGGCTGGGATTAGTGGAGTTGGGGTTCCTTCGG

GTAGGAGGTGGCCAAGTGAAGCGGATGGTTGGTTTCGGAGGCCTGTAAGTAGAGTGGCTAGTCATAGTGGGAAGGCTAGT

GCTAGGTTTATAGATAATTGTGTGGTAGGTGTGAATGTGTAGGGGAGTAGGCCTAGTAGGTTGATTGAGAGAAGGAAAAT

TATTAGTGATGACAGGATAAGGGCTCATTTGTGGCCTTTTTTGTTCAATGGGATTATCAGTTGTTTGGTGATTATATAGA

TGAATCACGATTGTAGGGTGGATGTTCGGTTGGTGATTCATCGGTTGTGGGGGGAGGGGAGTAGTAGGGCTGGGAATAAT

AGTGAGATAAGAATTAGGGGGATTCCTAGTAGGTGGGGGCTTATAAATTGGTCGAAGAAGCTTAGGTTCATGGTCAGGGT

CAGGATGAGGGTTTTGTGTTTGTTAGGGGTTTTTCTAGGGGGGAGTTAGTGGGCATGAATGAAAGGAGTTTGGGTTGGAT

GATTAGTGAAAAGGTCAGTCATGAAATAGTTATAATGAATAGTCATGGGTTTGGATTTAGTTGGGGCATATCATTAGGGA

GGGTTGGTAATCCTCTTTCTCTAGCTTAAAAGGCTAGTGCTGTTGCATAGCTTCCTAGTGGTTAAGATGATAGGAAAATT

AACATGATAAACATGATGATGGATGATGATTATTTTTTAATGTAATCTCGGTGTTGAATCTGGGTTAAAGTTAGATGATA

AAATGTGTGATGAAAATATCATGGATAGTATGGAAATTATAACTTAGAACTCCAGTGGTGTTGGTTAGAAAGTTAGAGGA

AGTGAAAAACTTGTAAAATATGTCCGGCAACCATAAATCAAAAAGCAACATAGCTTAGTAGGTATGTGAAAATACCATAA

CCAAATGCAAGACAAAGTGCATCAGTGTCAAGATGAGACTGAATTACACATGAAACCGTCTCATTCAGCAACTCCTGAAG

GAGAGAAAAGGCCCGGGAGCCACTGATGCCCAGGTCAAGAGATTGTGTGCTCCGCGTTAGCCACTGGAAGGGCCTATTGA

GATTACCCAAAAAAAAAGA-GAACCAACC-GCCTAAAAGAAGGGAACCATGCCGCGATTAAGAAAGGTATGAACTATAAA

GTACCAACCAAATGCCTTTGAAAGGAAATCTACAGGGGAGGAGTCTA

134_Campylopterus_curvipennis_Tux-7

GGATTATGGCTACTGCTACTTCTAGGATGGTTAGTAGGAGTAGTACGCAGGCGGTTAGAGCAGAGATTGCGGGTATGATG

GAGAATAGGACTGCGGTGGCTGTTGAGATGAGTTGGATTAGTAGGTGGCCTGCTGTTAGGTTAGCTGTGAGGCGGACTCC

TAGGGCTAGAGGGCGGATCAGGAGGCTGGTAGTTTCAATTATGATTAAGGCTGGGATTAGTGGAGTTGGGGTTCCTTCGG

GTAGGAGGTGGCCAAGTGAAGCGGATGGTTGGTTTCGGAGGCCTGTAAGTAGAGTGGCTAGTCATAGTGGGAAGGCTAGT

GCTAGGTTTATAGATAATTGTGTGGTAGGTGTGAATGTGTAGGGGAGTAGGCCTAGTAGGTTGATTGAGAGAAGGAAAAT

TATTAGTGATGACAGGATAAGGGCTCATTTGTGGCCTTTTTTGTTCAATGGGATTATCAGTTGTTTGGTGATTATATAGA

TGAATCACGATTGTAGGGTGGATGTTCGGTTGGTGATTCATCGGTTGTGGGGGGAGGGGAGTAGTAGGGCTGGGAATAAT

AGTGAGATAAGAATTAGGGGGATTCCTAGTAGGTGGGGGCTTATAAATTGGTCGAAGAAGCTTAGGTTCATGGTCAGGGT

CAGGATGAGGGTTTTGTGTTTGTTAGGGGTTTTTCTAGGGGGGAGTTAGTGGGCATGAATGAAAGGAGTTTGGGTTGGAT

GATTAGTGAAAAGGTCAGTCATGAAATAGTTATAATGAATAGTCATGGGTTTGGATTTAGTTGGGGCATATCATTAGGGA

GGGTTGGTAATCCTCTTTCTCTAGCTTAAAAGGCTAGTGCTGTTGCATAGCTTCCTAGTGGTTAAGATGATAGGAAAATT

AACATGATAAACATGATGATGGATGATGATTATTTTTTAATGTAATCTCGGTGTTGAATCTGGGTTAAAGTTAGATGATA

AAATGTGTGATGAAAATATCATGGATAGTATGGAAATTATAACTTAGAACTCCAGTGGTGTTGGTTAGAAAGTTAGAGGA

AGTGAAAAACTTGTAAAATATGTCCGGCAACCATAAATCAAAAAGCAACATAGCTTAGTAGGTATGTGAAAATACCATAA

CCAAATGCAAGACAAAGTGCATCAGTGTCAAGATGAGACTGAATTACACATGAAACCGTCTCATTCAGCAACTCCTGAAG

GAGAGAAAAGGCCCGGAAGCCACTGATGCCCAGGTCAAGAGATTGTGTGCTCCGCGTTAGCCACTGGAAGGGCCTATTGA

GATTACCCAAAAAAAAAGA-GAACCAACC-GCCTAAAAGAAGGGAACCATGCCGCGATTAAGAAAGGTATGAACTATAAA

GTACCAACCAAATGCCTTTGAAAGGAAATCCACAGGGGAGGAGTCTA

135_Campylopterus_curvipennis_Tux-8

GGATTATGGCTACTGCTACTTCTAGGATGGTTAGTAGGAGTAGTACGCAGGCGGTTAGAGCAGAGATTGCGGGTATGATG

GAGAATAGGACTGCGGTGGCTGTTGAGATGAGTTGGATTAGTAGGTGGCCTGCTGTTAGGTTAGCTGTGAGGCGGACTCC

TAGGGCTAGAGGGCGGATCAGGAGGCTGGTAGTTTCAATTATGATTAAGGCTGGGATTAGTGGAGTTGGGGTTCCTTCGG

GTAGGAGGTGGCCAAGTGAAGCGGATGGTTGGTTTCGGAGGCCTGTAAGTAGAGTGGCTAGTCATAGTGGGAAGGCTAGT

GCTAGGTTTATAGATAATTGTGTGGTAGGTGTGAATGTGTAGGGGAGTAGGCCTAGTAGGTTGATTGAGAGAAGGAAAAT

TATTAGTGATGACAGGATAAGGGCTCATTTGTGGCCTTTTTTGTTCAATGGGATTATCAGTTGTTTGGTGATTATATAGA

TGAATCACGATTGTAGGGTGGATGTTCGGTTGGTGATTCATCGGTTGTGGGGGGAGGGGAGTAGTAGGGCTGGGAATAAT

AGTGAGATAAGAATTAGGGGGATTCCTAGTAGGTGGGGGCTTATAAATTGGTCGAAGAAGCTTAGGTTCATGGTCAGGGT

CAGGATGAGGGTTTTGTGTTTGTTAGGGGTTTTTCTAGGGGGGAGTTAGTGGGCATGAATGAAAGGAGTTTGGGTTGGAT

GATTAGTGAAAAGGTCAGTCATGAAATAGTTATAATGAATAGTCATGGGTTTGGATTTAGTTGGGGCATATCATTAGGGA

GGGTTGGTAATCCTCTTTCTCTAGCTTAAAAGGCTAGTGCTGTTGCATAGCTTCCTAGTGGTTAAGATGATAGGAAAATT

AACATGATAAACATGATGATGGATGATGATTATTTTTTAATGTAATCTCGGTGTTGAATCTGGGTTAAAGTTAGATGATA

AAATGTGTGATGAAAATATCATGGATAGTATGGAAATTATAACTTAGAACTCCAGTGGTGTTGGTTAGAAAGTTAGAGGA

AGTGAAAAACTTGTAAAATATGTCCGGCAACCATAAATCAAAAAGCAACATAGCTTAGTAGGTATGTGAAAATACCATAA

CCAAATGCAAGACAAAGTGCATCAGTGTCAAGATGAGACTGAATTACACATGAAACCGTCTCATTCAGCAACTCCTGAAG

GAGAGAAAAGGCCCGGAAGCCACTGATGCCCAGGTCAAGAGATTGTGTGCTCCGCGTTAGCCACTGGAAGGGCCTATTGA

GATTACCCAAAAAAAAAGA-GAACCAACC-GCCTAAAAGAAGGGAACCATGCCGCGATTAAGAAAGGTATGAACTATAAA

GTACCAACCAAATGCCTTTGAAAGGAAATCCACAGGGGAGGAGTCTA

136_Campylopterus_curvipennis_Tux-9

GGATTATGGCTACTGCTACTTCTAGGATGGTTAGTAGGAGTAGTACGCAGGCGGTTAGAGCAGAGATTGCGGGTATGATG

GAGAATAGGACTGCGGTGGCTGTTGAGATGAGTTGGATTAGTAGGTGGCCTGCTGTTAGGTTAGCTGTGAGGCGGACTCC

TAGGGCTAGAGGGCGGATCAGGAGGCTGGTAGTTTCAATTATGATTAAGGCTGGGATTAGTGGAGTTGGGGTTCCTTCGG

GTAGGAGGTGGCCAAGTGAAGCGGATGGTTGGTTTCGGAGGCCTGTAAGTAGAGTGGCTAGTCATAGTGGGAAGGCTAGT

GCTAGGTTTATAGATAATTGTGTGGTAGGTGTGAATGTGTAGGGGAGTAGGCCTAGTAGGTTGATTGAGAGAAGGAAAAT

TATTAGTGATGACAGGATAAGGGCTCATTTGTGGCCTTTTTTGTTCAATGGGATTATCAGTTGTTTGGTGATTATATAGA

TGAATCACGATTGTAGGGTGGATGTTCGGTTGGTGATTCATCGGTTGTGGGGGGAGGGGAGTAGTAGGGCTGGGAATAAT

AGTGAGATAAGAATTAGGGGGATTCCTAGTAGGTGGGGGCTTATAAATTGGTCGAAGAAGCTTAGGTTCATGGTCAGGGT

CAGGATGAGGGTTTTGTGTTTGTTAGGGGTTTTTCTAGGGGGGAGTTAGTGGGCATGAATGAAAGGAGTTTGGGTTGGAT

GATTAGTGAAAAGGTCAGTCATGAAATAGTTATAATGAATAGTCATGGGTTTGGATTTAGTTGGGGCATATCATTAGGGA

GGGTTGGTAATCCTCTTTCTCTAGCTTAAAAGGCTAGTGCTGTTGCATAGCTTCCTAGTGGTTAAGATGATAGGAAAATT

AACATGATAAACATGATGATGGATGATGATTATTTTTTAATGTAATCTCGGTGTTGAATCTGGGTTAAAGTTAGATGATA

AAATGTGTGATGAAAATATCATGGATAGTATGGAAATTATAACTTAGAACTCCAGTGGTGTTGGTTAGAAAGTTAGAGGA

AGTGAAAAACTTGTAAAATATGTCCGGCAACCATAAATCAAAAAGCAACATAGCTTAGTAGGTATGTGAAAATACCATAA

CCAAATGCAAGACAAAGTGCATCAGTGTCAAGATGAGACTGAATTACACATGAAACCGTCTCATTCAGCAACTCCTGAAG

GAGAGAAAAGGCCCGGAAGCCACTGATGCCCAGGTCAAGAGATTGTGTGCTCCGCGTTAGCCACTGGAAGGGCCTATTGA

GATTACCCAAAAAAAAAGA-GAACCAACC-GCCTAAAAGAAGGGAACCATGCCGCGATTAAGAAAGGTATGAACTATAAA

GTACCAACCAAATGCCTTTGAAAGGAAATCCACAGGGGAGGAGTCTA

137_Campylopterus_curvipennis_Tux-10

GGATTATGGCTACTGCTACTTCTAGGATGGTTAGTAGGAGTAGTACGCAGGCGGTTAGAGCAGAGATTGCGGGTATGATG

GAGAATAGGACTGCGGTGGCTGTTGAGATGAGTTGGATTAGTAGGTGGCCTGCTGTTAGGTTAGCTGTGAGGCGGACTCC

TAGGGCTAGAGGGCGGATCAGGAGGCTGGTAGTTTCAATTATGATTAAGGCTGGGATTAGTGGAGTTGGGGTTCCTTCGG

GTAGGAGGTGGCCAAGTGAAGCGGATGGTTGGTTTCGGAGGCCTGTAAGTAGAGTGGCTAGTCATAGTGGGAAGGCTAGT

GCTAGGTTTATAGATAATTGTGTGGTAGGTGTGAATGTGTAGGGGAGTAGGCCTAGTAGGTTGATTGAGAGAAGGAAAAT

TATTAGTGATGACAGGATAAGGGCTCATTTGTGGCCTTTTTTGTTCAATGGGATTATCAGTTGTTTGGTGATTATATAGA

TGAATCACGATTGTAGGGTGGATGTTCGGTTGGTGATTCATCGGTTGTGGGGGGAGGGGAGTAGTAGGGCTGGGAATAAT

AGTGAGATAAGAATTAGGGGGATTCCTAGTAGGTGGGGGCTTATAAATTGGTCGAAGAAGCTTAGGTTCATGGTCAGGGT

CAGGATGAGGGTTTTGTGTTTGTTAGGGGTTTTTCTAGGGGGGAGTTAGTGGGCATGAATGAAAGGAGTTTGGGTTGGAT

GATTAGTGAAAAGGTCAGTCATGAAATAGTTATAATGAATAGTCATGGGTTTGGATTTAGTTGGGGCATATCATTAGGGA

GGGTTGGTAATCCTCTTTCTCTAGCTTAAAAGGCTAGTGCTGTTGCATAGCTTCCTAGTGGTTAAGATGATAGGAAAATT

AACATGATAAACATGATGATGGATGATGATTATTTTTTAATGTAATCTCGGTGTTGAATCTGGGTTAAAGTTAGATGATA

AAATGTGTGATGAAAATATCATGGATAGTATGGAAATTATAACTTAGAACTCCAGTGGTGTTGGTTAGAAAGTTAGAGGA

AGTGAAAAACTTGTAAAATATGTCCGGCAACCATAAATCAAAAAGCAACATAGCTTAGTAGGTATGTGAAAATACCATAA

CCAAATGCAAGACAAAGTGCATCAGTGTCAAGATGAGACTGAATTACACATGAAACCGTCTCATTCAGCAACTCCTGAAG

GAGAGAAAAGGCCCGGAAGCCACTGATGCCCAGGTCAAGAGATTGTGTGCTCCGCGTTAGCCACTGGAAGGGCCTATTGA

GATTACCCAAAAAAAAAGA-GAACCAACC-GCCTAAAAGAAGGGAACCATGCCGCGATTAAGAAAGGTATGAACTATAAA

GTACCAACCAAATGCCTTTGAAAGGAAATCCACAGGGGAGGAGTCTA

138_Campylopterus_curvipennis_Nopal-1

GGATTATGGCTACTGCTACTTCTAGGATGGTTAGTAGGAGTAGTACGCAGGCGGTTAGAGCAGAGATTGCGGGTATGATG

GAGAATAGGACTGCGGTGGCTGTTGAGATGAGTTGGATTAGTAGGTGGCCTGCTGTTAGGTTAGCTGTGAGGCGGACTCC

TAGGGCTAGAGGGCGGATCAGGAGGCTGGTAGTTTCAATTATGATTAAGGCTGGGATTAGTGGAGTTGGGGTTCCTTCGG

GTAGGAGGTGGCCAAGTGAAGCGGATGGTTGGTTTCGGAGGCCTGTGAGTAGAGTGGCTAGTCATAGTGGGAAGGCTAGT

GCTAGGTTTATAGATAATTGTGTGGTAGGTGTGAATGTGTAGGGGAGTAGGCCTAGTAGGTTGATTGAGAGAAGGAAAAT

TATTAGTGATGACAGGATAAGGGCTCATTTGTGGCCTTTTTTGTTCAATGGGATTATCAGTTGTTTGGTGATTATATAGA

TGAATCACGATTGTAGGGTGGATGTTCGGTTGGTGATTCATCGGTTGTGGGGGGAGGGGAGTAGTAGGGCTGGGAATAAT

AGTGAGATAAGAATTAGGGGGATTCCTAGTAGGTGGGGGCTTATAAATTGGTCGAAGAAGCTTAGGTTCATGGTCAGGGT

CAGGATGAGGGTTTTGTGTTTGTTAGGGGTTTTTCTAGGGGGGAGTTAGTGGGCATGAATGAAAGGAGTTTGGGTTGGAT

GATTAGTGAAAAGGTCAGTCATGAAATAGTTATAATGAATAGTCATGGGTTTGGATTTAGTTGGGGCATATCATTAGGGA

GGGTTGGTAATCCTCTTTCTCTAGCTTAAAAGGCTAGTGCTGTTGCATAGCTTCCTAGTGGTTAAGATGATAGGAAAATT

AACATGATAAACATGATGATGGATGATGATTATTTTTTAATGTAATCTCGGTGTTGAATCTGGGTTAAAGTTAGATGATA

AAATGTGTGATGAAAATATCATGGATAGTATGGAAATTATAACTTAGAACTCCAGTGGTGTTGGTTAGAAAGTTAGAGGA

AGTGAAAAACTTGTAAAATATGTCCGGCAACCATAAATCAAAAAGCAACATAGCTTAGTAGGTATGTGAAAATACCATAA

CCAAATGCAAGACAAAGTGCATCAGTGTCAAGATGAGACTGAATTACACATGAAACCGTCTCATTCAGCAACTCCTGAAG

GAGAGAAAAGGCCCGGAAGCCACTGATGCCCAGGTCAAGAGATTGTGTGCTCCGCGTTAGCCACTGGAAGGGCCTATTGA

GATTACCCAAAAAAAAAGA-GAACCAACC-GCCTAAAAGAAGGGAACCATGCCGCGATTAAGAAAGGTATGAACTATAAA

GTACCAACCAAATGCCTTTGAAAGGAAATCCACAGGGGAGGAGTCTA

139_Campylopterus_curvipennis_OAX

GGATTATGGCTACTGCTACTTCTAGGATGGTTAGTAGGAGTAGTACGCAGGCGGTTAGAGCAGAGATTGCGGGTATGATG

GAGAATAGGACTGTGGTGGCTGTTGAGATGAGTTGGATTAGTAGGTGGCCTGCTGTTAGGTTAGCTGTGAGGCGGACTCC

TAGGGCTAGAGGGCGGATCAGGAGGCTGGTAGTTTCAATTATGATTAAGGCTGGGATTAGTGGAGTTGGGGTTCCTTCGG

GTAGGAGGTGGCCAAGTGAAGCGGATGGTTGGTTTCGGAGGCCTGTGAGTAGAGTGGCTAGTCATAGTGGGAAGGCTAGT

GCTAGGTTTATAGATAATTGTGTGGTAGGTGTGAATGTGTAGGGGAGTAGGCCTAGTAGGTTGATTGAGAGAAGGAAAAT

TATTAGTGATGACAGGATAAGGGCTCATTTGTGGCCTTTTTTGTTCAATGGGATTATCAGTTGTTTGGTGATTATATAGA

TGAATCACGATTGTAGGGTGGATGTTCGGTTGGTGATTCATCGGTTGTGGGGGGAGGGGAGTAGTAGGGCTGGGAATAAT

AGTGAGATAAGAATTAGGGGGATTCCTAGTAGGTGGGGGCTTATAAATTGGTCGAAGAAGCTTAGGTTCATGGTCAGGGT

CAGGATGAGGGTTTTGTGTTTGTTAGGGGTTTTTCTAGGGGGGAGTTAGTGGGCATGAATGAAAGGAGTTTGGGTTGGAT

GATTAGTGAAAAGGTCAGTCATGAAATAGTTATAATGAATAGTCATGGGTTTGGATTTAGTTGGGGCATATCATTAGGGT

GGGTTGGTAATCCTCTTTCTCTAGCTTAAAAGGCTAGTGCTGTTGCATAGCTTCCTAGTGGTTAAGATGATAGGAAAATT

AACATGATAAACATGATGATGGATGATGATTATTTTTTAATGTAATCTCGGTGTTGAATCTGGGTTAAAGTTAGATGATA

AAATGTGTGATGAAAATATCATGGATAGTATGGAAATTATAACTTAGAACTCCAGTGGTGTTGGTTAGAAAGTTAGAGGA

AGTGAAAAACTTGTAAAATATGTCCGGCAACCATAAATCAAAAAGCAACATAGCTTAGTAGGTATGTGAAAATACCATAA

CCAAATGCAAGACAAAGTGCATCAGTGTCAAGATGAGACTGAATTACACATGAAACCGTCTCATTCAGCAACTCCTGAAG

GAGAGAAAAGGCCCGGAAGCCACTGATGCCCAGGTCAAGAGATTGTGTGCTCCGCGTTAGCCACTGGAAGGGCCTATTGA

GATTACCCAAAAAAAAAGA-GAACCAACC-GCCTAAAAGAAGGGAACCATGCCGCGATTAAGAAAGGTATGAACTATAAA

GTACCAACCAAATGCCTTTGAAAGGAAATCCACAGGGGAGGAGTCTA

140_Campylopterus_curvipennis_Esc-1

GGATTATGGCTACTGCTACTTCTAGGATGGTTAGTAGGAGTAGTACGCAGGCGGTTAGTGCAGAGATTGCGGGTATGATG

GAGAATAGGACTGCGGTGGCTGTTGAGATGAGTTGGATTAGTAGGTGTCCTGCTGTTAGGTTGGCTGTGAGGCGGACTCC

TAGGGCTAGAGGGCGGATCAGAAGGCTGGTAGTTTCAATTATGATTAAGGCCGGAATTAGTGGAGTTGGGGTTCCTTCGG

GTAGGAGGTGGCCAAGTGAAGCGGATGGTTGGTTTCGGAGGCCTGTGAGTAGAGTGGCTAGTCATAGTGGGAAGGCTAGT

GCTAGGTTTATAGATAATTGTGTGGTAGGTGTGAATGTGTAGGGGAGTAGGCCTAGTAGGTTGATTGAGAGAAGGAAAAT

TATCAGTGATGACAGGATAAGGGCTCATTTGTGGCCTTTTTTGTTCAATGGGATTATCAGTTGTTTGGTGATTATATAAA

TGAATCACGATTGTAGGGTGGATGTTCGGTTAGTGATTCATCGGTTGTGGGGGGAGGGGAGTAGTAGAGCTGGGAATAAT

AGTGAGATGAGGATTAGGGGGATTCCTAGTAGGTGGGGGCTTATAAATTGGTCGAAGAAGCTTAGGTTCATGGTCAGGGT

CAGGATGGGGGTTTTGTGCCTGTTAGGGGTTTTTCTAGGGGGGAGTTGGTGGGCGTGAATGAAAGGAGTTTGGGTTGGAT

GATTAGTGAAAAGGTCAGTCATGAAATAGTTATAACGAATAGTCATGGGTTTGGATTTAGTTGGGGCATATCATTAGGGA

GGGTTGGTAATCCTCTTTCTCTAGCTTAAAAGGCTAGTGCTGTTGCATAGCTTCCTAGTGGTTAAGATGATAGGAAAATT

AACATGATAAACATGATGATGGATGGTGATTATTTTTTAATGTAATCTCGGTGTTGAATATAGGTTAAAGTTAGATGATA

AAATGTGTGATAAAAATATCATGGATAATATGGAAATTATAACTTAGAACTCCAGTGGTGTTGGTTAGAAAGTTAGAGGA

AGTGAAAAACTTGTAAAATATGTCCGGCAACCATAAATCAAAAAGCAACATAGCTTAGTAAGTATGTGAAAATTCCATAA

CCAAATGCAAGAAAAAGTGCATCAGTGTCAAAGTGAGACTGAATTACACATGAAACCGTCTCATTCAGCAACTCTTGAAG

GAGAGAAAGAGCTCGAAAGCCACTGATGCTCAGGTCAAGAGATTGTGTGCTCCGCGCTAGCCACCGG-GGGGCCTATTGA

AATTACCCAAAAAAAAAAGAGAACCAACC-GCCTAAAAGAAGGGAACCATGCCGCGATTAAGAAAGGTATGAACTATAAA

GTACCAACCAAATGCCTTTGAAAGGAAGTCCACAGGGGAGAAGTCTG

141_Campylopterus_curvipennis_Bec-1

GGATTATGGCTACTGCTACTTCTAGGATGGTTAGTAGGAGTAGTACGCAGGCGGTTAGTGCAGAGATTGCGGGTATGATG

GAGAATAGGACTGCGGTGGCTGTTGAGATGAGTTGGATTAGTAGGTGTCCTGCTGTTAGGTTGGCTGTGAGGCGGACTCC

TAGGGCTAGAGGGCGGATCAGAAGGCTGGTAGTTTCAATTATGATTAAGGCCGGAATTAGTGGAGTTGGGGTTCCTTCGG

GTAGGAGGTGGCCAAGTGAAGCGGATGGTTGGTTTCGGAGGCCTGTGAGTAGAGTGGCTAGTCATAGTGGGAAGGCTAGT

GCTAGGTTTATAGATAATTGTGTGGTAGGTGTGAATGTGTAGGGGAGTAGGCCTAGTAGGTTGATTGAGAGAAGGAAAAT

TATCAGTGATGACAGGATAAGGGCTCATTTGTGGCCTTTTTTGTTCAATGGGATTATCAGTTGTTTGGTGATTATATAAA

TGAATCACGATTGTAGGGTGGATGTTCGGTTAGTGATTCATCGGTTGTGGGGGGAGGGGAGTAGTAGAGCTGGGAATAAT

AGTGAGATGAGGATTAGGGGGATTCCTAGTAGGTGGGGGCTTATAAATTGGTCGAAGAAGCTTAGGTTCATGGTCAGGGT

CAGGATGGGGGTTTTGTGTCTGTTAGGGGTTTTTCTAGGGGGGAGTTGGTGGGCGTGAATGAAAGGAGTTTGGGTTGGAT

GATTAGTGAAAAGGTCAGTCATGAAATAGTTATAACGAATAGTCATGGGTTTGGATTTAGTTGGGGCATATCATTAGGGA

GGGTTGGTAATCCTCTTTCTCTAGCTTAAAAGGCTAGTGCTGTTGCATAGCTTCCTAGTGGTTAAGATGATAGGAAAATT

AACATGATAAACATGATGATGGATGGTGATTATTTTTTAATGTAATCTCGGTGTTGAATATAGGTTAAAGTTAGATGATA

AAATGTGTGATAAAAATATCATGGATAATATGGAAATTATAACTTAGAACTCCAGTGGTGTTGGTTAGAAAGTTAGAGGA

AGTGAAAAACTTGTAAAATATGTCCGGCAACCATAAATCAAAAAGCAACATAGCTTAGTAAGTATGTGAAAATTCCATAA

CCAAATGCAAGAAAAAGTGCATCAGTGTCAAAGTGAGACTGAATTACACATGAAACCGTCTCATTCAGCAACTCTTGAAG

GAGAGAAAGAGCTCGAAAGCCACTGATGCTCAGGTCAAGAGATTGTGTGCTCCGCGCTAGCCACCGG-GGGGCCTATTGA

AATTACCCAAAAAAAAAAGAGAACCAACC-GCCTAAAAGAAGGGAACCATGCCGCGATTAAGAAAGGTATGAACTATAAA

GTACCAACCAAATGCCTTTGAAAGGAAGTCCACAGGGGAGAAGTCTG

142_Campylopterus_curvipennis_Bec-2

GGATTATGGCTACTGCTACTTCTAGGATGGTTAGTAGGAGTAGTACGCAGGCGGTTAGTGCAGAGATTGCGGGTATGATG

GAGAATAGAACTGCGGTGGCTGTTGAGATGAGTTGGATTAGTAGGTGTCCTGCTGTTAGGTTGGCTGTGAGGCGGACTCC

TAGGGCTAGAGGGCGGATCAGAAGGCTGGTAGTTTCAATTATGATTAAGGCCGGAATTAGTGGAGTTGGGGTTCCTTCGG

GTAGGAGGTGGCCAAGTGAAGCGGATGGTTGGTTTCGGAGGCCTGTGAGTAGAGTGGCTAGTCATAGTGGGAAGGCTAGT

GCTAGGTTTATAGATAATTGTGTGGTAGGTGTGAATGTGTAGGGGAGTAGGCCTAGTAGGTTGATTGAGAGAAGGAAAAT

TATCAGTGATGACAGGATAAGGGCTCATTTGTGGCCTTTTTTGTTCAATGGGATTATCAGTTGTTTGGTGATTATATAAA

TGAATCACGATTGTAGGGTGGATGTTCGGTTAGTGATTCATCGGTTGTGGGGGGAGGGGAGTAGTAGAGCTGGGAATAAT

AGTGAGATGAGGATTAGGGGGATTCCTAGTAGGTGGGGGCTTATAAATTGGTCGAAGAAGCTTAGGTTCATGGTCAGGGT

CAGGATGGGGGTTTTGTGCCTGTTAGGGGTTTTTCTAGGGGGGAGTTGGTGGGCGTGAATGAAAGGAGTTTGGGTTGGAT

GATTAGTGAAAAGGTCAGTCATGAAATAGTTATAACGAATAGTCATGGGTTTGGATTTAGTTGGGGCATATCATTAGGGA

GGGTTGGTAATCCTCTTTCTCTAGCTTAAAAGGCTAGTGCTGTTGCATAGCTTCCTAGTGGTTAAGATGATAGGAAAATT

AACATGATAAACATGATGATGGATGGTGATTATTTTTTAATGTAATCTCGGTGTTGAATATAGGTTAAAGTTAGATGATA

AAATGTGTGATAAAAATATCATGGATAATATGGAAATTATAACTTAGAACTCCAGTGGTGTTGGTTAGAAAGTTAGAGGA

AGTGAAAAACTTGTAAAATATGTCCGGCAACCATAAATCAAAAAGCAACATAGCTTAGTAAGTATGTGAAAATTCCATAA

CCAAATGCAAGAAAAAGTGCATCAGTGTCAAAGTGAGACTGAATTACACATGAAACCGTCTCATTCAGCAACTCTTGAAG

GAGAGAAAGAGCTCGAAAGCCACTGATGCTCAGGTCAAGAGATTGTGTGCTCCGCGCTAGCCACCGG-GGGGCCTATTGA

AATTACCCAAAAAAAAAAGAGAACCAACC-GCCTAAAAGAAGGGAACCATGCCGCGATTAAGAAAGGTATGAACTATAAA

GTACCAACCAAATGCCTTTGAAAGGAAGTCCACAGGGGAGAAGTCTG

143_Campylopterus_curvipennis_20Nov-1

GGATTATGGCTACTGCTACTTCTAGGATGGTTAGTAGGAGTAGTACGCAGGCGGTTAGTGCAGAGATTGCGGGTATGATG

GAGAATAGAACTGCGGTGGCTGTTGAGATGAGTTGGATTAGTAGGTGTCCTGCTGTTAGGTTGGCTGTGAGGCGGACTCC

TAGGGCTAGAGGGCGGATCAGAAGGCTGGTAGTTTCAATTATGATTAAGGCCGGAATTAGTGGAGTTGGGGTTCCTTCGG

GTAGGAGGTGGCCAAGTGAAGCGGATGGTTGGTTTCGGAGGCCTGTGAGTAGAGTGGCTAGTCATAGTGGGAAGGCTAGT

GCTAGGTTTATAGATAATTGTGTGGTAGGTGTGAATGTGTAGGGGAGTAGGCCTAGTAGGTTGATTGAGAGAAGGAAAAT

TATCAGTGATGACAGGATAAGGGCTCATTTGTGGCCTTTTTTGTTCAATGGGATTATCAGTTGTTTGGTGATTATATAAA

TGAATCACGATTGTAGGGTGGATGTTCGGTTAGTGATTCATCGGTTGTGGGGGGAGGGGAGTAGTAGAGCTGGGAATAAT

AGTGAGATGAGGATTAGGGGGATTCCTAGTAGGTGGGGGCTTATAAATTGGTCGAAGAAGCTTAGGTTCATGGTCAGGGT

CAGGATGGGGGTTTTGTGTCTGTTAGGGGTTTTTCTAGGGGGGAGTTGGTGGGCGTGAATGAAAGGAGTTTGGGTTGGAT

GATTAGTGAAAAGGTCAGTCATGAAATAGTTATAACGAATAGTCATGGGTTTGGATTTAGTTGGGGCATATCATTAGGGA

GGGTTGGTAATCCTCTTTCTCTAGCTTAAAAGGCTAGTGCTGTTGCATAGCTTCCTAGTGGTTAAGATGATAGGAAAATT

AACATGATAAACATGATGATGGATGGTGATTATTTTTTAATGTAATCTCGGTGTTGAATATAGGTTAAAGTTAGATGATA

AAATGTGTGATAAAAATATCATGGATAATATGGAAATTATAACTTAGAACTCCAGTGGTGTTGGTTAGAAAGTTAGAGGA

AGTGAAAAACTTGTAAAATATGTCCGGCAACCATAAATCAAAAAGCAACATAGCTTAGTAAGTATGTGAAAATTCCATAA

CCAAATGCAAGAAAAAGTGCATCAGTGTCAAAGTGAGACTGAATTACACATGAAACCGTCTCATTCAGCAACTCTTGAAG

GAGAGAAAGAGCTCGAAAGCCACTGATGCCCAGGTCAAGAGATTGTGTGCTCCGCGCTAGCCACCGG-GGGGCCTATTGA

AATTACCCCAAAAAAAAAGAGAACCAACC-GCCAAAAAGAAGGGAACCATGCCGCGATTAAGAAAGGTATGAACTATAAA

GTACCAACCAAATGCCTTTGAAAGGAAGTCCACAGGGGAGAAGTCTG

144_Campylopterus_curvipennis_20Nov-2

GGATTATGGCTACTGCTACTTCTAGGATGGTTAGTAGGAGTAGTACGCAGGCGGTTAGTGCAGAGATTGCGGGTATGATG

GAGAATAGGACTGCGGTGGCTGTTGAGATGAGTTGGATTAGTAGGTGTCCTGCTGTTAGGTTGGCTGTGAGGCGGACTCC

TAGGGCTAGAGGGCGGATCAGAAGGCTGGTAGTTTCAATTATGATTAAGGCCGGAATTAGTGGAGTTGGGGTTCCTTCGG

GTAGGAGGTGGCCAAGTGAAGCGGATGGTTGGTTTCGGAGGCCTGTGAGTAGAGTGGCTAGTCATAGTGGGAAGGCTAGT

GCTAGGTTTATAGATAATTGTGTGGTAGGTGTGAATGTGTAGGGGAGTAGGCCTAGTAGGTTGATTGAGAGAAGGAAAAT

TATCAGTGATGACAGGATAAGGGCTCATTTGTGGCCTTTTTTGTTCAATGGGATTATCAGTTGTTTGGTGATTATATAAA

TGAATCACGATTGTAGGGTGGATGTTCGGTTAGTGATTCATCGGTTGTGGGGGGAGGGGAGTAGTAGAGCTGGGAATAAT

AGTGAGATGAGGATTAGGGGGATTCCTAGTAGGTGGGGGCTTATAAATTGGTCGAAGAAGCTTAGGTTCATGGTCAGGGT

CAGGATGGGGGTTTTGTGTCTGTTAGGGGTTTTTCTAGGGGGGAGTTGGTGGGCGTGAATGAAAGGAGTTTGGGTTGGAT

GATTAGTGAAAAGGTCAGTCATGAAATAGTTATAACGAATAGTCATGGGTTTGGATTTAGTTGGGGCATATCATTAGGGA

GGGTTGGTAATCCTCTTTCTCTAGCTTAAAAGGCTAGTGCTGTTGCATAGCTTCCTAGTGGTTAAGATGATAGGAAAATT

AACATGATAAACATGATGATGGATGGTGATTATTTTTTAATGTAATCTCGGTGTTGAATATAGGTTAAAGTTAGATGATA

AAATGTGTGATAAAAATATCATGGATAATATGGAAATTATAACTTAGAACTCCAGTGGTGTTGGTTAGAAAGTTAGAGGA

AGTGAAAAACTTGTAAAATATGTCCGGCAACCATAAATCAAAAAGCAACATAGCTTAGTAAGTATGTGAAAATTCCATAA

CCAAATGCAAGAAAAAGTGCATCAGTGTCAAAGTGAGACTGAATTACACATGAAACCGTCTCATTCAGCAACTCTTGAAG

GAGAGAAAGAGCTCGAAAGCCACTGATGCTCAGGTCAAGAGATTGTGTGCTCCGCGCTAGCCACCGG-GGGGCCTATTGA

AATTACCCCAAAAAAAAAGAGAACCAACC-GCCTAAAAGAAGGGAACCATGCCGCGATTAAGAAAGGTATGAACTATAAA

GTACCAACCAAATGCCTTTGAAAGGAAGTCCACAGGGGAGAAGTCTG

145_Campylopterus_curvipennis_20Nov-3

GGATTATGGCTACTGCTACTTCTAGGATGGTTAGTAGGAGTAGTACGCAGGCGGTTAGTGCAGAGATTGCGGGTATGATG

GAGAATAGGACTGCGGTGGCTGTTGAGATGAGTTGGATTAGTAGGTGTCCTGCTGTTAGGTTGGCTGTGAGGCGGACTCC

TAGGGCTAGAGGGCGGATCAGAAGGCTGGTAGTTTCAATTATGATTAAGGCCGGAATTAGTGGAGTTGGGGTTCCTTCGG

GTAGGAGGTGGCCAAGTGAAGCGGATGGTTGGTTTCGGAGGCCTGTGAGTAGAGTGGCTAGTCATAGTGGGAAGGCTAGT

GCTAGGTTTATAGATAATTGTGTGGTAGGTGTGAATGTGTAGGGGAGTAGGCCTAGTAGGTTGATTGAGAGAAGGAAAAT

TATCAGTGATGACAGGATAAGGGCTCATTTGTGGCCTTTTTTGTTCAATGGGATTATCAGTTGTTTGGTGATTATATAAA

TGAATCACGATTGTAGGGTGGATGTTCGGTTAGTGATTCATCGGTTGTGGGGGGAGGGGAGTAGTAGAGCTGGGAATAAT

AGTGAGATGAGGATTAGGGGGATTCCTAGTAGGTGGGGGCTTATAAATTGGTCGAAGAAGCTTAGGTTCATGGTCAGGGT

CAGGATGGGGGTTTTGTGTCTGTTAGGGGTTTTTCTAGGGGGGAGTTGGTGGGCGTGAATGAAAGGAGTTTGGGTTGGAT

GATTAGTGAAAAGGTCAGTCATGAAATAGTTATAACGAATAGTCATGGGTTTGGATTTAGTTGGGGCATATCATTAGGGA

GGGTTGGTAATCCTCTTTCTCTAGCTTAAAAGGCTAGTGCTGTTGCATAGCTTCCTAGTGGTTAAGATGATAGGAAAATT

AACATGATAAACATGATGATGGATGGTGATTATTTTTTAATGTAATCTCGGTGTTGAATATAGGTTAAAGTTAGATGATA

AAATGTGTGATAAAAATATCATGGATAATATGGAAATTATAACTTAGAACTCCAGTGGTGTTGGTTAGAAAGTTAGAGGA

AGTGAAAAACTTGTAAAATATGTCCGGCAACCATAAATCAAAAAGCAACATAGCTTAGTAAGTATGTGAAAATTCCATAA

CCAAATGCAAGAAAAAGTGCATCAGTGTCAAAGTGAGACTGAATTACACATGAAACCGTCTCATTCAGCAACTCTTGAAG

GAGAGAAAGAGCTCGAAAGCCACTGATGCTCAGGTCAAGAGATTGTGTGCTCCGCGCTAGCCACCGG-GGGGCCTATTGA

AATTACCCCAAAAAAAAAGAGAACCAACC-GCCTAAAAGAAGGGAACCATGCCGCGATTAAGAAAGGTATGAACTATAAA

GTACCAACCAAATGCCTTTGAAAGGAAGTCCACAGGGGAGAAGTCTG

146_Campylopterus_curvipennis_20Nov-4

GGATTATGGCTACTGCTACTTCTAGGATGGTTAGTAGGAGTAGTACGCAGGCGGTTAGTGCAGAGATTGCGGGTATGATG

GAGAATAGGACTGCGGTGGCTGTTGAGATGAGTTGGATTAGTAGGTGTCCTGCTGTTAGGTTGGCTGTGAGGCGGACTCC

TAGGGCTAGAGGGCGGATCAGAAGGCTGGTAGTTTCAATTATGATTAAGGCCGGAATTAGTGGAGTTGGGGTTCCTTCGG

GTAGGAGGTGGCCAAGTGAAGCGGATGGTTGGTTTCGGAGGCCTGTGAGTAGAGTGGCTAGTCATAGTGGGAAGGCTAGT

GCTAGGTTTATAGATAATTGTGTGGTAGGTGTGAATGTGTAGGGGAGTAGGCCTAGTAGGTTGATTGAGAGAAGGAAAAT

TATCAGTGATGACAGGATAAGGGCTCATTTGTGGCCTTTTTTGTTCAATGGGATTATCAGTTGTTTGGTGATTATATAAA

TGAATCACGATTGTAGGGTGGATGTTCGGTTAGTGATTCATCGGTTGTGGGGGGAGGGGAGTAGTAGAGCTGGGAATAAT

AGTGAGATGAGGATTAGGGGGATTCCTAGTAGGTGGGGGCTTATAAATTGGTCGAAGAAGCTTAGGTTCATGGTCAGGGT

CAGGATGGGGGTTTTGTGTCTGTTAGGGGTTTTTCTAGGGGGGAGTTGGTGGGCGTGAATGAAAGGAGTTTGGGTTGGAT

GATTAGTGAAAAGGTCAGTCATGAAATAGTTATAACGAATAGTCATGGGTTTGGATTTAGTTGGGGCATATCATTAGGGA

GGGTTGGTAATCCTCTTTCTCTAGCTTAAAAGGCTAGTGCTGTTGCATAGCTTCCTAGTGGTTAAGATGATAGGAAAATT

AACATGATAAACATGATGATGGATGGTGATTATTTTTTAATGTAATCTCGGTGTTGAATATAGGTTAAAGTTAGATGATA

AAATGTGTGATAAAAATATCATGGATAATATGGAAATTATAACTTAGAACTCCAGTGGTGTTGGTTAGAAAGTTAGAGGA

AGTGAAAAACTTGTAAAATATGTCCGGCAACCATAAATCAAAAAGCAACATAGCTTAGTAAGTATGTGAGAATTCCATAA

CCAAATGCAAGAAAAAGTGCATCAGTGTCAAAGTGAGACTGAATTACACATGAAACCGTCTCATTCAGCAACTCTTGAAG

GAGAGAAAGAGCTCGAAAGCCACTGATGCTCAGGTCAAGAGATTGTGTGCTCCGCGCTAGCCACCGG-GGGGCCTATTGA

AATTACCCAAAAAAAAAAGAGAACCAACC-GCCAAAAAGAAGGGAACCATGCCGCGATTAAGAAAGGTATGAACTATAAA

GTACCAACCAAATGCCTTTGAAAGGAAGTCCACAGGGGAGAAGTCTG

147_Campylopterus_curvipennis_20Nov-5

GGATTATGGCTACTGCTACTTCTAGGATGGTTAGTAGGAGTAGTACGCAGGCGGTTAGTGCAGAGATTGCGGGTATGATG

GAGAATAGGACTGCGGTGGCTGTTGAGATGAGTTGGATTAGTAGGTGTCCTGCTGTTAGGTTGGCTGTGAGGCGGACTCC

TAGGGCTAGAGGGCGGATCAGAAGGCTGGTAGTTTCAATTATGATTAAGGCCGGAATTAGTGGAGTTGGGGTTCCTTCGG

GTAGGAGGTGGCCAAGTGAAGCGGATGGTTGGTTTCGGAGGCCTGTGAGTAGAGTGGCTAGTCATAGTGGGAAGGCTAGT

GCTAGGTTTATAGATAATTGTGTGGTAGGTGTGAATGTGTAGGGGAGTAGGCCTAGTAGGTTGATTGAGAGAAGGAAAAT

TATCAGTGATGACAGGATAAGGGCTCATTTGTGGCCTTTTTTGTTCAATGGGATTATCAGTTGTTTGGTGATTATATAAA

TGAATCACGATTGTAGGGTGGATGTTCGGTTAGTGATTCATCGGTTGTGGGGGGAGGGGAGTAGTAGAGCTGGGAATAAT

AGTGAGATGAGGATTAGGGGGATTCCTAGTAGGTGGGGGCTTATAAATTGGTCGAAGAAGCTTAGGTTCATGGTCAGGGT

CAGGATGGGGGTTTTGTGCCTGTTAGGGGTTTTTCTAGGGGGGAGTTGGTGGGCGTGAATGAAAGGAGTTTGGGTTGGAT

GATTAGTGAAAAGGTCAGTCATGAAATAGTTATAACGAATAGTCATGGGTTTGGATTTAGTTGGGGCATATCATTAGGGA

GGGTTGGTAATCCTCTTTCTCTAGCTTAAAAGGCTAGTGCTGTTGCATAGCTTCCTAGTGGTTAAGATGATAGGAAAATT

AACATGATAAACATGATGATGGATGGTGATTATTTTTTAATGTAATCTCGGTGTTGAATATAGGTTAAAGTTAGATGATA

AAATGTGTGATAAAAATATCATGGATAATATGGAAATTATAACTTAGAACTCCAGTGGTGTTGGTTAGAAAGTTAGAGGA

AGTGAAAAACTTGTAAAATATGTCCGGTAACCATAAATCAAAAAGCAACATAGCTTAGTAAGTATGTGAAAATTCCATAA

CCAAATGCAAGAAAAAGTGCATCAGTGTCAAAGTGAGACTGAATTACACATGAAACCGTCTCATTCAGCAACTCTTGAAG

GAGAGAAAGAGCTCGAAAGCCACTGATGCTCAGGTCAAGAGATTGTGTGCTCCGCGCTAGCCACCGG-GGGGCCTATTGA

AATTACCCAAAAAAAAAAGAGAACCAACC-GCCTAAAAGAAGGGAACCATGCCGCGATTAAGAAAGGTATGAACTATAAA

GTACCAACCAAATGCCTTTGAAAGGAAGTCCACAGGGGAGAAGTCTG

148_Campylopterus_curvipennis_20Nov-6

GGATTATGGCTACTGCTACTTCTAGGATGGTTAGTAGGAGTAGTACGCAGGCGGTTAGTGCAGAGATTGCGGGTATGATG

GAGAATAGGACTGCGGTGGCTGTTGAGATGAGTTGGATTAGTAGGTGTCCTGCTGTTAGGTTGGCTGTGAGGCGGACTCC

TAGGGCTAGAGGGCGGATCAGAAGGCTGGTAGTTTCAATTATGATTAAGGCCGGAATTAGTGGAGTTGGGGTTCCTTCGG

GTAGGAGGTGGCCAAGTGAAGCGGATGGTTGGTTTCGGAGGCCTGTGAGTAGAGTGGCTAGTCATAGTGGGAAGGCTAGT

GCTAGGTTTATAGATAATTGTGTGGTAGGTGTGAATGTGTAGGGGAGTAGGCCTAGTAGGTTGATTGAGAGAAGGAAAAT

TATCAGTGATGACAGGATGAGGGCTCATTTGTGGCCTTTTTTGTTCAATGGGATTATCAGTTGTTTGGTGATTATATAAA

TGAATCACGATTGTAGGGTGGATGTTCGGTTAGTGATTCATCGGTTGTGGGGGGAGGGGAGTAGTAGAGCTGGGAATAAT

AGTGAGATGAGGATTAGGGGGATTCCTAGTAGGTGGGGGCTTATAAATTGGTCGAAGAAGCTTAGGTTCATGGTCAGGGT

CAGGATGGGGGTTTTGTGTCTGTTAGGGGTTTTTCTAGGGGGGAGTTGGTGGGCGTGAATGAAAGGAGTTTGGGTTGGAT

GATTAGTGAAAAGGTCAGTCATGAAATAGTTATAACGAATAGTCATGGGTTTGGATTTAGTTGGGGCATATCATTAGGGA

GGGTTGGTAATCCTCTTTCTCTAGCTTAAAAGGCTAGTGCTGTTGCATAGCTTCCTAGTGGTTAAGATGATAGGAAAATT

AACATGATAAACATGATGATGGATGGTGATTATTTTTTAATGTAATCTCGGTGTTGAATATAGGTTAAAGTTAGATGATA

AAATGTGTGATAAAAATATCATGGATAATATGGAAATTATAACTTAGAACTCCAGTGGTGTTGGTTAGAAAGTTAGAGGA

AGTGAAAAACTTGTAAAATATGTCCGGCAACCATAAATCAAAAAGCAACATAGCTTAGTAAGTATGTGAAAATTCCATAA

CCAAATGCAAGAAAAAGTGCATCAGTGTCAAAGTGAGACTGAATTACACATGAAACCGTCTCATTCAGCAACTCTTGAAG

GAGAGAAAGAGCTCGAAAGCCACTGATGCCCAGGTCAAGAGATTGTGTGCTCCGCGCTAGCCACCGG-GGGGCCTATTGA

AATTACCCCAAAAAAAAAGAGAACCAACC-GCCAAAAAGAAGGGAACCATGCCGCGATTAAGAAAGGTATGAACTATAAA

GTACCAACCAAATGCCTTTGAAAGGAAGTCCACAGGGGAGAAGTCTG

149_Campylopterus_curvipennis_20Nov-7

GGATTATGGCTACTGCTACTTCTAGGATGGTTAGTAGGAGTAGTACGCAGGCGGTTAGTGCAGAGATTGCGGGTATGATG

GAGAATAGGACTGCGGTGGCTGTTGAGATGAGTTGGATTAGTAGGTGTCCTGCTGTTAGGTTGGCTGTGAGGCGGACTCC

TAGGGCTAGAGGGCGGATCAGAAGGCTGGTAGTTTCAATTATGATTAAGGCCGGAATTAGTGGAGTTGGGGTTCCTTCGG

GTAGGAGGTGGCCAAGTGAAGCGGATGGTTGGTTTCGGAGGCCTGTGAGTAGAGTGGCTAGTCATAGTGGGAAGGCTAGT

GCTAGGTTTATAGATAATTGTGTGGTAGGTGTGAATGTGTAGGGGAGTAGGCCTAGTAGGTTGATTGAGAGAAGGAAAAT

TATCAGTGATGACAGGATGAGGGCTCATTTGTGGCCTTTTTTGTTCAATGGGATTATCAGTTGTTTGGTGATTATATAAA

TGAATCACGATTGTAGGGTGGATGTTCGGTTAGTGATTCATCGGTTGTGGGGGGAGGGGAGTAGTAGAGCTGGGAATAAT

AGTGAGATGAGGATTAGGGGGATTCCTAGTAGGTGGGGGCTTATAAATTGGTCGAAGAAGCTTAGGTTCATGGTCAGGGT

CAGGATGGGGGTTTTGTGTCTGTTAGGGGTTTTTCTAGGGGGGAGTTGGTGGGCGTGAATGAAAGGAGTTTGGGTTGGAT

GATTAGTGAAAAGGTCAGTCATGAAATAGTTATAACGAATAGTCATGGGTTTGGATTTAGTTGGGGCATATCATTAGGGA

GGGTTGGTAATCCTCTTTCTCTAGCTTAAAAGGCTAGTGCTGTTGCATAGCTTCCTAGTGGTTAAGATGATAGGAAAATT

AACATGATAAACATGATGATGGATGGTGATTATTTTTTAATGTAATCTCGGTGTTGAATATAGGTTAAAGTTAGATGATA

AAATGTGTGATAAAAATATCATGGATAATATGGAAATTATAACTTAGAACTCCAGTGGTGTTGGTTAGAAAGTTAGAGGA

AGTGAAAAACTTGTAAAATATGTCCGGCAACCATAAATCAAAAAGCAACATAGCTTAGTAAGTATGTGAAAATTCCATAA

CCAAATGCAAGAAAAAGTGCATCAGTGTCAAAGTGAGACTGAATTACACATGAAACCGTCTCATTCAGCAACTCCTGAAG

GAGAGAAAGAGCTCGAAAGCCACTGATGCCCAGGTCAAGAGATTGTGTGCTCCGCGCTAGCCACCGG-GGGGCCTATTGA

AATTACCCCAAAAAAAAAGAGAACCAACC-GCCAAAAAGAAGGGAACCATGCCGCGATTAAGAAAGGTATGAACTATAAA

GTACCAACCAAATGCCTTTGAAAGGAAGTCCACAGGGGAGAAGTCTG

150_Campylopterus_curvipennis_20Nov-8

GGATTATGGCTACTGCTACTTCTAGGATGGTTAGTAGGAGTAGTACGCAGGCGGTTAGTGCAGAGATTGCGGGTATGATG

GAGAATAGGACTGCGGTGGCTGTTGAGATGAGTTGGATTAGTAGGTGTCCTGCTGTTAGGTTGGCTGTGAGGCGGACTCC

TAGGGCTAGAGGGCGGATCAGAAGGCTGGTAGTTTCAATTATGATTAAGGCCGGAATTAGTGGAGTTGGGGTTCCTTCGG

GTAGGAGGTGGCCAAGTGAAGCGGATGGTTGGTTTCGGAGGCCTGTGAGTAGAGTGGCTAGTCATAGTGGGAAGGCTAGT

GCTAGGTTTATAGATAATTGTGTGGTAGGTGTGAATGTGTAGGGGAGTAGGCCTAGTAGGTTGATTGAGAGAAGGAAAAT

TATCAGTGATGACAGGATAAGGGCTCATTTGTGGCCTTTTTTGTTCAATGGGATTATCAGTTGTTTGGTGATTATATAAA

TGAATCACGATTGTAGGGTGGATGTTCGGTTAGTGATTCATCGGTTGTGGGGGGAGGGGAGTAGTAGAGCTGGGAATAAT

AGTGAGATGAGGATTAGGGGGATTCCTAGTAGGTGGGGGCTTATAAATTGGTCGAAGAAGCTTAGGTTCATGGTCAGGGT

CAGGATGGGGGTTTTGTGCCTGTTAGGGGTTTTTCTAGGGGGGAGTTGGTGGGCGTGAATGAAAGGAGTTTGGGTTGGAT

GATTAGTGAAAAGGTCAGTCATGAAATAGTTATAACGAATAGTCATGGGTTTGGATTTAGTTGGGGCATATCATTAGGGA

GGGTTGGTAATCCTCTTTCTCTAGCTTAAAAGGCTAGTGCTGTTGCATAGCTTCCTAGTGGTTAAGATGATAGGAAAATT

AACATGATAAACATGATGATGGATGGTGATTATTTTTTAATGTAATCTCGGTGTTGAATATAGGTTAAAGTTAGATGATA

AAATGTGTGATAAAAATATCATGGATAATATGGAAATTATAACTTAGAACTCCAGTGGTGTTGGTTAGAAAGTTAGAGGA

AGTGAAAAACTTGTAAAATATGTCCGGCAACCATAAATTAAAAAGCAACATAGCTTAGTAAGTATGTGAAAATTCCATAA

CCAAATGCAAGAAAAAGTGCATCAGTGTCAAAGTGAGACTGAATTACACATGAAACCGTCTCATTCAGCAACTCTTGAAG

GAGAGAAAGAGCTCGAAAGCCACTGATGCTCAGGTCAAGAGATTGTGTGCTCCGCGCTAGCCACCGG-GGGGCCTATTGA

AATTACCCAAAAAAAAAAGAGAACCAACC-GCCTAAAAGAAGGGAACCATGCCGCGATTAAGAAAGGTATGAACTATAAA

GTACCAACCAAATGCCTTTGAAAGGAAGTCCACAGGGGAGAAGTCTG

151_Campylopterus_curvipennis_20Nov-9

GGATTATGGCTACTGCTACTTCTAGGATGGTTAGTAGGAGTAGTACGCAGGCGGTTAGTGCAGAGATTGCGGGTATGATG

GAGAATAGGACTGCGGTGGCTGTTGAGATGAGTTGGATTAGTAGGTGTCCTGCTGTTAGGTTGGCTGTGAGGCGGACTCC

TAGGGCTAGAGGGCGGATCAGAAGGCTGGTAGTTTCAATTATGATTAAGGCCGGAATTAGTGGAGTTGGGGTTCCTTCGG

GTAGGAGGTGGCCAAGTGAAGCGGATGGTTGGTTTCGGAGGCCTGTGAGTAGAGTGGCTAGTCATAGTGGGAAGGCTAGT

GCTAGGTTTATAGATAATTGTGTGGTAGGTGTGAATGTGTAGGGGAGTAGGCCTAGTAGGTTGATTGAGAGAAGGAAAAT

TATCAGTGATGACAGGATAAGGGCTCATTTGTGGCCTTTTTTGTTCAATGGGATTATCAGTTGTTTGGTGATTATATAAA

TGAATCACGATTGTAGGGTGGATGTTCGGTTAGTGATTCATCGGTTGTGGGGGGAGGGGAGTAGTAGAGCTGGGAATAAT

AGTGAGATGAGGATTAGGGGGATTCCTAGTAGGTGGGGGCTTATAAATTGGTCGAAGAAGCTTAGGTTCATGGTCAGGGT

CAGGATGGGGGTTTTGTGTCTGTTAGGGGTTTTTCTAGGGGGGAGTTGGTGGGCGTGAATGAAAGGAGTTTGGGTTGGAT

GATTAGTGAAAAGGTCAGTCATGAAATAGTTATAACGAATAGTCATGGGTTTGGATTTAGTTGGGGCATATCATTAGGGA

GGGTTGGTAATCCTCTTTCTCTAGCTTAAAAGGCTAGTGCTGTTGCATAGCTTCCTAGTGGTTAAGATGATAGGAAAATT

AACATGATAAACATGATGATGGATGGTGATTATTTTTTAATGTAATCTCGGTGTTGAATATAGGTTAAAGTTAGATGATA

AAATGTGTGATAAAAATATCATGGATAATATGGAAATTATAACTTAGAACTCCAGTGGTGTTGGTTAGAAAGTTAGAGGA

AGTGAAAAACTTGTAAAATATGTCCGGCAACCATAAATCAAAAAGCAACATAGCTTAGTAAGTATGTGAAAATTCCATAA

CCAAATGCAAGAAAAAGTGCATCAGTGTCAAAGTGAGACTGAATTACACATGAAACCGTCTCATTCAGCAACTCTTGAAG

GAGAGAAAGAGCTCGAAAGCCACTGATGCTCAGGTCAAGAGATTGTGTGCTCCGCGCTAGCCACCGG-GGGGCCTATTGA

AATTACCCAAAAAAAAAAGAGAACCAACC-GCCTAAAAGAAGGGAACCATGCCGCGATTAAGAAAGGTATGAACTATAAA

GTACCAACCAAATGCCTTTGAAAGGAAGTCCACAGGGGAGAAGTCTG

152_Campylopterus_curvipennis_20Nov-10

GGATTATGGCTACTGCTACTTCTAGGATGGTTAGTAGGAGTAGTACGCAGGCGGTTAGTGCAGAGATTGCGGGTATGATG

GAGAATAGGACTGCGGTGGCTGTTGAGATGAGTTGGATTAGTAGGTGTCCTGCTGTTAGGTTGGCTGTGAGGCGGACTCC

TAGGGCTAGAGGGCGGATCAGAAGGCTGGTAGTTTCAATTATGATTAAGGCCGGAATTAGTGGAGTTGGGGTTCCTTCGG

GTAGGAGGTGGCCAAGTGAAGCGGATGGTTGGTTTCGGAGGCCTGTGAGTAGAGTGGCTAGTCATAGTGGGAAGGCTAGT

GCTAGGTTTATAGATAATTGTGTGGTAGGTGTGAATGTGTAGGGGAGTAGGCCTAGTAGGTTGATTGAGAGAAGGAAAAT

TATCAGTGATGACAGGATGAGGGCTCATTTGTGGCCTTTTTTGTTCAATGGGATTATCAGTTGTTTGGTGATTATATAAA

TGAATCACGATTGTAGGGTGGATGTTCGGTTAGTGATTCATCGGTTGTGGGGGGAGGGGAGTAGTAGAGCTGGGAATAAT

AGTGAGATGAGGATTAGGGGGATTCCTAGTAGGTGGGGGCTTATAAATTGGTCGAAGAAGCTTAGGTTCATGGTCAGGGT

CAGGATGGGGGTTTTGTGTCTGTTAGGGGTTTTTCTAGGGGGGAGTTGGTGGGCGTGAATGAAAGGAGTTTGGGTTGGAT

GATTAGTGAAAAGGTCAGTCATGAAATAGTTATAACGAATAGTCATGGGTTTGGATTTAGTTGGGGCATATCATTAGGGA

GGGTTGGTAATCCTCTTTCTCTAGCTTAAAAGGCTAGTGCTGTTGCATAGCTTCCTAGTGGTTAAGATGATAGGAAAATT

AACATGATAAACATGATGATGGATGGTGATTATTTTTTAATGTAATCTCGGTGTTGAATATAGGTTAAAGTTAGATGATA

AAATGTGTGATAAAAATATCATGGATAATATGGAAATTATAACTTAGAACTCCAGTGGTGTTGGTTAGAAAGTTAGAGGA

AGTGAAAAACTTGTAAAATATGTCCGGCAACCATAAATCAAAAAGCAACATAGCTTAGTAAGTATGTGAAAATTCCATAA

CCAAATGCAAGAAAAAGTGCATCAGTGTCAAAGTGAGACTGAATTACACATGAAACCGTCTCATTCAGCAACTCTTGAAG

GAGAGAAAGAGCTCGAAAGCCACTGATGCCCAGGTCAAGAGATTGTGTGCTCCGCGCTAGCCACCGG-GGGGCCTATTGA

AATTACCCCAAAAAAAAAGAGAACCAACC-GCCAAAAAGAAGGGAACCATGCCGCGATTAAGAAAGGTATGAACTATAAA

GTACCAACCAAATGCCTTTGAAAGGAAGTCCACAGGGGAGAAGTCTG

153_Campylopterus_curvipennis_20Nov-11

GGATTATGGCTACTGCTACTTCTAGGATGGTTAGTAGGAGTAGTACGCAGGCGGTTAGTGCAGAGATTGCGGGTATGATG

GAGAATAGGACTGCGGTGGCTGTTGAGATGAGTTGGATTAGTAGGTGTCCTGCTGTTAGGTTGGCTGTGAGGCGGACTCC

TAGGGCTAGAGGGCGGATCAGAAGGCTGGTAGTTTCAATTATGATTAAGGCCGGAATTAGTGGAGTTGGGGTTCCTTCGG

GTAGGAGGTGGCCAAGTGAAGCGGATGGTTGGTTTCGGAGGCCTGTGAGTAGAGTGGCTAGTCATAGTGGGAAGGCTAGT

GCTAGGTTTATAGATAATTGTGTGGTAGGTGTGAATGTGTAGGGGAGTAGGCCTAGTAGGTTGATTGAGAGAAGGAAAAT

TATCAGTGATGACAGGATGAGGGCTCATTTGTGGCCTTTTTTGTTCAATGGGATTATCAGTTGTTTGGTGATTATATAAA

TGAATCACGATTGTAGGGTGGATGTTCGGTTAGTGATTCATCGGTTGTGGGGGGAGGGGAGTAGTAGAGCTGGGAATAAT

AGTGAGATGAGGATTAGGGGGATTCCTAGTAGGTGGGGGCTTATAAATTGGTCGAAGAAGCTTAGGTTCATGGTCAGGGT

CAGGATGGGGGTTTTGTGTCTGTTAGGGGTTTTTCTAGGGGGGAGTTGGTGGGCGTGAATGAAAGGAGTTTGGGTTGGAT

GATTAGTGAAAAGGTCAGTCATGAAATAGTTATAACGAATAGTCATGGGTTTGGATTTAGTTGGGGCATATCATTAGGGA

GGGTTGGTAATCCTCTTTCTCTAGCTTAAAAGGCTAGTGCTGTTGCATAGCTTCCTAGTGGTTAAGATGATAGGAAAATT

AACATGATAAACATGATGATGGATGGTGATTATTTTTTAATGTAATCTCGGTGTTGAATATAGGTTAAAGTTAGATGATA

AAATGTGTGATAAAAATATCATGGATAATATGGAAATTATAACTTAGAACTCCAGTGGTGTTGGTTAGAAAGTTAGAGGA

AGTGAAAAACTTGTAAAATATGTCCGGCAACCATAAATCAAAAAGCAACATAGCTTAGTAAGTATGTGAAAATTCCATAA

CCAAATGCAAGAAAAAGTGCATCAGTGTCAAAGTGAGACTGAATTACACATGAAACCGTCTCATTCAGCAACTCCTGAAG

GAGAGAAAGAGCTCGAAAGCCACTGATGCCCAGGTCAAGAGATTGTGTGCTCCGCGCTAGCCACCGG-GGGGCCTATTGA

AATTACCCCAAAAAAAAAGAGAACCAACC-GCCAAAAAGAAGGGAACCATGCCGCGATTAAGAAAGGTATGAACTATAAA

GTACCAACCAAATGCCTTTGAAAGGAAGTCCACAGGGGAGAAGTCTG

154_Campylopterus_curvipennis_20Nov-12

GGATTATGGCTACTGCTACTTCTAGGATGGTTAGTAGGAGTAGTACGCAGGCGGTTAGTGCAGAGATTGCGGGTATGATG

GAGAATAGGACTGCGGTGGCTGTTGAGATGAGTTGGATTAGTAGGTGTCCTGCTGTTAGGTTGGCTGTGAGGCGGACTCC

TAGGGCTAGAGGGCGGATCAGAAGGCTGGTAGTTTCAATTATGATTAAGGCCGGAATTAGTGGAGTTGGGGTTCCTTCGG

GTAGGAGGTGGCCAAGTGAAGCGGATGGTTGGTTTCGGAGGCCTGTGAGTAGAGTGGCTAGTCATAGTGGGAAGGCTAGT

GCTAGGTTTATAGATAATTGTGTGGTAGGTGTGAATGTGTAGGGGAGTAGGCCTAGTAGGTTGATTGAGAGAAGGAAAAT

TATCAGTGATGACAGGATAAGGGCTCATTTGTGGCCTTTTTTGTTCAATGGGATTATCAGTTGTTTGGTGATTATATAAA

TGAATCACGATTGTAGGGTGGATGTTCGGTTAGTGATTCATCGGTTGTGGGGGGAGGGGAGTAGTAGAGCTGGGAATAAT

AGTGAGATGAGGATTAGGGGGATTCCTAGTAGGTGGGGGCTTATAAATTGGTCGAAGAAGCTTAGGTTCATGGTCAGGGT

CAGGATGGGGGTTTTGTGCCTGTTAGGGGTTTTTCTAGGGGGGAGTTGGTGGGCGTGAATGAAAGGAGTTTGGGTTGGAT

GATTAGTGAAAAGGTCAGTCATGAAATAGTTATAACGAATAGTCATGGGTTTGGATTTAGTTGGGGCATATCATTAGGGA

GGGTTGGTAATCCTCTTTCTCTAGCTTAAAAGGCTAGTGCTGTTGCATAGCTTCCTAGTGGTTAAGATGATAGGAAAATT

AACATGATAAACATGATGATGGATGGTGATTATTTTTTAATGTAATCTCGGTGTTGAATATAGGTTAAAGTTAGATGATA

AAATGTGTGATAAAAATATCATGGATAATATGGAAATTATAACTTAGAACTCCAGTGGTGTTGGTTAGAAAGTTAGAGGA

AGTGAAAAACTTGTAAAATATGTCCGGTAACCATAAATCAAAAAGCAACATAGCTTAGTAAGTATGTGAAAATTCCATAA

CCAAATGCAAGAAAAAGTGCATCAGTGTCAAAGTGAGACTGAATTACACATGAAACCGTCTCATTCAGCAACTCTTGAAG

GAGAGAAAGAGCTCGAAAGCCACTGATGCTCAGGTCAAGAGATTGTGTGCTCCGCGCTAGCCACCGG-GGGGCCTATTGA

AATTACCCAAAAAAAAAAGAGAACCAACC-GCCTAAAAGAAGGGAACCATGCCGCGATTAAGAAAGGTATGAACTATAAA

GTACCAACCAAATGCCTTTGAAAGGAAGTCCACAGGGGAGAAGTCTG

155_Campylopterus_curvipennis_20Nov-13

GGATTATGGCTACTGCTACTTCTAGGATGGTTAGTAGGAGTAGTACGCAGGCGGTTAGTGCAGAGATTGCGGGTATGATG

GAGAATAGGACTGCGGTGGCTGTTGAGATGAGTTGGATTAGTAGGTGTCCTGCTGTTAGGTTGGCTGTGAGGCGGACTCC

TAGGGCTAGAGGGCGGATCAGAAGGCTGGTAGTTTCAATTATGATTAAGGCCGGAATTAGTGGAGTTGGGGTTCCTTCGG

GTAGGAGGTGGCCAAGTGAAGCGGATGGTTGGTTTCGGAGGCCTGTGAGTAGAGTGGCTAGTCATAGTGGGAAGGCTAGT

GCTAGGTTTATAGATAATTGTGTGGTAGGTGTGAATGTGTAGGGGAGTAGGCCTAGTAGGTTGATTGAGAGAAGGAAAAT

TATCAGTGATGACAGGATAAGGGCTCATTTGTGGCCTTTTTTGTTCAATGGGATTATCAGTTGTTTGGTGATTATATAAA

TGAATCACGATTGTAGGGTGGATGTTCGGTTAGTGATTCATCGGTTGTGGGGGGAGGGGAGTAGTAGAGCTGGGAATAAT

AGTGAGATGAGGATTAGGGGGATTCCTAGTAGGTGGGGGCTTATAAATTGGTCGAAGAAGCTTAGGTTCATGGTCAGGGT

CAGGATGGGGGTTTTGTGCCTGTTAGGGGTTTTTCTAGGGGGGAGTTGGTGGGCGTGAATGAAAGGAGTTTGGGTTGGAT

GATTAGTGAAAAGGTCAGTCATGAAATAGTTATAACGAATAGTCATGGGTTTGGATTTAGTTGGGGCATATCATTAGGGA

GGGTTGGTAATCCTCTTTCTCTAGCTTAAAAGGCTAGTGCTGTTGCATAGCTTCCTAGTGGTTAAGATGATAGGAAAATT

AACATGATAAACATGATGATGGATGGTGATTATTTTTTAATGTAATCTCGGTGTTGAATATAGGTTAAAGTTAGATGATA

AAATGTGTGATAAAAATATCATGGATAATATGGAAATTATAACTTAGAACTCCAGTGGTGTTGGTTAGAAAGTTAGAGGA

AGTGAAAAACTTGTAAAATATGTCCGGCAACCATAAATCAAAAAGCAACATAGCTTAGTAAGTATGTGAAAATTCCATAA

CCAAATGCAAGAAAAAGTGCATCAGTGTCAAAGTGAGACTGAATTACACATGAAACCGTCTCATTCAGCAACTCTTGAAG

GAGAGAAAGAGCTCGAAAGCCACTGATGCTCAGGTCAAGAGATTGTGTGCTCCGCGCTAGCCACCGG-GGGGCCTATTGA

AATTACCCAAAAAAAAAAGAGAACCAACC-GCCTAAAAGAAGGGAACCATGCCGCGATTAAGAAAGGTATGAACTATAAA

GTACCAACCAAATGCCTTTGAAAGGAAGTCCACAGGGGAGAAGTCTG

156_Campylopterus_curvipennis_20Nov-14

GGATTATGGCTACTGCTACTTCTAGGATGGTTAGTAGGAGTAGTACGCAGGCGGTTAGTGCAGAGATTGCGGGTATGATG

GAGAATAGGACTGCGGTGGCTGTTGAGATGAGTTGGATTAGTAGGTGTCCTGCTGTTAGGTTGGCTGTGAGGCGGACTCC

TAGGGCTAGAGGGCGGATCAGAAGGCTGGTAGTTTCAATTATGATTAAGGCCGGAATTAGTGGAGTTGGGGTTCCTTCGG

GTAGGAGGTGGCCAAGTGAAGCGGATGGTTGGTTTCGGAGGCCTGTGAGTAGAGTGGCTAGTCATAGTGGGAAGGCTAGT

GCTAGGTTTATAGATAATTGTGTGGTAGGTGTGAATGTGTAGGGGAGTAGGCCTAGTAGGTTGATTGAGAGAAGGAAAAT

TATCAGTGATGACAGGATAAGGGCTCATTTGTGGCCTTTTTTGTTCAGTGGGATTATCAGTTGTTTGGTGATTATATAAA

TGAATCACGATTGTAGGGTGGATGTTCGGTTAGTGATTCATCGGTTGTGGGGGGAGGGGAGTAGTAGAGCTGGGAATAAT

AGTGAGATGAGGATTAGGGGGATTCCTAGTAGGTGGGGGCTTATAAATTGGTCGAAGAAGCTTAGGTTCATGGTCAGGGT

CAGGATGGGGGTTTTGTGTCTGTTAGGGGTTTTTCTAGGGGGGAGTTGGTGGGCGTGAATGAAAGGAGTTTGGGTTGGAT

GATTAGTGAAAAGGTCAGTCATGAAATAGTTATAACGAATAGTCATGGGTTTGGATTTAGTTGGGGCATATCATTAGGGA

GGGTTGGTAATCCTCTTTCTCTAGCTTAAAAGGCTAGTGCTGTTGCATAGCTTCCTAGTGGTTAAGATGATAGGAAAATT

AACATGATAAACATGATGATGGATGGTGATTATTTTTTAATGTAATCTCGGTGTTGAATATAGGTTAAAGTTAGATGATA

AAATGTGTGATAAAAATATCATGGATAATATGGAAATTATAACTTAGAACTCCAGTGGTGTTGGTTAGAAAGTTAGAGGA

AGTGAAAAACTTGTAAAATATGTCCGGCAACCATAAATCAAAAAGCAACATAGCTTAGTAAGTATGTGAAAATTCCATAA

CCAAATGCAAGAAAAAGTGCATCAGTGTCAAAGTGAGACTGAATTACACATGAAACCGTCTCATTCAGCAACTCTTGAAG

GAGAGAAAGAGCTCGAAAGCCACTGATGCTCAGGTCAAGAGATTGTGTGCTCCGCGCTAGCCACCGG-GGGGCCTATTGA

AATTACCCAAAAAAAAAAGAGAACCAACC-GCCTAAAAGAAGGGAACCATGCCGCGATTAAGAAAGGTATGAACTATAAA

GTACCAACCAAATGCCTTTGAAAGGAAGTCCACAGGGGAGAAGTCTG

157_Campylopterus_curvipennis_20Nov-15

GGATTATGGCTACTGCTACTTCTAGGATGGTTAGTAGGAGTAGTACGCAGGCGGTTAGTGCAGAGATTGCGGGTATGATG

GAGAATAGGACTGCGGTGGCTGTTGAGATGAGTTGGATTAGTAGGTGTCCTGCTGTTAGGTTGGCTGTGAGGCGGACTCC

TAGGGCTAGAGGGCGGATCAGAAGGCTGGTAGTTTCAATTATGATTAAGGCCGGAATTAGTGGAGTTGGGGTTCCTTCGG

GTAGGAGGTGGCCAAGTGAAGCGGATGGTTGGTTTCGGAGGCCTGTGAGTAGAGTGGCTAGTCATAGTGGGAAGGCTAGT

GCTAGGTTTATAGATAATTGTGTGGTAGGTGTGAATGTGTAGGGGAGTAGGCCTAGTAGGTTGATTGAGAGAAGGAAAAT

TATCAGTGATGACAGGATGAGGGCTCATTTGTGGCCTTTTTTGTTCAATGGGATTATCAGTTGTTTGGTGATTATATAAA

TGAATCACGATTGTAGGGTGGATGTTCGGTTAGTGATTCATCGGTTGTGGGGGGAGGGGAGTAGTAGAGCTGGGAATAAT

AGTGAGATGAGGATTAGGGGGATTCCTAGTAGGTGGGGGCTTATAAATTGGTCGAAGAAGCTTAGGTTCATGGTCAGGGT

CAGGATGGGGGTTTTGTGTCTGTTAGGGGTTTTTCTAGGGGGGAGTTGGTGGGCGTGAATGAAAGGAGTTTGGGTTGGAT

GATTAGTGAAAAGGTCAGTCATGAAATAGTTATAACGAATAGTCATGGGTTTGGATTTAGTTGGGGCATATCATTAGGGA

GGGTTGGTAATCCTCTTTCTCTAGCTTAAAAGGCTAGTGCTGTTGCATAGCTTCCTAGTGGTTAAGATGATAGGAAAATT

AACATGATAAACATGATGATGGATGGTGATTATTTTTTAATGTAATCTCGGTGTTGAATATAGGTTAAAGTTAGATGATA

AAATGTGTGATAAAAATATCATGGATAATATGGAAATTATAACTTAGAACTCCAGTGGTGTTGGTTAGAAAGTTAGAGGA

AGTGAAAAACTTGTAAAATATGTCCGGTAACCATAAATCAAAAAGCAACATAGCTTAGTAAGTATGTGAAAATTCCATAA

CCAAATGCAAGAAAAAGTGCATCAGTGTCAAAGTGAGACTGAATTACACATGAAACCGTCTCATTCAGCAATTCCTGAAG

GAGAGAAAGAGCTCGAAAGCCACTGATGCCCAGGTCAAGAGATTGTGTGCTCCGCGCTAGCCACCGG-GGGGCCTATTGA

AATTACCCCAAAAAAAAAGAGAACCAACC-GCCAAAAAGAAGGGAACCATGCCGCGATTAAGAAAGGTATGAACTATAAA

GTACCAACCAAATGCCTTTGAAAGGAAGTCCACAGGGGAGAAGTCTG

158_Campylopterus_curvipennis_3gar-1

GGATTATGGCTACTGCTACTTCTAGGATGGTTAGTAGGAGTAGTACGCAGGCGGTTAGTGCAGAGATTGCGGGTATGATG

GAGAATAGGACTGCGGTGGCTGTTGAGATGAGTTGGATTAGTAGGTGTCCTGCTGTTAGGTTGGCTGTGAGGCGGACTCC

TAGGGCTAGAGGGCGGATCAGAAGGCTGGTAGTTTCAATTATGATTAAGGCCGGAATTAGTGGAGTTGGGGTTCCTTCGG

GTAGGAGGTGGCCAAGTGAAGCGGATGGTTGGTTTCGGAGGCCTGTGAGTAGAGTGGCTAGTCATAGTGGGAAGGCTAGT

GCTAGGTTTATAGATAATTGTGTGGTAGGTGTGAATGTGTAGGGGAGTAGGCCTAGTAGGTTGATTGAGAGAAGGAAAAT

TATCAGTGATGACAGGATGAGGGCTCATTTGTGGCCTTTTTTGTTCAATGGGATTATCAGTTGTTTGGTGATTATATAAA

TGAATCACGATTGTAGGGTGGATGTTCGGTTAGTGATTCATCGGTTGTGGGGGGAGGGGAGTAGTAGAGCTGGGAATAAT

AGTGAGATGAGGATTAGGGGGATTCCTAGTAGGTGGGGGCTTATAAATTGGTCGAAGAAGCTTAGGTTCATGGTCAGGGT

CAGGATGGGGGTTTTGTGTCTGTTAGGGGTTTTTCTAGGGGGGAGTTGGTGGGCGTGAATGAAAGGAGTTTGGGTTGGAT

GATTAGTGAAAAGGTCAGTCATGAAATAGTTATAACGAATAGTCATGGGTTTGGATTTAGTTGGGGCATATCATTAGGGA

GGGTTGGTAATCCTCTTTCTCTAGCTTAAAAGGCTAGTGCTGTTGCATAGCTTCCTAGTGGTTAAGATGATAGGAAAATT

AACATGATAAACATGATGATGGATGGTGATTATTTTTTAATGTAATCTCGGTGTTGAATATAGGTTAAAGTTAGATGATA

AAATGTGTGATAAAAATATCATGGATAATATGGAAATTATAACTTAGAACTCCAGTGGTGTTGGTTAGAAAGTTAGAGGA

AGTGAAAAACTTGTAAAATATGTCCGGCAACCATAAATCAAAAAGCAACATAGCTTAGTAAGTATGTGAAAATTCCATAA

CCAAATGCAAGAAAAAGTGCATCAGTGTCAAAGTGAGACTGAATTACACATGAAACCGTCTCATTCAGCAACTCTTGAAG

GAGAGAAAGAGCTCGAAAGCCACTGATGCCCAGGTCAAGAGATTGTGTGCTCCGCGCTAGCCACCGG-GGGGCCTATTGA

AATTACCCCAAAAAAAAAGAGAACCAACC-GCCAAAAAGAAGGGAACCATGCCGCGATTAAGAAAGGTATGAACTATAAA

GTACCAACCAAATGCCTTTGAAAGGAAGTCCACAGGGGAGAAGTCTG

159_Campylopterus_curvipennis_3gar-2

GGATTATGGCTACTGCTACTTCTAGGATGGTTAGTAGGAGTAGTACGCAGGCGGTTAGTGCAGAGATTGCGGGTATGATG

GAGAATAGGACTGCGGTGGCTGTTGAGATGAGTTGGATTAGTAGGTGTCCTGCTGTTAGGTTGGCTGTGAGGCGGACTCC

TAGGGCTAGAGGGCGGATCAGAAGGCTGGTAGTTTCAATTATGATTAAGGCCGGAATTAGTGGAGTTGGGGTTCCTTCGG

GTAGGAGGTGGCCAAGTGAAGCGGATGGTTGGTTTCGGAGGCCTGTGAGTAGAGTGGCTAGTCATAGTGGGAAGGCTAGT

GCTAGGTTTATAGATAATTGTGTGGTAGGTGTGAATGTGTAGGGGAGTAGGCCTAGTAGGTTGATTGAGAGAAGGAAAAT

TATCAGTGATGACAGGATAAGGGCTCATTTGTGGCCTTTTTTGTTCAATGGGATTATCAGTTGTTTGGTGATTATATAAA

TGAATCACGATTGTAGGGTGGATGTTCGGTTAGTGATTCATCGGTTGTGGGGGGAGGGGAGTAGTAGAGCTGGGAATAAT

AGTGAGATGAGGATTAGGGGGATTCCTAGTAGGTGGGGGCTTATAAATTGGTCGAAGAAGCTTAGGTTCATGGTCAGGGT

CAGGATGGGGGTTTTGTGCCTGTTAGGGGTTTTTCTAGGGGGGAGTTGGTGGGCGTGAATGAAAGGAGTTTGGGTTGGAT

GATTAGTGAAAAGGTCAGTCATGAAATAGTTATAACGAATAGTCATGGGTTTGGATTTAGTTGGGGCATATCATTAGGGA

GGGTTGGTAATCCTCTTTCTCTAGCTTAAAAGGCTAGTGCTGTTGCATAGCTTCCTAGTGGTTAAGATGATAGGAAAATT

AACATGATAAACATGATGATGGATGGTGATTATTTTTTAATGTAATCTCGGTGTTGAATATAGGTTAAAGTTAGATGATA

AAATGTGTGATAAAAATATCATGGATAATATGGAAATTATAACTTAGAACTCCAGTGGTGTTGGTTAGAAAGTTAGAGGA

AGTGAAAAACTTGTAAAATATGTCCGGCAACCATAAATCAAAAAGCAACATAGCTTAGTAAGTATGTGAAAATTCCATAA

CCAAATGCAAGAAAAAGTGCATCAGTGTCAAAGTGAGACTGAATTACACATGAAACCGTCTCATTCAGCAACTCTTGAAG

GAGAGAAAGAGCTCGAAAGCCACTGATGCTCAGGTCAAGAGATTGTGTGCTCCGCGCTAGCCACCGG-GGGGCCTATTGA

AATTACCCAAAAAAAAAAGAGAACCAACC-GCCTAAAAGAAGGGAACCATGCCGCGATTAAGAAAGGTATGAACTATAAA

GTACCAACCAAATGCCTTTGAAAGGAAGTCCACAGGGGAGAAGTCTG

160_Campylopterus_curvipennis_3gar-3

GGATTATGGCTACTGCTACTTCTAGGATGGTTAGTAGGAGTAGTACGCAGGCGGTTAGTGCAGAGATTGCGGGTATGATG

GAGAATAGGACTGCGGTGGCTGTTGAGATGAGTTGGATTAGTAGGTGTCCTGCTGTTAGGTTGGCTGTGAGGCGGACTCC

TAGGGCTAGAGGGCGGATCAGAAGGCTGGTAGTTTCAATTATGATTAAGGCCGGAATTAGTGGAGTTGGGGTTCCTTCGG

GTAGGAGGTGGCCAAGTGAAGCGGATGGTTGGTTTCGGAGGCCTGTGAGTAGAGTGGCTAGTCATAGTGGGAAGGCTAGT

GCTAGGTTTATAGATAATTGTGTGGTAGGTGTGAATGTGTAGGGGAGTAGGCCTAGTAGGTTGATTGAGAGAAGGAAAAT

TATCAGTGATGACAGGATAAGGGCTCATTTGTGGCCTTTTTTGTTCAATGGGATTATCAGTTGTTTGGTGATTATATAAA

TGAATCACGATTGTAGGGTGGATGTTCGGTTAGTGATTCATCGGTTGTGGGGGGAGGGGAGTAGTAGAGCTGGGAATAAT

AGTGAGATGAGGATTAGGGGGATTCCTAGTAGGTGGGGGCTTATAAATTGGTCGAAGAAGCTTAGGTTCATGGTCAGGGT

CAGGATGGGGGTTTTGTGCCTGTTAGGGGTTTTTCTAGGGGGGAGTTGGTGGGCGTGAATGAAAGGAGTTTGGGTTGGAT

GATTAGTGAAAAGGTCAGTCATGAAATAGTTATAACGAATAGTCATGGGTTTGGATTTAGTTGGGGCATATCATTAGGGA

GGGTTGGTAATCCTCTTTCTCTAGCTTAAAAGGCTAGTGCTGTTGCATAGCTTCCTAGTGGTTAAGATGATAGGAAAATT

AACATGATAAACATGATGATGGATGGTGATTATTTTTTAATGTAATCTCGGTGTTGAATATAGGTTAAAGTTAGATGATA

AAATGTGTGATAAAAATATCATGGATAATATGGAAATTATAACTTAGAACTCCAGTGGTGTTGGTTAGAAAGTTAGAGGA

AGTGAAAAACTTGTAAAATATGTCCGGCAACCATAAATCAAAAAGCAACATAGCTTAGTAAGTATGTGAAAATTCCATAA

CCAAATGCAAGAAAAAGTGCATCAGTGTCAAAGTGAGACTGAATTACACATGAAACCGTCTCATTCAGCAACTCTTGAAG

GAGAGAAAGAGCTCGAAAGCCACTGATGCTCAGGTCAAGAGATTGTGTGCTCCGCGCTAGCCACCGG-GGGGCCTATTGA

AATTACCCAAAAAAAAAAGAGAACCAACC-GCCTAAAAGAAGGGAACCATGCCGCGATTAAGAAAGGTATGAACTATAAA

GTACCAACCAAATGCCTTTGAAAGGAAGTCCACAGGGGAGAAGTCTG

161_Campylopterus_hemileucurus

GGATTATGGCTACTGCTACTTCTAGGATGGTTAGTAGGAGTAGGATGCAGGCGGTTAGGGCAGAGATAGTGGGTATAATG

GAAAATAGAACTGCAGTGGCTGTTGAGATGAGTTGGATCAGTAAGTGCCCCGCGGTTAGGTTGGCTGTGAGGCGTACTCC

TAGGGCTAGGGGACGAATTAGGAGGCTAGTGGTTTCGATTATAATTAGGGCCGGGATTAGTGGGGTGGGGGTGCCTTCGG

GCAGGAGGTGGCCTAGTGAGGCGGATGGTTGATTTCGGAGGCCCGTGAGGAGGGTGGCCAGTCACAGTGGGAAGGCTAGT

GCCAGGTTTATAGATAGTTGTGTAGTGGGAGTGAATGTGTATGGAAGTAGGCCCAACAGGTTGATTGAGAGAAGGAAAAT

TATTAGTGATGATAGGATGAGGGCTCATTTGTGGCCTTTCTTGTTTAGTGGGGTTATTAGCTGTTTTGTGGTTGTATAAA

TAAATCATGATTGTAGGGTGGATACTCGGTTAGTTAGCCATCGGTTGTGGGGGGAGGGGAATAGTAGGGCTGGGAATAGT

AATGAGATGAGGATTAGGGGGATTCCTAGCAGGTGGGGGCTTATAAATTGGTCGAAGAAGCTTAGGTTCATGGTCAGGGT

CAGGATGAGCTCTTTGCGCTTATTGAGATTTTTTCTGAGGGGGTGTTAGTGGGTATGAATGAGAGGAGTTTGGGTTGAAT

GATTAGTGAGAAGGTCAGTCATGACACAATTATAATAAATAATCATGGGTTTGGGTTTAGTTGGGGCATGTCATTAGGGA

GGGTTAGTAATCCTCTTTTTCTAGCTTAAAAGGCTAGTGCTGTTGCATAGCTTCCTAGTGGTTAGGATGATAGGAAAATT

AACATGATAAACACGATGATGAATGGTAATTATTTTTTAATGTAACTCCAGTGTTGAAGATAGATTAAAGTTGAAGAATA

AAATGGGTGATGAAAATATGATGGATGGTATATAAATTATAACTTAGAACTCCAGTGGTGTTGGTTAGAAAGTTAGAGGA

AGTGAAAAATTTGTAAAATATGTCCAGCGACCATAAACTAAAAAGCAACATAGCTTAGTAGGTATGTGAGAATTCCATAA

CCAGATGCTATCCAAAGTGCATCAGTGTCAAGATGAGACTCAGTAACACATGAAACCGTCTCATTCAGCAATCCTTGAAA

GAGAGGAAAGGCCCGGGGGCCACTGATGCTCAGGTCAAGAGATTGTATGCTCTGCGTTAGCCACCGGGGGGGCCTGTTGA

AGTTATCCAGAAAAAAAAAAGAACCAACCACGCTAAGAGAAGGGAACCATGCCGCGATTAAGAGAGAGATGAAATATAAA

GTACCAACCAAATGCCTTTGAAGAGAAGCTTATAGGGGGGCAATCTA

162_Campylopterus_rufus

GGATTATGGCTACTGCTACCTCTAGGACGGTTAGTAGGAGTAGTACGCAGGCGGTCAATGCAGAGATTGCGGGTATGATG

GAGAATAGAACTGCGGTGGCTGTTGAGATAAGTTGGATTAGTAGGTGTCCTGCTGTTAGGTTGGCTGTGAGGCGGACTCC

TAGGGCTAAGGGGCGGATCAGGAGGCTGGTAGTTTCGATTATAATTAGGGCTGGGATTAGTGGAGTTGGGGTCCCTTCGG

GTAGGAGATGGCCTAGTGAAGCGGACGGTTGGTTTCGGAGGCCTGTGAGTAGGGTAGCTAGTCATAGTGGAAAGGCTAGT

GCTAGGTTTATAGATAGTTGTGTAGTGGGTGTGAATGTGTATGGGAGTAGGCCAAGTAGGTTGATTGAGAGAAGGAAGAT

TATTAGTGATGATAGGATGAGGGCTCATTTGTGGCCTTTTTTGTCTAATGGGATTATCAGTTGTTTTGTGACTATATAGA

TGAATCACGATTGTAGGGTGGATGTTCGGTTAGTGATTCATCGGTTTTGGGGGGAGGGGAATAGTAGAGTTGGGAATAGT

AGTGAGATGAGGATTAGGGGGATTCCTAGCAGGTGGGGGCTTATAAATTGGTCGAAGAAGCTTAGGTTCATGGTCAGGGT

CAGGATGGAGTTTTTGTATTTATTAGGGATTTTTCTGAGGGAGAGTTAGTGGGTGTGAATGAGAGGAGTTTGGGTTGGAT

GATTAGTGAAAAGGTCAATCATGATATAGTTATAATGAATAGTCATGGGTTTGGGTTTAGTTGAGGCATGTCATTAGGGA

GGGTTAGTAACCCTCTTTCTCTAGCTTAAAAGGCTAGTGCTGTTGCATAGCTTCCTAGTGGTTAGGATGATAGGAAAATT

AACATAATAAACACGATGATGAATGGTATTTATTTTTTAATGTGATTCCAGTGTTGAAGATAGGTTAAAGTTGGATAACA

AAATGTGTGATAAAAATATCATGGATAATGTAGAAATTATAACTTAGAACTCCAGTGGTGTTGGTTAGAAAGTTAGAGGA

AATGAAAAATTTGTAAAATATGTCCAGCGACCATAAACTAAAAAGCAACATAGCTTAGTAGGTATGTGAGAATTCCATAA

CCAAATGCAAAGCAAAGTGCATCAGTGTCAAAATGAGACTCAGTTACACATGAAACCGTCTCATTCAGCAATCCTTGAAA

GAGAGAAAAGGCCCGGGGGCCACTGATGCTCAGGTCAAGAGATTGTGTGCTCCGCGCCAGCCACCGG-GGGGCCCGTTGA

AATTACCCCAAAAAAAAAGAGAACCAACC-GCCTAAAAGAAGGGAACCATGCCGCGATTAAGAGAGGTATGAACTATAAA

GTACCAACCAAATGCCTTTGAAGAGGA-CCTATAGGG-AGTAATCTA

163_Campylopterus_largipennis

GGATTATGGCTACTGCTACCTCTAGGATGGTTAGTAGGAGAAGGATGCAGGCGGTTAGGGCGGAGATAGCGGGTATGATG

GAGAATAGAACTGCGGTGGCTGTTGAGATGAGTTGAATAAGTAAGTGCCCTGCTGTTAGGTTGGCTGTAAGGCGTACTCC

TAGGGCTAGGGGGCGAATTAGGAGGCTAGTAGTCTCGATTATGATTAGGGCTGGGATTAGTGGGGTAGGGGTGCCTTCGG

GTAGGAGGTGACCTAGTGAGGCGGATGGTTGGTTTCGGAGGCCCGTGAGGAGGGTGGCTAGTCACAGCGGGAAGGCCAGT

GCTAGGTTCATGGATAGTTGTGTAGTAGGGGTGAATGTATATGGGAGTAGGCCGAGCAGGTTGATCGAAAGAAGGAAAAT

TATTAGTGATGATAGGATGAGGGCCCATTTGTGGCCTTTTTTATCTAGGGGGATTATTAGTTGTTTTGTGGTTGTATAAA

TAAGTCATGATTGTAAGGTGGACACTCGGTTGGTGACTCATCGGTTACGAGGGGAGGGGAGTAATAGGGCTGGGAATAGT

AGTGAGATGAGGATCAGGGGGATTCCTAGAAGGTGAGGGCTTATAAACTGGTCGAAGAAGCTTAGGTTCATGGTCAGGGT

CAGGATGGGCTTTTTGTGTTTGTTGAGGATTTTTCTGAGGGGGTGTTAGTGGGTGTGAATGAGAGGAGCTTGGGTTGAAT

GATCAATGAGAAGGTTAATCACGATATAATTATGGTAAATAGTCATGGATTTGGATTTAGTTGGGGCATATCATTAGGGA

GGATTAGTAATCCTCTTTCTCTAGCTTAAAAGGCTAGTGCTGTTGCATAGCTTCCTAGTGGTTAGGATGATAGGAAAATT

AACATGATAAATACGATGATAGATCG-AATTATTTTTTAATGTAACTTCAGTGTTGGAGGAAGGTTAAAGTTGAAGAATA

AAATGCGTGATAAAAACATGATGAATGGTGTGTAAATTATAACTTAAAACTCCAGTGGTGTTGGTTAGAAAGTTAGAGGA

AGTGAAAAATTCGTAAAATATGTCCAGCGACCATGAATCAAAAAGCAACATAGCTTAGTAGGTATGTGAGAATTCCATAA

CCAGATGCCAGAAAAAGTGCATCAGTGTCAAGATGAGACTCAGTTACACATGAAACCGTCTCATTCAGCAACTCTTGAAG

GAGAGAAAAGGCCCGGGGGCCACTGATGCTCAGGTCAAGAGATTGTATGGTCCGCGCTAGCCACCGG-GGGGCCTATTGA

GGTTATCCAGAAAAAAAAAGGAACCAACCGCGCTAAAAAGAG-AAA--ATGCCGCGATTAAGAGGGAGATGTAATATAAA

GTACCAACCAAATTCCTCTGAAGTGGAGTCTCCAGGGGAGTAGGCTA
